# Lotka–Volterra approximations for evolutionary trait-substitution processes

**DOI:** 10.1007/s00285-020-01493-y

**Published:** 2020-05-21

**Authors:** Hiroshi C. Ito, Ulf Dieckmann, Johan A. J. Metz

**Affiliations:** 1 Evolution and Ecology Program, International Institute for Applied Systems Analysis, Schlossplatz 1, 2361 Laxenburg, Austria; 2 Department of Evolutionary Studies of Biosystems, The Graduate University for Advanced Studies (Sokendai), Hayama, 240-0193 Kanagawa Japan; 3 Mathematical Institute and Institute of Biology, Leiden University, P.O. Box 9512, 2300 RA Leiden, The Netherlands; 4 Naturalis Biodiversity Center, P.O. Box 9517, 2300 RA Leiden, The Netherlands

**Keywords:** Adaptive dynamics, Lotka–Volterra models, Lotka–Volterra approximations, Attractor inheritance, 92D15

## Abstract

A set of axioms is formulated characterizing ecologically plausible community dynamics. Using these axioms, it is proved that the transients following an invasion into a sufficiently stable equilibrium community by a mutant phenotype similar to one of the community's finitely many resident phenotypes can always be approximated by means of an appropriately chosen Lotka–Volterra model. To this end, the assumption is made that similar phenotypes in the community form clusters that are well-separated from each other, as is expected to be generally the case when evolution proceeds through small mutational steps. Each phenotypic cluster is represented by a single phenotype, which we call an approximate phenotype and assign the cluster’s total population density. We present our results in three steps. First, for a set of approximate phenotypes with arbitrary equilibrium population densities before the invasion, the Lotka–Volterra approximation is proved to apply if the changes of the population densities of these phenotypes are sufficiently small during the transient following the invasion. Second, quantitative conditions for such small changes of population densities are derived as a relationship between within-cluster differences and the leading eigenvalue of the community’s Jacobian matrix evaluated at the equilibrium population densities before the invasion. Third, to demonstrate the utility of our results, the ‘invasion implies substitution’ result for monomorphic populations is extended to arbitrarily polymorphic populations consisting of well-recognizable and -separated clusters.

## Introduction

Ecological interactions create selection pressures that may change those very interactions. Such eco-evolutionary feedback can induce rich coevolutionary dynamics including cyclic coevolution (e.g., Dieckmann et al. 1995; Dieckmann and Law 1996), adaptive radiation (e.g., Ackermann and Doebeli 2004; Egas et al. 2005), adaptive speciation (e.g., Dieckmann and Doebeli 1999; Dieckmann et al. 2004; Rundle and Nosil 2004), taxon cycles (e.g., Kisdi et al. 2001; Ito and Dieckmann 2007), and community formation (e.g., Loeuille and Loreau 2005; Dieckmann et al. 2007; Ito et al. 2009; Takahashi et al. 2013). To arrive at tractable descriptions of such evolutionary dynamics, the assumption is often made that mutation rates are low relative to the timescale of population dynamics. This assumption reduces the evolutionary dynamics to a trait-substitution sequence resulting from repeated mutant invasions (Metz et al. 1992, 1996; Dieckmann and Law 1996). Such invasions potentially bring about various outcomes: most often, (1) extinction of only the resident that is parental to the mutant, and more rarely, (2) coexistence of the mutant with all residents, or (3) other combinations of extinctions of the parental resident, non-parental residents, and mutant.

It has been proved that when for all residents all potentially invading mutants are subject to directional selection and the resulting perturbations to the system are sufficiently weak, as measured by the product of fitness gradients and mutational step sizes relative to the return rate to their population-dynamical equilibrium before the invasion, invading mutants replace their parental residents—a statement referred to as the invasion–implies–substitution theorem (Geritz 2005; Dercole and Rinaldi 2008). The resulting trait-substitution sequences describe directional coevolution, characterized well by a set of ordinary differential equations called the canonical equations of adaptive dynamics theory (Dieckmann and Law 1996), which have a form similar to Lande’s equations of quantitative genetics theory (Lande 1979).

Eventually, directional coevolution may take some residents to the neighborhood of peaks, troughs, or saddles of the community’s fitness landscape, which means that those populations experience very weak directional selection. Here, an invading mutant may coexist with its parental resident, which may be followed by diversifying evolution of the two morphs, called evolutionary branching (Metz et al. 1996). If the community has a one-dimensional trait space and a single resident, necessary and sufficient conditions for its evolutionary branching into two distinct residents have been obtained (Metz et al. 1996; Geritz et al. 1998).

On the other hand, for higher-dimensional traits or more than one resident, obtaining formal conditions for the occurrence of evolutionary branching is difficult (but see Ito and Dieckmann (2014) for a special case). This is largely because in these more complex community dynamics it is not easy to analyze the outcomes of mutant invasions (Metz et al. 1996). This difficulty may be reduced when the population dynamics can be approximated by Lotka–Volterra (LV) models, which are analytically more tractable and have been studied well (e.g., Zeeman 1993; Hofbauer and Sigmund 1998). The LV-approximation is possible when all existing residents and the mutant are similar to each other, so that they form a single phenotypic cluster (Meszéna et al. 2005; Durinx et al. 2008), which yields an expression for the invasion-fitness function in terms of resident and mutant phenotypes that is given by a rational function. By using this rational form, considerable progress in deriving conditions for multidimensional evolutionary branching has recently been made (Geritz et al. 2016; Sect. 9.3).

Dercole and Rinaldi (2008) proved that the LV-approximation holds also when all of the existing residents are not similar to each other, i.e., when every cluster has only a single resident, and their initial equilibrium population densities are not small. (Although such limiting assumptions for residents are not made in their proof, these assumptions are required when we consider trait-substitution sequences, as explained in Sect. 4.4). Thus, the remaining cases to be analyzed are (a) only some residents are similar to each other and (b) the population densities of some residents are very small so that they may go extinct as a result of the invasion. Both cases are likely to occur in multispecies coevolution, including processes involving multiple evolutionary branching and taxon cycles, commonly observed in numerical simulations of trait-mediated community dynamics (e.g., Doebeli and Dieckmann 2000; Ito and Dieckmann 2007). Therefore, the goal of the present paper is to obtain formal conditions for ensuring the LV-approximation for an arbitrary set of residents, including the aforementioned two cases. Based on the obtained conditions, the invasion–implies–substitution theorem can be extended to a mutant with an arbitrary set of residents.

The next section, Sect. 2, formulates a set of axioms that are expected to hold for ecologically plausible differential equations describing trait-mediated community dynamics. Section 3 derives a condition for ensuring the LV-approximation. Sections 4 and 5 derive sufficient conditions for satisfying this condition, in terms of properties of the fitness-generating function and mutational step sizes. Section 6 explains how the thresholds for the obtained sufficient conditions can be improved further. Section 7 shows how to examine the obtained sufficient conditions for a specific ecological model. Section 8 extends the invasion–implies–substitution theorem.

## Framework and assumptions

### Axioms for fitness-generating functions

We consider community dynamics written as

2.1 $$ \frac{{{\text{d}}n_{i} }}{{{\text{d}}t}} = n_{i} F(s_{i} ;{\mathbf{s}};{\mathbf{n}}) $$

with population densities $n_{i}$ for $i = 1, \ldots ,N$.

We denote by $\mathcal{\mathcal{S}} \subset {\mathbb{R}}^{Z}$ a compact $Z$-dimensional trait space, by ${\mathbf{s}} = (s_{1} , \ldots ,s_{N} )^{\text{T}} \in \mathcal{\mathcal{S}}^{N}$ an $N$-dimensional vector of trait values of the phenotypes present in the community, and by ${\mathbf{n}} = (n_{1} , \ldots ,n_{N} )^{\text{T}} \in {\mathbb{R}}_{ + }^{N}$ the vector of their population densities. The fitness-generating function

2.2 $$ F:\mathcal{\mathcal{S}} \times \bigcup\limits_{N = 1}^{\infty } {\left( {\mathcal{\mathcal{S}}^{N} \times {\mathbb{R}}^{N} } \right)} \to {\mathbb{R}}:(s^{{\prime }} ,{\mathbf{s}},{\mathbf{n}}) \mapsto F(s^{{\prime }} ;{\mathbf{s}};{\mathbf{n}}) $$

describes the instantaneous per capita growth rate of an arbitrary phenotype $s^{{\prime }}$ with an infinitesimally small population density in the instantaneous environment produced by resident community composition $({\mathbf{s}},{\mathbf{n}})$ (Brown and Vincent 1987; Cohen et al. 1999). The fitness-generating function provides a fitness landscape for each community composition $({\mathbf{s}},{\mathbf{n}})$. We assume that it satisfies the following axioms:

(i) *Smoothness*: $F$ is smooth on each component of its domain $\mathcal{\mathcal{S}} \times \mathcal{\mathcal{S}}^{N} \times {\mathbb{R}}^{N} .$
(ii) *Symmetry*: $F(s^{{\prime }} ;\sigma {\mathbf{s}};\sigma {\mathbf{n}}) = F(s^{{\prime }} ;{\mathbf{s}};{\mathbf{n}})$ for all permutations $\sigma$ operating on the indices of $({\mathbf{s}};{\mathbf{n}}).$
(iii) *Reducibility*: $$ F(s^{{\prime }} ;(s_{1} , \ldots ,s_{N} )^{\text{T}} ;(n_{1} , \ldots ,n_{N - 1} ,0)^{\text{T}} ) = F(s^{{\prime }} ;(s_{1} , \ldots ,s_{N - 1} )^{\text{T}} ;(n_{1} , \ldots ,n_{N - 1} )^{\text{T}} ). $$
(iv) *Exchangeability*: If $$ s_{N} = s_{N - 1} $$, then $$ F(s^{{\prime }} ;(s_{1} , \ldots ,s_{N} )^{\text{T}} ;(n_{1} , \ldots ,n_{N} )^{\text{T}} ) $$$$ = F(s^{{\prime }} ;(s_{1} , \ldots ,s_{N - 1} )^{\text{T}} ;(n_{1} , \ldots ,n_{N - 1} + n_{N} )^{\text{T}} ). $$
(v) *Bounded world*: There exists an upper bound $\eta > 0$ for the community's total population density, i.e., Eq. (2.1) eventually restricts the population densities to $\left\{ {(n_{1} , \ldots ,n_{N} ) \in {\mathbb{R}}_{ + }^{N} \left| {\sum\nolimits_{i = 1}^{N} {n_{i} } \le \eta } \right.} \right\}.$

Below, we restrict the community's space of population densities to $[0,\eta ]^{N} .$

The smoothness axiom (i) follows from the assumption that the population-dynamical behavior of individuals depends smoothly on their traits and that all ecological interactions are instantaneous. The latter assumption is implicit in the assumption that the per capita growth rate depends only on the arguments $s^{{\prime }}$ and $({\mathbf{s}},{\mathbf{n}})$. Axioms (ii) to (iv) are consistency conditions that go with representing the behaviour of large collectives of individuals by differential equations for their densities. Axiom (ii) follows from the arbitrariness of the ordering of the trait *N*-tuples, and axiom (iv) from the fact that individuals with the same trait values are assumed to be indistinguishable. The consequent additivity for identical phenotypes mechanistically lies at the heart of the LV-approximability. The bounded-world axiom (v) is just what it says: there necessarily is a limit to the biomass that a patch of world can support. Models that do not acknowledge this may on occasion be good approximations for specific purposes, but when we run into results contradicting the bounded-world assumption, we have to start modifying the model.

To keep the exposition simple, we assume from now on a one-dimensional trait space $\mathcal{\mathcal{S}} \subset {\mathbb{R}}$. The results are generalized to higher-dimensional trait spaces in Sect. 5.4.

### Population dynamics triggered by mutant invasion

We assume that the community is at a locally stable equilibrium $$ \overset{\lower0.5em\hbox{$\smash{\scriptscriptstyle\frown}$}}{\mathbf{n}}= ( \overset{\lower0.5em\hbox{$\smash{\scriptscriptstyle\frown}$}}{n}_{1} , \ldots , \overset{\lower0.5em\hbox{$\smash{\scriptscriptstyle\frown}$}}{n}_{N} )^{\text{T}}  $$, determined by $$ F(s_{i} ;{\mathbf{s}};\overset{\lower0.5em\hbox{$\smash{\scriptscriptstyle\frown}$}}{\mathbf{n}}) = 0 $$ for all $$ i = 1,\ldots,N $$. When an invasion by a mutant $$ s^{{\prime }} = s_{N + 1} $$ with $$ \left| {s_{N + 1} - s_{N} } \right| = \varepsilon_{\mu } $$ has occurred, the combined population dynamics can be written as

2.3 $$ \frac{{{\text{d}}n_{i} }}{{{\text{d}}t}} = n_{i} F(s_{i} ;{\mathbf{s^{\prime}}};{\mathbf{n^{\prime}}}) $$

for $$ i = 1, \ldots ,N + 1 $$, where $$ {\mathbf{s}}^{{\prime }} = (s_{1} , \ldots ,s_{N} ,s_{N + 1} )^{\text{T}} $$ and $$ {\mathbf{n}}^{{\prime }} = (n_{1} , \ldots ,n_{N} ,n_{N + 1} )^{\text{T}} $$, starting from $$ {\mathbf{n}}^{{\prime }} = ( \overset{\lower0.5em\hbox{$\smash{\scriptscriptstyle\frown}$}}{n}_{1} , \ldots , \overset{\lower0.5em\hbox{$\smash{\scriptscriptstyle\frown}$}}{n}_{N} ,n_{N + 1} )^{\text{T}} $$ with very small $$ n_{N + 1} $$, which means that $$ {\mathbf{n}}^{{\prime }} $$ is almost identical to the equilibrium before the invasion, $$ \overset{\lower0.5em\hbox{$\smash{\scriptscriptstyle\frown}$}}{\mathbf{n}}^{\prime } = ( \overset{\lower0.5em\hbox{$\smash{\scriptscriptstyle\frown}$}}{n}_{1} , \ldots , \overset{\lower0.5em\hbox{$\smash{\scriptscriptstyle\frown}$}}{n}_{N} ,0)^{\text{T}} $$.

Please notice that here we have introduced the notational convention, to which we adhere throughout this paper, that vectors of dimension *N* + 1 directly corresponding to vectors of dimension *N* are denoted by an added prime, as in $$ {\mathbf{s}}^{{\prime }} $$, $$ {\mathbf{n}}^{{\prime }} $$, and $$\overset{{\lower0.5em\hbox{$\smash{\scriptscriptstyle\frown}$}}}{{{\textbf n}}}^{\prime}$$.

#### **Proposition 1**

*For a sufficiently small mutational step size*$\varepsilon_{\mu }$*, the fitness*-*generating function during the transient following mutant invasion can be approximated by a linear function of*${\mathbf{n}}^{{\prime }}$,

2.4 $$ \begin{aligned} & F(s_{i} ;{\mathbf{s}}^{{\prime }} ;{\mathbf{n}}^{{\prime }} ) =  \overset{\lower0.5em\hbox{$\smash{\scriptscriptstyle\frown}$}}{F}_{i} + \sum\limits_{j = 1}^{N + 1} {a_{ij} (n_{j} -  \overset{\lower0.5em\hbox{$\smash{\scriptscriptstyle\frown}$}}{n}_{j} )} , \\ &  \overset{\lower0.5em\hbox{$\smash{\scriptscriptstyle\frown}$}}{F}_{i} : = F(s_{i} ;{\mathbf{s}}^{{\prime }} ;\overset{\lower0.5em\hbox{$\smash{\scriptscriptstyle\frown}$}}{\mathbf{n}}^{\prime} ), \\ & a_{ij} : = \left. {\frac{{\partial F(s_{i} ;{\mathbf{s}}^{{\prime }} ;{\mathbf{n}}^{{\prime }} )}}{{\partial n_{j} }}} \right|_{{{\mathbf{n^{\prime}}} = \overset{\lower0.5em\hbox{$\smash{\scriptscriptstyle\frown}$}}{\mathbf{n}}^{\prime}}} , \\ \end{aligned} $$

*which upon substitution into Eq.* (2.3) *gives the approximating Lotka*–*Volterra model,*

2.5 $$ \frac{{{\text{d}}n_{i} }}{{{\text{d}}t}} = n_{i} \left[ {\gamma_{i} + \sum\limits_{j = 1}^{N + 1} {a_{ij} n_{j} } } \right] $$

*with*$$ \gamma_{i} =  \overset{\lower0.5em\hbox{$\smash{\scriptscriptstyle\frown}$}}{F}_{i} - \sum\nolimits_{j = 1}^{N + 1} {a_{ij}  \overset{\lower0.5em\hbox{$\smash{\scriptscriptstyle\frown}$}}{n}_{j} } . $$

The remainder of this paper is devoted to making precise the, very general, conditions under which this proposition holds, and to calculating the corresponding error bounds. Important variables and parameters used in our analysis are shown in Table 1.

**Table 1:** List of notation for parameters, phenotypes, population densities, fitness functions, and other quantities

Parameter	Formula	Explanation	Location
*Important parameters*			
$\eta$		Upper bound for the total population density a community can sustain	Axiom (v)
$\varepsilon_{\mu }$		Phenotypic distance of a mutant phenotype from its parental resident phenotype	Above Eq. (2.3)
$\varepsilon$	$= \rho_{\mu } \varepsilon_{\mu } > \varepsilon_{\mu }$	Threshold phenotypic distance for clustering phenotypes	Above Eq. (3.1a)
$\rho_{\text{m}}$		Threshold for treating population densities less than $\rho_{\text{m}} \varepsilon$ as small	Above Eq. (5.2a)
$N$		Number of resident phenotypes before mutant invasion	Eq. (2.1)
$M$	$< N$	Number of phenotypic clusters described by approximate phenotypes	Above Eq. (3.1a)
$L$	$\le M$	Number of approximate phenotypes with not-small population densities	Above Eq. (5.2a)
$K$	$= M - L \le M$	Number of approximate phenotypes with small population densities	Below Eq. (5.8c)
$d$		Positive constant in Lyapunov function	Eq. (5.5c)

Phenotype	Formula	Explanation	Location
*Phenotypes*			
${\mathbf{s}}$	$= (s_{1} , \ldots ,s_{N} )^{\text{T}}$	Phenotypes before mutant invasion	
$s^{{\prime }}$	$= s_{N + 1} = s_{N} + \varepsilon_{\mu }$	Mutant phenotype	
${\mathbf{s}}^{{\prime }}$	$= (s_{1} , \ldots ,s_{N} ,s_{N + 1} )^{\text{T}}$	Phenotypes after mutant invasion	
${\mathbf{s}}_{\text{a}}$	$= (s_{1} , \ldots ,s_{M} )^{\text{T}}$	Approximate phenotypes, which serve as the representative phenotypes of clusters	
$\rho_{j}$	$= (s_{j} - s_{{{\text{cid}}(j)}} ) /\varepsilon < 1$	Difference of the $j$th phenotype from its cluster’s approximate phenotype, scaled by $\varepsilon$	
${\varvec{\uprho}}$	$= (0, \ldots ,0,\rho_{M + 1} , \ldots ,\rho_{N + 1} )^{\text{T}}$	Vector of the differences of all phenotypes from their clusters’ approximate phenotypes, scaled by $\varepsilon$	
${\text{cid}}(j)$	$\in \left\{ {1, \ldots ,M} \right\}$	Identity of cluster to which the $j$th phenotype belongs	
${\text{com}}(i)$	$= \{ \left. j \right|\,{\text{cid}}(j) = i,j = 1, \ldots ,N + 1\}$	Set of identities of phenotypes that belong to the $i$th cluster	

Densities	Formula	Explanation	Location
*Population densities*			
${\mathbf{n}}$	$= (n_{1} , \ldots ,n_{N} )^{\text{T}}$	Population densities before mutant invasion	Eq. (2.1)
$$ \overset{\lower0.5em\hbox{$\smash{\scriptscriptstyle\frown}$}}{n} $$	$$ = ( \overset{\lower0.5em\hbox{$\smash{\scriptscriptstyle\frown}$}}{n}_{1} , \ldots , \overset{\lower0.5em\hbox{$\smash{\scriptscriptstyle\frown}$}}{n}_{N} )^{\text{T}} $$	Equilibrium population densities before mutant invasion	Below Eq. (2.3)
$n_{N + 1}$		Mutant population density	Below Eq. (2.3)
${\mathbf{n}}^{{\prime }}$	$= (n_{1} , \ldots ,n_{N} ,n_{N + 1} )^{\text{T}}$	Population densities after mutant invasion	Below Eq. (2.3)
$$ \overset{\lower0.5em\hbox{$\smash{\scriptscriptstyle\frown}$}}{\mathbf{n}}^{{\prime }} $$	$$ = ( \overset{\lower0.5em\hbox{$\smash{\scriptscriptstyle\frown}$}}{n}_{1} , \ldots , \overset{\lower0.5em\hbox{$\smash{\scriptscriptstyle\frown}$}}{n}_{N} ,0)^{\text{T}} $$		Below Eq. (2.3)
${\mathbf{m}}$	$= (m_{1} , \ldots ,m_{M} )^{\text{T}}$	Population densities of approximate phenotypes ${\mathbf{s}}_{\text{a}}$	Above Eq. (3.1a)
$m_{i}$	$= \sum\limits_{{j \in {\text{com}}(i)}} {n_{j} }$	Total population density of $i$th cluster, assigned to the $i$th approximate phenotype	Eq. (3.1a)
$(m_{M + 1} , \ldots ,m_{N + 1} )$	$= (\varepsilon n_{M + 1} , \ldots ,\varepsilon n_{N + 1} )$		Eq. (3.1b)
${\mathbf{m}}^{{\prime }}$	$= (m_{1} , \ldots ,m_{M} ,m_{M + 1} , \ldots ,m_{N + 1} )^{\text{T}}$		Above Eq. (3.3a)
$$ \overset{\lower0.5em\hbox{$\smash{\scriptscriptstyle\frown}$}}{\mathbf{m}}^{{\prime }} $$	$$ \begin{aligned} & = ( \overset{\lower0.5em\hbox{$\smash{\scriptscriptstyle\frown}$}}{m}_{1} , \ldots , \overset{\lower0.5em\hbox{$\smash{\scriptscriptstyle\frown}$}}{m}_{N + 1} )^{\text{T}} \\ & = ( \overset{\lower0.5em\hbox{$\smash{\scriptscriptstyle\frown}$}}{m}_{1} , \ldots , \overset{\lower0.5em\hbox{$\smash{\scriptscriptstyle\frown}$}}{m}_{M} ,\varepsilon \overset{\lower0.5em\hbox{$\smash{\scriptscriptstyle\frown}$}}{n}_{M + 1} , \ldots ,\varepsilon \overset{\lower0.5em\hbox{$\smash{\scriptscriptstyle\frown}$}}{n}_{N} ,0)^{\text{T}} \\ \end{aligned} $$		Above Eq. (3.3a)
$$ \overset{\lower0.5em\hbox{$\smash{\scriptscriptstyle\frown}$}}{m}$$	$$ = ( \overset{\lower0.5em\hbox{$\smash{\scriptscriptstyle\frown}$}}{m}_{1} , \ldots , \overset{\lower0.5em\hbox{$\smash{\scriptscriptstyle\frown}$}}{m}_{M} )^{\text{T}} $$		Eq. (3.4a)
$$ {\mathbf{x}} $$ (in Sect. 4)	$$ = (x_{1} , \ldots ,x_{M} )^{\text{T}} = {\mathbf{P}}({\mathbf{m}} - \overset{\lower0.5em\hbox{$\smash{\scriptscriptstyle\frown}$}}{\mathbf{m}}$$	Transformed vectors of $$ {\mathbf{m}} $$ for stability analysis	Eq. (4.4)
${\mathbf{m}}_{\text{x}}$	$= (m_{1} , \ldots ,m_{L} )^{\text{T}}$	Approximate phenotypes with not-small initial population densities	Eq. (5.2a)
${\mathbf{m}}_{\text{y}}$	$= (m_{L + 1} , \ldots ,m_{M} )^{\text{T}}$	Approximate phenotypes with small initial population densities	Eq. (5.2a)
$$ {\tilde{\mathbf{m}}}_{\text{x}} ({\mathbf{m}}_{\text{y}} ) $$	$$ = \overset{\lower0.5em\hbox{$\smash{\scriptscriptstyle\frown}$}}{\mathbf{m}}_{\text{x}} - {\mathbf{B}}_{\text{xx}}^{ - 1} {\mathbf{B}}_{\text{xy}} {\mathbf{m}}_{\text{y}} $$	Center manifold	Eq. (5.3b)
$$ {\overset{\lower0.5em\hbox{$\smash{\scriptscriptstyle\frown}$}}{\tilde{\mathbf{m}} }} $$	$$ = ({\overset{\lower0.5em\hbox{$\smash{\scriptscriptstyle\frown}$}}{{\tilde{\mathbf{m}}} }}_{\text{x}} ,{\overset{\lower0.5em\hbox{$\smash{\scriptscriptstyle\frown}$}}{\tilde{\mathbf{m}}}}_{\text{y}} )^{\text{T}} = ({{\overset{\lower0.5em\hbox{$\smash{\scriptscriptstyle\frown}$}}{{\mathbf{m}}} }}_{\text{x}} ,{\mathbf{0}})^{\text{T}} $$	Modified equilibrium	Above Eq. (5.4a)
${\mathbf{x}}$ (in Sect. 5)	$$ \begin{aligned} & = (x_{1} , \ldots ,x_{L} )^{\text{T}} \\ & = {\mathbf{P({m}}}_{{\mathrm{x}}}-{\tilde{\mathbf{m}}}_{{\mathrm{x}}}(\mathbf{m}_{\mathrm{y}})) \\ \end{aligned} $$	Transformed vectors of ${\mathbf{m}_{\mathrm{x}}-{\tilde{\mathbf{m}}_{\mathrm{x}}}}$ for stability analysis	Eq. (5.4a)
${\mathbf{y}}$	$= {{\mathbf{m}}}_{\text{y}}$		Eq. (5.4a)
${\mathbf{w}}$	$= ({\mathbf{x}},{\mathbf{y}})^{\text{T}}$		Eq. (5.4a)

Fitness	Formula	Explanation	Location
*Fitness functions*			
$F(s_{i} ;{\mathbf{s}}^{{\prime }} ;{\mathbf{n}}^{{\prime }} )$	$= \frac{1}{{n_{i} }}\frac{{{\text{d}}n_{i} }}{{{\text{d}}t}}$	Per capita growth rate of phenotype $s_{i}$ in the environment determined by phenotypes ${\mathbf{s}}^{{\prime }}$ with population densities ${\mathbf{n}}^{{\prime }}$	Eqs. (2.1), (2.3)
$$ \overset{\lower0.5em\hbox{$\smash{\scriptscriptstyle\smile}$}}{F} (s_{i} ;{\mathbf{s}}^{{\prime }} ;{\mathbf{m}}^{{\prime }} ) $$	$$ = F(s_{i} ;{\mathbf{s}}^{{\prime }} ;{\mathbf{n}}^{{\prime }} ) $$		Eq. (3.2)
$f(s_{i} ;{\mathbf{s}}^{{\prime }} ;{\mathbf{n}}^{{\prime }} )$	$= \sum\limits_{{j \in {\text{com(}}i )}} {\frac{{n_{j} }}{{m_{i} }}F(s_{i} ;{\mathbf{s}}^{{\prime }} ;{\mathbf{n}}^{{\prime }} )}$	Per capita growth rate of approximate phenotype $s_{i}$ in the environment determined by phenotypes ${\mathbf{s}}^{{\prime }}$ with population densities ${\mathbf{n}}^{{\prime }}$	Eq. (4.1a)

Quantity	Formula or explanation	Location
*Other quantities*		
$$ \overset{\lower0.5em\hbox{$\smash{\scriptscriptstyle\frown}$}}{F}_{i} $$	$$ = F(s_{i} ;{\mathbf{s}}^{{\prime }} ;{{\overset{\lower0.5em\hbox{$\smash{\scriptscriptstyle\frown}$}}{{\mathbf{n}}^{{\prime }}} }} ) $$	Eq. (2.4)
$a_{ij}$	$= \frac{{\partial F(s_{i} ;{\mathbf{s}}^{{\prime }} ;{\mathbf{n}}^{{\prime }} )}}{{\partial n_{j}^{{\prime }} }}$	Eq. (2.4)
${\mathbf{a}}_{i}^{\text{T}}$	$= \left( {a_{i,1} , \ldots ,a_{i,N + 1} } \right)$	Eq. (3.5a)
${\mathbf{W}}$	$= \frac{{\partial {\mathbf{m}}^{{\prime }} }}{{\partial {\mathbf{n}}^{{\prime }} }}$	Eq. (3.5a)
$$ b_{ij}^{{\prime }} $$	$$ \left. { = \frac{{\partial \overset{\lower0.5em\hbox{$\smash{\scriptscriptstyle\smile}$}}{F} (s_{i} ;{\mathbf{s}}^{{\prime }} ;{\mathbf{m}}^{{\prime }} )}}{{\partial m_{j} }}} \right|_{{{\mathbf{m}}^{{\prime }} = {{\overset{\lower0.5em\hbox{$\smash{\scriptscriptstyle\frown}$}}{{\mathbf{m}}^{{\prime }}} }} }} $$	Eq. (3.3c)
${\mathbf{b}}_{i}^{{{\prime }{\text{T}}}}$	$= \left( {b_{i1}^{{\prime }} , \ldots ,b_{i\,N + 1}^{{\prime }} } \right)$	Eq. (3.3c)
$$ b_{ij} $$	$$ \left. {\frac{{\partial \overset{\lower0.5em\hbox{$\smash{\scriptscriptstyle\smile}$}}{F} (s_{i} ;{\mathbf{s}}_{\text{a}} ;{\mathbf{m}})}}{{\partial m_{j} }}} \right|_{{{\mathbf{m}} = {{\overset{\lower0.5em\hbox{$\smash{\scriptscriptstyle\frown}$}}{{\mathbf{m}}} }}}} $$	Eq. (4.3b)
${\mathbf{B}}$	$$ = \left( {\begin{array}{*{20}l} {b_{11} } \hfill & \cdots \hfill & {b_{1M} } \hfill \\ \vdots \hfill & \ddots \hfill & \vdots \hfill \\ {b_{M1} } \hfill & \cdots \hfill & {b_{MM} } \hfill \\ \end{array} } \right) $$	Eq. (4.3b)
$$ {\mathbf{J}} $$	$$ = {\text{diag}}({{\overset{\lower0.5em\hbox{$\smash{\scriptscriptstyle\frown}$}}{{\mathbf{m}}} }}){\mathbf{B}} $$	Eq. (4.3b)
${\mathbf{A}}$	$= {\mathbf{PJP}}^{ - 1}$	Eq. (4.4)
${\mathbf{P}}$ (in Sect. 4)	Matrix for transforming ${\mathbf{J}}$ for Lemma 4	Eq. (4.4)
$\lambda_{\rm max}$	Leading eigenvalue of ${\mathbf{J}}$, possibly adjusted for repeated eigenvalues	Eq. 4.5a)
${\mathbf{B}}_{\text{xx}} ,{\mathbf{B}}_{\text{xy}} ,{\mathbf{B}}_{\text{yx}} ,{\mathbf{B}}_{\text{yy}}$	$$ \left( {\begin{array}{*{20}l} {{\mathbf{B}}_{\text{xx}} } \hfill & {{\mathbf{B}}_{\text{xy}} } \hfill \\ {{\mathbf{B}}_{\text{yx}} } \hfill & {{\mathbf{B}}_{\text{yy}} } \hfill \\ \end{array} } \right) = {\mathbf{B}} $$	Eq. (5.2c)
$$ {\mathbf{A}}_{\text{x}} $$	$$ = {\mathbf{PJ}}_{\text{x}} {\mathbf{P}}^{ - 1} = {\mathbf{P}}{\text{diag(}}{{\overset{\lower0.5em\hbox{$\smash{\scriptscriptstyle\frown}$}}{{\mathbf{m}}} }}_{{\mathbf{x}}} ){\mathbf{B}}_{{{\mathbf{xx}}}} {\mathbf{P}}^{ - 1} $$	Eq. (5.4c)
${\mathbf{P}}$ (in Sect. 5)	Matrix for transforming ${\mathbf{J}}_{\text{x}}$ for Lemma 4 with ${\mathbf{A}} = {\mathbf{A}}_{\text{x}}$	Eq. (5.4c)
${\mathbf{U}}$	$= {\mathbf{B}}_{\text{yx}} {\mathbf{P}}^{ - 1}$	Eq. (5.4c)
${\mathbf{J}}_{\text{y}}$	$= {\mathbf{B}}_{\text{yy}} - {\mathbf{B}}_{\text{yx}} {\mathbf{B}}_{\text{xx}}^{ - 1} {\mathbf{B}}_{\text{xy}}$	Eq. (5.4c)
${\tilde{\mathbf{A}}}$	$$ = \left( {\begin{array}{*{20}c} {{\mathbf{A}}_{\text{x}} } & 0 \\ {d{\mathbf{U}}} & {d{\mathbf{J}}_{\text{y}} } \\ \end{array} } \right) $$	Eq. (5.5a)
$\tilde{\lambda }_{\rm {max} }$	Leading eigenvalue of $\tfrac{1}{2}({\tilde{\mathbf{A}}} + {\tilde{\mathbf{A}}}^{\text{T}} )$	Eq. (5.5b)
${\mathbf{Q}}$	$$ = \left( {\begin{array}{*{20}l} {{\mathbf{P}}_{{\mathbf{x}}} } \hfill & {{\mathbf{P}}_{{\mathbf{x}}} {\mathbf{B}}_{\text{xx}}^{ - 1} {\mathbf{B}}_{\text{xy}} } \hfill \\ {\mathbf{0}} \hfill & {{\mathbf{I}}_{\text{y}} } \hfill \\ \end{array} } \right) $$	Eq. (5.9b)

## Linear approximation of the fitness-generating function

### Basic idea

The root of the LV-approximability is the exchangeability axiom (iv) combined with the smoothness axiom (i). Under the exchangeability axiom (iv), the fitness-generating function does not distinguish individuals with identical phenotypes. Hence, the function responds only to the sum of their densities. Under the smoothness axiom (i), this property is approximately inherited by slightly different phenotypes; the fitness-generating function responds primarily to the sum of their densities. In the remainder of this paper, we will work out how to lowest order of approximation the fitness-generating function responds linearly to the separate contributions to this sum, leading to the LV-approximation.

To get a more specific picture, we first suppose that there exist only two phenotypes, a resident phenotype $$ s_{1} $$ and a mutant phenotype $$ s_{2} $$, with population densities $$ n_{1} $$ and $$ n_{2} $$, respectively, and with their phenotypic difference given by the mutational step size, $$ |s_{2} - s_{1} | = \varepsilon_{\mu } $$, with $$ \varepsilon_{\mu } $$ being small. Proposition 1 trivially holds when the deviations of $$ n_{1} $$ and $$ n_{2} $$ from their initial states $$ {{\overset{\lower0.5em\hbox{$\smash{\scriptscriptstyle\frown}$}}{{\mathbf{n}}^{{\prime }}} }} = (\overset{\lower0.5em\hbox{$\smash{\scriptscriptstyle\frown}$}}{n}_{1} ,\overset{\lower0.5em\hbox{$\smash{\scriptscriptstyle\frown}$}}{n}_{2} )^{\text{T}} $$ are both small during the transient following mutant invasion. In many cases, however, those changes are large, resulting in the exclusion of the resident (Dercole and Rinaldi 2008, Appendix B). In the latter case, it is not obvious whether a linear approximation of the fitness-generating function in $$ {\mathbf{n}}^{{\prime }} = (n_{1} ,n_{2} )^{\text{T}} $$ is valid.

On the other hand, as the mutant is similar to the resident, due to the smoothness and exchangeability property of the fitness-generating function, they act almost like a single phenotype in their effect on the environment. Thus, invasion by the mutant in many cases causes only a slight change in their total population density $n_{1} + n_{2}$, and only their fractions may change substantially, but will do so slowly (Dercole and Rinaldi 2008, Appendix B; Meszéna et al. 2005; Durinx et al. 2008). In other words, the fitness-generating function is not sensitive to even large changes of $n_{1}$ and $n_{2}$, as long as $n_{1} + n_{2}$ is kept almost constant. As shown later, this implies that the change of $F(s_{i} ;{\mathbf{s}}^{{\prime }} ;{\mathbf{n}}^{{\prime }} )$ induced by a large change of $n_{2}$, keeping $n_{1} + n_{2}$ constant, is slight, so that $F(s_{i} ;{\mathbf{s}}^{{\prime }} ;{\mathbf{n}}^{{\prime }} )$ can be expanded with respect to ${\mathbf{m}}^{{\prime }} = (m_{1} ,m_{2} )^{\text{T}} = (n_{1} + n_{2} ,\varepsilon_{\mu } n_{2} )^{\text{T}}$, even for $\varepsilon_{\mu } \to 0$. The linear relationship between ${\mathbf{m}}^{{\prime }}$ and ${\mathbf{n^{\prime}}}$ then makes Proposition 1 hold: as the change of $m_{2} = \varepsilon_{\mu } n_{2}$ is always small because of the smallness of $\varepsilon_{\mu }$, this is the case whenever the change of the population density $m_{1} = n_{1} + n_{2}$ is small. Below, we introduce the notion of approximate phenotypes, so we can abbreviate the preceding condition by stating that the change in the population density $m_{1} = n_{1} + n_{2}$ of the approximate phenotype ($s_{\text{a}} = s_{1}$ or $s_{\text{a}} = s_{2}$) is small.

The strategy above is readily extended to multiple residents $s_{1} , \ldots ,s_{N}$ and a mutant $s_{N + 1}$ emerged from the parental phenotype $s_{N}$ with $|s_{N + 1} - s_{N} | = \varepsilon_{\mu }$, by choosing an approximate phenotype from each of the existing phenotypic clusters, so that density changes of those approximate phenotypes can be kept small during the transient following mutant invasion, and thus an LV-approximation can be warranted (Sect. 3). We can gauge the smallness of their density changes from the leading eigenvalue of the community’s Jacobian matrix evaluated at the equilibrium population densities of the approximate phenotypes before the invasion (Sect. 4). However, this linear stability analysis does not work well when some approximate phenotypes have very small initial equilibrium densities, because those small densities inevitably cause the leading eigenvalue of the community’s Jacobian matrix to be close to zero. To overcome this difficulty, we analyze not only the linear terms but also the quadratic terms of the transient dynamics around the initial equilibrium (Sect. 5). In the remainder of this section, we show how we can easily find approximate phenotypes for a set of phenotypes ${\mathbf{s}}^{{\prime }} = (s_{1} , \ldots ,s_{N + 1} )^{\text{T}}$, such that Proposition 1 holds when the changes of the population densities of these approximate phenotypes are sufficiently small.

### Approximate phenotypes

**Fig. 1:**
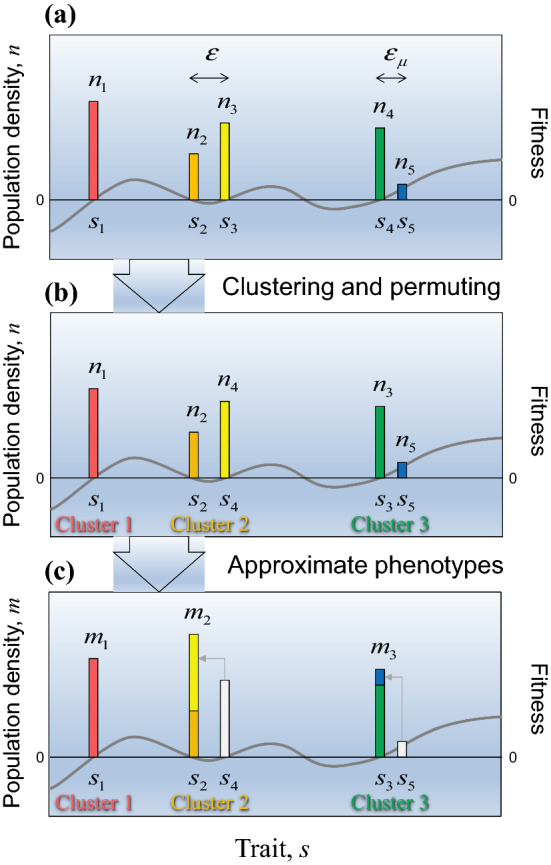
Construction of approximate phenotypes and their population densities. **a** The population densities ${\mathbf{n}}^{{\prime }} = (n_{1} ,n_{2} ,n_{3} ,n_{4} ,n_{5} )^{\text{T}}$ of existing phenotypes ${\mathbf{s}}^{{\prime }} = (s_{1} ,s_{2} ,s_{3} ,s_{4} ,s_{5} )^{\text{T}}$—comprising four residents $s_{1}$, $s_{2}$, $s_{3}$, $s_{4}$, and a mutant $s_{5}$—are indicated by colored histogram bars. The thick gray curve shows the fitness landscape, which passes through 0 at the resident phenotypes. The existing phenotypes are clustered so that within-cluster phenotypic differences do not exceed the threshold $\varepsilon$, which is chosen to be larger than the mutational step size $\varepsilon_{\mu }$, so that the mutant $s_{5}$ and its parental resident $s_{4}$ are guaranteed to be part of the same cluster. **b** The existing phenotypes are permuted so that the approximate phenotypes $s_{1}$, $s_{2}$, $s_{3}$ come first. **c** Within each cluster, an approximate phenotype is chosen to represent the cluster. The population densities $m_{1}$, $m_{2}$, $m_{3}$ of the approximate phenotypes are assigned so that each equals the total population density of the corresponding cluster (color figure online)

We consider an arbitrary set of residents together with a mutant, ${\mathbf{s}}^{{\prime }} = (s_{1} , \ldots ,s_{N} ,s_{N + 1} )^{\text{T}}$. We choose phenotypic clusters so that within-cluster phenotypic differences do not exceed $\varepsilon = \rho_{\mu } \varepsilon_{\mu }$ (Fig. 1a), with an arbitrarily chosen constant $\rho_{\mu }$ larger than 1 (but not too large, so that the clustering is meaningful, i.e., the error estimates to be derived below are small). We assume that those phenotypic clusters are well-recognizable and well-separated from each other, so that we can find an $\varepsilon$ that is much smaller than the smallest distance among the approximate phenotypes. Generally, this assumption is warranted in evolutionary dynamics with small mutational step sizes (as explained in Sect. 9.2) by the principle of limiting similarity. Notice that in any case the mutant $s_{N + 1}$ and its parental phenotype $s_{N}$ form a cluster. Any resident not similar to any other phenotype forms a cluster by itself. Thus, the number of clusters, denoted by $M$, satisfies $1 \le M \le N$. From each cluster, we arbitrarily pick one phenotype as its representative. Then, by the symmetry axiom (ii), we can permute ${\mathbf{s}}^{{\prime }} = (s_{1} , \ldots ,s_{N} ,s_{N + 1} )^{\text{T}}$ so that those representatives come first as $s_{1} , \ldots ,s_{M}$, followed by the other phenotypes, i.e., ${\mathbf{s}}^{{\prime }} = (s_{1} , \ldots ,s_{M} ,s_{M + 1} , \ldots ,s_{N + 1} )^{\text{T}}$ (Fig. 1b). We refer to those representatives as approximate phenotypes ${\mathbf{s}}_{\text{a}} = (s_{1} , \ldots ,s_{M} )^{\text{T}} .$

We introduce the cluster-identifying function ${\text{cid}}$, such that ${\text{cid}}(j) = i$ means that phenotype $s_{j}$ belongs to the $i$th cluster, with $s_{{{\text{cid}}(j)}}$ as the representative—i.e., approximate—phenotype of that cluster, and ${\text{cid}}(j) = j$ for $j \le M$. We also introduce the component-identifying function ${\text{com}}$, which returns the set of indices of the phenotypes comprising the *i*-th cluster, i.e., ${\text{com}}(i) = \left\{ {\left. j \right|\;{\text{cid}}(j) = i} \right\}$. Then, the population densities of these clusters are given by a vector ${\mathbf{m}} = (m_{1} , \ldots ,m_{M} )^{\text{T}}$, with the population densities

3.1a $$ m_{i} : = \sum\limits_{{j \in {\text{com}}(i)}} {n_{j} } $$

for $i = 1, \ldots ,M$ treated as belonging to the approximate phenotypes ${\mathbf{s}}_{\text{a}} = (s_{1} , \ldots ,s_{M} )^{\text{T}}$ (Fig. 1c). While the approximate phenotype of the $i$th cluster is identical to the representative phenotype of that cluster, the population densities of the former and latter are different and given by $m_{i}$ and $n_{i}$, respectively.

Notice that the number $M$ of approximate phenotypes is less than the number $N + 1$ of phenotypes in the original community dynamics. Thus, for expanding the fitness-generating function, we need to define the other $(N - M + 1)$ variables in such a way that their changes stay small during the transient following mutant invasion. As long as the population densities of the approximate phenotypes are kept almost constant, the fitness-generating function is expected to be insensitive to $n_{i}$ for all $i = 1,\ldots,N+1$. Thus, we describe the remaining degrees of freedom, $m_{M + 1} , \ldots ,m_{N + 1}$, by

3.1b $$ m_{i} : = \varepsilon n_{i} $$

for $i = M + 1, \ldots ,N + 1$. Combining Eqs. (3.1a) and (3.1b), we write ${\mathbf{m}}^{{\prime }} = (m_{1} , \ldots ,m_{M} ,m_{M + 1} , \ldots ,m_{N + 1} )^{\text{T}}$, which has the same dimension as ${\mathbf{n}}^{{\prime }}$. Then, by the smoothness axiom (i), the exchangeability axiom (iv), and the bounded-world axiom (v), we have

#### **Lemma 1**

*With*$$ \overset{\lower0.5em\hbox{$\smash{\scriptscriptstyle\smile}$}}{F} (s_{i} ;{\mathbf{s}}^{{\prime }} ;{\mathbf{m}}^{{\prime }} ): = F(s_{i} ;{\mathbf{s}}^{{\prime }} ;{\mathbf{n}}^{{\prime }} ) $$*, for sufficiently small*$$ \varepsilon $$*there exists a constant*$$ C_{\text{Fm}}^{{\prime }} $$*such that*

3.2 $$ \left| {\frac{{\partial \overset{\lower0.5em\hbox{$\smash{\scriptscriptstyle\smile}$}}{F} (s_{i} ;{\mathbf{s}}^{{\prime }} ;{\mathbf{m}}^{{\prime }} )}}{{\partial m_{j} }}} \right| \le C_{\text{Fm}}^{{\prime }} $$

*for all*$i,j = 1, \ldots ,N + 1$, ${\mathbf{n}}^{{\prime }} \in [0,\eta ]^{N + 1}$*, and any*${\mathbf{s}}^{{\prime }}$*such that*$\left| {s_{j} - s_{{{\text{cid}}(j)}} } \right| \le \varepsilon .$

See Appendix A for the proof. Although $$ \overset{\lower0.5em\hbox{$\smash{\scriptscriptstyle\smile}$}}{F} (s_{i} ;{\mathbf{s}}^{{\prime }} ;{\mathbf{m}}^{{\prime }} ) $$ differs from $$ F(s_{i} ;{\mathbf{s}}^{{\prime }} ;{\mathbf{n}}^{{\prime }} ) $$ as a mathematical object, their biological meaning is the same. Lemma 1 thus ensures the expandability of $$ F(s_{i} ;{\mathbf{s}}^{{\prime }} ;{\mathbf{n}}^{{\prime }} ) $$ in terms of $$ {\mathbf{m}}^{{\prime }} $$. The estimate $$ C_{\text{Fm}}^{{\prime }} $$ still depends on $${\mathbf{s}}^{{\prime }}$$, but is positive and uniformly bounded away from 0 and ∞.

As we did for $C_{\text{Fm}}^{{\prime }}$, below we will introduce bounds for other important variables and functions in the form of expressions $C_{ \cdot }$ that are independent of population densities (but may be functions of other model parameters). Please notice that here we have introduced the notational convention, to which we adhere throughout this paper, that $C_F{ \cdot }$ denotes the upper bound for the absolute value (or norm) of $\cdot$ the derivative of the fitness function with respect to ${ \cdot },$ while $C_{ \cdot }$ denotes the upper bound for the absolute value (or norm) of ${ \cdot }$ or for the derivative of the first symbol in ${ \cdot }$ with respect to the subsequent symbol(s). All $C_F{ \cdot }$ and $C_{ \cdot }$are positive and uniformly bounded away from 0 and ∞. In the propositions below, we just indicate that such constants exist. Expressions for determining their values are derived in the associated appendices and are shown in Table 2.

**Table 2:** List of constants describing the upper bounds for variables dependent on population densities and (or) phenotypes. Notation: $C_{{{\text{F}} \cdot }}$ denotes the upper bound for the absolute value (or norm) of the derivative of the fitness function with respect to $\cdot$, while $C_{ \cdot }$ denotes the upper bound for the absolute value (or norm) of $\cdot$ or for the derivative of the first symbol in $\cdot$ with respect to the subsequent symbol(s)

Constant and formula	Location
*Constants used in formulas below*^*a*^	
$C_{\text{Fn}}^{{\prime }} = \hbox{max} \left\{ {\left. { \, \left| {\frac{{\partial F(s_{i} ;{\mathbf{s}}^{{\prime }} ;{\mathbf{n}}^{{\prime }} )}}{{\partial n_{j} }}} \right|\;} \right|\;\;i,j = 1, \ldots ,N + 1,\;{\mathbf{n}}^{{\prime }} \in [0,\eta ]^{N + 1} } \right\}$	Eq. (A.2)^b^
$$ C_{\text{Fsn}}^{{\prime }} = \hbox{max} \left\{ { \, \left| {\frac{{\partial^{2} F(s_{i} ;{\mathbf{s}}^{{\prime }} ;{\mathbf{n}}^{{\prime }} )}}{{\partial s_{j} \partial n_{j} }} - \frac{{\partial^{2} F(s_{i} ;{\mathbf{s}}^{{\prime }} ;{\mathbf{n}}^{{\prime }} )}}{{\partial s_{j} \partial n_{{{\text{cid}}(j)}} }}} \right|_{{s_{j} = s_{{j{\text{T}}}} }} \left| {\begin{array}{*{20}l} {i = 1, \ldots ,N + 1,} \hfill \\ {j = M + 1, \ldots ,N + 1,} \hfill \\ {{\mathbf{n}}^{{\prime }} \in [0,\eta ]^{N + 1} ,} \hfill \\ {s_{{j{\text{T}}}} \in [s_{j} ,s_{{{\text{cid}}(j)}} ]} \hfill \\ \end{array} } \right.} \right\} $$	Eq. (A.12)
$$ \overset{\lower0.5em\hbox{$\smash{\scriptscriptstyle\frown}$}}{C_{\text{Fz}}^{{\prime }}} = \hbox{max} \left\{ {\left. {\left| {\frac{{\partial F(z_{j} ;{\mathbf{s}}^{{\prime }} ;{{\overset{\lower0.5em\hbox{$\smash{\scriptscriptstyle\frown}$}}{{\mathbf{n}}^{{\prime }}} }} )}}{{\partial z_{j} }}} \right|_{{z_{j} = s_{{j{\text{T}}}} }} } \right|\,j = M + 1, \ldots ,N + 1,\,\,s_{{j{\text{T}}}} \in [s_{j} ,s_{{{\text{cid}}(j)}} ]} \right\} $$	Eq. (B.12)
$C_{\text{Fnn}}^{{\prime }} = \hbox{max} \left\{ {\left. {\left| {\frac{{\partial F(s_{i} ;{\mathbf{s}}^{{\prime }} ;{\mathbf{n}}^{{\prime }} )}}{{\partial n_{k} \partial n_{j} }}} \right| \, } \right|\;\;i,j,k = 1, \ldots ,N + 1,\;{\mathbf{n}}^{{\prime }} \in [0,\eta ]^{N + 1} } \right\}$	Eq. (B.17)
$$ C_{\text{Fsnn}}^{{\prime }} = \hbox{max} \left\{ {\left| {\frac{{\partial^{3} F(s_{i} ;{\mathbf{s}}^{{\prime }} ;{\mathbf{n}}^{{\prime }} )}}{{\partial s_{j} \partial n_{k} \partial n_{j} }} - \frac{{\partial^{3} F(s_{i} ;{\mathbf{s}}^{{\prime }} ;{\mathbf{n}}^{{\prime }} )}}{{\partial s_{j} \partial n_{k} \partial n_{{{\text{cid}}(j)}} }}} \right|_{{s_{j} = s_{{j{\text{T}}}} }} \;\left| {\begin{array}{*{20}l} {i = 1, \ldots ,N + 1,} \hfill \\ {j = M + 1, \ldots ,N + 1,} \hfill \\ {k = 1, \ldots ,M,} \hfill \\ {{\mathbf{n}}^{{\prime }} \in [0,\eta ]^{N + 1} ,} \hfill \\ {s_{{j{\text{T}}}} \in [s_{j} ,s_{{{\text{cid}}(j)}} ]} \hfill \\ \end{array} } \right.} \right\} $$	Eq. (B.26)
$$ C_{\text{Fssnn}}^{{\prime }} = \hbox{max} \left\{ {\left. {\begin{array}{*{20}l} {\frac{{\partial^{4} F(s_{i} ;{\mathbf{s}}^{{\prime }} ;{\mathbf{n}}^{{\prime }} )}}{{\partial s_{k} \partial s_{j} \partial n_{k} \partial n_{j} }} - \frac{{\partial^{4} F(s_{i} ;{\mathbf{s}}^{{\prime }} ;{\mathbf{n}}^{{\prime }} )}}{{\partial s_{k} \partial s_{j} \partial n_{k} \partial n_{{{\text{cid}}(j)}} }}} \hfill \\ {\quad - \,\frac{{\partial^{4} F(s_{i} ;{\mathbf{s}}^{{\prime }} ;{\mathbf{n}}^{{\prime }} )}}{{\partial s_{k} \partial s_{j} \partial n_{{{\text{cid}}(k)}} \partial n_{j} }} + \frac{{\partial^{4} F(s_{i} ;{\mathbf{s}}^{{\prime }} ;{\mathbf{n}}^{{\prime }} )}}{{\partial s_{k} \partial s_{j} \partial n_{{{\text{cid}}(k)}} \partial n_{{{\text{cid}}(j)}} }}} \hfill \\ \end{array} } \right|_{{s_{k} = s_{{k{\text{T}}}} ,s_{j} = s_{{j{\text{T}}}} }} \left| {\begin{array}{*{20}l} {i,j,k = 1, \ldots ,N + 1,\,} \hfill \\ {{\mathbf{n}}^{{\prime }} \in [0,\eta ]^{N + 1} ,} \hfill \\ {s_{{j{\text{T}}}} \in [s_{j} ,s_{{{\text{cid}}(j)}} ],} \hfill \\ {s_{{k{\text{T}}}} \in [s_{k} ,s_{{{\text{cid}}(k)}} ]} \hfill \\ \end{array} } \right.} \right\} $$	Eq. (B.28)
$$ C_{{{\text{F}}\upvarepsilon}}^{{\prime }} = \hbox{max} \left\{ {\left| {\frac{{\partial F(s_{{{\text{cid}}(j)}} + \varepsilon \rho_{j} ;{\mathbf{s}}_{\text{a}}^{{\prime }} + \varepsilon {\varvec{\uprho}}^{{\prime }} ;{\mathbf{n}}^{{\prime }} )}}{\partial \varepsilon }} \right|_{{\varepsilon = \varepsilon_{{{\text{T}}j}} }} \;\left| {\begin{array}{*{20}l} {j = 1, \ldots ,N + 1,} \hfill \\ {{\mathbf{n}}^{{\prime }} \in [0,\eta ]^{N + 1} ,} \hfill \\ {\varepsilon_{{{\text{T}}j}} \in [0,\varepsilon ]} \hfill \\ \end{array} } \right.} \right\} $$	Eq. (C.3)
$C_{{{\text{F}}{\mathbf{mm}}}} = \hbox{max} \left\{ {\left. {\left\| {\frac{{\partial^{2} F(s_{i} ;{\mathbf{s}}_{\text{a}} ;{\mathbf{m}})}}{{\partial {\mathbf{m}}\partial {\mathbf{m}}^{\text{T}} }}} \right\|_{\text{Q}} } \right|\;i = 1, \ldots ,M,\;{\mathbf{m}} \in [0,\eta ]^{M} } \right\}$	Eq. (C.12)
$$ C_{{{\text{F}}\varepsilon }} = \hbox{max} \left\{ {\left| {\frac{{\partial F(s_{{{\text{cid}}(j)}} + \varepsilon \rho_{j} ;{\mathbf{s}}_{\text{a}}^{{\prime }} + \varepsilon {\varvec{\uprho}}^{{\prime }} ;{\mathbf{n}}^{{\prime }} )}}{\partial \varepsilon }} \right|_{{\varepsilon = \varepsilon_{{{\text{T}}j}} }} \;\left| {\begin{array}{*{20}l} {j = 1, \ldots ,M,} \hfill \\ {{\mathbf{n}}^{{\prime }} \in [0,\eta ]^{N + 1} ,} \hfill \\ {\varepsilon_{{{\text{T}}j}} \in [0,\varepsilon ]} \hfill \\ \end{array} } \right.} \right\} $$	Eq. (J.21)
$$ \begin{aligned} & C_{{{\mathbf{F}}\varepsilon {\mathbf{m}}}} = \hbox{max} \left\{ {\left\| {\frac{{\partial {\mathbf{F}}_{\varepsilon }^{{\prime }} }}{{\partial {\mathbf{m}}}}} \right\|\;\left| {\begin{array}{*{20}l} {\varepsilon_{{{\text{T}}i}} \in [0,\varepsilon ],} \hfill \\ {m_{1} , \ldots ,m_{M} \in [0,\eta ],} \hfill \\ {m_{M + 1} , \ldots ,m_{N + 1} \in [0,\varepsilon \eta ]} \hfill \\ \end{array} } \right.} \right\} \\ & {\text{ with}}\;{\mathbf{F}}_{\varepsilon }^{{\prime }} = \left( {\begin{array}{*{20}c} {F_{\varepsilon 1} } \\ \vdots \\ {F_{\varepsilon ,N + 1} } \\ \end{array} } \right) = \left( {\begin{array}{*{20}c} {\frac{{\partial \overset{\lower0.5em\hbox{$\smash{\scriptscriptstyle\smile}$}}{F} (s_{{{\text{cid}}(1)}} + \varepsilon \rho_{1} ;{\mathbf{s}}_{\text{a}}^{{\prime }} + \varepsilon {\varvec{\uprho}}^{{\prime }} ;{\mathbf{m}}^{{\prime }} )}}{\partial \varepsilon }} \\ \vdots \\ {\frac{{\partial \overset{\lower0.5em\hbox{$\smash{\scriptscriptstyle\smile}$}}{F} (s_{{{\text{cid}}(N + 1)}} + \varepsilon \rho_{N + 1} ;{\mathbf{s}}_{\text{a}}^{{\prime }} + \varepsilon {\varvec{\uprho}}^{{\prime }} ;{\mathbf{m}}^{{\prime }} )}}{\partial \varepsilon }} \\ \end{array} } \right) \\ \end{aligned} $$	Eq. (J.22)
$$ C_{\text{Fzz}}^{{\prime }} = \hbox{max} \left\{ {\left| {\frac{{\partial^{2} F(z;{\mathbf{s}}^{{\prime }} ;{\mathbf{n}}^{{\prime }} )}}{{\partial z^{2} }}} \right|_{{z = s_{{j{\text{T}}}} }} \left| {\begin{array}{*{20}l} {\left| {\Delta {\mathbf{m}}^{{\prime }} } \right| \in [0,\varepsilon C_{{\mathbf{m}}}^{{\prime }} ],} \hfill \\ {j = M + 1, \ldots ,N + 1,} \hfill \\ {s_{{j{\text{T}}}} \in [s_{j} ,s_{{{\text{cid(}}j)}} ]} \hfill \\ \end{array} } \right. \, } \right\} $$	Eq. (K.6)
$$ C_{\text{Fzn}}^{{\prime }} = \hbox{max} \left\{ {\left| {\frac{{\partial^{2} F(z;{\mathbf{s}}^{{\prime }} ;{\mathbf{n}}^{{\prime }} )}}{{\partial z\partial n_{j} }}} \right|_{{z = s_{{j{\text{T}}}} }} \left| {\begin{array}{*{20}l} {j = 1, \ldots ,N + 1, \, } \hfill \\ {{\mathbf{n}}^{{\prime }} \in [0,\eta ]^{N + 1} ,} \hfill \\ {s_{{j{\text{T}}}} \in [s_{{{\text{cid}}(j)}} ,s_{j} ]} \hfill \\ \end{array} } \right.} \right\} $$	Eq. (K.11)
$$ C_{\text{Fzsn}}^{{\prime }} = \hbox{max} \left\{ {\left| {\frac{{\partial^{3} F(z;{\mathbf{s}}^{{\prime }} ;{\mathbf{n}}^{{\prime }} )}}{{\partial z\partial s_{j} \partial n_{j} }} - \frac{{\partial^{3} F(z;{\mathbf{s}}^{{\prime }} ;{\mathbf{n}}^{{\prime }} )}}{{\partial z\partial s_{j} \partial n_{{{\text{cid}}(j)}} }}} \right|_{{z = s_{{j{\text{T}}}} ,s_{j} = s_{{j{\text{TT}}}} }} \left| {\begin{array}{*{20}l} {j = 1, \ldots ,N + 1, \, } \hfill \\ {{\mathbf{n}}^{{\prime }} \in [0,\eta ]^{N + 1} ,} \hfill \\ {s_{{j{\text{T}}}} \in [s_{{{\text{cid}}(j)}} ,s_{j} ],} \hfill \\ {s_{{j{\text{TT}}}} \in [s_{{{\text{cid}}(j)}} ,s_{j} ]} \hfill \\ \end{array} } \right.} \right\} $$	Eq. (K.13)

Constant and formula	Location and proof
*Constants defined based on definitions above*	
$C_{\text{Fm}}^{{\prime }} = \hbox{max} \left\{ {C_{\text{Fn}}^{{\prime }} ,C_{\text{Fsn}}^{{\prime }} } \right\}$	Eq. (3.2) Appendix A
$C_{\text{Fmm}}^{{\prime }} = \hbox{max} \left\{ {C_{\text{Fnn}}^{{\prime }} ,C_{\text{Fsnn}}^{{\prime }} ,C_{\text{Fssnn}}^{{\prime }} } \right\}$	Eq. (B.29) Appendix B.4
$C_{{{\text{F}}{\mathbf{mm}}}}^{{\prime }} = (N + 1)C_{\text{Fmm}}^{{\prime }}$	Eq. (B.31) Appendix B.4
$C_{{\mathbf{m}}} = \frac{{2\left\| {{\mathbf{P}}^{ - 1} } \right\|C_{{\mathbf{h}}} }}{{\left| {\lambda_{\rm max} } \right|}}$ (in Theorem 2)	Eqs. (3.4a), (4.9a) Lemma 6
$$ \begin{aligned} \\ C_{{\mathbf{m}}} & = \frac{{2\alpha \left\| {{\mathbf{Q}}^{ - 1} } \right\|\hbox{max} \left\{ {\tilde{C}_{{\mathbf{h}}} ,\, - \tfrac{1}{2}\,\tilde{\lambda }_{\rm {max} } \rho_{\text{m}} } \right\}}}{{\left| {\tilde{\lambda }_{\rm {max} } } \right|}} + \rho_{\text{m}} \\ \end{aligned} $$ (in Theorem 3)	Eqs. (3.4a), (5.9a) Lemma 11
$C_{{\mathbf{m}}}^{{\prime }} = \sqrt {C_{{\mathbf{m}}}^{2} + (N + 1 - M)\eta^{2} }$	Eq. (3.4b) Appendix B.3
$C_{{{\mathbf{rm}}}} = \left\| {\mathbf{B}} \right\| + \eta \sqrt M C_{{{\text{F}}{\mathbf{mm}}}}$	Lemma 3 Appendix C.2
$$ C_{\text{hm}} = \eta \sqrt M \left[ {\overset{\lower0.5em\hbox{$\smash{\scriptscriptstyle\frown}$}}{C_{\text{Fz}}^{{\prime }}} + 2C_{{{\text{F}}\upvarepsilon}}^{{\prime }} } \right] $$	Lemma 3 Appendix C.2
$C_{{\mathbf{r}}} = \left\| {\mathbf{P}} \right\|\left\| {{\mathbf{P}}^{ - 1} } \right\|^{2} C_{{{\mathbf{rm}}}} = \left\| {\mathbf{P}} \right\|\left\| {{\mathbf{P}}^{ - 1} } \right\|^{2} \left[ {\left\| {\mathbf{B}} \right\| + \eta \sqrt M C_{{{\text{F}}{\mathbf{mm}}}} } \right]$	Eq. (4.4) Appendix D
$$ C_{{\mathbf{h}}} = \left\| {\mathbf{P}} \right\|C_{{{\mathbf{hm}}}} = \left\| {\mathbf{P}} \right\|\eta \sqrt M \left[ {\overset{\lower0.5em\hbox{$\smash{\scriptscriptstyle\frown}$}}{C_{\text{Fz}}^{{\prime }} }+ 2C_{{{\text{F}}\upvarepsilon}}^{{\prime }} } \right] $$	Eq. (4.4) Appendix D
$C_{{{\mathbf{r}}{\text{f}}}} = \sqrt M C_{{{\text{F}}{\mathbf{mm}}}}$	Eq. (5.1) Appendix C.1
$$ C_{{{\mathbf{h}}{\text{f}}}} = \sqrt M \left[ {\overset{\lower0.5em\hbox{$\smash{\scriptscriptstyle\frown}$}}{C_{\text{Fz}}^{{\prime }} }+ 2C_{{{\text{F}}\upvarepsilon}}^{{\prime }} } \right] $$	Eq. (5.1) Appendix C.1
$\tilde{C}_{{{\mathbf{r}}{\text{f}}}} = C_{{{\mathbf{r}}{\text{f}}}}$	Eq. (F.6) AppendixF.1
$\tilde{C}_{{{\mathbf{h}}{\text{f}}}} = C_{{{\mathbf{h}}{\text{f}}}} + \left\| {\mathbf{B}} \right\| \rho_{\text{m}} + C_{{{\mathbf{r}}{\text{f}}}} \left[ {2\eta + \varepsilon \rho_{\text{m}} } \right]\rho_{\text{m}}$	Eq. (F.6) Appendix F.1
$\tilde{C}_{{{\mathbf{rx}}}} = \left\| {\mathbf{P}} \right\|\left( {1 + \left\| {\mathbf{T}} \right\|} \right)\left\| {{\mathbf{Q}}^{ - 1} } \right\|^{2} \left[ {\left\| {\mathbf{B}} \right\| + \eta \tilde{C}_{{{\mathbf{r}}{\text{f}}}} } \right]$	Eq. (5.4a) Appendix F.4
$\tilde{C}_{{{\mathbf{ry}}}} = \tilde{C}_{{{\mathbf{r}}{\text{f}}}} \left\| {{\mathbf{Q}}^{ - 1} } \right\|^{2}$	Eq. (5.4a) Appendix F.4
$\tilde{C}_{{{\mathbf{hx}}}} = \left\| {\mathbf{P}} \right\|\left( {1 + \left\| {\mathbf{T}} \right\|} \right)\eta \tilde{C}_{{{\mathbf{h}}{\text{f}}}}$	Eq. (5.4a) Appendix F.4
$\tilde{C}_{{{\mathbf{hy}}}} = \tilde{C}_{\text{hf}}$	Eq. (5.4a) Appendix F.4
$\tilde{C}_{{\mathbf{r}}} = \sqrt {\tilde{C}_{{{\mathbf{rx}}}}^{2} + d^{2} \tilde{C}_{{{\mathbf{ry}}}}^{2} }$	Eq. (5.6c) Appendix F.5
$\tilde{C}_{{\mathbf{h}}} = \sqrt {\tilde{C}_{{{\mathbf{hx}}}}^{2} + d^{2} \tilde{C}_{{{\mathbf{hy}}}}^{2} }$	Eq. (5.6c) Appendix F.5
$$ \overset{\lower0.5em\hbox{$\smash{\scriptscriptstyle\frown}$}}{C}_{{\mathbf{h}}} = \left\| {{\mathbf{P}}{\text{diag}}({\overset{\lower0.5em\hbox{$\smash{\scriptscriptstyle\frown}$}}{{\mathbf{m}}} }){\mathbf{h}}_{\text{f}} } \right\|_{{{\mathbf{m}} = {{\overset{\lower0.5em\hbox{$\smash{\scriptscriptstyle\frown}$}}{{\mathbf{m}}} }}}} $$	Eq. (6.2) Appendix J.2
$C_{{\mathbf{H}}} = \left\| {\mathbf{P}} \right\|\left\| {{\mathbf{P}}^{ - 1} } \right\|\left[ {2C_{{{\text{F}}\varepsilon }} + (N + 1)\eta C_{{{\mathbf{F}}\varepsilon {\mathbf{m}}}} } \right]$	Eq. (6.2) Appendix J.2
$C_{{{\text{Fz}}{\mathbf{m}}}}^{{\prime }} = \sqrt {MC_{\text{Fzn}}^{{{\prime }2}} + (N + 1 - M)C_{\text{Fzsn}}^{{{\prime }2}} }$	Eq. (8.2b) Appendix K

^a^Tighter estimates that apply under various restrictions are presented in the appendices

^b^Here $$ \left\| {\mathbf{B}} \right\|_{\text{Q}} : = \hbox{max} \left\{ {\left. {\left| {{\mathbf{v}}^{\text{T}} {\mathbf{Bv}}} \right|\,} \right|\,\left| {\mathbf{v}} \right| = 1} \right\} $$

### Taylor expansion in the population densities of the approximate phenotypes

We now expand the fitness-generating function in $$ {\mathbf{m}}^{{\prime }} $$. We denote by $$ {{\overset{\lower0.5em\hbox{$\smash{\scriptscriptstyle\frown}$}}{{\mathbf{m}}^{{\prime }}} }} = (\overset{\lower0.5em\hbox{$\smash{\scriptscriptstyle\frown}$}}{m}_{1} , \ldots ,\overset{\lower0.5em\hbox{$\smash{\scriptscriptstyle\frown}$}}{m}_{N + 1} )^{\text{T}} $$ the initial state $$ {{\overset{\lower0.5em\hbox{$\smash{\scriptscriptstyle\frown}$}}{{\mathbf{n}}^{{\prime }}} }} = (\overset{\lower0.5em\hbox{$\smash{\scriptscriptstyle\frown}$}}{n}_{1} , \ldots ,\overset{\lower0.5em\hbox{$\smash{\scriptscriptstyle\frown}$}}{n}_{N + 1} ) $$ expressed in terms of $$ {\mathbf{m}}^{{\prime }} $$, with $$ \overset{\lower0.5em\hbox{$\smash{\scriptscriptstyle\frown}$}}{m}_{i} = \sum\nolimits_{{j \in {\text{com}}(i)}} {\overset{\lower0.5em\hbox{$\smash{\scriptscriptstyle\frown}$}}{n}_{j} } $$ for $$ i = 1, \ldots ,M $$ and $$ \overset{\lower0.5em\hbox{$\smash{\scriptscriptstyle\frown}$}}{m}_{i} = \varepsilon \overset{\lower0.5em\hbox{$\smash{\scriptscriptstyle\frown}$}}{n}_{i} $$ for $$ i = M + 1, \ldots ,N + 1 $$. Lemma 1 allows $$ F(s_{i} ;{\mathbf{s}}^{{\prime }} ;{\mathbf{n}}^{{\prime }} ) $$ to be expanded in $$ {\mathbf{m}}^{{\prime }} $$ around $$ {{\overset{\lower0.5em\hbox{$\smash{\scriptscriptstyle\frown}$}}{{\mathbf{n}}}^{{\prime }}} } $$ as

3.3a $$ F(s_{i} ;{\mathbf{s}}^{{\prime }} ;{\mathbf{n}}^{{\prime }} ) = \overset{\lower0.5em\hbox{$\smash{\scriptscriptstyle\frown}$}}{F}_{i} + {\mathbf{b}}_{i}^{{{\prime }{\text{T}}}} ({\mathbf{m}}^{{\prime }} - {{\overset{\lower0.5em\hbox{$\smash{\scriptscriptstyle\frown}$}}{{\mathbf{m}}^{{\prime }} } }}) + R_{i} , $$

where

3.3b $$ \overset{\lower0.5em\hbox{$\smash{\scriptscriptstyle\frown}$}}{F}_{i} : = F(s_{i} ;{\mathbf{s}}^{{\prime }} ;{{\overset{\lower0.5em\hbox{$\smash{\scriptscriptstyle\frown}$}}{{\mathbf{n}}^{{\prime }}} }} ), $$

3.3c $$ {\mathbf{b}}_{i}^{{{\prime }{\text{T}}}} = \left( {b_{i1}^{{\prime }} , \ldots ,b_{i\,N + 1}^{{\prime }} } \right): = \left. {\frac{{\partial \overset{\lower0.5em\hbox{$\smash{\scriptscriptstyle\smile}$}}{F} (s_{i} ;{\mathbf{s}}^{{\prime }} ;{\mathbf{m}}^{{\prime }} )}}{{\partial {\mathbf{m}}^{{\prime }} }}} \right|_{{{\mathbf{m}}^{{\prime }} = {{\overset{\lower0.5em\hbox{$\smash{\scriptscriptstyle\frown}$}}{{\mathbf{m}}^{{\prime }}} }} }} = \left( {\frac{{\partial \overset{\lower0.5em\hbox{$\smash{\scriptscriptstyle\smile}$}}{F} (s_{i} ;{\mathbf{s}}^{{\prime }} ;{\mathbf{m}}^{{\prime }} )}}{{\partial m_{1}^{{\prime }} }}, \ldots ,\frac{{\partial \overset{\lower0.5em\hbox{$\smash{\scriptscriptstyle\smile}$}}{F} (s_{i} ;{\mathbf{s}}^{{\prime }} ;{\mathbf{m}}^{{\prime }} )}}{{\partial m_{N + 1}^{{\prime }} }}} \right)_{{{\mathbf{m}}^{{\prime }} = {{\overset{\lower0.5em\hbox{$\smash{\scriptscriptstyle\frown}$}}{{\mathbf{m}}^{{\prime }}} }} }} , $$

and

3.3d $$ R_{i} : = \frac{1}{2}({\mathbf{m}}^{{\prime }} - {{\overset{\lower0.5em\hbox{$\smash{\scriptscriptstyle\frown}$}}{\mathbf{m}^{{\prime }}} }} )^{\text{T}} \left. {\frac{{\partial^{2} \overset{\lower0.5em\hbox{$\smash{\scriptscriptstyle\smile}$}}{F} (s_{i} ;{\mathbf{s}}^{{\prime }} ;{\mathbf{m}}^{{\prime }} )}}{{\partial {\mathbf{m}}^{{\prime }} \partial {\mathbf{m}}^{{{\prime }{\text{T}}}} }}} \right|_{{{\mathbf{m}}^{{\prime }} = {{\overset{\lower0.5em\hbox{$\smash{\scriptscriptstyle\frown}$}}{{\mathbf{m}}^{{\prime }} } }}}} ({\mathbf{m}}^{{\prime }} - {{\overset{\lower0.5em\hbox{$\smash{\scriptscriptstyle\frown}$}}{{\mathbf{m}}^{{\prime }}} }} ) + {\text{h}} . {\text{o}} . {\text{t}} . , $$

with

3.3e $$ \left. {\frac{{\partial^{2} \overset{\lower0.5em\hbox{$\smash{\scriptscriptstyle\smile}$}}{F} (s_{i} ;{\mathbf{s}}^{{\prime }} ;{\mathbf{m}}^{{\prime }} )}}{{\partial {\mathbf{m}}^{{\prime }} \partial {\mathbf{m}}^{{{\prime }{\text{T}}}} }}} \right|_{{{\mathbf{m}}^{{\prime }} = {{\overset{\lower0.5em\hbox{$\smash{\scriptscriptstyle\frown}$}}{{\mathbf{m}}^{{\prime }} } }}}} = \left( {\begin{array}{*{20}c} {\frac{{\partial^{2} \overset{\lower0.5em\hbox{$\smash{\scriptscriptstyle\smile}$}}{F} (s_{i} ;{\mathbf{s}}^{{\prime }} ;{\mathbf{m}}^{{\prime }} )}}{{\partial m_{1}^{{{\prime }2}} }}} & \cdots & {\frac{{\partial^{2} \overset{\lower0.5em\hbox{$\smash{\scriptscriptstyle\smile}$}}{F} (s_{i} ;{\mathbf{s}}^{{\prime }} ;{\mathbf{m}}^{{\prime }} )}}{{\partial m_{1}^{{\prime }} \partial m_{N + 1}^{{\prime }} }}} \\ \vdots & \ddots & \vdots \\ {\frac{{\partial^{2} \overset{\lower0.5em\hbox{$\smash{\scriptscriptstyle\smile}$}}{F} (s_{i} ;{\mathbf{s}}^{{\prime }} ;{\mathbf{m}}^{{\prime }} )}}{{\partial m_{1}^{{\prime }} \partial m_{N + 1}^{{\prime }} }}} & \cdots & {\frac{{\partial^{2} \overset{\lower0.5em\hbox{$\smash{\scriptscriptstyle\smile}$}}{F} (s_{i} ;{\mathbf{s}}^{{\prime }} ;{\mathbf{m}}^{{\prime }} )}}{{\partial m_{N + 1}^{{{\prime }2}} }}} \\ \end{array} } \right)_{{{\mathbf{m}}^{{\prime }} = {{\overset{\lower0.5em\hbox{$\smash{\scriptscriptstyle\frown}$}}{{\mathbf{m}}^{{\prime }}} }} }}. $$

Here $$ \overset{\lower0.5em\hbox{$\smash{\scriptscriptstyle\frown}$}}{F}_{i} = 0 $$ for $$ i = 1, \ldots ,N $$ (from the equilibrium equation of the residents), and

3.3f $$ \left| {\overset{\lower0.5em\hbox{$\smash{\scriptscriptstyle\frown}$}}{F}_{N + 1} } \right| \le \varepsilon \overset{\lower0.5em\hbox{$\smash{\scriptscriptstyle\frown}$}}{C_{\text{Fz}}^{{\prime }}} $$

(Appendix B.2). Moreover, by the bounded-world axiom (v) and Taylor’s theorem, we have

#### **Lemma 2**

*If there exists a constant* $C_{{\mathbf{m}}}$*such that*

3.4a $$ \left| {{\mathbf{m}} - {{\overset{\lower0.5em\hbox{$\smash{\scriptscriptstyle\frown}$}}{{\mathbf{m}}} }}} \right| \le \varepsilon C_{{\mathbf{m}}} , $$

*then there exist constants* $C_{{\mathbf{m}}}^{{\prime }} = \sqrt {C_{{\mathbf{m}}}^{2} + (N - M + 1)\eta^{2} }$*and* $C_{{{\text{F}}{\mathbf{mm}}}}^{{\prime }}$*satisfying*

3.4b $$ \left| {{\mathbf{m}}^{{\prime }} - {{\overset{\lower0.5em\hbox{$\smash{\scriptscriptstyle\frown}$}}{{\mathbf{m}}^{{\prime }}} }} } \right| \le \varepsilon C_{{\mathbf{m}}}^{{\prime }} $$

*and*

3.4c $$ \left| {R_{i} } \right| \le \tfrac{1}{2}C_{{{\text{F}}{\mathbf{mm}}}}^{{\prime }} \left| {{\mathbf{m}}^{{\prime }} - {{\overset{\lower0.5em\hbox{$\smash{\scriptscriptstyle\frown}$}}{{\mathbf{m}}^{{\prime }}} }} } \right|^{2} \le \tfrac{1}{2}\varepsilon^{2} C_{{{\text{F}}{\mathbf{mm}}}}^{{\prime }} C_{{\mathbf{m}}}^{{{\prime }2}} , $$

*where for vectors* $\left| {\, \cdot \,} \right|$*denotes the Euclidian norm.*

See Appendix B.3 for the proof. Thus, if Eq. (3.4a) is satisfied for a sufficiently small $\varepsilon$, the fitness-generating function is approximated well by a linear function of ${\mathbf{m}}^{{\prime }}$.

### Taylor expansion in the population densities of the original phenotypes

Next, we transform the term linear in ${\mathbf{m}}^{{\prime }}$ in Eq. (3.3a) into one in ${\mathbf{n}}^{{\prime }}$. As ${\mathbf{m}}^{{\prime }}$ is a linear function of ${\mathbf{n}}^{{\prime }}$, ${\mathbf{m}}^{{\prime }}$ can be written as ${\mathbf{m}}^{{\prime }} = {\mathbf{Wn}}^{{\prime }}$, where ${\mathbf{W}}$ is a $(N + 1)$-by-$(N + 1)$ matrix with components given by Eq. (3.1a). Therefore, substituting this relationship into Eq. (3.3a) gives

3.5a $$ \begin{aligned} F(s_{i} ;{\mathbf{s}}^{{\prime }} ;{\mathbf{n}}^{{\prime }} ) & = \overset{\lower0.5em\hbox{$\smash{\scriptscriptstyle\frown}$}}{F}_{i} + {\mathbf{b}}_{i}^{{{\prime }{\text{T}}}} {\mathbf{W}}({\mathbf{n}}^{{\prime }} - {{\overset{\lower0.5em\hbox{$\smash{\scriptscriptstyle\frown}$}}{{\mathbf{n}}^{{\prime }}} }} ) + R_{i} \\ & = \overset{\lower0.5em\hbox{$\smash{\scriptscriptstyle\frown}$}}{F}_{i} + {\mathbf{a}}_{i}^{\text{T}} ({\mathbf{n}}^{{\prime }} - {{\overset{\lower0.5em\hbox{$\smash{\scriptscriptstyle\frown}$}}{{\mathbf{n}}^{{\prime }}} }} ) + R_{i} , \\ \end{aligned} $$

where, since $$ \overset{\lower0.5em\hbox{$\smash{\scriptscriptstyle\smile}$}}{F} (s_{i} ;{\mathbf{s}}^{{\prime }} ;{\mathbf{m}}^{{\prime }} ) = F(s_{i} ;{\mathbf{s}}^{{\prime }} ;{\mathbf{n}}^{{\prime }} ) $$,

3.5b $$ \begin{aligned} {\mathbf{a}}_{i}^{\text{T}} & = \left( {a_{i,1} , \ldots ,a_{i\,,N + 1} } \right) := {\mathbf{b}}_{i}^{{{\prime }{\text{T}}}} {\mathbf{W}} \\ & = \left. {\frac{{\partial \overset{\lower0.5em\hbox{$\smash{\scriptscriptstyle\smile}$}}{F} (s_{i} ;{\mathbf{s}}^{{\prime }} ;{\mathbf{m}}^{{\prime }} )}}{{\partial {\mathbf{m}}^{{\prime }} }}} \right|_{{{\mathbf{m}}^{{\prime }} = {{\overset{\lower0.5em\hbox{$\smash{\scriptscriptstyle\frown}$}}{{\mathbf{m}}^{{\prime }}} }} }} {\mathbf{W}} \\ & = \left. {\frac{{\partial F(s_{i} ;{\mathbf{s}}^{{\prime }} ;{\mathbf{n}}^{{\prime }} )}}{{\partial {\mathbf{n}}^{{\prime }} }}} \right|_{{{\mathbf{n}}^{{\prime }} = {{\overset{\lower0.5em\hbox{$\smash{\scriptscriptstyle\frown}$}}{{\mathbf{n}}^{{\prime }}} }} }} \\ & = \left. {\left( {\frac{{\partial F(s_{i} ;{\mathbf{s}}^{{\prime }} ;{\mathbf{n}}^{{\prime }} )}}{{\partial n_{1}^{{\prime }} }}, \ldots ,\frac{{\partial F(s_{i} ;{\mathbf{s}}^{{\prime }} ;{\mathbf{n}}^{{\prime }} )}}{{\partial n_{N + 1}^{{\prime }} }}} \right)} \right|_{{{\mathbf{n}}^{{\prime }} = {{\overset{\lower0.5em\hbox{$\smash{\scriptscriptstyle\frown}$}}{{\mathbf{n}}^{{\prime }}} }} }} . \\ \end{aligned} $$

By combining the equations above with Lemma 2, and by using Eq. (2.3), we get

#### **Theorem 1**

*For the population densities*${\mathbf{m}} = (m_{1} , \ldots ,m_{M} )^{\text{T}}$*of approximate phenotypes*${\mathbf{s}}_{\text{a}} = (s_{1} , \ldots ,s_{M} )^{\text{T}}$*formed by clustering resident and mutant phenotypes*${\mathbf{s}}^{{\prime }} = (s_{1} , \ldots ,s_{N + 1} )^{\text{T}}$*according to a threshold phenotypic distance*$\varepsilon = \varepsilon_{\mu } \rho_{\mu }$*, if*${\mathbf{m}}$*satisfies*

$$ \left| {{\mathbf{m}} - {{\overset{\lower0.5em\hbox{$\smash{\scriptscriptstyle\frown}$}}{{\mathbf{m}}} }}} \right| \le \varepsilon C_{{\mathbf{m}}} , $$

*during the transient following mutant invasion, then the fitness*-*generating function can be expanded as*

3.6a $$ F(s_{i} ;{\mathbf{s}}^{{\prime }} ;{\mathbf{n}}^{{\prime }} ) = \overset{\lower0.5em\hbox{$\smash{\scriptscriptstyle\frown}$}}{F}_{i} + \sum\limits_{j = 1}^{N + 1} {a_{ij} (n_{j} - \overset{\lower0.5em\hbox{$\smash{\scriptscriptstyle\frown}$}}{n}_{j} )} + R_{i} $$

*with*$$ \left| {R_{i} } \right| \le C_{{{\text{F}}{\mathbf{mm}}}}^{{\prime }} \left| {{\mathbf{m}}^{{\prime }} - {{\overset{\lower0.5em\hbox{$\smash{\scriptscriptstyle\frown}$}}{{\mathbf{m}}^{{\prime }}} }} } \right|^{2} \le \varepsilon^{2} C_{{{\text{F}}{\mathbf{mm}}}}^{{\prime }} C_{{\mathbf{m}}}^{{\prime }} $$*, which gives the LV*-*approximation*

3.6b $$ \frac{{{\text{d}}n_{i} }}{{{\text{d}}t}} = n_{i} \left[ {\gamma_{i} + \sum\limits_{j = 1}^{N + 1} {a_{ij} n_{j} } + R_{i} } \right] $$

*with*$$ \gamma_{i} = \overset{\lower0.5em\hbox{$\smash{\scriptscriptstyle\frown}$}}{F}_{i} - \sum\nolimits_{j = 1}^{N + 1} {a_{ij} \overset{\lower0.5em\hbox{$\smash{\scriptscriptstyle\frown}$}}{n}_{j} } $$.

## Approximability condition when the population densities of the approximate phenotypes are large

In this section, we consider the sufficient condition in Eq. (3.4a) for LV-approximability, $$ \left| {{\mathbf{m}} - {{\overset{\lower0.5em\hbox{$\smash{\scriptscriptstyle\frown}$}}{{\mathbf{m}}} }}} \right| < \varepsilon C_{{\mathbf{m}}} $$. We refer to this as the approximability condition. If the initial equilibrium population densities of approximate phenotypes are not small, so that $$ \overset{\lower0.5em\hbox{$\smash{\scriptscriptstyle\frown}$}}{m}_{i} \gg \varepsilon $$ is satisfied for all $$ i = 1, \ldots ,M $$, their dynamics can be analyzed by a linear stability analysis of the resident equilibrium. On the other hand, if some approximate phenotypes have very small equilibrium population densities, also the corresponding eigenvalues of the associated Jacobian come very close to zero and examining linear terms alone is not sufficient. In this section, we analyze the first, simpler, case to show that the approximability condition (3.4a) can generally be fulfilled. The second, more complicated, case is then analyzed in a similar manner in the next section.

### Dynamics of approximate phenotypes

The dynamics of approximate phenotypes ${\mathbf{m}} = (m_{1} , \ldots ,m_{M} )^{\text{T}}$ satisfies, by Eqs. (3.1a) and (2.3),

4.1a $$ \frac{{{\text{d}}m_{i} }}{{{\text{d}}t}} = m_{i} f(s_{i} ;{\mathbf{s}}^{{\prime }} ;{\mathbf{n}}^{{\prime }} ) $$

for $i = 1, \ldots ,M$, where the growth rate of $m_{i}$,

4.1b $$ f(s_{i} ;{\mathbf{s}}^{{\prime }} ;{\mathbf{n}}^{{\prime }} ): = \sum\limits_{{j \in {\text{com}}(i)}} {p_{j} F(s_{j} ;{\mathbf{s}}^{{\prime }} ;{\mathbf{n}}^{{\prime }} )} , $$

is the average growth rate within the $i$th cluster weighted with the fractions $p_{j} : = n_{j} /m_{{{\text{cid}}(j)}}$ of its component phenotypes. As for the remaining degrees of freedom in ${\mathbf{m}}^{{\prime }}$, i.e., $m_{i} = \varepsilon n_{i}$ for $i = M + 1, \ldots ,N + 1$, their dynamics are given by

4.1c $$ \frac{{{\text{d}}m_{i} }}{{{\text{d}}t}} = m_{i} F(s_{i} ;{\mathbf{s}}^{{\prime }} ;{\mathbf{n}}^{{\prime }} ). $$

When ${\mathbf{m}}$ is kept constant, these remaining degrees of freedom describe the relatively slow dynamics of the cluster compositions, corresponding to the dynamics of the fractions $p_{j} .$

### Transformation into perturbed community

For convenience, Eq. (4.1a) is rewritten in vector–matrix form as

4.2 $$ \frac{{{\text{d}}{\mathbf{m}}}}{{{\text{d}}t}} = {\text{diag}}({\mathbf{m}}){\mathbf{f}}({\mathbf{s}}_{\text{a}} ;{\mathbf{s}}^{{\prime }} ;{\mathbf{n}}^{{\prime }} ), $$

where ${\text{diag}}({\mathbf{m}})$ is a diagonal matrix with diagonal entries $m_{1} , \ldots ,m_{M}$, ${\mathbf{s}}_{\text{a}} = (s_{1} , \ldots ,s_{M} )^{\text{T}}$, and ${\mathbf{f}}({\mathbf{s}}_{\text{a}} ;{\mathbf{s}}^{{\prime }} ;{\mathbf{n}}^{{\prime }} ): = \left( {f(s_{1} ;{\mathbf{s}}^{{\prime }} ;{\mathbf{n}}^{{\prime }} ), \ldots ,f(s_{M} ;{\mathbf{s}}^{{\prime }} ;{\mathbf{n}}^{{\prime }} )} \right)^{\text{T}}$. We decompose the right-hand side of Eq. (4.2) into a component determined by ${\mathbf{m}}$ alone and a residual of order $\varepsilon$, which is treated as a perturbation. The former component is further decomposed into linear and higher-order terms. Specifically, we have

#### **Lemma 3**

*The dynamics of*${\mathbf{m}}$*in Eq*. (4.2) *can be transformed into*

4.3a $$ \frac{{{\text{d}}{\mathbf{m}}}}{{{\text{d}}t}} = {\mathbf{J}}({\mathbf{m}} - {{\overset{\lower0.5em\hbox{$\smash{\scriptscriptstyle\frown}$}}{\mathbf{m}} }}) + {\mathbf{r}}_{\text{m}} \left| {{\mathbf{m}} - {{\overset{\lower0.5em\hbox{$\smash{\scriptscriptstyle\frown}$}}{\mathbf{m}} }}} \right|^{2} + \varepsilon {\mathbf{h}}_{\text{m}} , $$

*where*

4.3b $$ \begin{aligned} {\mathbf{J}} & : = {\text{diag}}({{\overset{\lower0.5em\hbox{$\smash{\scriptscriptstyle\frown}$}}{\mathbf{m}} }}){\mathbf{B}} = \left( {\begin{array}{*{20}c} {\overset{\lower0.5em\hbox{$\smash{\scriptscriptstyle\frown}$}}{m}_{1} b_{11} } & \ldots & {\overset{\lower0.5em\hbox{$\smash{\scriptscriptstyle\frown}$}}{m}_{1} b_{1M} } \\ \ldots & \ldots & \ldots \\ {\overset{\lower0.5em\hbox{$\smash{\scriptscriptstyle\frown}$}}{m}_{M} b_{M1} } & \ldots & {\overset{\lower0.5em\hbox{$\smash{\scriptscriptstyle\frown}$}}{m}_{M} b_{MM} } \\ \end{array} } \right), \\ b_{ij} & : = \left. {\frac{{\partial F(s_{i} ;{\mathbf{s}}_{\text{a}} ;{\mathbf{m}})}}{{\partial m_{j} }}} \right|_{{{\mathbf{m}} = {{\overset{\lower0.5em\hbox{$\smash{\scriptscriptstyle\frown}$}}{\mathbf{m}} }}}} , \\ \end{aligned} $$

*and*${\mathbf{r}}_{\text{m}} = (r_{\text{m1}} , \ldots ,r_{{{\text{m}}M}} )^{\text{T}}$*is a function of*${\mathbf{m}}$*satisfying*$\left| {{\mathbf{r}}_{\text{m}} } \right| \le C_{{{\mathbf{rm}}}}$*, while*${\mathbf{h}}_{\text{m}} = (h_{\text{m1}} , \ldots ,h_{{{\text{m}}M}} )^{\text{T}}$*is a function of*${\mathbf{m}}^{{\prime }}$*and*$\varepsilon$*satisfying*$\left| {{\mathbf{h}}_{\text{m}} } \right| \le C_{{{\mathbf{hm}}}}$.

See Appendix C for the proof. Notice that ${\mathbf{J}}$ and ${\mathbf{r}}_{\text{m}}$ are both independent of $\varepsilon$.

### Local Lyapunov function

If the perturbation term is neglected in Eq. (4.3a), i.e., $$ \varepsilon = 0 $$, we can easily examine the local stability of the fixed point $$ {{\overset{\hbox{$\smash{\scriptscriptstyle\frown}$}}{\mathbf{m}} }} $$ by checking whether all eigenvalues of $$ {\mathbf{J}} $$ have negative real parts. With the perturbation, however, we also have to compare the magnitudes of those eigenvalues with the perturbation. Moreover, as the perturbation causes a deviation of the community from $$ {{\overset{\lower0.5em\hbox{$\smash{\scriptscriptstyle\frown}$}}{\mathbf{m}} }} $$, the effect of the higher-order term $$ {\mathbf{r}}_{\text{m}} \left| {{\mathbf{m}} - {{\overset{\lower0.5em\hbox{$\smash{\scriptscriptstyle\frown}$}}{{\mathbf{m}}} }}} \right|^{2} $$ has to be examined as well.

To simplify the analysis, we introduce a new vector $$ {\mathbf{x}} = (x_{1} , \ldots ,x_{M} )^{\text{T}} = {\mathbf{P}}({\mathbf{m}} - {{\overset{\lower0.5em\hbox{$\smash{\scriptscriptstyle\frown}$}}{{\mathbf{m}}} }}) $$ with a real matrix $$ {\mathbf{P}} $$ and write Eq. (4.3a) as

4.4 $$ \frac{{{\text{d}}{\mathbf{x}}}}{{{\text{d}}t}} = {\mathbf{Ax}} + {\mathbf{r}}\left| {\mathbf{x}} \right|^{2} + \varepsilon {\mathbf{h}}, $$

with ${\mathbf{A}}: = {\mathbf{PJP}}^{ - 1}$, ${\mathbf{r}}: = {\mathbf{Pr}}_{\text{m}} \left| {{\mathbf{P}}^{ - 1} {\mathbf{x}}} \right|^{2} /\left| {\mathbf{x}} \right|^{2}$, ${\mathbf{h}}: = {\mathbf{Ph}}_{\text{m}}$, $\left| {\mathbf{r}} \right| \le C_{{\mathbf{r}}}$, and $\left| {\mathbf{h}} \right| \le C_{{\mathbf{h}}}$; see Appendix D. As proved in Appendix E, we have

#### **Lemma 4**

*A real matrix*${\mathbf{P}}$*can be chosen so that*${\mathbf{A}} = {\mathbf{PJP}}^{ - 1}$*satisfies*

4.5a $$ {\mathbf{x}}^{\text{T}} {\mathbf{Ax}} \le \lambda_{\rm {max} } \left| {\mathbf{x}} \right|^{2} $$

*for*$$ \lambda_{\rm {max} } < 0 $$*with*

4.5b $$ \lambda_{\rm {max} } : = \hbox{max} \left\{ {\text{Re} (\lambda_{1} ), \ldots ,\text{Re} (\lambda_{M} )} \right\} $$

*(when the eigenvalues*$\lambda_{1} , \ldots ,\lambda_{M}$*of*${\mathbf{J}}$*in Eq.* (4.3b) *are all distinct) or*

4.5c $$ \lambda_{\rm {max} } : = \hbox{max} \left\{ {\text{Re} (\lambda_{1} ), \ldots ,\text{Re} (\lambda_{D} ),\tfrac{1}{2}\text{Re} (\lambda_{D + 1} ), \ldots ,\tfrac{1}{2}\text{Re} (\lambda_{M} )} \right\} $$

*(when some eigenvalues are repeated, with distinct eigenvalues*$\lambda_{1} , \ldots ,\lambda_{D}$*and repeated eigenvalues*$\lambda_{D + 1} , \ldots ,\lambda_{M}$*).*

When the second and third terms in Eq. (4.4) are both neglected, the time derivative of $\left| {\mathbf{x}} \right|^{2}$ is a monotonically decreasing function if $\lambda_{\rm {max} } < 0$, which gives

#### **Lemma 5**

*For Eq.* (4.4) *with*$\varepsilon = 0$*, if*

4.6a $$ \lambda_{\rm {max} } < 0, $$

*then*

4.6b $$ V: = \sum\limits_{i = 1}^{M} {x_{i}^{2} } = \left| {\mathbf{x}} \right|^{2} = {\mathbf{x}}^{\text{T}} {\mathbf{x}} $$

*is a local Lyapunov function, i.e.,*$V = 0$*for*${\mathbf{x}} = {\mathbf{0}}$*and*${\text{d}}V/{\text{d}}t < 0$*for*$0 < \left| {\mathbf{x}} \right| < \phi$*with a sufficiently small*$\phi .$

#### *Proof*

By Eqs. (4.4) and (4.6a), the time derivative of $V$ equals

4.7a $$ \begin{aligned} \frac{{{\text{d}}V}}{{{\text{d}}t}} & = 2{\mathbf{x}}^{\text{T}} \frac{{{\text{d}}{\mathbf{x}}}}{{{\text{d}}t}} \\ & = 2{\mathbf{x}}^{\text{T}} {\mathbf{Ax}} + 2{\mathbf{x}}^{\text{T}} {\mathbf{r}}\left| {\mathbf{x}} \right|^{2} + 2\varepsilon {\mathbf{x}}^{\text{T}} {\mathbf{h}} \\ & \le 2\lambda_{\rm {max} } \left| {\mathbf{x}} \right|^{2} + 2{\mathbf{x}}^{\text{T}} {\mathbf{r}}\left| {\mathbf{x}} \right|^{2} + 2\varepsilon {\mathbf{x}}^{\text{T}} {\mathbf{h}} \\ & \le 2\lambda_{\rm {max} } \left| {\mathbf{x}} \right|^{2} + 2C_{{\mathbf{r}}} \left| {\mathbf{x}} \right|^{3} + 2\varepsilon C_{{\mathbf{h}}} \left| {\mathbf{x}} \right| \\ & = 2C_{{\mathbf{r}}} \left| {\mathbf{x}} \right|^{2} \left( {\left| {\mathbf{x}} \right| - \phi_{\text{r}} } \right) + \lambda_{\rm {max} } \left| {\mathbf{x}} \right|\left( {\left| {\mathbf{x}} \right| - \phi_{\text{h}} } \right), \\ \end{aligned} $$

where

4.7b $$ \begin{aligned} \phi_{\text{r}} & : = \frac{{\left| {\lambda_{\rm {max} } } \right|}}{{2C_{{\mathbf{r}}} }} > 0, \\ \phi_{\text{h}} & : = \frac{{2\varepsilon C_{{\mathbf{h}}} }}{{\left| {\lambda_{\rm {max} } } \right|}} > 0. \\ \end{aligned} $$

Thus, if $\varepsilon = 0$ (i.e., $\phi_{\text{h}} = 0$), then $V = 0$ for ${\mathbf{x}} = {\mathbf{0}}$ and ${\text{d}}V/{\text{d}}t < 0$ for $0 < \left| {\mathbf{x}} \right| < \phi_{\text{r}}$. Therefore, $V$ is a local Lyapunov function for ${\mathbf{x}} = {\mathbf{0}}$. □

### Stability condition under perturbation

**Fig. 2:**
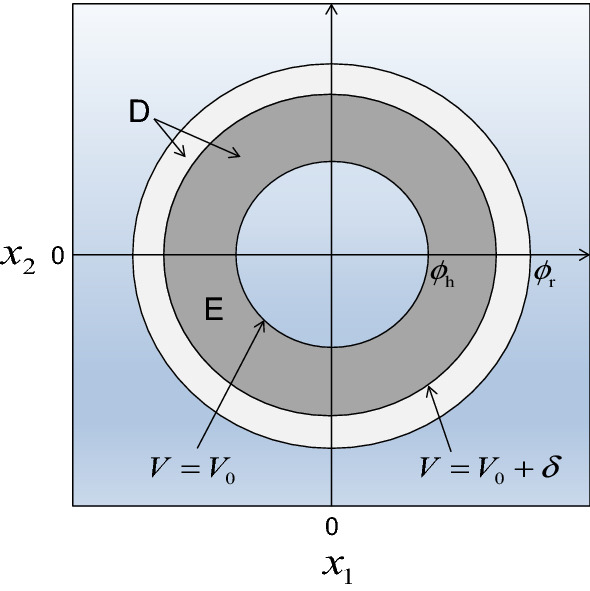
Stability against perturbation when the initial equilibrium population densities of approximate phenotypes are not small. As a simple example, a community composed of two approximate phenotypes ${\mathbf{s}}_{\text{a}} = (s_{1} ,s_{2} )^{\text{T}}$ is considered. Their population densities ${\mathbf{m}} = (m_{1} ,m_{2} )^{\text{T}}$ are transformed into ${\mathbf{x}} = (x_{1} ,x_{2} )^{\text{T}}$ so that ${\mathbf{x}} = {\mathbf{0}}$ corresponds to the initial equilibrium before mutant invasion. A local Lyapunov function $V = \left| {\mathbf{x}} \right|^{2}$ of the community dynamics monotonically decreases with time within the light-gray and dark-gray regions marked by D. The dark-gray region marked by E is associated with a repeller that prevents the community dynamics from passing its inner boundary $\left| {\mathbf{x}} \right| = \phi_{\text{h}}$ from its inner side $\left| {\mathbf{x}} \right| < \phi_{\text{h}}$

For $$ \lambda_{\rm {max} } < 0 $$ and $$ \varepsilon < \lambda_{\rm {max} }^{2} /(4C_{{\mathbf{h}}} C_{{\mathbf{r}}} ) $$, there exists a contour $$ V = V_{0} $$ with $$ \phi_{\text{h}}^{2} < V_{0} < \phi_{\text{r}}^{2} $$ on which $$ {\text{d}}V / {\text{d}}t < 0 $$ (Fig. 2). Hence, all solutions of Eq. (4.4) that start within this contour stay inside of it. As the initial state $$ {{\overset{\lower0.5em\hbox{$\smash{\scriptscriptstyle\frown}$}}{{\mathbf{x}}} }} $$ satisfies $$ {{\overset{\lower0.5em\hbox{$\smash{\scriptscriptstyle\frown}$}}{{\mathbf{x}}} }} = {\mathbf{P}}({{\overset{\lower0.5em\hbox{$\smash{\scriptscriptstyle\frown}$}}{{\mathbf{m}}} }} - {{\overset{\lower0.5em\hbox{$\smash{\scriptscriptstyle\frown}$}}{{\mathbf{m}}} }}) = {\mathbf{0}} $$, we have

#### **Lemma 6**

*If*

4.8a $$ \sqrt \varepsilon < \frac{{ - \lambda_{\rm {max} } }}{{2\sqrt {C_{{\mathbf{h}}} C_{{\mathbf{r}}} } }}, $$

*then*

4.8b $$ \left| {\mathbf{x}} \right| < 2\varepsilon \frac{{C_{{\mathbf{h}}} }}{{\left| {\lambda_{\rm max} } \right|}} $$

*during the transient following mutant invasion.*

Finally, by translating Eq. (4.8b) back to $$ {\mathbf{m}} - {{\overset{\hbox{$\smash{\scriptscriptstyle\frown}$}}{\mathbf{m}} }} = {\mathbf{P}}^{ - 1} {\mathbf{x}} $$, and by substituting it into Eq. (3.4a), we have

#### **Theorem 2**

*For the population densities*${\mathbf{m}} = (m_{1} , \ldots ,m_{M} )^{\text{T}}$*of approximate phenotypes*${\mathbf{s}}_{\text{a}} = (s_{1} , \ldots ,s_{M} )^{\text{T}}$*formed by clustering resident and mutant phenotypes*${\mathbf{s}}^{{\prime }} = (s_{1} , \ldots ,s_{N + 1} )^{\text{T}}$*according to a threshold phenotypic distance*$\varepsilon = \rho_{\mu } \varepsilon_{\mu }$*, if*$\lambda_{\rm max}$*defined by Eq.* (4.5) *satisfies the approximability condition, Eq.* (4.8a),

$$ \sqrt \varepsilon < - \frac{{\lambda_{\rm max} }}{{2\sqrt {C_{\text{h}} C}_{\text{r}} }}, $$

*then*

4.9a $$ \left| {{\mathbf{m}} - {{\overset{\lower0.5em\hbox{$\smash{\scriptscriptstyle\frown}$}}{{\mathbf{m}}} }}} \right| \le C_{{\mathbf{m}}} \varepsilon $$

*during the transient following mutant invasion, where*

4.9b $$ C_{{\mathbf{m}}} = \frac{{2\left\| {{\mathbf{P}}^{ - 1} } \right\|C_{{\mathbf{h}}} }}{{\left| {\lambda_{\rm max} } \right|}} $$

*with*${\mathbf{P}}$*defined by Eq.* (E.24) *of Appendix *E*and with*$\left\| {\, \cdot \,} \right\|$*denoting the induced norm for the matrix⋅, i.e., the maximum absolute value among its eigenvalues.*

In evolutionary dynamics determined by trait-substitution sequences, i.e., induced by repeated mutant invasions, when the fitness gradients for all residents are sufficiently strong, so that the coexistence of a mutant and its parental resident is impossible for any resident (as explained in Sect. 8), each of the resident phenotypes is not similar to any other. Then, $\varepsilon$ may be chosen at $\varepsilon = \varepsilon_{\mu }$ (i.e., $\rho_{\mu } = 1$), so that only the mutant and its parental resident are clustered. This parental resident can be chosen as the approximate phenotype of that cluster, in which case all approximate phenotypes are identical to the resident phenotypes before the mutant invasion. Thus, as long as the initial equilibrium before the invasion is linearly stable, $\lambda_{\rm max}$ is negative, because of Eq. (4.1a) in conjunction with Eq. (2.3). Hence, for sufficiently small $\varepsilon_{\mu }$, Eq. (4.8a) is always satisfied, in accordance with the proof by Dercole and Rinaldi (2008).

On the other hand, when the fitness gradients for some residents become small as a consequence of their directional coevolution toward higher fitnesses, effects of the higher-order properties of the fitness function may induce evolutionary branching. During the early stage of evolutionary branching, phenotypic distances among residents branched from the ancestral resident have magnitudes that are comparable with $\varepsilon_{\mu }$. In this case, clustering only the mutant and its parental resident with $\varepsilon = \varepsilon_{\mu }$ may provide too small a value of $|\lambda_{\rm max} |$ to satisfy Eq. (4.8a), while including similar residents in the cluster for an appropriate $\varepsilon$ larger than $\varepsilon_{\mu }$ may provide a sufficiently large $|\lambda_{\rm max} |$ to satisfy Eq. (4.8a).

In Dercole and Rinaldi (2008), only the mutant and its parental resident are clustered together, and the other residents are not clustered. Thus, when the phenotypic distance among some residents is small, say, equal to $\varepsilon_{\text{resident}}$, the leading eigenvalue of the community’s Jacobian matrix inevitably is close to zero as well. This problem is avoided in their proof by assuming sufficiently small mutational step sizes compared to $\varepsilon_{\text{resident}}$. However, when we consider a trait substitution sequence under a given magnitude of mutational step sizes, early stages of evolutionary branching inevitably lead to $\varepsilon_{\text{resident}}$ of the same order of magnitude as the mutational step sizes, no matter what is assumed for the latter. From this perspective, the proof by Dercole and Rinaldi (2008) requires all the residents to be dissimilar.

According to Eq. (4.3b), the leading eigenvalue also becomes close to zero when initial equilibrium densities of some residents are small. Analogously to the above case of similar residents, while this problem is seemingly avoided in Dercole and Rinaldi (2008) by assuming sufficiently small mutational step sizes, it inevitably occurs in trait-substitution sequences in which residents gradually go extinct. Hence, their proof fails to cover all the cases that one may wish to consider (and are actually considered in their book).

## Approximability condition when the population densities of some approximate phenotypes are small

If an approximate phenotype $$ s_{1} $$ has a small population density $$ \overset{\lower0.5em\hbox{$\smash{\scriptscriptstyle\frown}$}}{m}_{1} = {\text{O}}(\varepsilon ) $$ at the initial equilibrium, then the corresponding eigenvalue of $$ {\mathbf{J}} = {\text{diag}}({{\overset{\lower0.5em\hbox{$\smash{\scriptscriptstyle\frown}$}}{\mathbf{m}} }}){\mathbf{B}} $$ will be close to zero, making it difficult to satisfy the approximability condition in Eq. (4.8a). Even in this case, however, $$ \left| {{\mathbf{m}} - {{\overset{\lower0.5em\hbox{$\smash{\scriptscriptstyle\frown}$}}{\mathbf{m}} }}} \right| = {\text{O}}(\varepsilon ) $$ may hold during the transient following mutant invasion. To cover this situation by developing a refined approximability condition, we examine in this section not only linear terms, but also quadratic terms, of the Taylor expansions investigated in the preceding section.

### Transformation into perturbed community

First, we decompose the function ${\mathbf{f}}$ in Eq. (4.2),

$$ \frac{{{\text{d}}{\mathbf{m}}}}{{{\text{d}}t}} = {\text{diag(}}{\mathbf{m}} ){\mathbf{f}}({\mathbf{s}}_{\text{a}} ;{\mathbf{s}}^{{\prime }} ;{\mathbf{n}}^{{\prime }} ), $$

into the terms that are linear in ${\mathbf{m}}$, the terms that are of higher order in ${\mathbf{m}}$, and the perturbation terms, in a manner similar to Lemma 3 in the previous section. Specifically, we have

#### **Lemma 7**

*Equation* (4.2) *can be transformed into*

5.1 $$ \frac{{{\text{d}}{\mathbf{m}}}}{{{\text{d}}t}} = {\text{diag(}}{\mathbf{m}} )\left[ {{\mathbf{B}}({\mathbf{m}} - {{\overset{\lower0.5em\hbox{$\smash{\scriptscriptstyle\frown}$}}{\mathbf{m}} }}) + \varepsilon {\mathbf{h}}_{\text{f}} + {\mathbf{r}}_{\text{f}} \left| {{\mathbf{m}} - {{\overset{\lower0.5em\hbox{$\smash{\scriptscriptstyle\frown}$}}{\mathbf{m}} }}} \right|^{2} } \right] , $$

*where*${\mathbf{r}}_{\text{f}} = (r_{\text{f1}} , \ldots ,r_{{{\text{f}}M}} )^{\text{T}}$*is a function of*${\mathbf{m}}$*satisfying*$\left| {{\mathbf{r}}_{\text{f}} } \right| \le C_{{{\mathbf{r}}{\text{f}}}}$*and*${\mathbf{h}}_{\text{f}} = (h_{\text{f1}} , \ldots ,h_{{{\text{f}}M}} )^{\text{T}}$*is a function of*${\mathbf{m}}^{{\prime }}$*and*$\varepsilon$*satisfying*$\left| {{\mathbf{h}}_{\text{f}} } \right| \le C_{{{\mathbf{h}}{\text{f}}}} .$

See Appendix C.1 for the proof.

We now consider situations in which $$ L $$ population densities, i.e., $$ \overset{\lower0.5em\hbox{$\smash{\scriptscriptstyle\frown}$}}{m}_{i} $$ for $$ i = 1, \ldots ,L $$, are large, while the remaining $$ K = M - L $$ population densities, i.e., $$ \overset{\lower0.5em\hbox{$\smash{\scriptscriptstyle\frown}$}}{m}_{i} $$ for $$ i = L + 1, \ldots ,M $$, are small, such that $$ \left| {(\overset{\lower0.5em\hbox{$\smash{\scriptscriptstyle\frown}$}}{m}_{L + 1} , \ldots ,\overset{\lower0.5em\hbox{$\smash{\scriptscriptstyle\frown}$}}{m}_{M} )} \right| = \rho_{\text{m}} \varepsilon $$ for a positive constant $$ \rho_{\text{m}} $$. To treat the small population densities differently from the larger ones, we decompose $$ {\mathbf{m}} $$ into the larger population densities $$ {\mathbf{m}}_{\text{x}} = (m_{1} , \ldots ,m_{L} )^{\text{T}} $$ and the small population densities $$ {\mathbf{m}}_{\text{y}} = (m_{L + 1} , \ldots ,m_{M} )^{\text{T}} $$, $$ {\mathbf{m}} = ({\mathbf{m}}_{\text{x}} ,{\mathbf{m}}_{\text{y}} )^{\text{T}} $$. Then, Eq. (5.1) is split into

5.2a $$ \frac{{{\text{d}}{\mathbf{m}}_{\text{x}} }}{{{\text{d}}t}} = {\text{diag(}}{\mathbf{m}}_{\text{x}} )\left[ {{\mathbf{B}}_{\text{xx}} ({\mathbf{m}}_{\text{x}} - {{\overset{\lower0.5em\hbox{$\smash{\scriptscriptstyle\frown}$}}{\mathbf{m}} }}_{\text{x}} ) + {\mathbf{B}}_{\text{xy}} ({\mathbf{m}}_{\text{y}} - {{\overset{\lower0.5em\hbox{$\smash{\scriptscriptstyle\frown}$}}{\mathbf{m}} }}_{\text{y}} ) + \varepsilon {\mathbf{h}}_{\text{fx}} + {\mathbf{r}}_{\text{fx}} \left| {{\mathbf{m}} - {\mathbf{\overset{\lower0.5em\hbox{$\smash{\scriptscriptstyle\frown}$}}{m} }}} \right|^{2} } \right] $$

and

5.2b $$ \frac{{{\text{d}}{\mathbf{m}}_{\text{y}} }}{{{\text{d}}t}} = {\text{diag(}}{\mathbf{m}}_{\text{y}} )\left[ {{\mathbf{B}}_{\text{yx}} ({\mathbf{m}}_{\text{x}} - {{\overset{\lower0.5em\hbox{$\smash{\scriptscriptstyle\frown}$}}{{\mathbf{m}}} }}_{\text{x}} ) + {\mathbf{B}}_{\text{yy}} ({\mathbf{m}}_{\text{y}} - {{\overset{\lower0.5em\hbox{$\smash{\scriptscriptstyle\frown}$}}{{\mathbf{m}}} }}_{\text{y}} ) + \varepsilon {\mathbf{h}}_{\text{fy}} + {\mathbf{r}}_{\text{fy}} \left| {{\mathbf{m}} - {{\overset{\lower0.5em\hbox{$\smash{\scriptscriptstyle\frown}$}}{{\mathbf{m}}} }}} \right|^{2} } \right] , $$

with

5.2c $$ \left( {\begin{array}{*{20}c} {{\mathbf{B}}_{\text{xx}} } & {{\mathbf{B}}_{\text{xy}} } \\ {{\mathbf{B}}_{\text{yx}} } & {{\mathbf{B}}_{\text{yy}} } \\ \end{array} } \right) = {\mathbf{B}},\quad \left( {\begin{array}{*{20}c} {{\mathbf{h}}_{\text{fx}} } \\ {{\mathbf{h}}_{\text{fy}} } \\ \end{array} } \right) = {\mathbf{h}}_{\text{f}} ,\quad \left( {\begin{array}{*{20}c} {{\mathbf{r}}_{\text{fx}} } \\ {{\mathbf{r}}_{\text{fy}} } \\ \end{array} } \right) = {\mathbf{r}}_{\text{f}} . $$

Around the initial equilibrium $$ {\mathbf{\overset{\hbox{$\smash{\scriptscriptstyle\frown}$}}{m} }} $$, the dynamics of $$ {\mathbf{m}}_{\text{y}} $$ are much slower than those of $$ {\mathbf{m}}_{\text{x}} $$. When $$ \varepsilon $$ goes to zero, the equilibrium population densities $$ \left| {(\overset{\lower0.5em\hbox{$\smash{\scriptscriptstyle\frown}$}}{m}_{L + 1} , \ldots ,\overset{\lower0.5em\hbox{$\smash{\scriptscriptstyle\frown}$}}{m}_{M} )} \right| \le \rho_{\text{m}} \varepsilon $$ do so as well, i.e.,

5.3a $$ {{\overset{\lower0.5em\hbox{$\smash{\scriptscriptstyle\frown}$}}{{\mathbf{m}}} }} \to (\overset{\lower0.5em\hbox{$\smash{\scriptscriptstyle\frown}$}}{m}_{1} , \ldots ,\overset{\lower0.5em\hbox{$\smash{\scriptscriptstyle\frown}$}}{m}_{L} ,0, \ldots ,0)^{\text{T}} = \left( {\begin{array}{*{20}c} {{{\overset{\lower0.5em\hbox{$\smash{\scriptscriptstyle\frown}$}}{{\mathbf{m}}} }}_{\text{x}} } \\ {\mathbf{0}} \\ \end{array} } \right) = :{{\overset{\lower0.5em\hbox{$\smash{\scriptscriptstyle\frown}$}}{\tilde{{\mathbf{m}}}} }}, $$

and the whole dynamics get confined to a center manifold given by $$ {\text{d}}{\mathbf{m}}_{\text{x}} / {\text{d}}t = 0 $$. The slow dynamics close to the center manifold are governed by $$ {\text{d}}{\mathbf{m}}_{\text{y}} / {\text{d}}t $$. An approximation for this center manifold can be derived by setting $$ {\text{d}}{\mathbf{m}}_{\text{x}} / {\text{d}}t = 0 $$ with $$ \varepsilon {\mathbf{h}}_{\text{fx}} + {\mathbf{r}}_{\text{fx}} \left| {{\mathbf{m}} - {{\overset{\lower0.5em\hbox{$\smash{\scriptscriptstyle\frown}$}}{{\mathbf{m}}} }}} \right|^{2} = 0 $$, yielding

5.3b $$ {\mathbf{m}}_{\text{x}} = {{\overset{\lower0.5em\hbox{$\smash{\scriptscriptstyle\frown}$}}{{\mathbf{m}}} }}_{\text{x}} - {\mathbf{B}}_{\text{xx}}^{ - 1} {\mathbf{B}}_{\text{xy}} {\mathbf{m}}_{\text{y}} = :{\tilde{\mathbf{m}}}_{\text{x}} ({\mathbf{m}}_{\text{y}} ) , $$

which passes through the fixed point $$ {\tilde{\mathbf{m}}}_{\text{x}} ({\mathbf{0}}) = :{{\overset{\lower0.5em\hbox{$\smash{\scriptscriptstyle\frown}$}}{\tilde{{\mathbf{m}}}} }}. $$

Although for $$ \varepsilon > 0 $$, $$ {{\overset{\lower0.5em\hbox{$\smash{\scriptscriptstyle\frown}$}}{{\mathbf{m}}} }} = ({{\overset{\lower0.5em\hbox{$\smash{\scriptscriptstyle\frown}$}}{{\mathbf{m}}} }}_{\text{x}} ,{{\overset{\lower0.5em\hbox{$\smash{\scriptscriptstyle\frown}$}}{{\mathbf{m}}} }}_{\text{y}} )^{\text{T}} $$ will deviate from $$ {{\overset{\lower0.5em\hbox{$\smash{\scriptscriptstyle\frown}$}}{\tilde{{\mathbf{m}}}} }} = ({{\overset{\lower0.5em\hbox{$\smash{\scriptscriptstyle\frown}$}}{{\mathbf{m}}} }}_{\text{x}} ,{\mathbf{0}})^{\text{T}} $$, it is expected that for small $$ \varepsilon $$ the dynamics can still be effectively characterized by their projection onto the center manifold $$ {\mathbf{m}}_{\text{x}} = {\tilde{\mathbf{m}}}_{\text{x}} ({\mathbf{m}}_{\text{y}} ) $$. Thus, we transform Eq. (5.2a) into

5.4a $$ \frac{{{\text{d}}{\mathbf{x}}}}{{{\text{d}}t}} = {\mathbf{A}}_{\text{x}} {\mathbf{x}} + \varepsilon {\tilde{\mathbf{h}}}_{\text{x}} + {\tilde{\mathbf{r}}}_{\text{x}} \left| {\mathbf{w}} \right|^{2} , $$

5.4b $$ \frac{{{\text{d}}{\mathbf{y}}}}{{{\text{d}}t}}{\text{ = diag(}}{\mathbf{y}} )\left[ {{\mathbf{J}}_{\text{y}} {\mathbf{y}} + {\mathbf{Ux}} + \varepsilon {\tilde{\mathbf{h}}}_{\text{y}} + {\tilde{\mathbf{r}}}_{\text{y}} \left| {\mathbf{w}} \right|^{2} } \right], $$

with $\left| {{\tilde{\mathbf{h}}}_{\text{x}} } \right| \le \tilde{C}_{{{\mathbf{h}}{\text{x}}}}$, $\left| {{\tilde{\mathbf{h}}}_{\text{y}} } \right| \le \tilde{C}_{{{\mathbf{h}}{\text{y}}}}$, $\left| {{\tilde{\mathbf{r}}}_{\text{x}} } \right| \le \tilde{C}_{{{\mathbf{r}}{\text{x}}}}$, and $\left| {{\tilde{\mathbf{r}}}_{\text{y}} } \right| \le \tilde{C}_{{{\mathbf{r}}{\text{y}}}}$, where ${\mathbf{x}}: = {\mathbf{P}}({\mathbf{m}}_{\text{x}} - {\tilde{\mathbf{m}}}_{\text{x}} ({\mathbf{m}}_{\text{y}} ))$ describes the convergence to, or deviation from, the center manifold ${\mathbf{m}}_{\text{x}} = {\tilde{\mathbf{m}}}_{\text{x}} ({\mathbf{m}}_{\text{y}} )$, and ${\mathbf{y}}: = {\mathbf{m}}_{\text{y}}$ describes the slow dynamics along the manifold. The other variables and parameters newly introduced in Eqs. (5.4a) and (5.4b) are given by

5.4c $$ \begin{aligned} {\mathbf{w}} & : = \left( {\begin{array}{*{20}c} {\mathbf{x}} \\ {\mathbf{y}} \\ \end{array} } \right), \\ {\mathbf{A}}_{\text{x}} & : = {\mathbf{PJ}}_{\text{x}} {\mathbf{P}}^{ - 1} = {\mathbf{P}}{\text{diag(}}{{\overset{\lower0.5em\hbox{$\smash{\scriptscriptstyle\frown}$}}{{\mathbf{m}}} }}_{\text{x}} ){\mathbf{B}}_{\text{xx}} {\mathbf{P}}^{ - 1} , \\ {\mathbf{U}} & : = {\mathbf{B}}_{\text{yx}} {\mathbf{P}}^{ - 1} , \\ {\mathbf{J}}_{\text{y}} & : = {\mathbf{B}}_{\text{yy}} - {\mathbf{B}}_{\text{yx}} {\mathbf{B}}_{\text{xx}}^{ - 1} {\mathbf{B}}_{\text{xy}} , \\ \end{aligned} $$

(Appendix F.1–F.4), where $$ {\mathbf{P}} $$ is chosen so that Eq. (4.5a) is satisfied for $$ {\mathbf{A}} = {\mathbf{A}}_{\text{x}} $$ (Appendix E). Notice that $$ {\mathbf{y}} $$ must be non-negative while $$ {\mathbf{x}} $$ is indeterminate. Here, the effect of $$ {{\overset{\lower0.5em\hbox{$\smash{\scriptscriptstyle\frown}$}}{{\mathbf{m}}} }} - {{\overset{\lower0.5em\hbox{$\smash{\scriptscriptstyle\frown}$}}{\tilde{{\mathbf{m}}}} }} $$ is subsumed into the perturbation terms $$ {\tilde{\mathbf{h}}}_{\text{x}} $$ and $$ {\tilde{\mathbf{h}}}_{\text{y}} $$. Neglecting them gives a fixed point $$ {\mathbf{w}} = \left( {\begin{array}{*{20}c} {\mathbf{x}} \\ {\mathbf{y}} \\ \end{array} } \right) = {\mathbf{0}} $$ that corresponds to $$ {{\overset{\lower0.5em\hbox{$\smash{\scriptscriptstyle\frown}$}}{\tilde{{\mathbf{m}}}} }} = ({{\overset{\lower0.5em\hbox{$\smash{\scriptscriptstyle\frown}$}}{{\mathbf{m}}} }}_{\text{x}} ,{\mathbf{0}})^{\text{T}} $$, which is slightly different from the initial equilibrium $$ {\mathbf{\overset{\lower0.5em\hbox{$\smash{\scriptscriptstyle\frown}$}}{m} }} = ({\mathbf{\overset{\lower0.5em\hbox{$\smash{\scriptscriptstyle\frown}$}}{m} }}_{\text{x}} ,{\mathbf{\overset{\lower0.5em\hbox{$\smash{\scriptscriptstyle\frown}$}}{m} }}_{\text{y}} )^{\text{T}} $$ when $$ \varepsilon > 0 $$. In the next subsections, we analyze the magnitude of $$ \left| {{\mathbf{m}} - {{\overset{\lower0.5em\hbox{$\smash{\scriptscriptstyle\frown}$}}{\tilde{\mathbf{m}}} }}} \right| $$ in Eq. (5.4a) during the transient following mutant invasion, to obtain the magnitude of $$ \left| {{\mathbf{m}} - {\mathbf{\overset{\lower0.5em\hbox{$\smash{\scriptscriptstyle\frown}$}}{m} }}} \right| = \left| {{\mathbf{m}} - {{\overset{\lower0.5em\hbox{$\smash{\scriptscriptstyle\frown}$}}{\tilde{\mathbf{m}}} }}} \right| + O(\varepsilon ) $$.

### Local Lyapunov function

Following Mazenc (2001), we construct a local Lyapunov function to examine the magnitude of $\left| {\mathbf{w}} \right|$ during the transient following mutant invasion. We have

#### **Lemma 8**

*In Eq.* (5.4a) *with*$\varepsilon = 0$*, if the eigenvalues*$\tilde{\lambda }_{1} , \ldots ,\tilde{\lambda }_{M}$*of the symmetric part of*

5.5a $$ \,{\tilde{\mathbf{A}}} = \left( {\begin{array}{ll} {{\mathbf{A}}_{\text{x}} } \hfill &\quad 0 \hfill \\ {d{\mathbf{U}}} \hfill &\quad {d{\mathbf{J}}_{\text{y}} } \hfill \\ \end{array} } \right), $$

*with*$d$*being a positive constant, satisfy*

5.5b $$ \tilde{\lambda }_{\rm max} = \hbox{max} \left\{ {\tilde{\lambda }_{1} , \ldots ,\tilde{\lambda }_{M} } \right\} < 0 $$

*and*$\left| {\mathbf{w}} \right| < \phi$*, with*$\phi$*being a sufficiently small constant, then*

5.5c $$ \begin{aligned} V & = \sum\limits_{i = 1}^{L} {x_{i}^{2} } + 2d\sum\limits_{i = L + 1}^{M} {y_{i} } \\ & = {\mathbf{x}}^{\text{T}} {\mathbf{x}} + 2d{\mathbf{c}}^{\text{T}} {\mathbf{y}} \\ \end{aligned} $$

*with*${\mathbf{c}} = (1, \ldots ,1)^{\text{T}}$*is a local Lyapunov function.*

#### *Proof*

We assume that $\tilde{\lambda }_{\rm max} < 0$. The time derivative of $V$ is

5.6a $$ \begin{aligned} \frac{{{\text{d}}V}}{{{\text{d}}t}} & = 2{\mathbf{x}}^{\text{T}} \frac{{{\text{d}}{\mathbf{x}}}}{{{\text{d}}t}} + 2d{\mathbf{c}}^{\text{T}} \frac{{{\text{d}}{\mathbf{y}}}}{{{\text{d}}t}} \\ & = 2{\mathbf{x}}^{\text{T}} \left[ {{\mathbf{A}}_{\text{x}} {\mathbf{x}} + \varepsilon {\tilde{\mathbf{h}}}_{\text{x}} + {\tilde{\mathbf{r}}}_{\text{x}} \left| {\mathbf{w}} \right|^{2} } \right] + 2d{\mathbf{c}}^{\text{T}} {\text{diag(}}{\mathbf{y}} )\left[ {{\mathbf{J}}_{\text{y}} {\mathbf{y}} + {\mathbf{Ux}} + \varepsilon {\tilde{\mathbf{h}}}_{\text{y}} + {\tilde{\mathbf{r}}}_{\text{y}} \left| {\mathbf{w}} \right|^{2} } \right] \\ & = 2{\mathbf{x}}^{\text{T}} \left[ {{\mathbf{A}}_{\text{x}} {\mathbf{x}} + \varepsilon {\tilde{\mathbf{h}}}_{\text{x}} + {\tilde{\mathbf{r}}}_{\text{x}} \left| {\mathbf{w}} \right|^{2} } \right] + 2d{\mathbf{y}}^{\text{T}} \left[ {{\mathbf{J}}_{\text{y}} {\mathbf{y}} + {\mathbf{Ux}} + \varepsilon {\tilde{\mathbf{h}}}_{\text{y}} + {\tilde{\mathbf{r}}}_{\text{y}} \left| {\mathbf{w}} \right|^{2} } \right] \\ & = 2\left( {\begin{array}{*{20}c} {\mathbf{x}} \\ {\mathbf{y}} \\ \end{array} } \right)^{\text{T}} \left( {\begin{array}{*{20}c} {{\mathbf{A}}_{\text{x}} } & 0 \\ {d{\mathbf{U}}} & {d{\mathbf{J}}_{\text{y}} } \\ \end{array} } \right)\left( {\begin{array}{*{20}c} {\mathbf{x}} \\ {\mathbf{y}} \\ \end{array} } \right) + 2\left( {\begin{array}{*{20}c} {\mathbf{x}} \\ {\mathbf{y}} \\ \end{array} } \right)^{\text{T}} \left( {\begin{array}{*{20}c} {{\tilde{\mathbf{r}}}_{\text{x}} } \\ {d{\tilde{\mathbf{r}}}_{\text{y}} } \\ \end{array} } \right)\left| {\mathbf{w}} \right|^{2} + 2\varepsilon \left( {\begin{array}{*{20}c} {\mathbf{x}} \\ {\mathbf{y}} \\ \end{array} } \right)^{\text{T}} \left( {\begin{array}{*{20}c} {{\tilde{\mathbf{h}}}_{\text{x}} } \\ {d{\tilde{\mathbf{h}}}_{\text{y}} } \\ \end{array} } \right) \\ & = 2{\mathbf{w}}^{\text{T}} {\tilde{\mathbf{A}}\mathbf{w}} + 2{\mathbf{w}}^{\text{T}} {\tilde{\mathbf{r}}}\left| {\mathbf{w}} \right|^{2} + 2\varepsilon {\mathbf{w}}^{\text{T}} {\tilde{\mathbf{h}}}, \\ \end{aligned} $$

with

5.6b $$ {\tilde{\mathbf{r}}} = \left( {\begin{array}{*{20}c} {{\mathbf{r}}_{\text{x}} } \\ {d{\mathbf{r}}_{\text{y}} } \\ \end{array} } \right),\quad {\tilde{\mathbf{h}}} = \left( {\begin{array}{*{20}c} {{\mathbf{h}}_{\text{x}} } \\ {d{\mathbf{h}}_{\text{y}} } \\ \end{array} } \right). $$

Notice that the last line of Eq. (5.6a) has a form identical to the second line of Eq. (4.7a). Although in this case the initial state $$ {\mathbf{\overset{\lower0.5em\hbox{$\smash{\scriptscriptstyle\frown}$}}{w} }}: = (0, \ldots ,0,\overset{\lower0.5em\hbox{$\smash{\scriptscriptstyle\frown}$}}{m}_{L + 1} , \ldots ,\overset{\lower0.5em\hbox{$\smash{\scriptscriptstyle\frown}$}}{m}_{M} )^{\text{T}} $$ is not zero, $$ \left| {{\mathbf{\overset{\lower0.5em\hbox{$\smash{\scriptscriptstyle\frown}$}}{w} }}} \right| = \left| {(0, \ldots ,0,\overset{\lower0.5em\hbox{$\smash{\scriptscriptstyle\frown}$}}{m}_{L + 1} , \ldots ,\overset{\lower0.5em\hbox{$\smash{\scriptscriptstyle\frown}$}}{m}_{M} )} \right| = \varepsilon \rho_{\text{m}} $$ holds. Therefore, Eq. (5.6a) can analogously be transformed further,

5.6c $$ \begin{aligned} \frac{{{\text{d}}V}}{{{\text{d}}t}} & \le 2\tilde{\lambda }_{\rm max} \left| {\mathbf{w}} \right|^{2} + 2\tilde{C}_{{\mathbf{r}}} \left| {\mathbf{w}} \right|^{3} + 2\varepsilon \tilde{C}_{{\mathbf{h}}} \left| {\mathbf{w}} \right| \\ & = 2\tilde{C}_{{\mathbf{r}}} \left| {\mathbf{w}} \right|^{2} \left( {\left| {\mathbf{w}} \right| - \frac{{\left| {\tilde{\lambda }_{\rm max} } \right|}}{{2\tilde{C}_{{\mathbf{r}}} }}} \right) + \tilde{\lambda }_{\rm max} \left| {\mathbf{w}} \right|\left( {\left| {\mathbf{w}} \right| - \frac{{2\varepsilon \tilde{C}_{{\mathbf{h}}} }}{{\left| {\tilde{\lambda }_{\rm max} } \right|}}} \right) \\ & \le 2\tilde{C}_{{\mathbf{r}}} \left| {\mathbf{w}} \right|^{2} \left( {\left| {\mathbf{w}} \right| - \frac{{\left| {\tilde{\lambda }_{\rm max} } \right|}}{{2\tilde{C}_{{\mathbf{r}}} }}} \right) + \tilde{\lambda }_{\rm max} \left| {\mathbf{w}} \right|\left( {\left| {\mathbf{w}} \right| - \hbox{max} \left\{ {\frac{{2\varepsilon \tilde{C}_{{\mathbf{h}}} }}{{\left| {\tilde{\lambda }_{\rm max} } \right|}},\varepsilon \rho_{\text{m}} } \right\}} \right) \\ & = 2\tilde{C}_{{\mathbf{r}}} \left| {\mathbf{w}} \right|^{2} \left( {\left| {\mathbf{w}} \right| - \tilde{\phi }_{\text{r}} } \right) + \tilde{\lambda }_{\rm max} \left| {\mathbf{w}} \right|\left( {\left| {\mathbf{w}} \right| - \tilde{\phi }_{\text{h}} } \right), \\ \end{aligned} $$

where $\tilde{C}_{{\mathbf{h}}} \ge \left| {{\tilde{\mathbf{h}}}} \right|$, $\tilde{C}_{{\mathbf{r}}} \ge \left| {{\tilde{\mathbf{r}}}} \right|$ (Appendix F.5) and

5.6d $$ \begin{aligned} \tilde{\phi }_{\text{h}} & = \hbox{max} \left\{ {\frac{{2\varepsilon \tilde{C}_{{\mathbf{h}}} }}{{\left| {\tilde{\lambda }_{\rm max} } \right|}},\varepsilon \rho_{\text{m}} } \right\}, \\ \tilde{\phi }_{\text{r}} & = \frac{{\left| {\tilde{\lambda }_{\rm max} } \right|}}{{2\tilde{C}_{{\mathbf{r}}} }}. \\ \end{aligned} $$

In Eq. (5.6c), the transformation from the second to the third row allows the initial state $$ {\mathbf{\overset{\lower0.5em\hbox{$\smash{\scriptscriptstyle\frown}$}}{w} }} $$ to satisfy $$ \left| {{\mathbf{\overset{\lower0.5em\hbox{$\smash{\scriptscriptstyle\frown}$}}{w} }}} \right| = \varepsilon \rho_{\text{m}} \le \tilde{\phi }_{\text{h}} $$, which is used for the case of $$ \varepsilon > 0 $$ in Lemma 10. Therefore, for $$ \varepsilon = 0 $$ (i.e., $$ \tilde{\phi }_{\text{h}} = 0 $$), $$ V $$ satisfies $$ V = 0 $$ for $$ {\mathbf{w}} = {\mathbf{0}} $$ and $$ {\text{d}}V/{\text{d}}t < 0 $$ for $$ \left| {\mathbf{w}} \right| < \tilde{\phi }_{\text{r}} $$. □

In addition, the following lemma is proved in Appendix G.

#### **Lemma 9**

*Equation* (5.5b), *i.e.,*

$$ \tilde{\lambda }_{\rm max} < 0, $$

*holds, if the real parts of the eigenvalues of*$$ {\mathbf{J}}_{\text{x}} = {\text{diag(}}{\mathbf{\overset{\lower0.5em\hbox{$\smash{\scriptscriptstyle\frown}$}}{m} }}_{\text{x}} ){\mathbf{B}}_{\text{xx}} $$*and the real eigenvalues of*$$ \tfrac{1}{2}\left( {{\mathbf{J}}_{\text{y}} + {\mathbf{J}}_{\text{y}}^{\text{T}} } \right) $$*with*$$ {\mathbf{J}}_{\text{y}} = {\mathbf{B}}_{\text{yy}} - {\mathbf{B}}_{\text{yx}} {\mathbf{B}}_{\text{xx}}^{ - 1} {\mathbf{B}}_{\text{xy}} $$*are all negative, and if*$$ d $$*is sufficiently small.*

### Stability under perturbation

Next, we take the perturbation into account, i.e., we consider $$ \varepsilon > 0 $$. In the previous section, the contour curves of the local Lyapunov function have the same shapes as the boundaries of the region ensuring that $$ {\text{d}}V / {\text{d}}t < 0 $$, i.e., as the two circles $$ \left| {\mathbf{w}} \right| = \phi_{\text{h}} $$ and $$ \left| {\mathbf{w}} \right| = \phi_{\text{r}} $$. In this section, although the contours have shapes different from the circles $$ \left| {\mathbf{w}} \right| = \tilde{\phi }_{\text{h}} $$ and $$ \left| {\mathbf{w}} \right| = \tilde{\phi }_{\text{r}} $$, the manner of analysis is the same. First, as the initial state $$ {{\overset{\lower0.5em\hbox{$\smash{\scriptscriptstyle\frown}$}}{\mathbf{w}} }} $$ satisfies $$ \left| {{\overset{\lower0.5em\hbox{$\smash{\scriptscriptstyle\frown}$}}{\mathbf{w} }}} \right| = \varepsilon \rho_{\text{m}} \le \tilde{\phi }_{\text{h}} $$ according to Eq. (5.6d), we trivially have

#### **Lemma 10**

**Fig. 3:**
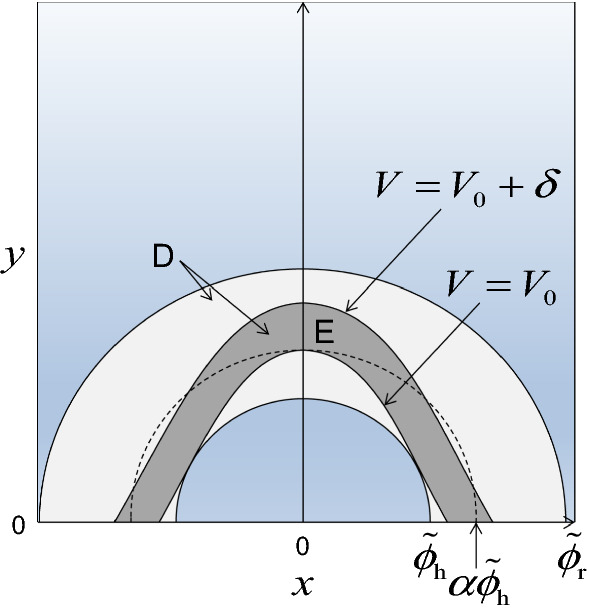
Stability against perturbation when the initial equilibrium population densities of some approximate phenotypes are small. As a simple example, a community composed of two approximate phenotypes ${\mathbf{s}}_{\text{a}} = (s_{1} ,s_{2} )^{\text{T}}$ is considered. Their population densities ${\mathbf{m}} = (m_{1} ,m_{2} )^{\text{T}}$ are transformed into ${\mathbf{w}} = (x,y)^{\text{T}}$ so that ${\mathbf{w}} = {\mathbf{0}}$ corresponds to the initial equilibrium before mutant invasion, where $y$ corresponds to the population density of the phenotype with small population density. A local Lyapunov function $V = x^{2} + dy$ (with $d > 0$) of the community dynamics monotonically decreases with time within the light-gray and dark-gray regions marked by D. The dark-gray region marked by E is associated with a repeller that prevents the community dynamics from passing a boundary $V = V_{0}$ from its inner side $V < V_{0}$, thus keeping $\left| {\mathbf{w}} \right| \le \alpha \tilde{\phi }_{\text{h}}$

*We assume that*$\varepsilon$*is sufficiently small so that*$\tilde{\phi }_{\text{h}} < \tilde{\phi }_{\text{r}}$*. For a region*$D = \left\{ {\left. {\mathbf{w}} \right|\,\,\,\tilde{\phi }_{\text{h}} < \left| {\mathbf{w}} \right| < \tilde{\phi }_{\text{r}} } \right\}$*, within which*${\text{d}}V/{\text{d}}t < 0$*with*$V$*defined in Eq.* (5.5c)*, consider a contour curve*$V = V_{0}$*such that its inscribed circle is given by*$\left| {\mathbf{w}} \right| = \tilde{\phi }_{\text{h}}$*and its circumscribed circle is given by*$\left| {\mathbf{w}} \right| = \alpha \tilde{\phi }_{\text{h}}$*with*$\alpha > 1$ (Fig. 3)*. If*

5.7a $$ \alpha \tilde{\phi }_{\text{h}} < \tilde{\phi }_{\text{r}} , $$

*then there exists a set*$E = \left\{ {\left. {\mathbf{w}} \right|\,\,\,V_{0} < V < V_{0} + \delta } \right\}$*with a sufficiently small*$\delta$*where*${\text{d}}V/{\text{d}}t < 0$*and which therefore ensures that*

5.7b $$ \left| {\mathbf{w}} \right| \le \alpha \tilde{\phi }_{\text{h}} $$

*holds during the transient following mutant invasion.*

This lemma is an extension of Lemma 5 relaxing the requirement that the contours be circular: Lemma 10 with $\alpha = 1$ corresponds to Lemma 5.

Then, by substituting Eqs. (5.6d) into (5.7a), we have

#### **Lemma 11**

*For some* $d$*, if*

5.8a $$ \sqrt \varepsilon < \frac{{ - \tilde{\lambda }_{\rm max} }}{{2\sqrt {\alpha \tilde{C}_{{\mathbf{r}}} \hbox{max} \left\{ {\tilde{C}_{{\mathbf{h}}} ,\tfrac{1}{2}\left| {\tilde{\lambda }_{\rm max} } \right|\rho_{\text{m}} } \right\}} }} , $$

*then*

5.8b $$ \left| {\mathbf{w}} \right| \le - \frac{2\varepsilon \alpha }{{\left| {\tilde{\lambda }_{\rm max} } \right|}}\hbox{max} \left\{ {\tilde{C}_{{\mathbf{h}}} ,\,\tfrac{1}{2}\,\left| {\tilde{\lambda }_{\rm max} } \right|\rho_{\text{m}} } \right\} $$

*holds during the transient following mutant invasion, where*

5.8c $$ \alpha = \left\{ {\begin{array}{ll} 1 \hfill &\quad {{\text{for}}\;\;K = 0} \hfill \\ {\hbox{max} \left\{ {\frac{{\sqrt {\tilde{\phi }_{\text{h}}^{ 2} + Kd^{2} } }}{{\tilde{\phi }_{\text{h}} }},\frac{{\tilde{\phi }_{\text{h}}^{ 2} + Kd^{2} }}{{2d\tilde{\phi }_{\text{h}} }}} \right\}} \hfill &\quad {{\text{for}}\;\;0 < K \le \frac{{\tilde{\phi }_{\text{h}}^{ 2} }}{{d^{2} }}} \hfill \\ {\hbox{max} \left\{ {\frac{{\sqrt {2dK^{1/2} \tilde{\phi }_{\text{h}} } }}{{\tilde{\phi }_{\text{h}} }},\sqrt K } \right\}} \hfill &\quad {{\text{for}}\;\;K > \frac{{\tilde{\phi }_{\text{h}}^{ 2} }}{{d^{2} }}} \hfill \\ \end{array} } \right. , $$

*and* $K = M - L$*is the number of approximate phenotypes with small equilibrium population densities.*

See Appendix H for the derivation of the expressions for $\alpha$. When $K = 0$, this lemma is independent of $d$ and becomes identical to Lemma 6 in the previous section, i.e., $\tilde{\lambda }_{\rm max} = \lambda_{\rm max}$, $\tilde{C}_{{\mathbf{r}}} = C_{{\mathbf{r}}}$, $\tilde{C}_{{\mathbf{h}}} = C_{{\mathbf{h}}}$, and $\alpha = 1$. Thus, this lemma includes Lemma 6 as a special case. When $K > 0$, on the other hand, $\tilde{\lambda }_{\rm max}$, $\alpha$, $\tilde{C}_{\text{h}}$, and $\tilde{C}_{\text{r}}$ in Eq. (5.8a) all depend on $d$. A choice for $d$ may be one that maximizes the right-hand side of Eq. (5.8a).

By translating Lemma 11 into a corresponding statement for ${\mathbf{m}}$, we get

#### **Theorem 3**

*For the population densities*${\mathbf{m}} = (m_{1} , \ldots ,m_{M} )^{\text{T}}$*of approximate phenotypes*${\mathbf{s}}_{\text{a}} = (s_{1} , \ldots ,s_{M} )^{\text{T}}$*formed by clustering resident phenotypes*${\mathbf{s}}^{{\prime }} = (s_{1} , \ldots ,s_{N + 1} )^{\text{T}}$*according to a threshold phenotypic distance*$\varepsilon = \rho_{\mu } \varepsilon_{\mu }$*, if the approximability condition, Eq.* (5.8a),

$$ \sqrt \varepsilon < \frac{{ - \tilde{\lambda }_{\rm max} }}{{2\sqrt {\alpha \tilde{C}_{\text{r}} \hbox{max} \left\{ {\tilde{C}_{{\mathbf{h}}} ,\,\tfrac{1}{2}\,\left| {\tilde{\lambda }_{\rm max} } \right|\rho_{\text{m}} } \right\}} }} $$

*is satisfied for some*$d$*, then*

5.9a $$ \begin{aligned} \left| {{\mathbf{m}} - {{\overset{\lower0.5em\hbox{$\smash{\scriptscriptstyle\frown}$}}{{\mathbf{m}}} }}} \right| & = \left| {{\mathbf{Q}}^{ - 1} {\mathbf{w}} + {{\overset{\lower0.5em\hbox{$\smash{\scriptscriptstyle\frown}$}}{{\mathbf{m}}} }} - {{\overset{\lower0.5em\hbox{$\smash{\scriptscriptstyle\frown}$}}{\tilde{{\mathbf{m}}}} }}} \right| \le C_{{\mathbf{m}}} \varepsilon , \\ C_{{\mathbf{m}}} & = \frac{{2\alpha \left\| {{\mathbf{Q}}^{ - 1} } \right\|\hbox{max} \left\{ {\tilde{C}_{{\mathbf{h}}} ,\, - \tfrac{1}{2}\,\tilde{\lambda }_{\rm max} \rho_{\text{m}} } \right\}}}{{\left| {\tilde{\lambda }_{\rm max} } \right|}} + \rho_{\text{m}} , \\ \end{aligned} $$

*holds during the transient following mutant invasion, where*$$ {\mathbf{m}} $$*is split into an*$$ L $$-*dimensional vector*$$ {\mathbf{m}}_{\text{x}} = (m_{1} , \ldots ,m_{L} )^{\text{T}} $$*of not*-*small initial population densities, and a*$$ K( = M - L) $$-*dimensional vector*$$ {\mathbf{m}}_{\text{y}} = (m_{L + 1} , \ldots ,m_{M} )^{\text{T}} $$*of small initial population densities*$$ \overset{\lower0.5em\hbox{$\smash{\scriptscriptstyle\frown}$}}{m}_{L + 1} , \ldots ,\overset{\lower0.5em\hbox{$\smash{\scriptscriptstyle\frown}$}}{m}_{M} $$*such that*$$ \left| {\left( {\overset{\lower0.5em\hbox{$\smash{\scriptscriptstyle\frown}$}}{m}_{L + 1} , \ldots ,\overset{\lower0.5em\hbox{$\smash{\scriptscriptstyle\frown}$}}{m_{M}} } \right)} \right| = \varepsilon \rho_{\text{m}} $$*(*$$ \rho_{\text{m}} = 0 $$*when all initial population densities are not small, i.e.,*$$ K = 0 $$*), and*$$ \tilde{\lambda }_{1} , \ldots ,\tilde{\lambda }_{M} $$*are the eigenvalues of*$$ \tfrac{1}{2}\left( {{\tilde{\mathbf{A}}} + {\tilde{\mathbf{A}}}^{\text{T}} } \right) $$*with*

5.9b $$ \begin{aligned} & {\tilde{\mathbf{A}}} = \left( {\begin{array}{cc} {{\mathbf{A}}_{\text{x}} } &\quad 0 \\ {d{\mathbf{B}}_{\text{yx}} {\mathbf{P}}^{ - 1} } &\quad {d{\mathbf{J}}_{\text{y}} } \\ \end{array} } \right), \\ & {\mathbf{A}}_{\text{x}} = {\mathbf{P}}^{ - 1} {\text{diag(}}{{\overset{\lower0.5em\hbox{$\smash{\scriptscriptstyle\frown}$}}{{\mathbf{m}}_{\text{x}}} }} ){\mathbf{B}}_{\text{xx}} {\mathbf{P}}, \\ & {\mathbf{J}}_{\text{y}} = {\mathbf{B}}_{\text{yy}} - {\mathbf{B}}_{\text{yx}} {\mathbf{B}}_{\text{xx}}^{ - 1} {\mathbf{B}}_{\text{xy}} , \\ & {\mathbf{Q}} = \left( {\begin{array}{cc} {\mathbf{P}} & \quad{{\mathbf{PB}}_{\text{xx}}^{ - 1} {\mathbf{B}}_{\text{xy}} } \\ 0 &\quad {{\mathbf{I}}_{\text{y}} } \\ \end{array} } \right), \\ & \left( {\begin{array}{cc} {{\mathbf{B}}_{\text{xx}} } &\quad {{\mathbf{B}}_{\text{xy}} } \\ {{\mathbf{B}}_{\text{yx}} } & \quad{{\mathbf{B}}_{\text{yy}} } \\ \end{array} } \right) = {\mathbf{B}}, \\ \end{aligned} $$

*where*${\mathbf{I}}_{\text{y}}$*denotes the*$K \times K$*identity matrix,*${\mathbf{B}}$*is defined in Eq.* (4.3b)*, and*${\mathbf{P}}$*is defined in Eq.* (E.24) *in Appendix*E.

Note that it is arbitrary which phenotypes we choose as having small initial population densities. Thus, whether each approximate phenotype’s initial population density is small or not can be decided in such a manner that satisfying the approximability condition becomes easiest. If none of the approximate population densities is treated as small, i.e., $K = 0$, Theorem 3 becomes identical to Theorem 2. Thus, Theorem 3 includes Theorem 2 as a special case. The threshold phenotypic distance $\varepsilon$ and the way of clustering can also be chosen arbitrarily, so that satisfying the approximability condition becomes easiest, as long as $\varepsilon > \varepsilon_{\mu }$. Notice that $\tilde{C}_{{\mathbf{h}}}$ depends on $\varepsilon$, although it is bounded when $\varepsilon \to 0$. In addition, all of the other mathematical objects in Eq. (5.8a) depend indirectly on $\varepsilon$, because $\varepsilon$ affects how to cluster the existing phenotypes. A procedure for the evaluation of Eq. (5.8a) would be as follows: first choose $\varepsilon$, and then choose approximate phenotypes, choose $K$, choose $d$ when $K > 0$ (so that the right-hand side of Eq. (5.8a) is maximized), and examine whether the inequality holds good.

Moreover, the initial state $$ {\mathbf{\overset{\hbox{$\smash{\scriptscriptstyle\frown}$}}{n^{{\prime }}} }} $$ need not be exactly at an equilibrium of the resident phenotypes $$ s_{1} , \ldots ,s_{N} $$ if the value of $$ \overset{\lower0.5em\hbox{$\smash{\scriptscriptstyle\frown}$}}{C_{\text{Fz}}^{{\prime }}} $$ is adjusted such that $$ F(s_{i} ;{\mathbf{s}}^{{\prime }} ;{{\overset{\lower0.5em\hbox{$\smash{\scriptscriptstyle\frown}$}}{{\mathbf{n}}^{{\prime }}} }} ) \le \varepsilon \overset{\lower0.5em\hbox{$\smash{\scriptscriptstyle\frown}$}}{C_{\text{Fz}}^{{\prime }} }$$ is satisfied for all $$ i = 1, \ldots ,N + 1 $$. Therefore, this theorem can be applied also to the case of higher mutation rates, in which frequent mutant invasions prevent the community from reaching the next population-dynamical equilibrium.

Note also that the smallness of changes of the population densities of the approximate phenotypes ensures not only the smallness of fitness changes of existing phenotypes $F(s_{i} ;{\mathbf{s}}^{{\prime }} ;{\mathbf{n}}^{{\prime }} )$ for $i = 1, \ldots ,N + 1$, i.e., LV-approximability, but also the smallness of fitness changes of any non-existing phenotype $z$, i.e., of the fitness landscape $F(z;{\mathbf{s}}^{{\prime }} ;{\mathbf{n}}^{{\prime }} )$. Specifically, from Theorem 3 we immediately see

#### **Corollary 1**

*If the approximability condition, Eq.* (5.8a)*, in Theorem*3*is satisfied, then the change of the fitness landscape*$F(z;{\mathbf{s}}^{{\prime }} ;{\mathbf{n}}^{{\prime }} )$*is slight during the transient following mutant invasion, because by using Taylor’s theorem we see that*

5.10a $$ \begin{aligned} & \left| {F(z;{\mathbf{s}}^{{\prime }} ;{\mathbf{n}}^{{\prime }} ) - F(z;{\mathbf{s}}^{{\prime }} ;{\mathbf{\overset{\lower0.5em\hbox{$\smash{\scriptscriptstyle\frown}$}}{n^{{\prime }}} }} )} \right| \\ & \quad = \left| {\overset{\lower0.5em\hbox{$\smash{\scriptscriptstyle\smile}$}}{F} (z;{\mathbf{s}}^{{\prime }} ;{\mathbf{m}}^{{\prime }} ) - \overset{\lower0.5em\hbox{$\smash{\scriptscriptstyle\smile}$}}{F} (z;{\mathbf{s}}^{{\prime }} ;{\mathbf{\overset{\lower0.5em\hbox{$\smash{\scriptscriptstyle\frown}$}}{m^{{\prime }}} }} )} \right| \\ & \quad = \left| {\left[ {\overset{\lower0.5em\hbox{$\smash{\scriptscriptstyle\smile}$}}{F} (z;{\mathbf{s}}^{{\prime }} ;{\mathbf{\overset{\lower0.5em\hbox{$\smash{\scriptscriptstyle\frown}$}}{m^{{\prime }}} }} ) + \left. {\frac{{\partial \overset{\lower0.5em\hbox{$\smash{\scriptscriptstyle\smile}$}}{F} (z;{\mathbf{s}}^{{\prime }} ;{\mathbf{m}}^{{\prime }} )}}{{\partial {\mathbf{m}}^{{\prime }} }}} \right|_{{{\mathbf{m}}^{{\prime }} = {\mathbf{m}}_{\text{T}}^{{\prime }} }} ({\mathbf{m}}^{{\prime }} - {{\overset{\lower0.5em\hbox{$\smash{\scriptscriptstyle\frown}$}}{{\mathbf{m}}^{{\prime }}} }} )} \right] - \overset{\lower0.5em\hbox{$\smash{\scriptscriptstyle\smile}$}}{F} (z;{\mathbf{s}}^{{\prime }} ;{\mathbf{\overset{\lower0.5em\hbox{$\smash{\scriptscriptstyle\frown}$}}{m^{{\prime }}} }} )} \right|\, \\ & \quad = \left| {\left. {\frac{{\partial \overset{\lower0.5em\hbox{$\smash{\scriptscriptstyle\smile}$}}{F} (z;{\mathbf{s}}^{{\prime }} ;{\mathbf{m}}^{{\prime }} )}}{{\partial {\mathbf{m}}^{{\prime }} }}} \right|_{{{\mathbf{m}}^{{\prime }} = {\mathbf{m}}_{\text{T}}^{{\prime }} }} ({\mathbf{m}}^{{\prime }} - {\mathbf{\overset{\lower0.5em\hbox{$\smash{\scriptscriptstyle\frown}$}}{m^{{\prime }}} }} )} \right| \\ & \quad \le \mathop {\hbox{max} }\limits_{{\left| {{\mathbf{m}}^{{\prime }} - {\mathbf{\overset{\lower0.5em\hbox{$\smash{\scriptscriptstyle\frown}$}}{m^{{\prime }}} }} } \right| < C_{{\mathbf{m}}}^{{\prime }} \varepsilon }} {\left\| {\frac{{\partial \overset{\lower0.5em\hbox{$\smash{\scriptscriptstyle\smile}$}}{F} (z;{\mathbf{s}}^{{\prime }} ;{\mathbf{m}}^{{\prime }} )}}{{\partial {\mathbf{m}}^{{\prime }} }}} \right\|} C_{{\mathbf{m}}}^{{\prime }} \varepsilon , \\ \end{aligned} $$

*where*$$ {\mathbf{m}}_{\text{T}} : = \theta_{\text{T}} ({\mathbf{m}} - {{\overset{\lower0.5em\hbox{$\smash{\scriptscriptstyle\frown}$}}{{\mathbf{m}}} }}) + {\mathbf{\overset{\lower0.5em\hbox{$\smash{\scriptscriptstyle\frown}$}}{m} }} $$*with an appropriately chosen*$$ \theta_{\text{T}} \in [0,1] $$*, and by using*$$ C_{{\mathbf{m}}} $$*from Eq.* (5.9a) *in*$$ C^{\prime}_{{\mathbf{m}}} = \sqrt {C_{\text{m}}^{ 2} + (N + 1 - M)\eta^{2} } $$*from Lemma* 2*, we see that*

5.10b $$ C_{{\mathbf{m}}}^{{\prime }} = \sqrt {\left( {\frac{{2\alpha \left\| {{\mathbf{Q}}^{ - 1} } \right\|\hbox{max} \left\{ {\tilde{C}_{{\mathbf{h}}} , - \tfrac{1}{2}\tilde{\lambda }_{\rm max} \rho_{\text{m}} } \right\}}}{{\left| {\tilde{\lambda }_{\rm max} } \right|}} + \rho_{\text{m}} } \right)^{2} + (N + 1 - M)\eta^{2} } . $$

### Generalization to higher-dimensional trait spaces

Theorems 1–3 and Corollary 1 apply to one-dimensional trait spaces. These results readily generalized to trait spaces of arbitrary dimensions by a slight modification of the analyses so that the derivative of the fitness function with respect to a phenotype in the one-dimensional trait space is replaced with the corresponding directional derivative in the higher-dimensional trait space, as shown in Appendix I.

## Tighter estimates

For deriving the approximability conditions in Eqs. (4.7a) and (5.8a), we have used the maximum possible values of the perturbation terms ($$ {\mathbf{h}} $$ and $$ {\tilde{\mathbf{h}}} $$) and nonlinear terms ($$ {\mathbf{r}} $$ and $$ {\tilde{\mathbf{r}}} $$) attainable for $$ {\mathbf{n}}^{{\prime }} \in [0,\eta ]^{N + 1} $$. These provide the simplest, but rather conservative, approximability conditions. By approximating those terms as linear or higher-order functions of population densities (corresponding to $$ {\mathbf{x}} $$ and $$ {\mathbf{w}} $$), we may improve the estimates underlying the approximability conditions. In Theorem 2, for example, the perturbation term $$ {\mathbf{h}} $$ can be expanded in $$ {\mathbf{w}} $$ around $$ {\mathbf{w}} = {\mathbf{0}} $$ (i.e., $$ {\mathbf{m}} = {\mathbf{\overset{\lower0.5em\hbox{$\smash{\scriptscriptstyle\frown}$}}{m} }} $$) up to the first-order remainder terms,

6.1 $$ \begin{aligned}   {\mathbf{h}} &  = {\mathbf{\overset{\lower0.5em\hbox{$\smash{\scriptscriptstyle\frown}$}}{h} }} + {\mathbf{Hx}}, \\    {\mathbf{\overset{\lower0.5em\hbox{$\smash{\scriptscriptstyle\frown}$}}{h} }} &  = \left. {\mathbf{h}} \right|_{{{\mathbf{m}} = {\mathbf{\overset{\lower0.5em\hbox{$\smash{\scriptscriptstyle\frown}$}}{m^{\prime } } }}}} , \\    {\mathbf{H}} &  = \left. {\frac{{\partial {\mathbf{h}}}}{{\partial {\mathbf{x}}}}} \right|_{{{\mathbf{m}} = {\mathbf{m}}_{{\text{T}}}^{\prime } }}  \\  \end{aligned},  $$

with some appropriately chosen ${\mathbf{m}}_{\text{T}} \in [0,\eta ]^{M}$. Then, applying Eqs. (6.1) to Lemma 6 gives the condition

6.2 $$ \sqrt \varepsilon < \frac{{ - \lambda_{\rm max} }}{{\sqrt {4\overset{\lower0.5em\hbox{$\smash{\scriptscriptstyle\frown}$}}{C}_{{\mathbf{h}}} C_{{\mathbf{r}}} + 2C_{{\mathbf{H}}} \big| {\lambda_{\rm max} } \big|} }}, $$

where $$ \overset{\lower0.5em\hbox{$\smash{\scriptscriptstyle\frown}$}}{C}_{{\mathbf{h}}} \ge \left| {{\mathbf{\overset{\lower0.5em\hbox{$\smash{\scriptscriptstyle\frown}$}}{h} }}} \right| $$ and $$ C_{{\mathbf{H}}} \ge \left\| {\mathbf{H}} \right\| $$; see Appendix J for the derivation. As the magnitude of the zeroth-order term for $$ {\mathbf{h}} $$ is estimated more tightly by $$ \overset{\lower0.5em\hbox{$\smash{\scriptscriptstyle\frown}$}}{C}_{{\mathbf{h}}} $$ at $$ {\mathbf{m}} = {\mathbf{\overset{\hbox{$\smash{\scriptscriptstyle\frown}$}}{m} }} $$, compared to $$ C_{{\mathbf{h}}} $$ used in the original approximability condition in Eq. (4.8a), this condition can work better than the original approximability condition, but is less simple.

## Example: Approximability condition for a resource-competition model

In this section, we give a simple example of how to examine the approximability condition in a specific ecological model.

### Model description

We consider a resource-competition model based on the Beddington–DeAngelis-type functional response (Beddington 1975; DeAngelis et al. 1975), known to describe both saturation of consumption and interference competition among consumers. Under $N$ coexisting consumer phenotypes ${\mathbf{s}} = (s_{1} , \ldots ,s_{N} )^{\text{T}}$ with their densities ${\mathbf{n}} = (n_{1} , \ldots ,n_{N} )^{\text{T}}$, we describe the $i$th phenotype’s per-capita growth rate as

7.1 $$ \frac{1}{{n_{i} }}\frac{{{\text{d}}n_{i} }}{{{\text{d}}t}} = \beta g(s_{i} ;{\mathbf{s}};{\mathbf{n}}) - \psi , $$

7.2 $$ g(s_{i} ;{\mathbf{s}};{\mathbf{n}}) = \frac{{\theta (s_{i} )}}{{\zeta_{1} + \zeta_{2} \theta (s_{i} ) + \zeta_{3} \sum\nolimits_{j = 1}^{N} {n_{j} \alpha (s_{j} ,s_{i} )} }} , $$

In Eq. (7.1), $g(s_{i} ;{\mathbf{s}};{\mathbf{n}})$ is the resource gain of phenotype $s_{i}$, $\beta$ is a constant assimilation efficiency, and $\psi$ is a constant natural death rate. In Eq. (7.2), $\theta (s_{i} )$ is the density of potential resources for $s_{i}$, and $\alpha (s_{j} ,s_{i} )$ describes the niche overlap between phenotypes $s_{i}$ and $s_{j}$. $\zeta_{1}$, $\zeta_{2}$, and $\zeta_{3}$ are constant parameters related to the encounter rate of resources, handling time of resources, and intensity of interference competition, respectively. Notice that $\zeta_{3} = 0$ gives the Holling type-II functional response (Holling 1959). Equation (7.2) can be derived from a generalized Beddington–deAngelis functional response with explicit description of a resource distribution and phenotypes’ niches expressed along a resource-quality axis (Appendix L.1).

We assume $\psi = 1$ and $\zeta_{3} = 1$ without loss of generality. For simplicity, we assume that $\theta (s_{i} )$ depends only on $s_{i}$ (i.e., constant inflows of resources into the system) and that

7.3 $$ \alpha (s_{j} ,s_{i} ) = \exp \left( { - \tfrac{1}{2}(s_{j} - s_{i} )^{2} } \right).$$

### Approximability condition

As a simplest example for the approximability condition in this model, we consider invasion by a mutant phenotype $$ s_{2} $$ into a single resident phenotype $$ s_{1} $$ at its population-dynamical equilibrium $$ \overset{\lower0.5em\hbox{$\smash{\scriptscriptstyle\frown}$}}{n}_{1} = [\beta - \zeta_{2} ]\theta (s_{1} ) - \zeta_{1} $$. From Eqs. (7.1) and (7.2), their population dynamics are given by

7.4a $$ \frac{1}{{n_{1} }}\frac{{{\text{d}}n_{1} }}{{{\text{d}}t}} = F(s_{1} ;{\mathbf{s}}^{{\prime }} ;{\mathbf{n}}^{{\prime }} ) = \frac{{\beta \theta (s_{1} )}}{{\zeta_{1} + \zeta_{2} \theta (s_{1} ) + n_{1} + n_{2} \alpha (s_{2} ,s_{1} )}} - 1 , $$

7.4b $$ \frac{1}{{n_{2} }}\frac{{{\text{d}}n_{2} }}{{{\text{d}}t}} = F(s_{2} ;{\mathbf{s}}^{{\prime }} ;{\mathbf{n}}^{{\prime }} ) = \frac{{\beta \theta (s_{2} )}}{{\zeta_{1} + \zeta_{2} \theta (s_{2} ) + n_{1} \alpha (s_{2} ,s_{1} ) + n_{2} }} - 1 , $$

with $$ {\mathbf{s}}^{{\prime }} = (s_{1} ,s_{2} )^{\text{T}} $$ and $$ {\mathbf{n}}^{{\prime }} = (n_{1} ,n_{2} )^{\text{T}} . $$

We choose $s_{1}$ as the approximate phenotype, i.e., $s_{\text{a}} = s_{1}$ and ${\mathbf{m}}^{{\prime }} = (m_{1} ,m_{2} )^{\text{T}} = (n_{1} + n_{2} ,\varepsilon n_{2} )^{\text{T}}$ with $\varepsilon = s_{2} - s_{1}$, $0 < \varepsilon \ll 1$. Then, from Eqs. (4.3b) and (4.5b), we see

7.5 $$ \begin{aligned} \lambda_{\rm max} & = \overset{\lower0.5em\hbox{$\smash{\scriptscriptstyle\frown}$}}{m}_{1} b_{11} = \overset{\lower0.5em\hbox{$\smash{\scriptscriptstyle\frown}$}}{n}_{1} \left[ {\frac{{{\text{d}}F(s_{2} ;s_{1} ;m_{1} )}}{{{\text{d}}m_{1} }}} \right]_{{m_{1} = \overset{\lower0.5em\hbox{$\smash{\scriptscriptstyle\frown}$}}{n}_{1} }} \\ & = - \frac{{\overset{\lower0.5em\hbox{$\smash{\scriptscriptstyle\frown}$}}{n}_{1} }}{{\beta \theta (s_{1} )}} \\ & = - \frac{{[\beta - \zeta_{2} ]\theta (s_{1} ) - \zeta_{1} }}{{\beta \theta (s_{1} )}}. \\ \end{aligned} $$

Notice that $$ \lambda_{\rm max} $$ is always negative because $$ \overset{\lower0.5em\hbox{$\smash{\scriptscriptstyle\frown}$}}{n}_{1} $$ must be positive. As for $$ C_{{\mathbf{h}}} $$ and $$ C_{{\mathbf{r}}} $$ in the approximability condition $$ \sqrt \varepsilon < - \lambda_{\rm max} /[2\sqrt {C_{{\mathbf{h}}} C_{{\mathbf{r}}} } ] $$ in Theorem 2, we find (as derived in Appendix L.2) that

7.6 $$ \begin{aligned} C_{{\mathbf{h}}} & \le \overset{\lower0.5em\hbox{$\smash{\scriptscriptstyle\smile}$}}{C}_{{\mathbf{h}}} , \\ \overset{\lower0.5em\hbox{$\smash{\scriptscriptstyle\smile}$}}{C}_{{\mathbf{h}}} & = \frac{{\eta \beta [5C_{\partial \theta } + 3\varepsilon C_{\theta } ]}}{{\zeta_{1} + \zeta_{2} C_{\theta {\rm min} } }}, \\ C_{{\mathbf{r}}} & = \frac{1}{{\beta \theta (s_{1} )}} + \eta \frac{{2\beta \theta (s_{1} )}}{{[\zeta_{1} + \zeta_{2} \theta (s_{1} )]^{3} }}, \\ \end{aligned} $$

with

7.7 $$ \begin{aligned} \eta & = [\beta - \zeta_{2} ]C_{\theta } - \zeta_{1} , \\ C_{\theta } & = \hbox{max} \left\{ {\theta (s)\left| {s \in [s_{1} ,s_{2} ]} \right.} \right\}, \\ C_{\theta {\rm min} } & = \hbox{min} \left\{ {\theta (s)\left| {s \in [s_{1} ,s_{2} ]} \right.} \right\}, \\ C_{\partial \theta } & = \hbox{max} \left\{ {\frac{{{\text{d}}\theta (s)}}{{{\text{d}}s}}\big| {s \in [s_{1} ,s_{2} ]} } \right\}. \\ \end{aligned} $$

Therefore, a sufficient condition for the approximability condition is given by

7.8 $$ \begin{aligned} \sqrt \varepsilon & < - \frac{{\lambda_{\rm max} }}{{2\sqrt {\overset{\lower0.5em\hbox{$\smash{\scriptscriptstyle\smile}$}}{C}_{{\mathbf{h}}} C_{{\mathbf{r}}} } }} \\ & = \frac{{\overset{\lower0.5em\hbox{$\smash{\scriptscriptstyle\frown}$}}{n}_{1} }}{{2\sqrt {\frac{{\eta \beta [5C_{\partial \theta } + 3\varepsilon C_{\theta } ]}}{{\zeta_{1} + \zeta_{2} C_{\theta {\rm min} } }}\left( {\beta \theta (s_{1} ) + \eta \frac{{2\beta^{3} \theta (s_{1} )^{3} }}{{[\zeta_{1} + \zeta_{2} \theta (s_{1} )]^{3} }}} \right)} }} \\ \end{aligned} $$

with $$ \overset{\lower0.5em\hbox{$\smash{\scriptscriptstyle\frown}$}}{n}_{1} = [\beta - \zeta_{2} ]\theta (s_{1} ) - \zeta_{1} $$ and Eqs. (7.7). Notice that the right-hand side of Eq. (7.8) includes $$ \varepsilon C_{\theta } $$, which is negligible when $$ \varepsilon C_{\theta } \ll C_{\partial \theta } $$.

## Application: Extending the invasion–implies–substitution theorem

The derived stability conditions and resultant Lotka–Volterra approximation can be used to analyze the community dynamics triggered by a mutant invasion. In this section, we apply them to extend the invasion–implies–substitution theorem (Dercole and Rinaldi 2008, Appendix B) to an arbitrary set of resident phenotypes that form well-recognizable and -separated clusters in a one-dimensional trait space; see Appendix K for details.

We assume an arbitrary set of resident phenotypes $s_{1} , \ldots ,s_{N}$ together with a mutant $s^{{\prime }} = s_{N + 1}$, with the resident and mutant phenotypes clustered into approximate phenotypes ${\mathbf{s}}_{\text{a}} = (s_{1} , \ldots ,s_{M} )^{\text{T}}$ that satisfy the approximability condition of Theorem 3. Then, from Lemma 2, $\left| {\Delta {\mathbf{m}}^{{\prime }} } \right| \le \varepsilon C_{\text{m}}^{{\prime }} = \varepsilon \sqrt {C_{\text{m}}^{ 2} + (N + 1 - M)\eta^{2} }$ is conserved during the transient following mutant invasion. We denote the identity of the cluster containing the mutant by $i$, i.e., ${\text{cid}}(N + 1) = i$. Using Eq. (4.1a), the dynamics of the mutant fraction $p_{N + 1} = n_{N + 1} /m_{i}$ within this cluster can be expressed as

8.1 $$ \begin{aligned} \frac{{{\text{d}}p_{N + 1} }}{{{\text{d}}t}} & = \frac{\text{d}}{{{\text{d}}t}}\frac{{n_{N + 1} }}{{m_{i} }} \\ & = \frac{{n_{N + 1} }}{{m_{i} }}F(s_{N + 1} ;{\mathbf{s}}^{{\prime }} ;{\mathbf{n}}^{{\prime }} ) - \frac{{n_{N + 1} }}{{m_{i} }}f(s_{i} ;{\mathbf{s}}^{{\prime }} ;{\mathbf{n}}^{{\prime }} ) \\ & = p_{N + 1} F(s_{N + 1} ;{\mathbf{s}}^{{\prime }} ;{\mathbf{n}}^{{\prime }} ) - p_{N + 1} \sum\limits_{{j \in {\text{com}}(i)}} {p_{j} F(s_{j} ;{\mathbf{s}}^{{\prime }} ;{\mathbf{n}}^{{\prime }} )} . \\ \end{aligned} $$

For convenience, we assume that the representative phenotype $s_{i}$ of this cluster is chosen as the phenotype most similar to the mutant, i.e., $\left| {s_{N + 1} - s_{i} } \right| = \mathop {\hbox{min} }\nolimits_{{j \in {\text{com}}(i)}} \left| {s_{N + 1} - s_{j} } \right|$. Then, by Taylor’s theorem, we transform Eq. (8.1) into

8.2a $$ \frac{{{\text{d}}p_{N + 1} }}{{{\text{d}}t}} \ge p_{N + 1} (1 - p_{N + 1} )\left[ {\overset{\lower0.5em\hbox{$\smash{\scriptscriptstyle\frown}$}}{F}_{\text{z}} (s_{i} )(s_{N + 1} - s_{i} ) - \varepsilon C_{{{\text{Fz}}{\mathbf{m}}}}^{{\prime }} \left| {\Delta {\mathbf{m}}^{{\prime }} } \right| - \varepsilon^{2} C_{\text{Fzz}} } \right] . $$

Here, $$ \overset{\lower0.5em\hbox{$\smash{\scriptscriptstyle\frown}$}}{F}_{\text{z}} (s_{i} ) = \left. {\partial F(z;{\mathbf{s}}^{{\prime }} ;{\mathbf{\overset{\lower0.5em\hbox{$\smash{\scriptscriptstyle\frown}$}}{n^{{\prime }}} }} ) /\partial z} \right|_{{z = s_{i} }} $$ is the fitness gradient at $$ s_{i} $$, and the constants $$ C_{{{\text{Fz}}{\mathbf{m}}}}^{{\prime }} $$ and $$ C_{\text{Fzz}}^{{\prime }} $$ bound the remainder terms through

8.2b $$ \left| {\frac{{\partial \overset{\lower0.5em\hbox{$\smash{\scriptscriptstyle\smile}$}}{F} (z;{\mathbf{s}}^{{\prime }} ;{\mathbf{m}}^{{\prime }} )}}{{\partial {\mathbf{m}}^{{\prime }} \partial z}}} \right|_{{z = z_{{j{\text{T}}}} }} \le C_{{{\text{Fz}}{\mathbf{m}}}}^{{\prime }} ,\quad \left| {\frac{{\partial^{2} F(z;{\mathbf{s}}^{{\prime }} ;{\mathbf{n}}^{{\prime }} )}}{{\partial z^{2} }}} \right|_{{z = z_{{j{\text{T}}}} }} \le C_{\text{Fzz}}^{{\prime }} , $$

for $z_{{j{\text{T}}}} \in [s_{j} ,s_{{{\text{cid}}(j)}} ]$ for all $j = 1, \ldots ,N + 1$ during the transient following mutant invasion. Then, by substituting our results $\left| {\Delta {\mathbf{m}}^{{\prime }} } \right| \le \varepsilon C_{{\mathbf{m}}}^{{\prime }} = \varepsilon \sqrt {C_{\text{m}}^{ 2} + (N + 1 - M)\eta^{2} }$ and Eq. (5.9a) into Eq. (8.2a), a sufficient condition for ${\text{d}}p_{N + 1} / {\text{d}}t$ to be always positive is given by

8.3 $$ \overset{\lower0.5em\hbox{$\smash{\scriptscriptstyle\frown}$}}{F}_{\text{z}} (s_{i} )(s_{N + 1} - s_{i} ) > \varepsilon^{2} [C_{\text{Fzz}} + C_{{{\text{Fz}}{\mathbf{m}}}}^{{\prime }} C_{{\mathbf{m}}}^{{\prime }} ] $$

with $C_{{\mathbf{m}}}^{{\prime }}$ in Eq. (5.10b).

If the fitness gradient $$ \overset{\lower0.5em\hbox{$\smash{\scriptscriptstyle\frown}$}}{F}_{\text{z}} (s_{i} ) $$ is sufficiently strong, so that it satisfies Eq. (8.3), then $$ p_{N + 1} $$ monotonically increases until it reaches 1, i.e., until all other phenotypes within the cluster containing the mutant are excluded. Equation (8.3) means that the fitness advantage of $$ s_{N + 1} $$ against $$ s_{i} $$ due to the fitness gradient must exceed the effects of the curvature of the fitness landscape ($$ \varepsilon^{2} C_{\text{Fzz}} $$) and of the perturbation due to the population dynamics ($$ \varepsilon^{2} C_{{{\text{Fz}}{\mathbf{m}}}}^{{\prime }} C_{{\mathbf{m}}}^{{\prime }} $$). As long as Eq. (8.3) holds for any resident phenotype and its mutants, repeated mutant invasions always result in monomorphic phenotype clusters, i.e., resident phenotypes are kept dissimilar, corresponding to the situation considered by Dercole and Rinaldi (2008). Notice that when $$ \left| {\tilde{\lambda }_{\rm max} } \right| $$ becomes close to zero, e.g., when the community is close to a bifurcation point of its population dynamics, $$ C_{{\mathbf{m}}}^{{\prime }} $$ becomes large and Eq. (8.3) thus becomes difficult to satisfy.

## Discussion

As explained in the beginning of this paper, ecological interactions engender various evolutionary dynamics, including cyclic coevolution, adaptive radiation, adaptive speciation, taxon cycles, and community formation. To analyze how ecological interactions induce selection pressures that drive such dynamics, the following two assumptions are often made (Metz et al. 1992, 1996; Dieckmann and Law 1996). First, mutation rates are sufficiently small relative to the timescale of the population dynamics, so that the evolutionary dynamics are reduced to trait-substitution sequences resulting from repeated mutant invasions. Second, mutational step sizes are sufficiently small, so that a mutant invasion typically results in an equilibrium phenotype distribution similar to that before the invasion. The latter is called attractor inheritance (Geritz et al. 2002). In such cases, each mutant invasion modifies the fitness landscape only slightly. The fitness landscape can then be treated as a smooth function of resident phenotypes at equilibrium population densities, enabling effective analyses of directional coevolution (Dieckmann and Law 1996) and diversification through evolutionary branching (Metz et al. 1992, 1996; Geritz et al. 1997, 1998). Using the concept of approximate phenotypes introduced in the present paper, attractor inheritance can be translated into the smallness of changes of the population densities of approximate phenotypes during the transient population dynamics following mutant invasion, toward the next population-dynamical equilibrium.

### Conditions for attractor inheritance

Prior to our analyses in the present paper, qualitative conditions for attractor inheritance have been proved for sufficiently small mutational step sizes in the following two cases: (1) all residents and the mutant are similar to each other (Geritz et al. 2002; Meszéna et al. 2005; Durinx et al. 2008), or (2) no two residents are similar to each other and their initial equilibrium population densities are not small (Dercole and Rinaldi 2008, Appendix B). In this paper, we have derived quantitative conditions for attractor inheritance for a set of residents and a mutant, by clustering them according to a threshold phenotypic distance into approximate phenotypes. The conditions ensuring attractor inheritance, i.e., the approximability conditions in Theorems 2 and 3, establish relationships among the magnitudes of the mutational step size, the return rate to an equilibrium of the population dynamics of approximate phenotypes, the nonlinearity of the population dynamics, and the perturbation due to within-cluster population dynamics. These conditions are especially important when finite, rather than infinitesimally small, mutational step sizes are required for analyzing the considered evolutionary dynamics, such as when investigating evolutionary suicide (Gyllenberg and Parvinen 2001) and evolutionary branching of directionally evolving populations (Ito and Dieckmann 2012, 2014). A next step would be to analyze whether it is really possible to satisfy the approximability condition, or rather, whether the condition can be satisfied with not too large error bounds in all but a set of theoretically possible but practically irrelevant cases. Although we here have considered only deterministic population dynamics, the impact of demographic stochasticity on trait-substitution sequences (Geritz et al. 2002) can be considered using the same framework we have introduced here, by subsuming its effect in the perturbation terms.

### Assumption of well-recognizable and -separated phenotypic clusters

Our analysis assumes that the number $N$ of existing phenotypes is finite, and that phenotypic clusters are well-recognizable and well-separated from each other so that the largest of within-cluster distances, $\varepsilon$, is much smaller than the smallest of between-cluster distances. We discuss the validity of our two assumptions below.

In principle, ODE population models should be seen as large-system-size limits of stochastic individual-based models. Generally, the larger the number of coexisting phenotypes, the slower is the convergence to the ODE limit. Thus, for all practical purposes, ODE models with very large numbers of phenotypes can be left out of the picture. If we do so, the finiteness of the number of existing phenotypes, $N$, ensures the existence of the smallest between-cluster distance and the largest within-cluster distance.

However, if a system has long chains of phenotypes in which the distances between any two consecutive members of the chain are small but the distance between the ends of the chain is large, we have no way to cluster them so that $\varepsilon$ becomes much smaller than the smallest between-cluster distance. In this case, the error estimate for perturbation terms in Theorems 2 or 3 ($C_{{\mathbf{h}}}$ or $\tilde{C}_{{\mathbf{h}}}$), in comparison with the leading eigenvalue of the community Jacobian matrix ($\lambda_{\rm max}$ or $\tilde{\lambda }_{\rm max}$), can be too large for the approximability condition to be satisfied.

Fortunately, there is effectively no chance of such configurations occurring in ongoing evolutionary dynamics with sufficiently small mutational step sizes as in such dynamics closely similar phenotypes only occur in the early stages of evolutionary branching. (The local coexistence regions that can occur in higher-dimensional trait spaces around the zero fitness contour for particular residents are that narrow that the chance of a mutant landing in them is practically negligible. The more so since the far more common mutants landing outside these coexistence regions will oust all those inside the regions, so there is no chance of the number of coexisting similar phenotypes ever becoming large (Durinx et al. 2008).) Finally, of phenotypes that evolve towards each other, only one will survive due to competitive exclusion. Therefore, the assumption of well-recognizable and -separated phenotypic clusters is warranted except for a fraction of cases that will be encountered only very exceptionally, as well as transiently, in the scenarios that have our interest.

### LV-approximation for analyzing evolutionary branching in multidimensional trait spaces

As shown in Sect. 3, attractor inheritance in approximate phenotypes directly enables LV-approximations of the population dynamics of the original phenotypes before clustering, similar to the previous studies (Meszéna et al. 2005; Dercole and Rinaldi 2008; Durinx et al. 2008). The derived LV-approximations may be especially useful for extending conditions for evolutionary branching from one-dimensional trait spaces to higher-dimensional trait spaces. In two-dimensional trait spaces, various numerical analyses have shown that phenotypes that are strongly convergence stable, but not evolutionarily stable, also known as strongly attracting invadable ESSes, induce evolutionary branching (e.g., Vukics et al. 2003; Ackermann and Doebeli 2004; Egas et al. 2005; Ravigné et al. 2009; Ito and Dieckmann 2012). Those phenotypes are fixed-point attractors that can be attained by directional evolution causing the convergence of a monomorphic population (Leimar 2009) to them, with sufficient proximity of a set in $\mathcal{\mathcal{S}}^{2}$ enabling the emergence of dimorphisms followed by directional evolution causing the divergence of the two morphs. However, whether an emergent polymorphism can evolutionarily diversify further into visually distinct morphs without collapse has not been proved until recently for higher-dimensional trait spaces. Based on the rational form of invasion-fitness functions in terms of existing phenotypes, which has been derived by LV-approximation (Durinx et al. 2008), Geritz et al. (2016) derived a set of conditions that ensure that such diversifying evolution does not collapse in trait spaces of arbitrary dimension, by describing the initial diversifying evolution with coupled Lande equations (Lande 1979). While those conditions are satisfied by strongly attracting invadable ESSes in two-dimensional trait spaces, the higher-dimensional cases remain to be analyzed further (Geritz et al. 2016).

### Axioms for fitness functions

The analyses in this paper are based on a set of axioms for the fitness-generating functions characterizing ecologically plausible differential equations describing trait-mediated community dynamics. Our set of axioms are similar to the set of properties assumed in Dercole (2016), which are used by him to derive a general procedure for formulating population-dynamical models resulting from individual pairwise interaction. Properties 1, 2, and 3 in Dercole (2016) are identical to our axiom (iii), (iv), and (ii), respectively, while property 4 in Dercole (2016) corresponds to our axiom (i). Dercole’s property 4, however, delimits a smaller class of models than ours.

The symmetry axiom (ii) and the reducibility axiom (iii) are no more than consistency conditions, as is the exchangeability axiom (iv). The latter axiom, however, is together with the remaining smoothness axiom (i) and bounded-world axiom (v) the root cause of the Lotka–Volterra approximabiliy. Indeed, Lemma 1, which is central for deriving the condition for attractor inheritance and LV-approximation, is proved by applying those three axioms (Appendix A). While the bounded-world axiom (v) seems to be well grounded in reality, the smoothness axiom (i) may not hold in an exact sense, because it assumes that the population-dynamical behavior of individuals depends smoothly on their traits and that all ecological interactions are instantaneous. This instantaneousness can arise when the timescale of the life-history dynamics among individuals is much faster than that of their population dynamics. Durinx et al. (2008) have proved attractor inheritance and LV-approximation in physiologically structured models with multiple birth states, in which the timescales of life-history dynamics and population dynamics are not separated. This instills us with cautious optimism that the assumption of instantaneousness we have used in the present paper might be relaxed as well.

## Appendix A: Proof of Lemma 1

Here we derive $C^{\prime}_{\text{Fm}}$ in Eq. (3.2) in the main text. We start from the observation that by the smoothness and exchangeability of $F$ and the compactness of the set of community states, i.e., axioms (i), (iv), and (v), there exists a constant $C_{\text{Fn}}^{{\prime }}$ such that

A.1 $$ \left| {\frac{{\partial F(s_{i} ;{\mathbf{s}}^{{\prime }} ;{\mathbf{n}}^{{\prime }} )}}{{\partial n_{j} }}} \right| \le C_{\text{Fn}}^{{\prime }} $$

for all *i*, *j *= 1, …, *N *+ 1, with

A.2 $$ C_{\text{Fn}}^{{\prime }} \le \hbox{max} \left\{ {\left. {\left| {\frac{{\partial F(s_{i} ;{\mathbf{s}}^{{\prime }} ;{\mathbf{n}}^{{\prime }} )}}{{\partial n_{j} }}} \right| \, } \right|\;\;i,j = 1, \ldots ,N + 1,\;{\mathbf{n}}^{{\prime }} \in [0,\eta ]^{N + 1} } \right\} . $$

The derivative of $$ \overset{\lower0.5em\hbox{$\smash{\scriptscriptstyle\smile}$}}{F} $$ with respect to $$ m_{j} $$ is calculated from

A.3 $$ \frac{{\partial \overset{\lower0.5em\hbox{$\smash{\scriptscriptstyle\smile}$}}{F} (s_{i} ;{\mathbf{s}}^{{\prime }} ;{\mathbf{m}}^{{\prime }} )}}{{\partial m_{j} }} = \sum\limits_{k = 1}^{N + 1} {\frac{{\partial n_{k} }}{{\partial m_{j} }}\frac{{\partial \overset{\lower0.5em\hbox{$\smash{\scriptscriptstyle\smile}$}}{F} (s_{i} ;{\mathbf{s}}^{{\prime }} ;{\mathbf{m}}^{{\prime }} )}}{{\partial n_{k} }}} . $$

To calculate the derivative $\frac{{\partial n_{k} }}{{\partial m_{j} }}$, we substitute Eq. (3.1b),

A.4 $$ m_{k} = \varepsilon n_{k},$$

for $i = M + 1, \ldots ,N + 1$ into Eq. (3.1a) for $i = 1, \ldots ,M,$

A.5 $$ \begin{aligned} m_{k} & = \sum\limits_{{l \in {\text{com}}(k)}} {n_{l} } \\ & = n_{k} + \sum\limits_{{l \in {\text{com}}(k),l \ne k}} {n_{l} } \\ & = n_{k} + \sum\limits_{{l \in {\text{com}}(k),l \ne k}} {\frac{1}{\varepsilon }m_{l} } , \\ \end{aligned} $$

which gives

A.6 $$ n_{k} = \left\{ {\begin{array}{*{20}l} {m_{k} - \frac{1}{\varepsilon }\sum\limits_{{l \in {\text{com}}(k),l \ne k}} {m_{l} } } \hfill & {{\text{for}}\;\;k = 1, \ldots ,M} \hfill \\ {\frac{1}{\varepsilon }m_{k} } \hfill & {{\text{for}}\;\;k = M + 1, \ldots ,N + 1.} \hfill \\ \end{array} } \right. $$

Since $l \in {\text{com}}(k)$ and $l \ne k$ require $l \in \left\{ {M + 1, \ldots ,N + 1} \right\}$, we see for $j = 1, \ldots ,M$

A.7 $$ \frac{{\partial n_{k} }}{{\partial m_{j} }} = \left\{ {\begin{array}{ll} 1 \hfill &\quad {{\text{for}}\;\;k = j} \hfill \\ 0 \hfill &\quad {{\text{otherwise,}}} \hfill \\ \end{array} } \right. $$

and for $$ j = M + 1, \ldots ,N + 1 $$

A.8 $$ \frac{{\partial n_{k} }}{{\partial m_{j} }} = \left\{ {\begin{array}{ll} { - \frac{1}{\varepsilon }} \hfill &\quad {{\text{for}}\;\;k = {\text{cid}}(j)\,\,({\text{i}} . {\text{e}} . ,\,\,j \in {\text{com}}(k))} \hfill \\ {\frac{1}{\varepsilon }} \hfill &\quad {{\text{for}}\;\;k = j} \hfill \\ 0 \hfill &\quad {{\text{otherwise}} .} \hfill \\ \end{array} } \right. $$

By substituting Eq. (A.7) into Eq. (A.3), we find for $j = 1, \ldots ,M$,

A.9 $$ \begin{aligned} \frac{{\partial \overset{\lower0.5em\hbox{$\smash{\scriptscriptstyle\smile}$}}{F} (s_{i} ;{\mathbf{s}}^{{\prime }} ;{\mathbf{m}}^{{\prime }} )}}{{\partial m_{j} }} & = \frac{{\partial F(s_{i} ;{\mathbf{s}}^{{\prime }} ;{\mathbf{n}}^{{\prime }} )}}{{\partial n_{j} }} \\ \left| {\frac{{\partial \overset{\lower0.5em\hbox{$\smash{\scriptscriptstyle\smile}$}}{F} (s_{i} ;{\mathbf{s}}^{{\prime }} ;{\mathbf{m}}^{{\prime }} )}}{{\partial m_{j} }}} \right| & = \left| {\frac{{\partial F(s_{i} ;{\mathbf{s}}^{{\prime }} ;{\mathbf{n}}^{{\prime }} )}}{{\partial n_{j} }}} \right| \le C_{\text{Fn}}^{{\prime }}. \\ \end{aligned} $$

Similarly, by substituting Eq. (A.8) into Eq. (A.3), we find for $j = M + 1, \ldots ,N + 1$

A.10 $$ \begin{aligned} \frac{{\partial \overset{\lower0.5em\hbox{$\smash{\scriptscriptstyle\smile}$}}{F} (s_{i} ;{\mathbf{s}}^{{\prime }} ;{\mathbf{m}}^{{\prime }} )}}{{\partial m_{j} }} & = \frac{{\partial n_{j} }}{{\partial m_{j} }}\frac{{\partial F(s_{i} ;{\mathbf{s}}^{{\prime }} ;{\mathbf{n}}^{{\prime }} )}}{{\partial n_{j} }} + \frac{{\partial n_{{{\text{cid}}(j)}} }}{{\partial m_{j} }}\frac{{\partial F(s_{i} ;{\mathbf{s}}^{{\prime }} ;{\mathbf{n}}^{{\prime }} )}}{{\partial n_{{{\text{cid}}(j)}} }} \\ & = \frac{1}{\varepsilon }\left[ {\frac{{\partial F(s_{i} ;{\mathbf{s}}^{{\prime }} ;{\mathbf{n}}^{{\prime }} )}}{{\partial n_{j} }} - \frac{{\partial F(s_{i} ;{\mathbf{s}}^{{\prime }} ;{\mathbf{n}}^{{\prime }} )}}{{\partial n_{{{\text{cid}}(j)}} }}} \right]. \\ \end{aligned} $$

We use Taylor’s theorem and the exchangeability axiom (iv) to derive an estimate for the term in square brackets,

A.11 $$ \begin{aligned} & \frac{{\partial F(s_{i} ;{\mathbf{s}}^{{\prime }} ;{\mathbf{n}}^{{\prime }} )}}{{\partial n_{j} }} - \frac{{\partial F(s_{i} ;{\mathbf{s}}^{{\prime }} ;{\mathbf{n}}^{{\prime }} )}}{{\partial n_{{{\text{cid}}(j)}} }} = \left[ {\frac{{\partial F(s_{i} ;{\mathbf{s}}^{{\prime }} ;{\mathbf{n}}^{{\prime }} )}}{{\partial n_{j} }} - \frac{{\partial F(s_{i} ;{\mathbf{s}}^{{\prime }} ;{\mathbf{n}}^{{\prime }} )}}{{\partial n_{{{\text{cid}}(j)}} }}} \right]_{{s_{j} = s_{{{\text{cid}}(j)}} }} \\ & \quad \quad + \left[ {\frac{{\partial^{2} F(s_{i} ;{\mathbf{s}}^{{\prime }} ;{\mathbf{n}}^{{\prime }} )}}{{\partial s_{j} \partial n_{j} }} - \frac{{\partial^{2} F(s_{i} ;{\mathbf{s}}^{{\prime }} ;{\mathbf{n}}^{{\prime }} )}}{{\partial s_{j} \partial n_{{{\text{cid}}(j)}} }}} \right]_{{s_{j} = s_{{j{\text{T}}}} }} (s_{j} - s_{{{\text{cid}}(j)}} ) \\ & \quad = \left[ {\frac{{\partial^{2} F(s_{i} ;{\mathbf{s}}^{{\prime }} ;{\mathbf{n}}^{{\prime }} )}}{{\partial s_{j} \partial n_{j} }} - \frac{{\partial^{2} F(s_{i} ;{\mathbf{s}}^{{\prime }} ;{\mathbf{n}}^{{\prime }} )}}{{\partial s_{j} \partial n_{{{\text{cid}}(j)}} }}} \right]_{{s_{j} = s_{{j{\text{T}}}} }} (s_{j} - s_{{{\text{cid}}(j)}} ) \\ & \quad = \left[ {\frac{{\partial^{2} F(s_{i} ;{\mathbf{s}}^{{\prime }} ;{\mathbf{n}}^{{\prime }} )}}{{\partial s_{j} \partial n_{j} }} - \frac{{\partial^{2} F(s_{i} ;{\mathbf{s}}^{{\prime }} ;{\mathbf{n}}^{{\prime }} )}}{{\partial s_{j} \partial n_{{{\text{cid}}(j)}} }}} \right]_{{s_{j} = s_{{j{\text{T}}}} }} \rho_{j} \varepsilon \\ \end{aligned} $$

for some appropriately chosen $s_{{j{\text{T}}}} \in [s_{j} ,s_{{{\text{cid}}(j)}} ]$, where $\rho_{j} = (s_{j} - s_{{{\text{cid}}(j)}} ) /\varepsilon$ with $\left| {\rho_{j} } \right| \le 1$ (because by assumption within-cluster phenotypic differences do not exceed $\varepsilon$). By the smoothness of $F$, there exists a constant $C_{\text{Fsn}}^{{\prime }}$ such that

A.12 $$ \begin{aligned} & \left| {\frac{{\partial^{2} F(s_{i} ;{\mathbf{s}}^{{\prime }} ;{\mathbf{n}}^{{\prime }} )}}{{\partial s_{j} \partial n_{j} }} - \frac{{\partial^{2} F(s_{i} ;{\mathbf{s}}^{{\prime }} ;{\mathbf{n}}^{{\prime }} )}}{{\partial s_{j} \partial n_{{{\text{cid}}(j)}} }}} \right|_{{s_{j} = s_{{j{\text{T}}}} }} \\ & \quad \le \hbox{max} \left\{ {\left| {\frac{{\partial^{2} F(s_{i} ;{\mathbf{s}}^{{\prime }} ;{\mathbf{n}}^{{\prime }} )}}{{\partial s_{j} \partial n_{j} }} - \frac{{\partial^{2} F(s_{i} ;{\mathbf{s}}^{{\prime }} ;{\mathbf{n}}^{{\prime }} )}}{{\partial s_{j} \partial n_{{{\text{cid}}(j)}} }}} \right|_{{s_{j} = s_{{j{\text{T}}}} }} \left| {\begin{array}{*{20}l} {i = 1, \ldots ,N + 1,j = M + 1, \ldots ,N + 1} \hfill \\ {\;\;{\mathbf{n}}^{{\prime }} \in [0,\eta ]^{N + 1} ,s_{{j{\text{T}}}} \in [s_{j} ,s_{{{\text{cid}}(j)}} ]} \hfill \\ \end{array} } \right.} \right\} \\ & \quad = :C_{\text{Fsn}}^{{\prime }} , \\ \end{aligned}$$

Thus, substituting Eqs. (A.11) and (A.12) into Eq. (A.10) yields for $j = M + 1, \ldots ,N + 1$

A.13 $$ \begin{aligned} & \frac{{\partial \overset{\lower0.5em\hbox{$\smash{\scriptscriptstyle\smile}$}}{F} (s_{i} ;{\mathbf{s}}^{{\prime }} ;{\mathbf{m}}^{{\prime }} )}}{{\partial m_{j} }} = \left[ {\frac{{\partial^{2} F(s_{i} ;{\mathbf{s}}^{{\prime }} ;{\mathbf{n}}^{{\prime }} )}}{{\partial s_{j} \partial n_{j} }} - \frac{{\partial^{2} F(s_{i} ;{\mathbf{s}}^{{\prime }} ;{\mathbf{n}}^{{\prime }} )}}{{\partial s_{j} \partial n_{{{\text{cid}}(j)}} }}} \right]_{{s_{j} = s_{{j{\text{T}}}} }} \rho_{j} , \\ & \left| {\frac{{\partial \overset{\lower0.5em\hbox{$\smash{\scriptscriptstyle\smile}$}}{F} (s_{i} ;{\mathbf{s}}^{{\prime }} ;{\mathbf{m}}^{{\prime }} )}}{{\partial m_{j} }}} \right| \le C_{\text{Fsn}}^{{\prime }} . \\ \end{aligned} $$

Finally, from Eqs. (A.9) and (A.13),

A.14 $$ \left| {\frac{{\partial \overset{\lower0.5em\hbox{$\smash{\scriptscriptstyle\smile}$}}{F} (s_{i} ;{\mathbf{s}}^{{\prime }} ;{\mathbf{m}}^{{\prime }} )}}{{\partial m_{j} }}} \right| \le \hbox{max} \left\{ {C_{\text{Fn}}^{{\prime }} ,C_{\text{Fsn}}^{{\prime }} } \right\} = :C_{\text{Fm}}^{{\prime }} . $$

□

## Appendix B: Derivation of Eq. (3.3a) and proof of Lemma 2

### B.1 Some preliminary estimates

To get an estimate for the remainder $R$ in

B.1 $$ G({\mathbf{x}} + \Delta {\mathbf{x}}) = G({\mathbf{x}}) + {\text{D}}G({\mathbf{x}})\Delta {\mathbf{x}} + R, $$

$G:{\mathbb{R}}^{N} \to {\mathbb{R}}$, consider the function $g:{\mathbb{R}} \to {\mathbb{R}}:g(z) = G({\mathbf{x}} + z\Delta {\mathbf{x}})$. Then by Taylor’s theorem,

B.2 $$ g(z) = g(0) + {\text{D}}g(0)z + \tfrac{1}{2}{\text{D}}^{2} g(\theta_{\text{T}} z)z^{2} , $$

with $\theta_{\text{T}}$ some appropriately chosen number between 0 and 1. By the chain rule,

B.3 $$  g(0) = G({\mathbf{x}}),Dg(0) = {\text{D}}G({\mathbf{x}})\Delta {\mathbf{x}},{\text{D}}^{2} g(\theta _{{\text{T}}} z) = \Delta {\mathbf{x}}^{{\text{T}}} {\text{D}}^{2} G({\mathbf{x}} + \theta _{{\text{T}}} z\Delta {\mathbf{x}})\Delta {\mathbf{x}}.  $$

Now we use that

B.4 $$ \Delta {\mathbf{x}}^{T} {\text{D}}^{2} G({\mathbf{x}} + \theta_{\text{T}} z\Delta {\mathbf{x}})\Delta {\mathbf{x}} \le \left\| {\text{D}^{2} G({\mathbf{x}} + \theta_{\text{T}} z\Delta {\mathbf{x}})} \right\|_{\text{Q}} \left| {\Delta {\mathbf{x}}} \right|^{2} $$

with $\left\| \cdot \right\|_{\text{Q}}$ defined for an arbitrary $K \times K$ symmetric matrix ${\mathbf{B}}$ by

B.5 $$ \left\| {\mathbf{B}} \right\|_{\text{Q}} : = \hbox{max} \left\{ {\left. {\left| {{\mathbf{u}}^{\text{T}} {\mathbf{Bu}}} \right| \, } \right| \, \left| {\mathbf{u}} \right| = 1} \right\}, $$

i.e., the absolute value of the dominant eigenvalue. By using Eq. (B.5), we see

B.6 $$ \left| R \right| \le \frac{1}{2}\hbox{max} \left\{ {\left. {\left\| {{\text{D}}^{2} G({\mathbf{x}} + \theta_{\text{T}} \Delta {\mathbf{x}})} \right\|_{\text{Q}} \, } \right| \, 0 \le \theta_{\text{T}} \le 1} \right\}\left| {\Delta {\mathbf{x}}} \right|^{2} . $$

When it is given that $\left| {\Delta {\mathbf{x}}} \right| < \delta$ this translates into the uniform estimates

B.7 $$ \left| R \right| < \frac{1}{2}\hbox{max} \left\{ {\left. {\left\| {{\text{D}}^{2} G({\mathbf{x}} + {\mathbf{x}}^{{\prime }} )} \right\|_{\text{Q}} \, } \right| \, \left| {{\mathbf{x}}^{{\prime }} } \right| \le \delta } \right\}\delta^{2} . $$

Note that $\left\| {\mathbf{B}} \right\|_{\text{Q}}$ satisfies

B.8 $$ \left\| {\mathbf{B}} \right\|_{\text{Q}} \le KB_{\rm max} $$

with $$ B_{\rm max} = \max_{{i,j \in \{ 1, \ldots ,K\} }} \left( {\left| {B_{ij} } \right|} \right) $$, because

B.9 $$ \begin{aligned} \left| {{\mathbf{u}}^{\text{T}} {\mathbf{Bu}}} \right| & = \left| {\sum\limits_{i = 1}^{K} {\sum\limits_{j = 1}^{K} {B_{ij} u_{i} u_{j} } } } \right| \\ & \le B_{\rm max} \sum\limits_{i = 1}^{K} {\sum\limits_{j = 1}^{K} {\left| {u_{i} } \right|\left| {u_{j} } \right|} } = B_{\rm max} \left[ {\sum\limits_{i = 1}^{K} {\left| {u_{i} } \right|^{2} } + 2\sum\limits_{i = 1}^{K - 1} {\sum\limits_{j > i}^{K} {\left| {u_{i} } \right|\left| {u_{j} } \right|} } } \right] \\ & = B_{\rm max} \left[ {\sum\limits_{i = 1}^{K} {\left| {u_{i} } \right|^{2} } + \sum\limits_{i = 1}^{K - 1} {\sum\limits_{j > i}^{K} {\left| {u_{i} } \right|^{2} } } + \sum\limits_{i = 1}^{K - 1} {\sum\limits_{j > i}^{K} {\left| {u_{j} } \right|^{2} } } - \sum\limits_{i = 1}^{K - 1} {\sum\limits_{j > i}^{K} {\left( {\left| {u_{i} } \right| - \left| {u_{j} } \right|} \right)^{2} } } } \right] \\ & = B_{\rm max} \left[ \begin{array}{l} \sum\limits_{i = 1}^{K} {\left| {u_{i} } \right|^{2} } + [(K - 1)\left| {u_{1} } \right|^{2} + \cdots + \left| {u_{K - 1} } \right|^{2} ] \hfill \\ \quad +\, [(\left| {u_{2} } \right|^{2} + \cdots + \left| {u_{K} } \right|^{2} ) + \cdots + \left| {u_{K} } \right|^{2} ] - \sum\limits_{i = 1}^{K - 1} {\sum\limits_{j > i}^{K} {\left( {\left| {u_{i} } \right| - \left| {u_{j} } \right|} \right)^{2} } } \hfill \\ \end{array} \right] \\ & = B_{\rm max} \left[ {\sum\limits_{i = 1}^{K} {\left| {u_{i} } \right|^{2} + (K - 1)} \sum\limits_{i = 1}^{K} {\left| {u_{i} } \right|^{2} } - \sum\limits_{i = 1}^{K - 1} {\sum\limits_{j > i}^{K} {\left( {\left| {u_{i} } \right| - \left| {u_{j} } \right|} \right)^{2} } } } \right] \\ & \le B_{\rm max} \left[ {K\sum\limits_{i = 1}^{K} {\left| {u_{i} } \right|^{2} } - \sum\limits_{i = 1}^{K - 1} {\sum\limits_{j > i}^{K} {\left( {\left| {u_{i} } \right| - \left| {u_{j} } \right|} \right)^{2} } } } \right] \\ & \le KB_{\rm max} \left| {\mathbf{u}} \right|^{2} . \\ \end{aligned} $$

### B.2 Proof of Eq. (3.3f)

By Taylor’s theorem, the $$ \overset{\lower0.5em\hbox{$\smash{\scriptscriptstyle\frown}$}}{F}_{j} $$ for $$ j = M + 1, \ldots ,N + 1 $$ can be written as

B.10 $$ \begin{aligned} \overset{\lower0.5em\hbox{$\smash{\scriptscriptstyle\frown}$}}{F}_{j} & = F(z_{j} ;{\mathbf{s}}^{{\prime }} ;{\mathbf{\overset{\lower0.5em\hbox{$\smash{\scriptscriptstyle\frown}$}}{n^{{\prime }}} }} ) \\ & = \left. {F(z_{j} ;{\mathbf{s}}^{{\prime }} ;{\mathbf{\overset{\lower0.5em\hbox{$\smash{\scriptscriptstyle\frown}$}}{n^{{\prime }}} }} )} \right|_{{z_{j} = s_{{{\text{cid}}(j)}} }} + \left. {\frac{{\partial F(z_{j} ;{\mathbf{s}}^{{\prime }} ;{\mathbf{\overset{\lower0.5em\hbox{$\smash{\scriptscriptstyle\frown}$}}{n^{{\prime }}} }} )}}{{\partial z_{j} }}} \right|_{{z_{j} = s_{{j{\text{T}}}} }} (s_{j} - s_{{{\text{cid}}(j)}} ) \\ & = \left. {\frac{{\partial F(z_{j} ;{\mathbf{s}}^{{\prime }} ;{\mathbf{\overset{\lower0.5em\hbox{$\smash{\scriptscriptstyle\frown}$}}{n^{{\prime }}} }} )}}{{\partial z_{j} }}} \right|_{{z_{j} = s_{{j{\text{T}}}} }} \rho_{j} \varepsilon, \\ \end{aligned} $$

with some appropriately chosen parameter $s_{{j{\text{T}}}} \in [s_{j} ,s_{{{\text{cid}}(j)}} ]$. Hence,

B.11 $$\begin{aligned} \frac{1}{\varepsilon }\left| {\overset{\lower0.5em\hbox{$\smash{\scriptscriptstyle\frown}$}}{F}_{j} } \right| = \frac{1}{\varepsilon }\left| {\left. {\frac{{\partial F(z_{j} ;{\mathbf{s}}^{{\prime }} ;{\mathbf{\overset{\lower0.5em\hbox{$\smash{\scriptscriptstyle\frown}$}}{n^{{\prime }}} }} )}}{{\partial z_{j} }}} \right|_{{z_{j} = s_{{j{\text{T}}}} }} \rho_{j} \varepsilon } \right| \\ \le \rho_{j} \hbox{max} \left\{ {\left. {\left| {\frac{{\partial F(z_{j} ;{\mathbf{s}}^{{\prime }} ;{\mathbf{\overset{\lower0.5em\hbox{$\smash{\scriptscriptstyle\frown}$}}{n^{{\prime }}} }} )}}{{\partial z_{j} }}} \right|_{{z_{j} = s_{{j{\text{T}}}} }} } \right|s_{{j{\text{T}}}} \in [s_{j} ,s_{{{\text{cid}}(j)}} ]} \right\} = :\overset{\lower0.5em\hbox{$\smash{\scriptscriptstyle\frown}$}}{C_{{{\text{Fz,}}j}}^{{\prime }}} , \end{aligned}$$

and

B.12 $$ \overset{\lower0.5em\hbox{$\smash{\scriptscriptstyle\frown}$}}{C_{\text{Fz}}^{{\prime }}} : = \mathop {\hbox{max} }\limits_{{j \in \{ M + 1, \ldots ,N + 1\} }} \overset{\lower0.5em\hbox{$\smash{\scriptscriptstyle\frown}$}}{C_{{{\text{Fz,}}j}}^{{\prime }}} = \hbox{max} \left\{ {\left. {\left| {\frac{{\partial F(z_{j} ;{\mathbf{s}}^{{\prime }} ;{\mathbf{\overset{\lower0.5em\hbox{$\smash{\scriptscriptstyle\frown}$}}{n^{{\prime }}} }} )}}{{\partial z_{j} }}} \right|_{{z_{j} = s_{{j{\text{T}}}} }} } \right| \, j = M + 1, \ldots ,N + 1,\;s_{{j{\text{T}}}} \in [s_{j} ,s_{{{\text{cid}}(j)}} ]} \right\} $$

because $$ \rho_{j} = 0 $$ for $$ j = 1, \ldots ,M $$. □

### B.3 Proof of Lemma 2

Using Taylor’s theorem, Eq. (3.3d) in the main text can be transformed to

B.13 $$ R_{i} = \frac{1}{2}({\mathbf{m}}^{{\prime }} - {\mathbf{\overset{\lower0.5em\hbox{$\smash{\scriptscriptstyle\frown}$}}{m^{{\prime }}} }} )^{\text{T}} \left. {\frac{{\partial^{2} \overset{\lower0.5em\hbox{$\smash{\scriptscriptstyle\smile}$}}{F} (s_{i} ;{\mathbf{s}}^{{\prime }} ;{\mathbf{m}}^{{\prime }} )}}{{\partial {\mathbf{m}}^{{\prime }} \partial {\mathbf{m}}^{{{\prime }{\text{T}}}} }}} \right|_{{{\mathbf{m}}^{{\prime }} = {\mathbf{m}}_{\text{T}}^{{\prime }} }} ({\mathbf{m}}^{{\prime }} - {\mathbf{\overset{\lower0.5em\hbox{$\smash{\scriptscriptstyle\frown}$}}{m^{{\prime }} } }}) , $$

where $$ {\mathbf{m}}_{\text{T}}^{{\prime }} = \theta_{\text{T}} \left( {{\mathbf{m}}^{{\prime }} - {\mathbf{\overset{\lower0.5em\hbox{$\smash{\scriptscriptstyle\frown}$}}{m^{{\prime }} } }}} \right) + {\mathbf{\overset{\lower0.5em\hbox{$\smash{\scriptscriptstyle\frown}$}}{m^{{\prime }} } }}$$ with some appropriately chosen $$ \theta_{\text{T}} \in [0,1] $$. By Eq. (3.4a) in the main text,

B.14 $$ \begin{aligned} \left| {{\mathbf{m}}^{{\prime }} - {\mathbf{\overset{\lower0.5em\hbox{$\smash{\scriptscriptstyle\frown}$}}{m^{{\prime }}} }} } \right| & = \sqrt {\left| {{\mathbf{m}} - {\mathbf{\overset{\lower0.5em\hbox{$\smash{\scriptscriptstyle\frown}$}}{m} }}} \right|^{2} + \left| {(m_{M + 1} , \ldots ,m_{N + 1} )^{\text{T}} - (\overset{\lower0.5em\hbox{$\smash{\scriptscriptstyle\frown}$}}{m}_{M + 1} , \ldots ,\overset{\lower0.5em\hbox{$\smash{\scriptscriptstyle\frown}$}}{m}_{N + 1} )^{\text{T}} } \right|^{2} } \\ & = \sqrt {\left| {{\mathbf{m}} - {\mathbf{\overset{\lower0.5em\hbox{$\smash{\scriptscriptstyle\frown}$}}{m} }}} \right|^{2} + \left| {(\varepsilon n_{M + 1} , \ldots ,\varepsilon n_{N + 1} )^{\text{T}} - (\varepsilon \overset{\lower0.5em\hbox{$\smash{\scriptscriptstyle\frown}$}}{n}_{M + 1} , \ldots ,\varepsilon \overset{\lower0.5em\hbox{$\smash{\scriptscriptstyle\frown}$}}{n}_{N + 1} )^{\text{T}} } \right|^{2} } \\ & \le \sqrt {\left| {{\mathbf{m}} - {\mathbf{\overset{\lower0.5em\hbox{$\smash{\scriptscriptstyle\frown}$}}{m} }}} \right|^{2} + \varepsilon^{2} \mathop { \hbox{max} }\limits_{{i \in \left\{ {M + 1, \ldots ,N + 1} \right\}}} \left( {\left| {n_{i} - \overset{\lower0.5em\hbox{$\smash{\scriptscriptstyle\frown}$}}{n}_{i} } \right|^{2} } \right)(N - M + 1)} \\ & \le \sqrt {\varepsilon^{2} C_{{\mathbf{m}}}^{2} + \varepsilon^{2} \eta^{2} (N - M + 1)} \\ & \le \varepsilon \sqrt {C_{{\mathbf{m}}}^{2} + (N - M + 1)\eta^{2} } = :\varepsilon C_{{\mathbf{m}}}^{{\prime }} . \\ \end{aligned} $$

Thus, the result from Appendix B.1 translates as

B.15 $$ \begin{aligned} \left| {R_{i} } \right| & = \frac{1}{2}\left| {({\mathbf{m}}^{{\prime }} - {\mathbf{\overset{\lower0.5em\hbox{$\smash{\scriptscriptstyle\frown}$}}{m^{{\prime }}} }} )^{\text{T}} \left. {\frac{{\partial^{2} \overset{\lower0.5em\hbox{$\smash{\scriptscriptstyle\smile}$}}{F} (s_{i} ;{\mathbf{s}}^{{\prime }} ;{\mathbf{m}}^{{\prime }} )}}{{\partial {\mathbf{m}}^{{\prime }} \partial {\mathbf{m}}^{{{\prime }{\text{T}}}} }}} \right|_{{{\mathbf{m}}^{{\prime }} = {\mathbf{m}}^{{{\prime }{\text{T}}}} }} ({\mathbf{m}}^{{\prime }} - {\mathbf{\overset{\lower0.5em\hbox{$\smash{\scriptscriptstyle\frown}$}}{m^{{\prime }}} }} )} \right| \\ & \le \frac{1}{2}\left\| {\left. {\frac{{\partial^{2} \overset{\lower0.5em\hbox{$\smash{\scriptscriptstyle\smile}$}}{F} (s_{i} ;{\mathbf{s}}^{{\prime }} ;{\mathbf{n}}^{{\prime }} )}}{{\partial {\mathbf{m}}^{{\prime }} \partial {\mathbf{m}}^{{{\prime }{\text{T}}}} }}} \right|_{{{\mathbf{m^{\prime}}} = {\mathbf{m^{\prime}}}_{\text{T}} }} } \right\|_{\text{Q}} \left| {{\mathbf{m}}^{{\prime }} - {\mathbf{\overset{\lower0.5em\hbox{$\smash{\scriptscriptstyle\frown}$}}{m^{{\prime }}} }} } \right|^{2} \\ & \le \frac{1}{2}C_{{{\text{F}}{\mathbf{mm}}}}^{{\prime }} \left| {{\mathbf{m}}^{{\prime }} - {\mathbf{\overset{\lower0.5em\hbox{$\smash{\scriptscriptstyle\frown}$}}{m^{{\prime }} } }}} \right|^{2} \\ & \le \frac{1}{2}\varepsilon^{2} C_{{{\text{F}}{\mathbf{mm}}}}^{{\prime }} C_{{\mathbf{m}}}^{{{\prime }2}} , \\ \end{aligned} $$

where, as proved in the next subsection,

B.16 $$\begin{aligned} C_{{{\text{F}}{\mathbf{mm}}}}^{{\prime }} & : = \hbox{max} \left\{ {\left\| {\frac{{\partial^{2} \overset{\lower0.5em\hbox{$\smash{\scriptscriptstyle\smile}$}}{F} (s_{i} ;{\mathbf{s}}^{{\prime }} ;{\mathbf{m}}^{{\prime }} )}}{{\partial {\mathbf{m}}^{{\prime }} \partial {\mathbf{m}}^{{{\prime }{\text{T}}}} }}} \right\|_{\text{Q}} \;\;\left| {\begin{array}{*{20}l} {i = 1, \ldots ,N + 1,} \hfill \\ {m_{1} , \ldots ,m_{M} \in [0,\eta ],} \hfill \\ {m_{M + 1} , \ldots ,m_{N + 1} \in [0,\varepsilon \eta ]} \hfill \\ \end{array} } \right.} \right\} \\ & \le (N + 1)\hbox{max} \left\{ {C_{\text{Fnn}}^{{\prime }} ,2C_{\text{Fsnn}}^{{\prime }} ,C_{\text{Fssnn}}^{{\prime }} } \right\} . \end{aligned} $$

□

### B.4 Finding $C_{{{\text{F}}{\mathbf{mm}}}}^{{\prime }}$

Here we prove Eq. (B.16). Analogously to Appendix A, we start from the observation that by the smoothness axiom (i) and bounded-world axiom (v) there exists a constant $C_{\text{Fnn}}^{{\prime }}$ such that

B.17 $$ \left| {\frac{{\partial F(s_{i} ;{\mathbf{s}}^{{\prime }} ;{\mathbf{n}}^{{\prime }} )}}{{\partial n_{k} \partial n_{j} }}} \right| \le C_{\text{Fnn}}^{{\prime }} $$

for all $i,j,k = 1, \ldots ,N + 1$, with

B.18 $$ C_{\text{Fnn}}^{{\prime }} : = \hbox{max} \left\{ {\left. {\left| {\frac{{\partial F(s_{i} ;{\mathbf{s}}^{{\prime }} ;{\mathbf{n}}^{{\prime }} )}}{{\partial n_{k} \partial n_{j} }}} \right| \, } \right|i,j,k = 1, \ldots ,N + 1,\;{\mathbf{n}}^{{\prime }} \in [0,\eta ]^{N + 1} } \right\} . $$

From Eqs. (A.9) and (A.10), the second derivative of the fitness-generating function with respect to $m_{j}$ and $m_{k}$ is expressed as

B.19 $$ \frac{{\partial^{2} \overset{\lower0.5em\hbox{$\smash{\scriptscriptstyle\smile}$}}{F} (s_{i} ;{\mathbf{s}}^{{\prime }} ;{\mathbf{m}}^{{\prime }} )}}{{\partial m_{k} \partial m_{j} }} = \left\{ {\begin{array}{*{20}l} {\frac{\partial }{{\partial n_{k} }} {\frac{{\partial \overset{\lower0.5em\hbox{$\smash{\scriptscriptstyle\smile}$}}{F} (s_{i} ;{\mathbf{s}}^{{\prime }} ;{\mathbf{m}}^{{\prime }} )}}{{\partial m_{j} }}}} \hfill & {{\text{for}}\;\;k = 1, \ldots ,M} \hfill \\ {\frac{1}{\varepsilon }\left\{ {\frac{\partial }{{\partial n_{k} }}\left\{{\frac{{\partial \overset{\lower0.5em\hbox{$\smash{\scriptscriptstyle\smile}$}}{F} (s_{i} ;{\mathbf{s}}^{{\prime }} ;{\mathbf{m}}^{{\prime }} )}}{{\partial m_{j} }}} \right\} - \frac{\partial }{{\partial n_{{{\text{cid}}(k)}} }} {\frac{{\partial \overset{\lower0.5em\hbox{$\smash{\scriptscriptstyle\smile}$}}{F} (s_{i} ;{\mathbf{s}}^{{\prime }} ;{\mathbf{m}}^{{\prime }} )}}{{\partial m_{j} }}}} \right\}} \hfill & {{\text{for}}\;\;k = M + 1, \ldots ,N + 1,} \hfill \\ \end{array} } \right. $$

while Eqs. (A.9) and (A.13) are combined into

B.20 $$ \frac{{\partial \overset{\lower0.5em\hbox{$\smash{\scriptscriptstyle\smile}$}}{F} (s_{i} ;{\mathbf{s}}^{{\prime }} ;{\mathbf{m}}^{{\prime }} )}}{{\partial m_{j} }} = \left\{ {\begin{array}{*{20}l} {\frac{{\partial F(s_{i} ;{\mathbf{s}}^{{\prime }} ;{\mathbf{n}}^{{\prime }} )}}{{\partial n_{j} }}} \hfill & {{\text{for}}\;\;j = 1, \ldots ,M} \hfill \\ {\left[ {\frac{{\partial^{2} F(s_{i} ;{\mathbf{s}}^{{\prime }} ;{\mathbf{n}}^{{\prime }} )}}{{\partial s_{j} \partial n_{j} }} - \frac{{\partial^{2} F(s_{i} ;{\mathbf{s}}^{{\prime }} ;{\mathbf{n}}^{{\prime }} )}}{{\partial s_{j} \partial n_{{{\text{cid}}(j)}} }}} \right]_{{s_{j} = s_{{j{\text{T}}}} }} }\rho_{j} \hfill & {{\text{for}}\;\;j = M + 1, \ldots ,N + 1} \hfill \\ \end{array} } \right. $$

with some appropriately chosen $s_{{j{\text{T}}}} \in [s_{j} ,s_{{{\text{cid}}(j)}} ]$. By substituting Eqs. (B.20) into (B.19), we see for $j,k = 1, \ldots ,M$

B.21 $$ \frac{{\partial^{2} \overset{\lower0.5em\hbox{$\smash{\scriptscriptstyle\smile}$}}{F} (s_{i} ;{\mathbf{s}}^{{\prime }} ;{\mathbf{m}}^{{\prime }} )}}{{\partial m_{k} \partial m_{j} }} = \frac{{\partial F(s_{i} ;{\mathbf{s}}^{{\prime }} ;{\mathbf{n}}^{{\prime }} )}}{{\partial n_{k} \partial n_{j} }} , $$

and for $j = M + 1, \ldots ,N + 1$ and $k = 1, \ldots ,M$ (and equivalently for $k = M + 1, \ldots ,N + 1$ and *j *= 1,…, *M*)

B.22 $$ \frac{{\partial^{2} \overset{\lower0.5em\hbox{$\smash{\scriptscriptstyle\smile}$}}{F} (s_{i} ;{\mathbf{s}}^{{\prime }} ;{\mathbf{m}}^{{\prime }} )}}{{\partial m_{k} \partial m_{j} }} = \left[ {\frac{{\partial^{3} F(s_{i} ;{\mathbf{s}}^{{\prime }} ;{\mathbf{n}}^{{\prime }} )}}{{\partial n_{k} \partial s_{j} \partial n_{j} }} - \frac{{\partial^{3} F(s_{i} ;{\mathbf{s}}^{{\prime }} ;{\mathbf{n}}^{{\prime }} )}}{{\partial n_{k} \partial s_{j} \partial n_{{{\text{cid}}(j)}} }}} \right]_{{s_{j} = s_{{j{\text{T}}}} }} \rho_{j} . $$

For $j,k = M + 1, \ldots ,N + 1$, we first transform Eq. (B.19) and then substitute Eq. (B.20) into the equation,

B.23 $$ \begin{aligned} \frac{{\partial^{2} \overset{\lower0.5em\hbox{$\smash{\scriptscriptstyle\smile}$}}{F} (s_{i} ;{\mathbf{s}}^{{\prime }} ;{\mathbf{m}}^{{\prime }} )}}{{\partial m_{k} \partial m_{j} }} & = \frac{1}{\varepsilon }\left[ {\frac{{\partial^{2} \overset{\lower0.5em\hbox{$\smash{\scriptscriptstyle\smile}$}}{F} (s_{i} ;{\mathbf{s}}^{{\prime }} ;{\mathbf{m}}^{{\prime }} )}}{{\partial n_{k} \partial m_{j} }} - \frac{{\partial^{2} \overset{\lower0.5em\hbox{$\smash{\scriptscriptstyle\smile}$}}{F} (s_{i} ;{\mathbf{s}}^{{\prime }} ;{\mathbf{m}}^{{\prime }} )}}{{\partial n_{{{\text{cid}}(k)}} \partial m_{j} }}} \right]_{{s_{k} = s_{{{\text{cid}}(k)}} }} \\ & \quad + \,\frac{1}{\varepsilon }\left\{ {\frac{\partial }{{\partial s_{k} }}\left[ {\frac{{\partial^{2} \overset{\lower0.5em\hbox{$\smash{\scriptscriptstyle\smile}$}}{F} (s_{i} ;{\mathbf{s}}^{{\prime }} ;{\mathbf{m}}^{{\prime }} )}}{{\partial n_{k} \partial m_{j} }} - \frac{{\partial^{2} \overset{\lower0.5em\hbox{$\smash{\scriptscriptstyle\smile}$}}{F} (s_{i} ;{\mathbf{s}}^{{\prime }} ;{\mathbf{m}}^{{\prime }} )}}{{\partial n_{{{\text{cid}}(k)}} \partial m_{j} }}} \right]} \right\}_{{s_{k} = s_{{k{\text{T}}}} }} (s_{k} - s_{{{\text{cid}}(k)}} ) \\ & = \rho_{k} \left\{ {\frac{\partial }{{\partial s_{k} }}\left[ {\frac{{\partial^{2} \overset{\lower0.5em\hbox{$\smash{\scriptscriptstyle\smile}$}}{F} (s_{i} ;{\mathbf{s}}^{{\prime }} ;{\mathbf{m}}^{{\prime }} )}}{{\partial n_{k} \partial m_{j} }} - \frac{{\partial^{2} \overset{\lower0.5em\hbox{$\smash{\scriptscriptstyle\smile}$}}{F} (s_{i} ;{\mathbf{s}}^{{\prime }} ;{\mathbf{m}}^{{\prime }} )}}{{\partial n_{{{\text{cid}}(k)}} \partial m_{j} }}} \right]} \right\}_{{s_{k} = s_{{k{\text{T}}}} }} \\ & = \rho_{k} \rho_{j} \left[ {\frac{{\partial^{4} F(s_{i} ;{\mathbf{s}}^{{\prime }} ;{\mathbf{n}}^{{\prime }} )}}{{\partial s_{k} \partial n_{k} \partial s_{j} \partial n_{j} }} - \frac{{\partial^{4} F(s_{i} ;{\mathbf{s}}^{{\prime }} ;{\mathbf{n}}^{{\prime }} )}}{{\partial s_{k} \partial n_{k} \partial s_{j} \partial n_{{{\text{cid}}(j)}} }}} \right. \\ & \quad - \,\left. {\frac{{\partial^{4} F(s_{i} ;{\mathbf{s}}^{{\prime }} ;{\mathbf{n}}^{{\prime }} )}}{{\partial s_{k} \partial n_{{{\text{cid}}(k)}} \partial s_{j} \partial n_{j} }} + \frac{{\partial^{4} F(s_{i} ;{\mathbf{s}}^{{\prime }} ;{\mathbf{n}}^{{\prime }} )}}{{\partial s_{k} \partial n_{{{\text{cid}}(k)}} \partial s_{j} \partial n_{{{\text{cid}}(j)}} }}} \right]_{{s_{k} = s_{{k{\text{T}}}} ,s_{j} = s_{{j{\text{T}}}} }} , \\ \end{aligned} $$

with some appropriately chosen $s_{{k{\text{T}}}} \in [s_{k} ,s_{{{\text{cid}}(k)}} ]$. Hence, from Eqs. (B.21) to (B.23), we find for *j*, *k *= 1, …, *M*

B.24 $$ \left| {\frac{{\partial \overset{\lower0.5em\hbox{$\smash{\scriptscriptstyle\smile}$}}{F} (s_{i} ;{\mathbf{s}}^{{\prime }} ;{\mathbf{m}}^{{\prime }} )}}{{\partial m_{k} \partial m_{j} }}} \right| \le C_{\text{Fnn}}^{{\prime }} , $$

for $j = M + 1, \ldots ,N + 1$ and $k = 1, \ldots ,M$ (and equivalently for $k = M + 1, \ldots ,N + 1$ and *j *= 1, …, *M*)

B.25 $$ \left| {\frac{{\partial \overset{\lower0.5em\hbox{$\smash{\scriptscriptstyle\smile}$}}{F} (s_{i} ;{\mathbf{s}}^{{\prime }} ;{\mathbf{m}}^{{\prime }} )}}{{\partial m_{k} \partial m_{j} }}} \right| \le C_{\text{Fsnn}}^{{\prime }} $$

with

B.26 $$ C_{\text{Fsnn}}^{{\prime }} : = \hbox{max} \left\{ {\left| {\frac{{\partial^{3} F(s_{i} ;{\mathbf{s}}^{{\prime }} ;{\mathbf{n}}^{{\prime }} )}}{{\partial s_{j} \partial n_{k} \partial n_{j} }} - \frac{{\partial^{3} F(s_{i} ;{\mathbf{s}}^{{\prime }} ;{\mathbf{n}}^{{\prime }} )}}{{\partial s_{j} \partial n_{k} \partial n_{{{\text{cid}}(j)}} }}} \right|_{{s_{j} = s_{{j{\text{T}}}} }} \left| {\begin{array}{*{20}l} {i = 1, \ldots ,N + 1,} \hfill \\ {j = M + 1, \ldots ,N + 1,} \hfill \\ {k = 1, \ldots ,M,} \hfill \\ {{\mathbf{n}}^{{\prime }} \in [0,\eta ]^{N + 1} ,\;s_{{j{\text{T}}}} \in [s_{j} ,s_{{{\text{cid}}(j)}} ]} \hfill \\ \end{array} } \right.} \right\} , $$

and for *j*, *k* = *M* + 1, …, *N* + 1

B.27 $$ \left| {\frac{{\partial \overset{\lower0.5em\hbox{$\smash{\scriptscriptstyle\smile}$}}{F} (s_{i} ;{\mathbf{s}}^{{\prime }} ;{\mathbf{m}}^{{\prime }} )}}{{\partial m_{k} \partial m_{j} }}} \right| \le C_{\text{Fssnn}}^{{\prime }} $$

with

B.28 $$ C_{\text{Fssnn}}^{{\prime }} : = \hbox{max} \left\{ {\left. \begin{array}{l} \dfrac{{\partial^{4} F(s_{i} ;{\mathbf{s}}^{{\prime }} ;{\mathbf{n}}^{{\prime }} )}}{{\partial s_{k} \partial s_{j} \partial n_{k} \partial n_{j} }} - \frac{{\partial^{4} F(s_{i} ;{\mathbf{s}}^{{\prime }} ;{\mathbf{n}}^{{\prime }} )}}{{\partial s_{k} \partial s_{j} \partial n_{k} \partial n_{{{\text{cid}}(j)}} }} \hfill \\ \quad - \,\dfrac{{\partial^{4} F(s_{i} ;{\mathbf{s}}^{{\prime }} ;{\mathbf{n}}^{{\prime }} )}}{{\partial s_{k} \partial s_{j} \partial n_{{{\text{cid}}(k)}} \partial n_{j} }} + \frac{{\partial^{4} F(s_{i} ;{\mathbf{s}}^{{\prime }} ;{\mathbf{n}}^{{\prime }} )}}{{\partial s_{k} \partial s_{j} \partial n_{{{\text{cid}}(k)}} \partial n_{{{\text{cid}}(j)}} }} \hfill \\ \end{array} \right|_{{s_{k} = s_{{k{\text{T}}}} ,s_{j} = s_{{j{\text{T}}}} }} \left| {\begin{array}{*{20}l} {i = 1, \ldots ,N + 1,} \hfill \\ {j,k = M + 1, \ldots ,N + 1,} \hfill \\ {{\mathbf{n}}^{{\prime }} \in [0,\eta ]^{N + 1} ,} \hfill \\ {s_{{j{\text{T}}}} \in [s_{j} ,s_{{{\text{cid}}(j)}} ],} \hfill \\ {s_{{k{\text{T}}}} \in [s_{k} ,s_{{{\text{cid}}(k)}} ]} \hfill \\ \end{array} } \right.} \right\}. $$

Collecting Eqs. (B.24), (B.25), and (B.27), we find

B.29 $$ \left| {\frac{{\partial \overset{\lower0.5em\hbox{$\smash{\scriptscriptstyle\smile}$}}{F} (s_{i} ;{\mathbf{s}}^{{\prime }} ;{\mathbf{m}}^{{\prime }} )}}{{\partial m_{k} \partial m_{j} }}} \right| \le \hbox{max} \left\{ {C_{\text{Fnn}}^{{\prime }} ,C_{\text{Fsnn}}^{{\prime }} ,C_{\text{Fssnn}}^{{\prime }} } \right\} = :C_{\text{Fmm}}^{{\prime }} . $$

Finally, for

B.30 $$ \frac{{\partial^{2} \overset{\lower0.5em\hbox{$\smash{\scriptscriptstyle\smile}$}}{F} (s_{i} ;{\mathbf{s}}^{{\prime }} ;{\mathbf{m}}^{{\prime }} )}}{{\partial {\mathbf{m}}^{{\prime }} \partial {\mathbf{m}}^{{{\prime }{\text{T}}}} }} = \left( {\begin{array}{*{20}c} {\frac{{\partial^{2} \overset{\lower0.5em\hbox{$\smash{\scriptscriptstyle\smile}$}}{F} (s_{i} ;{\mathbf{s}}^{{\prime }} ;{\mathbf{m}}^{{\prime }} )}}{{\partial m_{1} \partial m_{1} }}} & \ldots & {\frac{{\partial^{2} \overset{\lower0.5em\hbox{$\smash{\scriptscriptstyle\smile}$}}{F} (s_{i} ;{\mathbf{s}}^{{\prime }} ;{\mathbf{m}}^{{\prime }} )}}{{\partial m_{1} \partial m_{N + 1} }}} \\ \ldots & \ddots & \ldots \\ {\frac{{\partial^{2} \overset{\lower0.5em\hbox{$\smash{\scriptscriptstyle\smile}$}}{F} (s_{i} ;{\mathbf{s}}^{{\prime }} ;{\mathbf{m}}^{{\prime }} )}}{{\partial m_{N + 1} \partial m_{1} }}} & \ldots & {\frac{{\partial^{2} \overset{\lower0.5em\hbox{$\smash{\scriptscriptstyle\smile}$}}{F} (s_{i} ;{\mathbf{s}}^{{\prime }} ;{\mathbf{m}}^{{\prime }} )}}{{\partial m_{N + 1} \partial m_{N + 1} }}} \\ \end{array} } \right) , $$

we find its upper bound by using Eq. (B.8), as

B.31 $$ \left\| {\frac{{\partial^{2} \overset{\lower0.5em\hbox{$\smash{\scriptscriptstyle\smile}$}}{F} (s_{i} ;{\mathbf{s}}^{{\prime }} ;{\mathbf{m}}^{{\prime }} )}}{{\partial {\mathbf{m}}^{{\prime }} \partial {\mathbf{m}}^{{{\prime {\rm T}}}} }}} \right\|_{\text{Q}} \le (N + 1)C_{\text{Fmm}}^{{\prime }} = (N + 1)\hbox{max} \left\{ {C_{\text{Fnn}}^{{\prime }} ,C_{\text{Fsnn}}^{{\prime }} ,C_{\text{Fssnn}}^{{\prime }} } \right\} = :C_{{{\text{F}}{\mathbf{mm}}}}^{{\prime }} . $$

□

## Appendix C: Proof of Lemmas 3 and 7

### C.1 Expansion of $f$

We assume that non-representative phenotypes, $s_{j}$ for $j = M + 1, \ldots ,N + 1$, are functions of $\varepsilon$, $s_{j} = s_{{{\text{cid}}(j)}} + \varepsilon \rho_{j}$, so that when $\varepsilon \to 0$ they converge to their representative phenotypes, $s_{{{\text{cid}}(j)}}$. Then ${\mathbf{s}}^{{\prime }}$ is expressed as ${\mathbf{s}}^{{\prime }} = {\mathbf{s}}_{\text{a}}^{{\prime }} + \varepsilon {\varvec{\uprho}}^{{\prime }}$ with ${\mathbf{s}}_{\text{a}}^{{\prime }} : = (s_{1} , \ldots ,s_{M} ,s_{{{\text{cid}}(M + 1)}} , \ldots ,s_{{{\text{cid}}(N + 1)}} )^{\text{T}}$ and ${\varvec{\uprho}}^{{\prime }} : = (0, \ldots ,0,\rho_{M + 1} , \ldots ,\rho_{N + 1} )^{\text{T}}$. Notice that $\left| {\rho_{j} } \right| \le 1$ because within-cluster phenotypic differences are assumed not to exceed $\varepsilon$, i.e., $\left| {s_{j} - s_{{{\text{cid(}}j )}} } \right| \le \varepsilon$ for all $j = 1, \ldots ,N + 1$. By Taylor’s theorem and the exchangeability axiom (vi), the fitness of $s_{j}$ can be expanded in $\varepsilon$ around $\varepsilon = 0$, as

C.1 $$ \begin{aligned} F(s_{j} ;{\mathbf{s}}^{{\prime }} ;{\mathbf{n}}^{{\prime }} ) & = \left. {F(s_{{{\text{cid}}(j)}} + \varepsilon \rho_{j} ;{\mathbf{s}}_{\text{a}}^{{\prime }} + \varepsilon {\varvec{\uprho}}^{{\prime }} ;{\mathbf{n}}^{{\prime }} )} \right|_{\varepsilon = 0} + \varepsilon \left. {\frac{{\partial F(s_{{{\text{cid}}(j)}} + \varepsilon \rho_{j} ;{\mathbf{s}}_{\text{a}}^{{\prime }} + \varepsilon {\varvec{\uprho}}^{{\prime }} ;{\mathbf{n}}^{{\prime }} )}}{\partial \varepsilon }} \right|_{{\varepsilon = \varepsilon_{{{\text{T}}j}} }} \\ & = F(s_{{{\text{cid}}(j)}} ;{\mathbf{s}}_{\text{a}} ;{\mathbf{m}}) + \varepsilon F_{\varepsilon j} , \\ \end{aligned} $$

with some appropriately chosen $\varepsilon_{{{\text{T}}j}} \in [0,\varepsilon ]$ and

C.2 $$ F_{\varepsilon j} : = \left. {\frac{{\partial F(s_{{{\text{cid}}(j)}} + \varepsilon \rho_{j} ;{\mathbf{s}}_{\text{a}}^{{\prime }} + \varepsilon {\varvec{\uprho}}^{{\prime }} ;{\mathbf{n}}^{{\prime }} )}}{\partial \varepsilon }} \right|_{{\varepsilon = \varepsilon_{{{\text{T}}j}} }} .$$

Here, $$ \left| {F_{\varepsilon j} } \right| $$ satisfies

C.3 $$ \left| {F_{\varepsilon j} } \right| \le \hbox{max} \left\{ {\left| {\frac{{\partial F(s_{{{\text{cid}}(j)}} + \varepsilon \rho_{j} ;{\mathbf{s}}_{\text{a}}^{{\prime }} + \varepsilon {\varvec{\uprho}}^{{\prime }} ;{\mathbf{n}}^{{\prime }} )}}{\partial \varepsilon }} \right|_{{\varepsilon = \varepsilon_{{{\text{T}}j}} }} \left| {\begin{array}{*{20}l} {j = 1, \ldots ,N + 1,} \hfill \\ {{\mathbf{n}}^{{\prime }} \in [0,\eta ]^{N + 1} ,\;\varepsilon_{{{\text{T}}j}} \in [0,\varepsilon ]} \hfill \\ \end{array} } \right.} \right\} = :C_{{{\text{F}}\varepsilon }}^{{\prime }} .$$

By using Eq. (C.1), $f(s_{i} ;{\mathbf{s}}^{{\prime }} ;{\mathbf{n}}^{{\prime }} )$ from Eq. (4.1b) can be expressed as

C.4 $$ \begin{aligned} f(s_{i} ;{\mathbf{s}}^{{\prime }} ;{\mathbf{n}}^{{\prime }} ) & = \sum\limits_{{j \in {\text{com}}(i)}} {p_{j} F(s_{j} ;{\mathbf{s}}^{{\prime }} ;{\mathbf{n}}^{{\prime }} )} \\ & = F(s_{i} ;{\mathbf{s}}_{\text{a}} ;{\mathbf{m}}) + \varepsilon \sum\limits_{{j \in {\text{com}}(i)}} {p_{j} F_{\varepsilon j} } . \\ \end{aligned} $$

We further expand $$ F(s_{i} ;{\mathbf{s}}_{\text{a}} ;{\mathbf{m}}) $$ around $$ {\mathbf{m}} = {\mathbf{\overset{\lower0.5em\hbox{$\smash{\scriptscriptstyle\frown}$}}{m} }} = (\overset{\lower0.5em\hbox{$\smash{\scriptscriptstyle\frown}$}}{m_{1}} , \ldots ,\overset{\lower0.5em\hbox{$\smash{\scriptscriptstyle\frown}$}}{m}_{M} )^{\text{T}} $$ as

C.5 $$ F(s_{i} ;{\mathbf{s}}_{\text{a}} ;{\mathbf{m}}) = F(s_{i} ;{\mathbf{s}}_{\text{a}} ;{\mathbf{\overset{\lower0.5em\hbox{$\smash{\scriptscriptstyle\frown}$}}{m} }}) + {\mathbf{b}}_{i}^{\text{T}} ({\mathbf{m}} - {\mathbf{\overset{\lower0.5em\hbox{$\smash{\scriptscriptstyle\frown}$}}{m} }}) + ({\mathbf{m}} - {\mathbf{\overset{\lower0.5em\hbox{$\smash{\scriptscriptstyle\frown}$}}{m} }})^{\text{T}} {\mathbf{F}}_{{{\text{mm}}i}} ({\mathbf{m}} - {\mathbf{\overset{\lower0.5em\hbox{$\smash{\scriptscriptstyle\frown}$}}{m} }}) , $$

where

C.6 $$ {\mathbf{b}}_{i}^{\text{T}} : = (b_{i1} , \ldots ,b_{iM} ) = \left( {\frac{{\partial F(s_{i} ;{\mathbf{s}}_{\text{a}} ;{\mathbf{m}})}}{{\partial m_{1} }}, \ldots ,\frac{{\partial F(s_{i} ;{\mathbf{s}}_{\text{a}} ;{\mathbf{m}})}}{{\partial m_{M} }}} \right)_{{{\mathbf{m}} = {\mathbf{\overset{\lower0.5em\hbox{$\smash{\scriptscriptstyle\frown}$}}{m} }}}} , $$

and

C.7 $$ {\mathbf{F}}_{{{\text{mm}}i}} : = \left. {\frac{{\partial^{2} F(s_{i} ;{\mathbf{s}}_{\text{a}} ;{\mathbf{m}})}}{{\partial {\mathbf{m}}\partial {\mathbf{m}}^{\text{T}} }}} \right|_{{{\mathbf{m}} = {\mathbf{m}}_{\text{T}} }} = \left( {\begin{array}{*{20}c} {\frac{{\partial^{2} F(s_{i} ;{\mathbf{s}}_{\text{a}} ;{\mathbf{m}})}}{{\partial m_{1}^{2} }}} & \cdots & {\frac{{\partial^{2} F(s_{i} ;{\mathbf{s}}_{\text{a}} ;{\mathbf{m}})}}{{\partial m_{1} \partial m_{M} }}} \\ \vdots & \ddots & \vdots \\ {\frac{{\partial^{2} F(s_{i} ;{\mathbf{s}}_{\text{a}} ;{\mathbf{m}})}}{{\partial m_{M} \partial m_{1} }}} & \cdots & {\frac{{\partial^{2} F(s_{i} ;{\mathbf{s}}_{\text{a}} ;{\mathbf{m}})}}{{\partial m_{M}^{2} }}} \\ \end{array} } \right)_{{{\mathbf{m}} = {\mathbf{m}}_{\text{T}} }} , $$

where $$ {\mathbf{m}}_{\text{T}} : = \theta_{\text{T}} ({\mathbf{m}} - {\mathbf{\overset{\lower0.5em\hbox{$\smash{\scriptscriptstyle\frown}$}}{m} }}) + {\mathbf{\overset{\lower0.5em\hbox{$\smash{\scriptscriptstyle\frown}$}}{m} }} $$ with some appropriately chosen $$ \theta_{\text{T}} \in [0,1] $$. Notice that by Eq. (C.1) $$ F(s_{i} ;{\mathbf{s}}_{\text{a}} ;{\mathbf{\overset{\lower0.5em\hbox{$\smash{\scriptscriptstyle\frown}$}}{m} }}) $$ satisfies

C.8 $$ \begin{aligned} F(s_{i} ;{\mathbf{s}}^{{\prime }} ;{\mathbf{\overset{\lower0.5em\hbox{$\smash{\scriptscriptstyle\frown}$}}{n^{{\prime }}} }} ) & = F(s_{i} ;{\mathbf{s}}_{\text{a}} ;{\mathbf{\overset{\lower0.5em\hbox{$\smash{\scriptscriptstyle\frown}$}}{m} }}) + \varepsilon \left. {\frac{{\partial F(s_{i} + \varepsilon \rho_{i} ;{\mathbf{s}}_{\text{a}}^{{\prime }} + \varepsilon {\varvec{\uprho}}^{{\prime }} ;{\mathbf{\overset{\lower0.5em\hbox{$\smash{\scriptscriptstyle\frown}$}}{n^{{\prime }}} }} )}}{\partial \varepsilon }} \right|_{{\varepsilon = \varepsilon_{{{\text{T}}i}} }} \\ & = F(s_{i} ;{\mathbf{s}}_{\text{a}} ;{\mathbf{\overset{\lower0.5em\hbox{$\smash{\scriptscriptstyle\frown}$}}{m} }}) + \varepsilon \overset{\lower0.5em\hbox{$\smash{\scriptscriptstyle\frown}$}}{F_{\varepsilon i}} , \\ \end{aligned} $$

which gives

C.9 $$ F(s_{i} ;{\mathbf{s}}_{\text{a}} ;{\mathbf{\overset{\lower0.5em\hbox{$\smash{\scriptscriptstyle\frown}$}}{m} }}) = F(s_{i} ;{\mathbf{s}}^{{\prime }} ;{\mathbf{\overset{\lower0.5em\hbox{$\smash{\scriptscriptstyle\frown}$}}{n^{{\prime }}} }} ) - \varepsilon \overset{\lower0.5em\hbox{$\smash{\scriptscriptstyle\frown}$}}{F_{\varepsilon i}} . $$

Thus, combining Eqs. (C.4), (C.5), and (C.9), we obtain

C.10 $$ \begin{aligned} f(s_{i} ;{\mathbf{s}}^{{\prime }} ;{\mathbf{n}}^{{\prime }} ) & = {\mathbf{b}}_{i}^{\text{T}} ({\mathbf{m}} - {\mathbf{\overset{\lower0.5em\hbox{$\smash{\scriptscriptstyle\frown}$}}{m} }}) + ({\mathbf{m}} - {\mathbf{\overset{\lower0.5em\hbox{$\smash{\scriptscriptstyle\frown}$}}{m} }})^{\text{T}} {\mathbf{F}}_{{{\text{mm}}i}} ({\mathbf{m}} - {\mathbf{\overset{\lower0.5em\hbox{$\smash{\scriptscriptstyle\frown}$}}{m} }}) \\ & \quad + \,\varepsilon \left[ {\frac{1}{\varepsilon }F(s_{i} ;{\mathbf{s}}^{{\prime }} ;{\mathbf{\overset{\lower0.5em\hbox{$\smash{\scriptscriptstyle\frown}$}}{n^{{\prime }}} }} ) - \overset{\lower0.5em\hbox{$\smash{\scriptscriptstyle\frown}$}}{F_{\varepsilon i}} + \sum\limits_{{j \in {\text{com}}(i)}} {p_{j} F_{\varepsilon j} } } \right] \\ & = {\mathbf{b}}_{i}^{\text{T}} ({\mathbf{m}} - {\mathbf{\overset{\lower0.5em\hbox{$\smash{\scriptscriptstyle\frown}$}}{m} }}) + r_{{{\text{f}}i}} \left| {{\mathbf{m}} - {\mathbf{\overset{\lower0.5em\hbox{$\smash{\scriptscriptstyle\frown}$}}{m} }}} \right|^{2} + \varepsilon h_{{{\text{f}}i}} , \\ \end{aligned} $$

where

C.11 $$ \begin{aligned} r_{{{\text{f}}i}} & : = \frac{{({\mathbf{m}} - {\mathbf{\overset{\lower0.5em\hbox{$\smash{\scriptscriptstyle\frown}$}}{m} }})^{\text{T}} {\mathbf{F}}_{{{\text{mm}}i}} ({\mathbf{m}} - {\mathbf{\overset{\lower0.5em\hbox{$\smash{\scriptscriptstyle\frown}$}}{m} }})}}{{\left| {{\mathbf{m}} - {\mathbf{\overset{\lower0.5em\hbox{$\smash{\scriptscriptstyle\frown}$}}{m} }}} \right|^{2} }}, \\ h_{{{\text{f}}i}} & : = \frac{1}{\varepsilon }F(s_{i} ;{\mathbf{s}}^{{\prime }} ;{\mathbf{\overset{\lower0.5em\hbox{$\smash{\scriptscriptstyle\frown}$}}{n^{{\prime }}} }} ) - \overset{\lower0.5em\hbox{$\smash{\scriptscriptstyle\frown}$}}{F_{\varepsilon i}} + \sum\limits_{{j \in {\text{com}}(i)}} {p_{j} F_{\varepsilon j} } . \\ \end{aligned} $$

Here

C.12 $$ \begin{aligned} \left| {r_{{{\text{f}}i}} } \right| & = \left| {\frac{{({\mathbf{m}} - {\mathbf{\overset{\lower0.5em\hbox{$\smash{\scriptscriptstyle\frown}$}}{m} }})^{\text{T}} {\mathbf{F}}_{{{\text{mm}}i}} ({\mathbf{m}} - {\mathbf{\overset{\lower0.5em\hbox{$\smash{\scriptscriptstyle\frown}$}}{m} }})}}{{\left| {{\mathbf{m}} - {\mathbf{\overset{\lower0.5em\hbox{$\smash{\scriptscriptstyle\frown}$}}{m} }}} \right|^{2} }}} \right| \\ & \le \hbox{max} \left\{ {\left. {\left\| {\frac{{\partial^{2} F(s_{i} ;{\mathbf{s}}_{\text{a}} ;{\mathbf{m}})}}{{\partial {\mathbf{m}}\partial {\mathbf{m}}^{\text{T}} }}} \right\|_{\text{Q}} } \right|\;i = 1, \ldots ,M,{\mathbf{m}} \in [0,\eta ]^{M} } \right\} = :C_{{{\text{F}}{\mathbf{mm}}}}  \\ \end{aligned} $$

and by Eqs. (C.3) and (3.3f) in the main text, i.e., $$ \left| {F(s_{i} ;{\mathbf{s}}^{{\prime }} ;{\mathbf{\overset{\lower0.5em\hbox{$\smash{\scriptscriptstyle\frown}$}}{n^{{\prime }}} }} )} \right| = \left| {\overset{\lower0.5em\hbox{$\smash{\scriptscriptstyle\frown}$}}{F_{i} }} \right| = 0 $$ for $$ i = 1, \ldots ,N $$ and $$ \left| {F(s_{N + 1} ;{\mathbf{s}}^{{\prime }} ;{\mathbf{\overset{\lower0.5em\hbox{$\smash{\scriptscriptstyle\frown}$}}{n^{{\prime }}} }} )} \right| = \left| {\overset{\lower0.5em\hbox{$\smash{\scriptscriptstyle\frown}$}}{F_{N + 1} } }\right| \le C_{\text{Fz}}^{{\prime }} \varepsilon $$,

C.13 $$ \begin{aligned} \left| {h_{{{\text{f}}i}} } \right| & = \left| {\frac{1}{\varepsilon }F(s_{i} ;{\mathbf{s}}^{{\prime }} ;{\mathbf{\overset{\lower0.5em\hbox{$\smash{\scriptscriptstyle\frown}$}}{n^{{\prime }}} }} ) - \overset{\lower0.5em\hbox{$\smash{\scriptscriptstyle\frown}$}}{F_{\varepsilon i}} + \sum\limits_{{j \in {\text{com}}(i)}} {p_{j} F_{\varepsilon j} } } \right| \\ & \le \frac{1}{\varepsilon }\left| {F(s_{i} ;{\mathbf{s}}^{{\prime }} ;{\mathbf{\overset{\lower0.5em\hbox{$\smash{\scriptscriptstyle\frown}$}}{n^{{\prime }}} }} )} \right| + \left| {\overset{\lower0.5em\hbox{$\smash{\scriptscriptstyle\frown}$}}{F}_{\varepsilon i} } \right| + \left| {\sum\limits_{{j \in {\text{com}}(i)}} {p_{j} F_{\varepsilon j} } } \right| \\ & \le \overset{\lower0.5em\hbox{$\smash{\scriptscriptstyle\frown}$}}{C_{\text{Fz}}^{{\prime }}} + 2C_{{{\text{F}}\varepsilon }}^{{\prime }} . \\ \end{aligned} $$

Substituting Eq. (C.10) into Eq. (4.2) in the main text yields

C.14 $$ \frac{{{\text{d}}{\mathbf{m}}}}{{{\text{d}}t}} = {\text{diag}}({\mathbf{m}})\left[ {{\mathbf{B}}({\mathbf{m}} - {\mathbf{\overset{\lower0.5em\hbox{$\smash{\scriptscriptstyle\frown}$}}{m} }}) + {\mathbf{r}}_{\text{f}} \left| {{\mathbf{m}} - {\mathbf{\overset{\lower0.5em\hbox{$\smash{\scriptscriptstyle\frown}$}}{m} }}} \right|^{2} + \varepsilon {\mathbf{h}}_{\text{f}} } \right] , $$

where

C.15 $$ {\mathbf{B}}: = \left( {\begin{array}{*{20}c} {{\mathbf{b}}_{1}^{\text{T}} } \\ \vdots \\ {{\mathbf{b}}_{M}^{\text{T}} } \\ \end{array} } \right) = \left( {\begin{array}{*{20}c} {b_{11} } & \ldots & {b_{1M} } \\ \vdots & \ddots & \vdots \\ {b_{M1} } & \ldots & {b_{MM} } \\ \end{array} } \right) $$

with ${\mathbf{b}}_{i}^{\text{T}}$ defined by Eq. (C.6), ${\mathbf{h}}_{\text{f}} = (h_{{{\text{f}}1}} , \ldots ,h_{{{\text{f}}M}} )^{\text{T}}$, and ${\mathbf{r}}_{\text{f}} = (r_{{{\text{f}}1}} , \ldots ,r_{{{\text{f}}M}} )^{\text{T}}$, with

C.16 $$ \begin{aligned} \left| {{\mathbf{r}}_{\text{f}} } \right| & = \sqrt {\sum\limits_{i = 1}^{M} {\left| {{\mathbf{r}}_{{{\text{f}}i}} } \right|^{2} } } \le \sqrt M C_{{{\text{F}}{\mathbf{mm}}}} = :C_{{{\mathbf{r}}{\text{f}}}} , \\ \left| {{\mathbf{h}}_{\text{f}} } \right| & = \sqrt {\sum\limits_{i = 1}^{M} {\left| {{\mathbf{h}}_{{{\text{f}}i}} } \right|^{2} } } \le \sqrt M \mathop {\hbox{max} }\limits_{i} \left( {\left| {{\mathbf{h}}_{{{\text{f}}i}} } \right|} \right) = \sqrt M \left[ {\overset{\lower0.5em\hbox{$\smash{\scriptscriptstyle\frown}$}}{C_{\text{Fz}}^{\prime } }+ 2C_{{{\text{F}}\varepsilon }}^{\prime } } \right] = :C_{{{\mathbf{h}}{\text{f}}}} . \\ \end{aligned} $$

### C.2 Expansion of $\text{dm} / \text{dt}$

Eq. (C.14) is further transformed into

C.17 $$ \begin{aligned} \frac{{{\text{d}}{\mathbf{m}}}}{{{\text{d}}t}} & = {\text{diag(}}{\mathbf{m}} ){\mathbf{B}}({\mathbf{m}} - {\mathbf{\overset{\lower0.5em\hbox{$\smash{\scriptscriptstyle\frown}$}}{m} }}){\text{ + diag(}}{\mathbf{m}} ){\mathbf{r}}_{\text{f}} \left| {{\mathbf{m}} - {\mathbf{\overset{\lower0.5em\hbox{$\smash{\scriptscriptstyle\frown}$}}{m} }}} \right|^{2} + \varepsilon {\text{diag(}}{\mathbf{m}} ){\mathbf{h}}_{\text{f}} \\ & {\text{ = diag}}\left( {\left[ {{\mathbf{m}} - {\mathbf{\overset{\lower0.5em\hbox{$\smash{\scriptscriptstyle\frown}$}}{m} }}} \right] + {\mathbf{\overset{\lower0.5em\hbox{$\smash{\scriptscriptstyle\frown}$}}{m} }}} \right){\mathbf{B}}({\mathbf{m}} - {\mathbf{\overset{\lower0.5em\hbox{$\smash{\scriptscriptstyle\frown}$}}{m} }}){\text{ + diag(}}{\mathbf{m}} ){\mathbf{r}}_{\text{f}} \left| {{\mathbf{m}} - {\mathbf{\overset{\lower0.5em\hbox{$\smash{\scriptscriptstyle\frown}$}}{m} }}} \right|^{2} + \varepsilon {\text{diag(}}{\mathbf{m}} ){\mathbf{h}}_{\text{f}} \\ & = {\text{diag}}({\mathbf{\overset{\lower0.5em\hbox{$\smash{\scriptscriptstyle\frown}$}}{m} }}){\mathbf{B}}({\mathbf{m}} - {\mathbf{\overset{\lower0.5em\hbox{$\smash{\scriptscriptstyle\frown}$}}{m} }}) \\ & \quad + \,\left[ {{\text{diag}}({\mathbf{m}} - {\mathbf{\overset{\lower0.5em\hbox{$\smash{\scriptscriptstyle\frown}$}}{m} }}){\mathbf{B}}({\mathbf{m}} - {\mathbf{\overset{\lower0.5em\hbox{$\smash{\scriptscriptstyle\frown}$}}{m} }}) + {\text{diag(}}{\mathbf{m}} ){\mathbf{r}}_{\text{f}} \left| {{\mathbf{m}} - {\mathbf{\overset{\lower0.5em\hbox{$\smash{\scriptscriptstyle\frown}$}}{m} }}} \right|^{2} } \right] + \varepsilon {\text{diag(}}{\mathbf{m}} ){\mathbf{h}}_{\text{f}} \\ & = {\mathbf{J}}({\mathbf{m}} - {\mathbf{\overset{\lower0.5em\hbox{$\smash{\scriptscriptstyle\frown}$}}{m} }}) + {\mathbf{r}}_{\text{m}} \left| {{\mathbf{m}} - {\mathbf{\overset{\lower0.5em\hbox{$\smash{\scriptscriptstyle\frown}$}}{m} }}} \right|^{2} + \varepsilon {\mathbf{h}}_{\text{m}} \\ \end{aligned} $$

where

C.18 $$ {\mathbf{J}}: = {\text{diag}}({\mathbf{\overset{\lower0.5em\hbox{$\smash{\scriptscriptstyle\frown}$}}{m} }}){\mathbf{B}} = \left( {\begin{array}{*{20}c} {\overset{\lower0.5em\hbox{$\smash{\scriptscriptstyle\frown}$}}{m}_{1} b_{11} } & \ldots & {\overset{\lower0.5em\hbox{$\smash{\scriptscriptstyle\frown}$}}{m}_{1} b_{1M} } \\ \ldots & \ddots & \ldots \\ {\overset{\lower0.5em\hbox{$\smash{\scriptscriptstyle\frown}$}}{m}_{M} b_{M1} } & \ldots & {\overset{\lower0.5em\hbox{$\smash{\scriptscriptstyle\frown}$}}{m}_{M} b_{MM} } \\ \end{array} } \right) $$

is the Jacobian matrix at $$ {\mathbf{\overset{\lower0.5em\hbox{$\smash{\scriptscriptstyle\frown}$}}{m} }} $$, and $$ {\mathbf{r}}_{{\mathbf{m}}} = (r_{{{\text{m}}1}} , \ldots ,r_{{{\text{m}}M}} )^{\text{T}} $$ and $$ {\mathbf{h}}_{{\mathbf{m}}} = (h_{{{\text{m}}1}} , \ldots ,h_{{{\text{m}}M}} )^{\text{T}} $$ are given by

C.19 $$ \begin{aligned} {\mathbf{r}}_{\text{m}} & : { = }\frac{{{\text{diag}}({\mathbf{m}} - {\mathbf{\overset{\lower0.5em\hbox{$\smash{\scriptscriptstyle\frown}$}}{m} }}){\mathbf{B}}({\mathbf{m}} - {\mathbf{\overset{\lower0.5em\hbox{$\smash{\scriptscriptstyle\frown}$}}{m} }})}}{{\left| {{\mathbf{m}} - {\mathbf{\overset{\lower0.5em\hbox{$\smash{\scriptscriptstyle\frown}$}}{m} }}} \right|^{2} }} + {\text{diag}}({\mathbf{m}}){\mathbf{r}}_{\text{f}} , \\ {\mathbf{h}}_{\text{m}} & : = {\text{diag}}({\mathbf{m}}){\mathbf{h}}_{\text{f}} , \\ \end{aligned} $$

where from Eq. (C.16) we find

C.20 $$ \begin{aligned} \left| {{\mathbf{r}}_{\text{m}} } \right| & \le \frac{{\left| {{\text{diag}}({\mathbf{m}} - {\mathbf{\overset{\lower0.5em\hbox{$\smash{\scriptscriptstyle\frown}$}}{m} }}){\mathbf{B}}({\mathbf{m}} - {\mathbf{\overset{\lower0.5em\hbox{$\smash{\scriptscriptstyle\frown}$}}{m} }})} \right|}}{{\left| {{\mathbf{m}} - {\mathbf{\overset{\lower0.5em\hbox{$\smash{\scriptscriptstyle\frown}$}}{m} }}} \right|^{2} }} + \left| {{\text{diag}}({\mathbf{m}}){\mathbf{r}}_{\text{f}} } \right| \\ & \le \frac{{\left\| {{\text{diag}}({\mathbf{m}} - {\mathbf{\overset{\lower0.5em\hbox{$\smash{\scriptscriptstyle\frown}$}}{m} }})} \right\|\left\| {\mathbf{B}} \right\|}}{{\left| {{\mathbf{m}} - {\mathbf{\overset{\lower0.5em\hbox{$\smash{\scriptscriptstyle\frown}$}}{m} }}} \right|}} + \left\| {{\text{diag}}({\mathbf{m}})} \right\|\left| {{\mathbf{r}}_{\text{f}} } \right| \\ & = \frac{{\max\limits_{i = 1, \ldots M} \left( {\left| {m_{i} - \overset{\lower0.5em\hbox{$\smash{\scriptscriptstyle\frown}$}}{m}_{i} } \right|} \right)\left\| {\mathbf{B}} \right\|}}{{\left| {{\mathbf{m}} - {\mathbf{\overset{\lower0.5em\hbox{$\smash{\scriptscriptstyle\frown}$}}{m} }}} \right|}} + \mathop {\hbox{max} }\limits_{i = 1, \ldots M} \left( {\left| {m_{i} } \right|} \right)\left| {{\mathbf{r}}_{\text{f}} } \right| \\ & \le \sqrt {\frac{{\max\limits_{i = 1, \ldots M} \left( {\left| {m_{i} - \overset{\lower0.5em\hbox{$\smash{\scriptscriptstyle\frown}$}}{m}_{i} } \right|} \right)^{2} }}{{\sum\nolimits_{i = 1}^{M} {\left| {m_{i} - \overset{\lower0.5em\hbox{$\smash{\scriptscriptstyle\frown}$}}{m}_{i} } \right|^{2} } }}} \left\| {\mathbf{B}} \right\| + \eta \left| {{\mathbf{r}}_{\text{f}} } \right| \le \left\| {\mathbf{B}} \right\| + \eta C_{\text{rf}} = \left\| {\mathbf{B}} \right\| + \eta \sqrt {MC}_{{{\text{F}}{\mathbf{mm}}}} = :C_{\text{rm}} , \\ \left| {{\mathbf{h}}_{\text{m}} } \right| & \le \left\| {{\text{diag}}({\mathbf{m}})} \right\|\left| {{\mathbf{h}}_{\text{f}} } \right| \le \mathop {\hbox{max} }\limits_{i = 1, \ldots M} \left( {\left| {m_{i} } \right|} \right)\left| {{\mathbf{h}}_{\text{f}}} \right| \le \eta C_{\text{hf}} = \eta \sqrt M \left[ {\overset{\lower0.5em\hbox{$\smash{\scriptscriptstyle\frown}$}}{C_{\text{Fz}}^{{\prime }}} + 2C_{{{\text{F}}\upvarepsilon}}^{{\prime }} } \right] = :C_{\text{hm}} \\ \end{aligned} $$

□

## Appendix D: Finding $C_{{\mathbf{r}}}$ and $C_{{\mathbf{h}}}$

Substituting $$ {\mathbf{x}} = (x_{1} , \ldots ,x_{M} )^{\text{T}} = {\mathbf{P}}({\mathbf{m}} - {\mathbf{\overset{\lower0.5em\hbox{$\smash{\scriptscriptstyle\frown}$}}{m} }}) $$ into Eq. (4.3a) in the main text gives Eq. (4.4), $$ \frac{{{\text{d}}{\mathbf{x}}}}{{{\text{d}}t}} = {\mathbf{Ax}} + {\mathbf{r}}\left| {\mathbf{x}} \right|^{2} + \varepsilon {\mathbf{h}} $$, where $$ {\mathbf{A}} = {\mathbf{PJP}}^{ - 1} $$ and

D.2 $$ \begin{aligned} {\mathbf{r}} & : = (r_{1} , \ldots ,r_{M} )^{\text{T}} = \frac{{{\mathbf{Pr}}_{\text{m}} \left| {{\mathbf{P}}^{ - 1} {\mathbf{x}}} \right|^{2} }}{{\left| {\mathbf{x}} \right|^{2} }}, \\ {\mathbf{h}} & : = (h_{1} , \ldots ,h_{M} )^{\text{T}} = {\mathbf{Ph}}_{\text{m}} . \\ \end{aligned} $$

There exist constants $C_{{\mathbf{r}}}$ and $C_{{\mathbf{h}}}$ such that $\left| {\mathbf{r}} \right| \le C_{{\mathbf{r}}}$ and $\left| {\mathbf{h}} \right| \le C_{{\mathbf{h}}}$, given by, e.g.,

D.3 $$ \begin{aligned} \left| {\mathbf{r}} \right| & \le \frac{{\left\| {\mathbf{P}} \right\|\left| {{\mathbf{r}}_{\text{m}} } \right|\left\| {{\mathbf{P}}^{ - 1} } \right\|^{2} \left| {\mathbf{x}} \right|^{2} }}{{\left| {\mathbf{x}} \right|^{2} }} \\ & = \left\| {\mathbf{P}} \right\|\left\| {{\mathbf{P}}^{ - 1} } \right\|^{2} \left| {{\mathbf{r}}_{\text{m}} } \right| \\ & \le \left\| {\mathbf{P}} \right\|\left\| {{\mathbf{P}}^{ - 1} } \right\|^{2} C_{{{\mathbf{rm}}}} = :C_{{\mathbf{r}}} , \\ \end{aligned} $$

and

D.4 $$ \left| {\mathbf{h}} \right| \le \left\| {\mathbf{P}} \right\|\left| {{\mathbf{h}}_{\text{m}} } \right| \le \left\| {\mathbf{P}} \right\|C_{{{\mathbf{hm}}}} = :C_{{\mathbf{h}}} . $$

By substituting Eqs. (C.20) into Eqs. (D.3) and (D.4), we obtain

D.5 $$ \begin{aligned} C_{{\mathbf{r}}} & = \left\| {\mathbf{P}} \right\|\left\| {{\mathbf{P}}^{ - 1} } \right\|^{2} \left[ {\left\| {\mathbf{B}} \right\| + \eta \sqrt M C_{{{\text{F}}{\mathbf{mm}}}} } \right], \\ C_{{\mathbf{h}}} & = \left\| {\mathbf{P}} \right\|\eta \sqrt M \left[ {\overset{\lower0.5em\hbox{$\smash{\scriptscriptstyle\frown}$}}{C_{\text{Fz}}^{{\prime }}} + 2C_{{{\text{F}}\varepsilon }}^{{\prime }} } \right]. \\ \end{aligned} $$

## Appendix E: Proof of Lemma 4

We first prove three simple cases: all eigenvalues are (1) distinct, (2) the same real number, or (3) the same complex number, and then combine them for proving the general case.

### E.1 All eigenvalues are distinct

We assume that all eigenvalues of the $M \times M$ matrix ${\mathbf{J}}$ are distinct, comprising $G$ real eigenvalues $\lambda_{1} , \ldots ,\lambda_{G}$ with corresponding real eigenvectors ${\mathbf{v}}_{1} , \ldots ,{\mathbf{v}}_{G}$ and $2H$ complex eigenvalues $\beta_{1} \pm i\omega_{1} , \ldots ,\beta_{H} \pm i\omega_{H}$ with corresponding complex eigenvectors ${\mathbf{u}}_{1} \pm i{\mathbf{w}}_{1} , \ldots ,{\mathbf{u}}_{H} \pm i{\mathbf{w}}_{H}$, where $G + 2H = M$. Then ${\mathbf{J}}$ can be decomposed as ${\mathbf{J}} = {\mathbf{P}}^{ - 1} {\mathbf{AP}}$, where

E.1 $$ \begin{aligned} {\mathbf{P}} & : = ({\mathbf{v}}_{1} , \ldots ,{\mathbf{v}}_{G} ,{\mathbf{u}}_{1} ,{\mathbf{w}}_{1} ,{\mathbf{u}}_{2} ,{\mathbf{w}}_{2} , \ldots ,{\mathbf{u}}_{H} ,{\mathbf{w}}_{H} ), \\ {\mathbf{A}} & = \left( {\begin{array}{*{20}l} {\lambda_{1} } \hfill & 0 \hfill & \ldots \hfill & \ldots \hfill & \ldots \hfill & \ldots \hfill & \ldots \hfill & 0 \hfill \\ 0 \hfill & \ddots \hfill & 0 \hfill & \ddots \hfill & \ddots \hfill & \ddots \hfill & \ddots \hfill & \vdots \hfill \\ \vdots \hfill & 0 \hfill & {\lambda_{G} } \hfill & 0 \hfill & 0 \hfill & \ddots \hfill & \ddots \hfill & \vdots \hfill \\ \vdots \hfill & \ddots \hfill & 0 \hfill & {\beta_{1} } \hfill & {\omega_{1} } \hfill & 0 \hfill & \ddots \hfill & \vdots \hfill \\ \vdots \hfill & \ddots \hfill & 0 \hfill & { - \,\omega_{1} } \hfill & {\beta_{1} } \hfill & 0 \hfill & \ddots \hfill & \vdots \hfill \\ \vdots \hfill & \ddots \hfill & \ddots \hfill & 0 \hfill & 0 \hfill & \ddots \hfill & 0 \hfill & 0 \hfill \\ \vdots \hfill & \ddots \hfill & \ddots \hfill & \ddots \hfill & \ddots \hfill & 0 \hfill & {\beta_{H} } \hfill & {\omega_{H} } \hfill \\ 0 \hfill & \ldots \hfill & \ldots \hfill & \ldots \hfill & \ldots \hfill & 0 \hfill & { - \,\omega_{H} } \hfill & {\beta_{H} } \hfill \\ \end{array} } \right). \\ \end{aligned} $$

Thus, for $\lambda_{\rm max} = \hbox{max} \left\{ {\text{Re} (\lambda_{1} ), \ldots ,\text{Re} (\beta_H \pm i \omega_H)} \right\} = \hbox{max} \left\{ {\lambda_{1} , \ldots ,\lambda_{G} ,\beta_{1} , \ldots ,\beta_{H} } \right\}$ defined by Eq. (4.5b),

E.2 $$ {\mathbf{x}}^{\text{T}} {\mathbf{Ax}} = {\mathbf{x}}^{\text{T}} \frac{1}{2}\left( {{\mathbf{A}} + {\mathbf{A}}^{\text{T}} } \right){\mathbf{x}} \le \lambda_{\rm max} \left| {\mathbf{x}} \right|^{2} $$

□

### E.2 All eigenvalues are the same real number

We assume that ${\mathbf{m}} = (m_{1} ,m_{2} ,m_{3} )^{\text{T}}$ for which all three eigenvalues of ${\mathbf{J}}$ are the same real number, i.e., $\lambda_{1} = \lambda_{2} = \lambda_{3} = \lambda$. Then there exists a regular matrix ${\mathbf{P}}_{\text{J}}$ that transforms ${\mathbf{J}}$ into the Jordan normal form ${\varvec{\Lambda}}: = {\mathbf{P}}_{\text{J}} {\mathbf{JP}}_{\text{J}}^{ - 1}$ with

E.3 $$ {\varvec{\Lambda}} = \left( {\begin{array}{ccc} \lambda &\quad 1 &\quad 0 \\ 0 &\quad \lambda &\quad 1 \\ 0 &\quad 0 &\quad \lambda \\ \end{array} } \right) . $$

Here we modify ${\mathbf{P}}_{\text{J}}$ to ${\mathbf{P}}$ by multiplying it with ${\varvec{\Theta}}$,

E.4 $$ {\mathbf{P}}: = {\mathbf{P}}_{\text{J}} {\varvec{\Theta}} $$

with

E.5 $$ {\varvec{\Theta}}: = \left( {\begin{array}{ccc} {a^{2} } &\quad a &\quad 1 \\ 0 &\quad a &\quad 1 \\ 0 &\quad 0 &\quad 1 \\ \end{array} } \right),\quad {\varvec{\Theta}}^{ - 1} = \left( {\begin{array}{ccc} {a^{ - 2} } &\quad { - a^{ - 2} } &\quad 0 \\ 0 &\quad {a^{ - 1} } &\quad { - a^{ - 1} } \\ 0 &\quad 0 &\quad 1 \\ \end{array} } \right) . $$

Then

E.6 $$ \begin{aligned} {\mathbf{A}} & = {\mathbf{PJP}}^{ - 1} \\ & = {\mathbf{\varTheta P}}_{\text{J}} {\mathbf{JP}}_{\text{J}}^{ - 1} {\varvec{\Theta}}^{ - 1} = \left( {\begin{array}{ccc} {a^{2} } &\quad a &\quad 1 \\ 0 &\quad a &\quad 1 \\ 0 &\quad 0 &\quad 1 \\ \end{array} } \right)\left( {\begin{array}{ccc} \lambda &\quad 1 &\quad 0 \\ 0 &\quad \lambda &\quad 1 \\ 0 &\quad 0 &\quad \lambda \\ \end{array} } \right)\left( {\begin{array}{ccc} {a^{ - 2} } &\quad { - a^{ - 2} } &\quad 0 \\ 0 &\quad {a^{ - 1} } &\quad { - a^{ - 1} } \\ 0 &\quad 0 &\quad 1 \\ \end{array} } \right) \\ & = \left( {\begin{array}{ccc} {a^{2} \lambda } &\quad {a^{2} + a\lambda } &\quad {a + \lambda } \\ 0 &\quad {a\lambda } &\quad {a + \lambda } \\ 0 &\quad 0 &\quad \lambda \\ \end{array} } \right)\left( {\begin{array}{ccc} {a^{ - 2} } &\quad { - a^{ - 2} } &\quad 0 \\ 0 &\quad {a^{ - 1} } &\quad { - a^{ - 1} } \\ 0 &\quad 0 &\quad 1 \\ \end{array} } \right) = \left( {\begin{array}{ccc} \lambda &\quad a &\quad 0 \\ 0 &\quad \lambda &\quad a \\ 0 &\quad 0 &\quad \lambda \\ \end{array} } \right). \\ \end{aligned} . $$

Thus, for $$ \lambda < 0, $$$$ a = \lambda /2 $$ and $$ {\mathbf{x}} = (x_{1} ,x_{2} ,x_{3} )^{\text{T}} = {\mathbf{P}}({\mathbf{m}} - {\mathbf{\overset{\lower0.5em\hbox{$\smash{\scriptscriptstyle\frown}$}}{m} }}) $$, we see

E.7 $$ \begin{aligned} {\mathbf{x}}^{\text{T}} {\mathbf{Ax}} & = {\mathbf{x}}^{\text{T}} \left( {\begin{array}{ccc} \lambda &\quad {\lambda /2} &\quad 0 \\ 0 &\quad \lambda &\quad {\lambda /2} \\ 0 &\quad 0 &\quad \lambda \\ \end{array} } \right){\mathbf{x}} = \frac{\lambda }{4}\left[ {4x_{1}^{2} + 4x_{2}^{2} + 4x_{3}^{2} + 2x_{1} x_{2} + 2x_{2} x_{3} } \right] \\ & = \frac{\lambda }{4}\left[ {3x_{1}^{2} + 2x_{2}^{2} + 3x_{3}^{2} + (x_{1} + x_{2} )^{2} + (x_{2} + x_{3} )^{2} } \right] \\ & \le \frac{\lambda }{4}\left[ {3x_{1}^{2} + 2x_{2}^{2} + 3x_{3}^{2} } \right] \le \frac{\lambda }{2}\left| {\mathbf{x}} \right|^{2}. \\ \end{aligned} .$$

Similarly, for a $W \times W$ matrix ${\mathbf{J}}$ with $W$ repeated real eigenvalues $\lambda$, its Jordan normal form ${\varvec{\Lambda}} = {\mathbf{P}}_{\text{J}} {\mathbf{JP}}_{\text{J}}^{ - 1}$ is expressed as

E.8 $$ {\varvec{\Lambda}} = \left( {\begin{array}{*{20}c} \lambda & 1 & {} & {} & {} \\ {} & \lambda & \ddots & {} & {} \\ {} & {} & \ddots & \ddots & {} \\ {} & {} & {} & \lambda & 1 \\ {} & {} & {} & {} & \lambda \\ \end{array} } \right) , $$

where, here and below, all blank components are zero. We define

E.9 $$ {\varvec{\Theta}}: = \left( {\begin{array}{*{20}c} {a^{W - 1} } & {a^{W - 2} } & {a^{W - 3} } & \cdots & a & 1 \\ {} & {a^{W - 2} } & {a^{W - 3} } & \cdots & a & 1 \\ {} & {} & \ddots & \ddots & \vdots & \vdots \\ {} & {} & {} & \ddots & \ddots & \vdots \\ {} & {} & {} & {} & a & 1 \\ {} & {} & {} & {} & {} & 1 \\ \end{array} } \right) $$

with its inverse

E.10 $$ {\varvec{\Theta}}^{ - 1} = \left( {\begin{array}{*{20}c} {a^{ - W + 1} } & { - a^{ - W + 1} } & {} & {} & {} & {} \\ {} & {a^{ - W + 2} } & { - a^{ - W + 2} } & {} & {} & {} \\ {} & {} & \ddots & \ddots & {} & {} \\ {} & {} & {} & \ddots & \ddots & {} \\ {} & {} & {} & {} & {a^{ - 1} } & { - a^{ - 1} } \\ {} & {} & {} & {} & {} & 1 \\ \end{array} } \right) , $$

which gives

E.11 $$ {\mathbf{A}} = {\mathbf{PJP}}^{ - 1} \, = {\mathbf{\varTheta P}}_{\text{J}} {\mathbf{JP}}_{\text{J}}^{ - 1} {\varvec{\Theta}}^{ - 1} = \left( {\begin{array}{*{20}c} \lambda & a & {} & {} & {} \\ {} & \lambda & \ddots & {} & {} \\ {} & {} & \ddots & \ddots & {} \\ {} & {} & {} & \lambda & a \\ {} & {} & {} & {} & \lambda \\ \end{array} } \right) . $$

Thus, for $$ \lambda < 0 $$, $$ a = \lambda /2 ,$$ and $$ {\mathbf{x}} = (x_{1} , \ldots ,x_{W} )^{\text{T}} = {\mathbf{P}}({\mathbf{m}} - {\mathbf{\overset{\lower0.5em\hbox{$\smash{\scriptscriptstyle\frown}$}}{m} }}), $$ we see

E.12 $$ \begin{aligned} {\mathbf{x}}^{\text{T}} {\mathbf{Ax}} & = \frac{\lambda }{4}\left[ {4x_{1}^{2} + \cdots + 4x_{W}^{2} + 2x_{1} x_{2} + 2x_{2} x_{3} + \cdots + 2x_{W - 1} x_{W} } \right] \\ & = \frac{\lambda }{4}\left[ {3x_{1}^{2} + 2x_{2}^{2} + \cdots + 2x_{W - 1}^{2} + 3x_{W}^{2} + (x_{1} + x_{2} )^{2} + \cdots + (x_{W - 1} + x_{W} )^{2} } \right] \\ & \le \frac{\lambda }{4}\left[ {3x_{1}^{2} + 2x_{2}^{2} + \cdots + 2x_{W - 1}^{2} + 3x_{W}^{2} } \right] \le \frac{\lambda }{2}\left| {\mathbf{x}} \right|^{2}. \\ \end{aligned}. $$

□

### E.3 All eigenvalues are the same complex number

We assume that ${\mathbf{m}} = (m_{1} ,m_{2} ,m_{3} ,m_{4} ,m_{5} ,m_{6} )^{\text{T}}$ for which the six-by-six matrix ${\mathbf{J}}$ has three sets of repeated complex eigenvalues, i.e., $\beta_{1} \pm i_{1} \omega$, $\beta_{2} \pm i_{2} \omega$ and $\beta_{3} \pm i_{3} \omega$ with $\beta_{1} = \beta_{2} = \beta_{3} = \beta$ and $\omega_{1} = \omega_{2} = \omega_{3} = \omega$. Then there exists a regular matrix ${\mathbf{P}}_{\text{J}}$ that standardizes ${\mathbf{J}}$ to ${\varvec{\Lambda}} = {\mathbf{P}}_{\text{J}} {\mathbf{JP}}_{\text{J}}^{ - 1}$ in the following normal form

E.13 $$ \begin{aligned} {\varvec{\Lambda}} & = \left( {\begin{array}{ccc} {\mathbf{C}} &\quad {{\mathbf{I}}_{2} } &\quad {\mathbf{0}} \\ {\mathbf{0}} &\quad {\mathbf{C}} &\quad {{\mathbf{I}}_{2} } \\ {\mathbf{0}} &\quad {\mathbf{0}} &\quad {\mathbf{C}} \\ \end{array} } \right), \\ {\mathbf{C}} & = \left( {\begin{array}{cc} \beta & \omega \\ { - \,\omega } & \beta \\ \end{array} } \right),\quad {\mathbf{I}}_{2} = \left( {\begin{array}{cc} 1 &\quad 0 \\ 0 &\quad 1 \\ \end{array} } \right) \\ \end{aligned} . $$

(Morris et al. 2003). Here we define a regular matrix ${\mathbf{P}}$ as

E.14 $$ {\mathbf{P}}: = {\mathbf{P}}_{\text{J}} {\varvec{\Theta}} $$

with

E.15 $$ \begin{aligned} {\varvec{\Theta}} & : = \left( {\begin{array}{ccc} {a^{2} {\mathbf{E}}} & {a{\mathbf{E}}} & {\mathbf{E}} \\ {\mathbf{0}} & {a{\mathbf{E}}} & {\mathbf{E}} \\ {\mathbf{0}} & {\mathbf{0}} & {\mathbf{E}} \\ \end{array} } \right),\quad {\varvec{\Theta}}^{ - 1} = \left( {\begin{array}{ccc} {a^{ - 2} {\mathbf{E}}^{ - 1} } & { - \,a^{ - 1} {\mathbf{E}}^{ - 1} } & {\mathbf{0}} \\ {\mathbf{0}} & {a^{ - 1} {\mathbf{E}}^{ - 1} } & { - \,a^{ - 1} {\mathbf{E}}^{ - 1} } \\ {\mathbf{0}} & {\mathbf{0}} & {{\mathbf{E}}^{ - 1} } \\ \end{array} } \right), \\ {\mathbf{E}} & : = \left( {\begin{array}{cc} 1 & { - \,1} \\ 1 & 1 \\ \end{array} } \right),\quad {\mathbf{E}}^{ - 1} = \frac{1}{2}\left( {\begin{array}{cc} 1 & 1 \\ { - \,1} & 1 \\ \end{array} } \right). \\ \end{aligned} $$

Then we see

E.16 $$ \begin{aligned} {\mathbf{A}} & = {\mathbf{PJP}}^{ - 1} \\ & = {\mathbf{\varvec{\Theta} {\mathbf{P}}}}_{\text{J}} {\mathbf{JP}}_{\text{J}}^{ - 1} {\varvec{\Theta}}^{ - 1} = \left( {\begin{array}{ccc} {a^{2} {\mathbf{E}}} & {a{\mathbf{E}}} & {\mathbf{E}} \\ {\mathbf{0}} & {a{\mathbf{E}}} & {\mathbf{E}} \\ {\mathbf{0}} & {\mathbf{0}} & {\mathbf{E}} \\ \end{array} } \right)\left( {\begin{array}{ccc} {\mathbf{C}} & {{\mathbf{I}}_{2} } & {\mathbf{0}} \\ {\mathbf{0}} & {\mathbf{C}} & {{\mathbf{I}}_{2} } \\ {\mathbf{0}} & {\mathbf{0}} & {\mathbf{C}} \\ \end{array} } \right)\left( {\begin{array}{ccc} {a^{ - 2} {\mathbf{E}}^{ - 1} } & { - a^{ - 2} {\mathbf{E}}^{ - 1} } & {\mathbf{0}} \\ {\mathbf{0}} & {a^{ - 1} {\mathbf{E}}^{ - 1} } & { - a^{ - 1} {\mathbf{E}}^{ - 1} } \\ {\mathbf{0}} & {\mathbf{0}} & {{\mathbf{E}}^{ - 1} } \\ \end{array} } \right) \\ & = \left( {\begin{array}{*{20}c} {a^{2} {\mathbf{EC}}} & {a^{2} {\mathbf{E}} + a{\mathbf{EC}}} & {a{\mathbf{E}} + {\mathbf{EC}}} \\ {\mathbf{0}} & {a{\mathbf{EC}}} & {a{\mathbf{E}} + {\mathbf{EC}}} \\ {\mathbf{0}} & {\mathbf{0}} & {{\mathbf{EC}}} \\ \end{array} } \right)\left( {\begin{array}{ccc} {a^{ - 2} {\mathbf{E}}^{ - 1} } & { - \,a^{ - 2} {\mathbf{E}}^{ - 1} } & {\mathbf{0}} \\ {\mathbf{0}} & {a^{ - 1} {\mathbf{E}}^{ - 1} } & { - \,a^{ - 1} {\mathbf{E}}^{ - 1} } \\ {\mathbf{0}} & {\mathbf{0}} & {{\mathbf{E}}^{ - 1} } \\ \end{array} } \right) \\ & = \left( {\begin{array}{*{20}c} {{\mathbf{ECE}}^{ - 1} } & {a{\mathbf{I}}_{2} } & {\mathbf{0}} \\ {\mathbf{0}} & {{\mathbf{ECE}}^{ - 1} } & {a{\mathbf{I}}_{2} } \\ {\mathbf{0}} & {\mathbf{0}} & {{\mathbf{ECE}}^{ - 1} } \\ \end{array} } \right) = \left( {\begin{array}{ccc} {\mathbf{C}} & {a{\mathbf{I}}_{2} } & {\mathbf{0}} \\ {\mathbf{0}} & {\mathbf{C}} & {a{\mathbf{I}}_{2} } \\ {\mathbf{0}} & {\mathbf{0}} & {\mathbf{C}} \\ \end{array} } \right). \\ \end{aligned} $$

Thus, for $$ \lambda < 0 $$, $$ a = \lambda /2, $$ and $$ {\mathbf{x}} = (x_{1} ,x_{2} ,x_{3} ,x_{4} ,x_{5} ,x_{6} )^{\text{T}} = {\mathbf{P}}({\mathbf{m}} - {\mathbf{\overset{\lower0.5em\hbox{$\smash{\scriptscriptstyle\frown}$}}{m} }}) $$, we see

E.17 $$ \begin{aligned} {\mathbf{x}}^{\text{T}} {\mathbf{Ax}} & = {\mathbf{x}}^{\text{T}} \left( {\begin{array}{ccc} {\mathbf{C}} & {a{\mathbf{I}}_{2} } & {\mathbf{0}} \\ {\mathbf{0}} & {\mathbf{C}} & {a{\mathbf{I}}_{2} } \\ {\mathbf{0}} & {\mathbf{0}} & {\mathbf{C}} \\ \end{array} } \right){\mathbf{x}} = {\mathbf{x}}^{\text{T}} \left( {\begin{array}{cccccc} \beta & \omega & {\beta /2} & 0 & 0 & 0 \\ { - \,\omega } & \beta & 0 & {\beta /2} & 0 & 0 \\ 0 & 0 & \beta & \omega & {\beta /2} & 0 \\ 0 & 0 & { - \,\omega } & \beta & 0 & {\beta /2} \\ 0 & 0 & 0 & 0 & \beta & \omega \\ 0 & 0 & 0 & 0 & { - \,\omega } & \beta \\ \end{array} } \right){\mathbf{x}} \\ & = \frac{\beta }{4}\left[ {4x_{1}^{2} + 4x_{2}^{2} + 4x_{3}^{2} + 4x_{4}^{2} + 4x_{5}^{2} + 4x_{6}^{2} + 2x_{1} x_{3} + 2x_{2} x_{4} + 2x_{3} x_{5} + 2x_{4} x_{6} } \right] \\ & = \frac{\beta }{4}\left[ {3x_{1}^{2} + 3x_{2}^{2} + 2x_{3}^{2} + 2x_{4}^{2} + 3x_{5}^{2} + 3x_{6}^{2} + (x_{1} + x_{3} )^{2} + (x_{2} + x_{4} )^{2} + (x_{3} + x_{5} )^{2} + (x_{4} + x_{6} )^{2} } \right] \\ & \le \frac{\beta }{4}\left[ {3x_{1}^{2} + 3x_{2}^{2} + 2x_{3}^{2} + 2x_{4}^{2} + 3x_{5}^{2} + 3x_{6}^{2} } \right] \le \frac{\beta }{2}\left| {\mathbf{x}} \right|^{2}. \\ \end{aligned} $$

Similarly, for a $2W \times 2W$ matrix ${\mathbf{J}}$ with $W$ sets of repeated complex eigenvalues, i.e., $\beta_{1} \pm i \omega_{1}, \ldots ,\beta_{W} \pm i \omega_{W}$ with $\beta_{1} = \cdots = \beta_{W} = \beta$ and $\omega_{1} = \cdots = \omega_{W} = \omega$, ${\varvec{\Lambda}} = {\mathbf{P}}_{\text{J}} {\mathbf{JP}}_{\text{J}}^{ - 1}$ is expressed as

E.18 $$ {\varvec{\Lambda}} = \left( {\begin{array}{ccccc} {\mathbf{C}} & {{\mathbf{I}}_{2} } & {} & {} & {} \\ {} & {\mathbf{C}} & \ddots & {} & {} \\ {} & {} & \ddots & \ddots & {} \\ {} & {} & {} & {\mathbf{C}} & {{\mathbf{I}}_{2} } \\ {} & {} & {} & {} & {\mathbf{C}} \\ \end{array} } \right),{\mathbf{C}} = \left( {\begin{array}{cc} \beta & \omega \\ { - \,\omega } & \beta \\ \end{array} } \right) , $$

where, here and below, all blank components are zero. We define

E.19 $$ {\varvec{\Theta}}: = \left( {\begin{array}{*{20}c} {a^{W - 1} {\mathbf{E}}} & {a^{W - 2} {\mathbf{E}}} & {a^{W - 3} {\mathbf{E}}} & \cdots & {a{\mathbf{E}}} & {\mathbf{E}} \\ {} & {a^{W - 2} {\mathbf{E}}} & {a^{W - 3} {\mathbf{E}}} & \cdots & {a{\mathbf{E}}} & {\mathbf{E}} \\ {} & {} & \ddots & \ddots & \vdots & \vdots \\ {} & {} & {} & \ddots & \ddots & \vdots \\ {} & {} & {} & {} & {a{\mathbf{E}}} & {\mathbf{E}} \\ {} & {} & {} & {} & {} & {\mathbf{E}} \\ \end{array} } \right) $$

with

E.20 $$ {\varvec{\Theta}}^{ - 1} = \left( {\begin{array}{*{20}c} {a^{ - W + 1} {\mathbf{E}}^{ - 1} } & { - \,a^{ - W + 1} {\mathbf{E}}^{ - 1} } & {} & {} & {} & {} \\ {} & {a^{ - W + 2} {\mathbf{E}}^{ - 1} } & { - \,a^{ - W + 2} {\mathbf{E}}^{ - 1} } & {} & {} & {} \\ {} & {} & \ddots & \ddots & {} & {} \\ {} & {} & {} & \ddots & \ddots & {} \\ {} & {} & {} & {} & {a^{ - 1} {\mathbf{E}}^{ - 1} } & { - \,a^{ - 1} {\mathbf{E}}^{ - 1} } \\ {} & {} & {} & {} & {} & {{\mathbf{E}}^{ - 1} } \\ \end{array} } \right) , $$

which gives

E.21 $$ {\mathbf{A}} = {\mathbf{PJP}}^{ - 1} \, = {\mathbf{\varTheta P}}_{\text{J}} {\mathbf{JP}}_{\text{J}}^{ - 1} {\varvec{\Theta}}^{ - 1} = \left( {\begin{array}{*{20}c} {\mathbf{C}} & {a{\mathbf{I}}_{2} } & {} & {} & {} \\ {} & {\mathbf{C}} & \ddots & {} & {} \\ {} & {} & \ddots & \ddots & {} \\ {} & {} & {} & {\mathbf{C}} & {a{\mathbf{I}}_{2} } \\ {} & {} & {} & {} & {\mathbf{C}} \\ \end{array} } \right) . $$

Thus, for $$ \lambda < 0 $$, $$ a = \lambda /2, $$ and $$ {\mathbf{x}} = (x_{1} , \ldots ,x_{2W} )^{\text{T}} = {\mathbf{P}}({\mathbf{m}} - {\mathbf{\overset{\lower0.5em\hbox{$\smash{\scriptscriptstyle\frown}$}}{m} }}) $$, we see

E.22 $$ \begin{aligned} {\mathbf{x}}^{\text{T}} {\mathbf{Ax}} & = \frac{\beta }{4}\left[ {4x_{1}^{2} + \cdots + 4x_{2W}^{2} + 2x_{1} x_{3} + \cdots + 2x_{2W - 2} x_{2W} } \right] \\ & = \frac{\beta }{4}\left[ 3x_{1}^{2} + 3x_{2}^{2} + 2x_{3}^{2} + \cdots + 2x_{2W - 2}^{2} + 3x_{2W - 1}^{2}  \right.\\  &\quad \left. + 3x_{2W}^{2} + (x_{1} + x_{3} )^{2} + \cdots + (x_{2W - 2} + x_{2W} )^{2}  \right] \\ & \le \frac{\beta }{4}\left[ {3x_{1}^{2} + 3x_{2}^{2} + 2x_{3}^{2} + \cdots + 2x_{2W - 2}^{2} + 3x_{2W - 1}^{2} + 3x_{2W}^{2} } \right] \le \frac{\beta }{2}\left| {\mathbf{x}} \right|^{2} . \\ \end{aligned} $$

□

### E.4 General case

By combining the aforementioned three cases, here we prove Lemma 4. For an arbitrary $M \times M$ matrix ${\mathbf{J}}$, there exists a regular matrix ${\mathbf{P}}_{\text{J}}$ that transforms ${\mathbf{J}}$ into ${\varvec{\Lambda}} = {\mathbf{P}}_{\text{J}} {\mathbf{JP}}_{\text{J}}^{ - 1}$ in the following form

E.23 $$ {\varvec{\Lambda}} = \left( {\begin{array}{*{20}c} {{\varvec{\Lambda}}^{\text{R}} } & {} & {} & {} & {} & {} & {} & {} \\ {} & {{\varvec{\Lambda}}^{\text{C}} } & {} & {} & {} & {} & {} & {} \\ {} & {} & {{\varvec{\Lambda}}_{1}^{\text{repR}} } & {} & {} & {} & {} & {} \\ {} & {} & {} & \ddots & {} & {} & {} & {} \\ {} & {} & {} & {} & {{\varvec{\Lambda}}_{K}^{\text{repR}} } & {} & {} & {} \\ {} & {} & {} & {} & {} & {{\varvec{\Lambda}}_{1}^{\text{repC}} } & {} & {} \\ {} & {} & {} & {} & {} & {} & \ddots & {} \\ {} & {} & {} & {} & {} & {} & {} & {{\varvec{\Lambda}}_{L}^{\text{repC}} } \\ \end{array} } \right) , $$

where, here and below, all blank components are zero, ${\varvec{\Lambda}}^{\text{R}}$ is a $G \times G$ diagonal matrix with its entries given by the distinct real eigenvalues $\lambda_{1}^{\text{R}} , \ldots ,\lambda_{G}^{\text{R}}$, and ${\varvec{\Lambda}}^{\text{C}}$ is a $2H \times 2H$ block diagonal matrix with its entries determined by the distinct complex eigenvalues $\beta_{1}^{\text{C}} \pm i\omega_{1}^{\text{C}} , \ldots ,\beta_{H}^{\text{C}} \pm i\omega_{H}^{\text{C}}$, i.e.,

E.24 $$ {\varvec{\Lambda}}^{\text{C}} = \left( {\begin{array}{ccc} {{\mathbf{C}}_{1}^{\text{C}} } & {} & {} \\ {} & \ddots & {} \\ {} & {} & {{\mathbf{C}}_{H}^{\text{C}} } \\ \end{array} } \right),\,\,\,{\mathbf{C}}_{h}^{\text{C}} = \left( {\begin{array}{*{20}c} {\beta_{h}^{\text{C}} } & {\omega_{h}^{\text{C}} } \\ { - \omega_{h}^{\text{C}} } & {\beta_{h}^{\text{C}} } \\ \end{array} } \right) , $$

for $h = 1, \ldots ,H$ (Morris et al. 2003). The subsequent matrices in Eq. (E.23), ${\varvec{\Lambda}}_{k}^{\text{repR}}$ for $k = 1, \ldots ,K$ are for repeated real eigenvalues, with their repetition numbers denoted by ${\text{repr}}(k)$. ${\varvec{\Lambda}}_{k}^{\text{repR}}$ is a ${\text{repr}}(k) \times {\text{repr}}(k)$ matrix expressed in the form of Eq. (E.8) with its diagonal entries $\lambda = \lambda_{k}^{\text{repR}}$. Finally, ${\varvec{\Lambda}}_{l}^{\text{repC}}$ for $l = 1, \ldots ,L$ are for repeated complex eigenvalues, with their repetition numbers denoted by ${\text{repc}}(l)$. ${\varvec{\Lambda}}_{l}^{\text{repC}}$ is a $2{\text{repc}}(l) \times 2{\text{repc}}(l)$ matrix expressed in the form of Eq. (E.18) with

E.25 $$ {\mathbf{C}} = {\mathbf{C}}_{l}^{\text{repC}} = \left( {\begin{array}{cc} {\beta_{l}^{\text{repC}} } & {\omega_{l}^{\text{repC}} } \\ { - \omega_{l}^{\text{repC}} } & {\beta_{l}^{\text{repC}} } \\ \end{array} } \right) . $$

Since ${\mathbf{J}}$ is an $M \times M$ matrix, $G + 2H + {\text{repr}}(1) + \cdots + {\text{repr}}(K) + 2[{\text{repc}}(1) + \cdots + {\text{rep}}(L)] = M$ is fulfilled. Here we define

E.26 $$ {\mathbf{P}}: = {\mathbf{P}}_{\text{J}} {\varvec{\Theta}} $$

with

E.27 $$ \begin{aligned} \hfill {\varvec{\Theta}}: = \left( {\begin{array}{*{20}c} {{\mathbf{I}}^{\text{R}} } & {} & {} & {} & {} & {} & {} & {} \\ {} & {{\mathbf{I}}^{\text{C}} } & {} & {} & {} & {} & {} & {} \\ {} & {} & {{\varvec{\Theta}}_{1}^{\text{repR}} } & {} & {} & {} & {} & {} \\ {} & {} & {} & \ddots & {} & {} & {} & {} \\ {} & {} & {} & {} & {{\varvec{\Theta}}_{K}^{\text{repR}} } & {} & {} & {} \\ {} & {} & {} & {} & {} & {{\varvec{\Theta}}_{1}^{{{\text{rep}}C}} } & {} & {} \\ {} & {} & {} & {} & {} & {} & \ddots & {} \\ {} & {} & {} & {} & {} & {} & {} & {{\varvec{\Theta}}_{L}^{\text{repC}} } \\ \end{array} } \right), \\ \hfill \\ \end{aligned} $$

where ${\mathbf{I}}^{\text{R}}$ and ${\mathbf{I}}^{\text{C}}$ are, respectively, $G \times G$ and $2H \times 2H$ identity matrices, ${\varvec{\Theta}}_{k}^{\text{repR}}$ is given by Eq. (E.9) with $W = {\text{repr(k)}}$, and ${\varvec{\Theta}}_{l}^{\text{repC}}$ is given by Eq. (E.18) with $W = {\text{repc(}}l )$. Then we see

E.28 $$ \begin{aligned} {\mathbf{A}} & = {{\varvec{\Theta} \bf{P}}}_{\text{J}} {\mathbf{JP}}_{\text{J}}^{ - 1} {\varvec{\Theta}}^{ - 1} = {{\varvec{\Theta} \varvec{\Lambda} \varvec{\Theta} }}^{ - 1} \\ & = \left( {\begin{array}{*{20}c} {{\varvec{\Lambda}}^{\text{R}} } & {} & {} & {} & {} & {} & {} & {} \\ {} & {{\varvec{\Lambda}}^{\text{C}} } & {} & {} & {} & {} & {} & {} \\ {} & {} & {{\mathbf{\overset{\lower0.5em\hbox{$\smash{\scriptscriptstyle\smile}$}}{\varvec{\Lambda} } }}_{1}^{\text{repR}} } & {} & {} & {} & {} & {} \\ {} & {} & {} & \ddots & {} & {} & {} & {} \\ {} & {} & {} & {} & {{\mathbf{\overset{\lower0.5em\hbox{$\smash{\scriptscriptstyle\smile}$}}{\varvec{\Lambda} } }}_{K}^{\text{repR}} } & {} & {} & {} \\ {} & {} & {} & {} & {} & {{\mathbf{\overset{\lower0.5em\hbox{$\smash{\scriptscriptstyle\smile}$}}{\varvec{\Lambda} } }}_{1}^{\text{repC}} } & {} & {} \\ {} & {} & {} & {} & {} & {} & \ddots & {} \\ {} & {} & {} & {} & {} & {} & {} & {{\mathbf{\overset{\lower0.5em\hbox{$\smash{\scriptscriptstyle\smile}$}}{\varvec{\Lambda} } }}_{L}^{\text{repC}} } \\ \end{array} } \right) \\ \end{aligned} , $$

where $$ {\mathbf{\overset{\lower0.5em\hbox{$\smash{\scriptscriptstyle\smile}$}}{\varvec{\Lambda} } }}_{k}^{\text{repR}} $$ is the same as $$ {\varvec{\Lambda}}_{k}^{\text{repR}} $$ except that all 1 s in $$ {\varvec{\Lambda}}_{k}^{\text{repR}} $$ are replaced with $$ \lambda_{k}^{\text{repR}} /2 $$ for $$ k = 1, \ldots ,K $$, and in the same manner $$ {\mathbf{\overset{\lower0.5em\hbox{$\smash{\scriptscriptstyle\smile}$}}{\varvec{\Lambda }} }}_{l}^{\text{repC}} $$ is the same as $$ {\varvec{\Lambda}}_{l}^{\text{repC}} $$ except that all $$ {\mathbf{I}}_{2} $$ in $$ {\varvec{\Lambda}}_{l}^{\text{repC}} $$ are replaced with $$ \beta_{l}^{\text{repC}} {\mathbf{I}}_{2} /2 $$ for $$ l = 1, \ldots ,L $$. Therefore, we get

E.29 $$ \begin{aligned} {\mathbf{x}}^{\text{T}} {\mathbf{Ax}} & = \left( {\begin{array}{*{20}c} {{\mathbf{x}}^{\text{R}} } \\ {{\mathbf{x}}^{\text{C}} } \\ {{\mathbf{x}}_{1}^{\text{repR}} } \\ \vdots \\ {{\mathbf{x}}_{K}^{\text{repR}} } \\ {{\mathbf{x}}_{1}^{\text{repC}} } \\ \vdots \\ {{\mathbf{x}}_{L}^{\text{repC}} } \\ \end{array} } \right)^{\text{T}} {\mathbf{A}}\left( {\begin{array}{*{20}c} {{\mathbf{x}}^{\text{R}} } \\ {{\mathbf{x}}^{\text{C}} } \\ {{\mathbf{x}}_{1}^{\text{repR}} } \\ \vdots \\ {{\mathbf{x}}_{K}^{\text{repR}} } \\ {{\mathbf{x}}_{1}^{\text{repC}} } \\ \vdots \\ {{\mathbf{x}}_{L}^{\text{repC}} } \\ \end{array} } \right) \\ & = [{\mathbf{x}}^{\text{R}} ]^{\text{T}} {\varvec{\Lambda}}^{\text{R}} {\mathbf{x}}^{\text{R}} + [{\mathbf{x}}^{\text{C}} ]^{\text{T}} {\varvec{\Lambda}}^{\text{C}} {\mathbf{x}}^{\text{C}} \\ & \quad + [{\mathbf{x}}_{1}^{\text{repR}} ]^{\text{T}} {\mathbf{\overset{\lower0.5em\hbox{$\smash{\scriptscriptstyle\smile}$}}{\varvec{\Lambda}}}}_{1}^{\text{repR}} {\mathbf{x}}_{1}^{\text{repR}} + \cdots + [{\mathbf{x}}_{K}^{\text{repR}} ]^{\text{T}} {\mathbf{\overset{\lower0.5em\hbox{$\smash{\scriptscriptstyle\smile}$}}{\varvec{\Lambda}}}}_{K}^{\text{repR}} {\mathbf{x}}_{K}^{\text{repR}} \\ & \quad + [{\mathbf{x}}_{1}^{\text{repC}} ]^{\text{T}} {\mathbf{\overset{\lower0.5em\hbox{$\smash{\scriptscriptstyle\smile}$}}{\varvec{\Lambda}}}}_{1}^{\text{repC}} {\mathbf{x}}_{1}^{\text{repC}} + \cdots + [{\mathbf{x}}_{L}^{\text{repC}} ]^{\text{T}} {\mathbf{\overset{\lower0.5em\hbox{$\smash{\scriptscriptstyle\smile}$}}{\varvec{\Lambda}}}}_{L}^{\text{repC}} {\mathbf{x}}_{L}^{\text{repC}} , \\ \end{aligned} . $$

where

E.30 $$ \begin{aligned}    & [{\mathbf{x}}^{{\text{R}}} ]^{{\text{T}}} {\mathbf{\Lambda }}^{{\text{R}}} {\mathbf{x}}^{{\text{R}}}  \le \max \{ \lambda _{1}^{{\text{R}}} , \ldots , \lambda _{G}^{{\text{R}}} \} |{\mathbf{x}}^{{\text{R}}} |^{2} , \\     & [{\mathbf{x}}^{{\text{C}}} ]^{{\text{T}}} {\mathbf{\Lambda }}^{{\text{C}}} {\mathbf{x}}^{{\text{C}}}  \le \max \{ \beta _{1}^{{\text{C}}} , \ldots , \beta _{H}^{{\text{C}}} \} |{\mathbf{x}}^{{\text{C}}} |^{2} \\  \end{aligned}  $$

are satisfied. In addition, from Eqs. (E.12) and (E.22) we see

E.31 $$ \begin{aligned}    & [{\mathbf{x}}_{k}^{{{\text{repR}}}} ]^{{\text{T}}} {\mathbf{\overset{\lower0.5em\hbox{$\smash{\scriptscriptstyle\smile}$}}{\Lambda } }}_{k}^{{{\text{repR}}}} {\mathbf{x}}_{k}^{{{\text{repR}}}}  \le \frac{{\lambda _{k}^{{{\text{repR}}}} }}{2}{\mathbf{|x}}_{k}^{{{\text{repR}}}} |^{2} , \\     & [{\mathbf{x}}_{l}^{{{\text{repC}}}} ]^{{\text{T}}} {\mathbf{\overset{\lower0.5em\hbox{$\smash{\scriptscriptstyle\smile}$}}{\Lambda } }}_{l}^{{{\text{repC}}}} {\mathbf{x}}_{l}^{{{\text{repC}}}}  \le \frac{{\beta _{l}^{{{\text{repC}}}} }}{2}{\mathbf{|x}}_{l}^{{{\text{repC}}}} |^{2}  \\  \end{aligned}  $$

for $k = 1, \ldots ,K$ and $l = 1, \ldots ,L$. Thus, substituting Eqs. (E.30) and (E.31) into Eq. (E.29) gives

E.32 $$ \begin{aligned} {\mathbf{x}}^{\text{T}} {\mathbf{Ax}} & \le \hbox{max} \{ \lambda_{1}^{\text{R}} , \ldots ,\lambda_{G}^{\text{R}} \} \left| {{\mathbf{x}}^{\text{R}} } \right|^{2} + \hbox{max} \{ \beta_{1}^{\text{C}} , \ldots ,\beta_{H}^{\text{C}} \} \left| {{\mathbf{x}}^{\text{C}} } \right|^{2} \\ & \quad + \frac{{\lambda_{1}^{\text{repR}} }}{2}\left| {{\mathbf{x}}_{1}^{\text{repR}} } \right|^{2} + \cdots + \frac{{\lambda_{K}^{\text{repR}} }}{2}\left|
{{\mathbf{x}}_{K}^{\text{repR}} }
\right|^{2} \\ & \quad + \frac{{\beta_{1}^{\rm repC} }}{2}\left| {{\mathbf{x}}_{1}^{\text{repC}} }
\right|^{2} + \cdots
+ \frac{{\beta_{L}^{\text{repC}} }}{2}\left| {{\mathbf{x}}_{L}^{\text{repC}} } \right|^{2} , \\ & \le \overset{\lower0.5em\hbox{$\smash{\scriptscriptstyle\smile}$}}{\lambda }_{\rm max} \left[ {\left| {{\mathbf{x}}^{\text{R}} } \right|^{2} + \left| {{\mathbf{x}}^{\text{C}} } \right|^{2} + \left| {{\mathbf{x}}_{1}^{\text{repR}} } \right|^{2} + \cdots + \left| {{\mathbf{x}}_{K}^{\text{repR}} } \right|^{2} + \left| {{\mathbf{x}}_{1}^{\text{repC}} } \right|^{2} + \cdots + \left| {{\mathbf{x}}_{L}^{\text{repC}} } \right|^{2} } \right] \\ & = \overset{\lower0.5em\hbox{$\smash{\scriptscriptstyle\smile}$}}{\lambda }_{\rm max} \left| {\mathbf{x}} \right|^{2} \\ \end{aligned} . $$

with

E.33 $$ \overset{\lower0.5em\hbox{$\smash{\scriptscriptstyle\smile}$}}{\lambda }_{\rm max} : = \hbox{max} \left\{ {\lambda_{1}^{\text{R}} , \ldots ,\lambda_{G}^{\text{R}} ,\beta_{1}^{\text{C}} , \ldots ,\beta_{H}^{\text{C}} ,\frac{{\lambda_{1}^{\text{repR}} }}{2}, \ldots ,\frac{{\lambda_{K}^{\text{repR}} }}{2},\frac{{\beta_{1}^{\text{repC}} }}{2}, \ldots ,\frac{{\beta_{L}^{\text{repC}} }}{2}} \right\} , $$

which is identical to $$ \lambda_{\rm max} $$ in Lemma 4.□

## Appendix F: Derivation of Eq. (5.4a)

### F.1 Adjustment of equilibrium point

As introduced in the main text just before Eq. (5.2a), $$ {\mathbf{m}} $$ is expressed as $$ {\mathbf{m}} = \left( {\begin{array}{*{20}c} {{\mathbf{m}}_{\text{x}} } \\ {{\mathbf{m}}_{\text{y}} } \\ \end{array} } \right) $$ with $$ {\mathbf{m}}_{\text{x}} = (m_{1} , \ldots ,m_{L} )^{\text{T}} $$ and $$ {\mathbf{m}}_{\text{y}} = (m_{L + 1} , \ldots ,m_{M} )^{\text{T}} $$ with $$| {{\mathbf{\overset{\lower0.5em\hbox{$\smash{\scriptscriptstyle\frown}$}}{m} }}_{\text{y}} }| =| {(\overset{\lower0.5em\hbox{$\smash{\scriptscriptstyle\frown}$}}{m}_{L + 1} , \ldots ,\overset{\lower0.5em\hbox{$\smash{\scriptscriptstyle\frown}$}}{m}_{M} )}| \le \rho_{m} \varepsilon $$ for all $$ i = L + 1, \ldots ,M $$. First, we transform

F.1 $$ {\mathbf{f}}({\mathbf{s}}_{\text{a}} ;{\mathbf{s}}_{\text{a}} ;{\mathbf{m}}) = {\mathbf{B}}({\mathbf{m}} - {\mathbf{\overset{\lower0.5em\hbox{$\smash{\scriptscriptstyle\frown}$}}{m} }}) + {\mathbf{r}}_{{{\text{f}}\,}} \left| {{\mathbf{m}} - {\mathbf{\overset{\lower0.5em\hbox{$\smash{\scriptscriptstyle\frown}$}}{m} }}} \right|^{2} + \varepsilon {\mathbf{h}}_{\text{f}} $$

by adjusting the perturbation term to get an equilibrium not at $$ {\mathbf{m}} = {\mathbf{\overset{\lower0.5em\hbox{$\smash{\scriptscriptstyle\frown}$}}{m} }} = \left( {\begin{array}{*{20}c} {{\mathbf{\overset{\lower0.5em\hbox{$\smash{\scriptscriptstyle\frown}$}}{m} }}_{\text{x}} } \\ {{\mathbf{\overset{\lower0.5em\hbox{$\smash{\scriptscriptstyle\frown}$}}{m} }}_{\text{y}} } \\ \end{array} } \right) $$ but at $$ {\mathbf{m}} = {\overset{\lower0.5em\hbox{$\smash{\scriptscriptstyle\frown}$}}{\tilde{\mathbf{m}} }} = \left( {\begin{array}{*{20}c} {{\mathbf{\overset{\lower0.5em\hbox{$\smash{\scriptscriptstyle\frown}$}}{m} }}_{\text{x}} } \\ {\mathbf{0}} \\ \end{array} } \right) $$ when $$ \varepsilon = 0 $$. This is done by the transformations

F.2 $$ \begin{aligned} {\mathbf{B}}({\mathbf{m}} -{{\overset{\lower0.5em\hbox{$\smash{\scriptscriptstyle\frown}$}}{{\mathbf{m}}}}}) & = {\mathbf{B}}\left( {\begin{array}{*{20}c}{{\mathbf{m}}_{\text{x}} -{{\overset{\lower0.5em\hbox{$\smash{\scriptscriptstyle\frown}$}}{{\mathbf{m}}}}}_{\text{x}} } \\ {{\mathbf{m}}_{\text{y}} -{{\overset{\lower0.5em\hbox{$\smash{\scriptscriptstyle\frown}$}}{{\mathbf{m}}}}}_{\text{y}} } \\ \end{array} } \right) = {\mathbf{B}}\left({\begin{array}{*{20}c} {{\mathbf{m}}_{\text{x}} -{{\overset{\lower0.5em\hbox{$\smash{\scriptscriptstyle\frown}$}}{{\mathbf{m}}}}}_{\text{x}} } \\ {{\mathbf{m}}_{\text{y}} } \\ \end{array} }\right) - {\mathbf{B}}\left( {\begin{array}{*{20}c} 0 \\ {{{\overset{\lower0.5em\hbox{$\smash{\scriptscriptstyle\frown}$}}{{\mathbf{m}}}}}_{\text{y}} } \\ \end{array} } \right) \\ & = {\mathbf{B}}\left({\mathbf{m}} -{{\overset{\lower0.5em\hbox{$\smash{\scriptscriptstyle\frown}$}}{\tilde{{\mathbf{m}}}}}} \right) - \varepsilon \left[ {{\mathbf{B}}\left({\begin{array}{*{20}c} 0 \\{\frac{{{{\overset{\lower0.5em\hbox{$\smash{\scriptscriptstyle\frown}$}}{{\mathbf{m}}}}}_{\text{y}} }}{\varepsilon }} \\ \end{array} } \right)} \right] \\\end{aligned} $$

and

F.3 $$ \begin{aligned} {\mathbf{r}}_{{{\text{f}}\,}} \left| {{\mathbf{m}} - {{\overset{\lower0.5em\hbox{$\smash{\scriptscriptstyle\frown}$}}{{\mathbf{m}}} }}} \right|^{2} & = {\mathbf{r}}_{{{\text{f}}\,}} \left| {{\mathbf{m}} - {{\overset{\lower0.5em\hbox{$\smash{\scriptscriptstyle\frown}$}}{\tilde{{\mathbf{m}}}} }}} \right|^{2} + {\mathbf{r}}_{{{\text{f}}\,}} \left[ {\left| {{\mathbf{m}} - {{\overset{\lower0.5em\hbox{$\smash{\scriptscriptstyle\frown}$}}{{\mathbf{m}}} }}} \right|^{2} - \left| {{\mathbf{m}} - {\overset{\lower0.5em\hbox{$\smash{\scriptscriptstyle\frown}$}}{\tilde{\mathbf{m}} }}} \right|^{2} } \right] \\ & = {\mathbf{r}}_{{{\text{f}}\,}} \left| {{\mathbf{m}} - {\overset{\lower0.5em\hbox{$\smash{\scriptscriptstyle\frown}$}}{\tilde{\mathbf{m}} }}} \right|^{2} + {\mathbf{r}}_{{{\text{f}}\,}} \left[ {\left| {{\mathbf{m}} - {\mathbf{\overset{\lower0.5em\hbox{$\smash{\scriptscriptstyle\frown}$}}{m} }}} \right|^{2} - \left| {({\mathbf{m}} - {\mathbf{\overset{\lower0.5em\hbox{$\smash{\scriptscriptstyle\frown}$}}{m} }}) + ({\mathbf{\overset{\lower0.5em\hbox{$\smash{\scriptscriptstyle\frown}$}}{m} }} - {\overset{\lower0.5em\hbox{$\smash{\scriptscriptstyle\frown}$}}{\tilde{\mathbf{m}} }})} \right|^{2} } \right] \\ & = {\mathbf{r}}_{{{\text{f}}\,}} \left| {{\mathbf{m}} - {\overset{\lower0.5em\hbox{$\smash{\scriptscriptstyle\frown}$}}{\tilde{\mathbf{m}} }}} \right|^{2} + {\mathbf{r}}_{{{\text{f}}\,}} \left[ \left( {{\mathbf{m}} - {\mathbf{\overset{\lower0.5em\hbox{$\smash{\scriptscriptstyle\frown}$}}{m} }}} \right)^{\text{T}} \left( {{\mathbf{m}} - {\mathbf{\overset{\lower0.5em\hbox{$\smash{\scriptscriptstyle\frown}$}}{m} }}} \right) \right. \\ &\quad \left. - [({\mathbf{m}} - {\mathbf{\overset{\lower0.5em\hbox{$\smash{\scriptscriptstyle\frown}$}}{m} }}) + ({\mathbf{\overset{\lower0.5em\hbox{$\smash{\scriptscriptstyle\frown}$}}{m} }} - {\overset{\lower0.5em\hbox{$\smash{\scriptscriptstyle\frown}$}}{\tilde{\mathbf{m}} }})]^{\text{T}} [({\mathbf{m}}  - {\mathbf{\overset{\lower0.5em\hbox{$\smash{\scriptscriptstyle\frown}$}}{m} }}) + ({\mathbf{\overset{\lower0.5em\hbox{$\smash{\scriptscriptstyle\frown}$}}{m} }} - {\overset{\lower0.5em\hbox{$\smash{\scriptscriptstyle\frown}$}}{\tilde{\mathbf{m}} }})] \right] \\ & = {\mathbf{r}}_{{{\text{f}}\,}} \left| {{\mathbf{m}} - {\overset{\lower0.5em\hbox{$\smash{\scriptscriptstyle\frown}$}}{\tilde{\mathbf{m}} }}} \right|^{2} + {\mathbf{r}}_{{{\text{f}}\,}} \left[ { - 2\left( {{\mathbf{m}} - {\mathbf{\overset{\lower0.5em\hbox{$\smash{\scriptscriptstyle\frown}$}}{m} }}} \right)^{\text{T}} ({\mathbf{\overset{\lower0.5em\hbox{$\smash{\scriptscriptstyle\frown}$}}{m} }} - {\overset{\lower0.5em\hbox{$\smash{\scriptscriptstyle\frown}$}}{\tilde{\mathbf{m}} }}) - ({\mathbf{\overset{\lower0.5em\hbox{$\smash{\scriptscriptstyle\frown}$}}{m} }} - {\overset{\lower0.5em\hbox{$\smash{\scriptscriptstyle\frown}$}}{\tilde{\mathbf{m}} }})^{\text{T}} ({\mathbf{\overset{\lower0.5em\hbox{$\smash{\scriptscriptstyle\frown}$}}{m} }} - {\overset{\lower0.5em\hbox{$\smash{\scriptscriptstyle\frown}$}}{\tilde{\mathbf{m}} }})} \right] \\ & = {\mathbf{r}}_{{{\text{f}}\,}} \left| {{\mathbf{m}} - {\overset{\lower0.5em\hbox{$\smash{\scriptscriptstyle\frown}$}}{\tilde{\mathbf{m}} }}} \right|^{2} + {\mathbf{r}}_{{{\text{f}}\,}} \left[ { - 2\left( {{\mathbf{m}} - {\mathbf{\overset{\lower0.5em\hbox{$\smash{\scriptscriptstyle\frown}$}}{m} }}} \right) - ({\mathbf{\overset{\lower0.5em\hbox{$\smash{\scriptscriptstyle\frown}$}}{m} }} - {\overset{\lower0.5em\hbox{$\smash{\scriptscriptstyle\frown}$}}{\tilde{\mathbf{m}} }})} \right]^{\text{T}} ({\mathbf{\overset{\lower0.5em\hbox{$\smash{\scriptscriptstyle\frown}$}}{m} }} - {\overset{\lower0.5em\hbox{$\smash{\scriptscriptstyle\frown}$}}{\tilde{\mathbf{m}} }}) \\ & = {\mathbf{r}}_{{{\text{f}}\,}} \left| {{\mathbf{m}} - {\overset{\lower0.5em\hbox{$\smash{\scriptscriptstyle\frown}$}}{\tilde{\mathbf{m}} }}} \right|^{2} + {\mathbf{r}}_{{{\text{f}}\,}} \left[ { - 2\left( {{\mathbf{m}} - {\mathbf{\overset{\lower0.5em\hbox{$\smash{\scriptscriptstyle\frown}$}}{m} }}} \right) - \left( {\begin{array}{*{20}c} {\mathbf{0}} \\ {{\mathbf{\overset{\lower0.5em\hbox{$\smash{\scriptscriptstyle\frown}$}}{m} }}_{\text{y}} } \\ \end{array} } \right)} \right]^{\text{T}} \left( {\begin{array}{*{20}c} {\mathbf{0}} \\ {{\mathbf{\overset{\lower0.5em\hbox{$\smash{\scriptscriptstyle\frown}$}}{m} }}_{\text{y}} } \\ \end{array} } \right), \\ \end{aligned} $$

which upon substitution into Eq. (F.1) gives

F.4 $$ {\mathbf{f}}({\mathbf{s}}_{\text{a}} ;{\mathbf{s}}_{\text{a}} ;{\mathbf{m}}) = {\mathbf{B}}\left( {{\mathbf{m}} - {\overset{\lower0.5em\hbox{$\smash{\scriptscriptstyle\frown}$}}{\tilde{\mathbf{m}} }}} \right) + {\tilde{\mathbf{r}}}_{{{\text{f}}\,}} \left| {{\mathbf{m}} - {\overset{\lower0.5em\hbox{$\smash{\scriptscriptstyle\frown}$}}{\tilde{\mathbf{m}} }}} \right|^{2} + \varepsilon {\tilde{\mathbf{h}}}_{\text{f}} , $$

where

F.5 $$ \begin{aligned} {\tilde{\mathbf{r}}}_{{{\text{f}}\,}} & = {\mathbf{r}}_{{{\text{f}}\,}} , \\ {\tilde{\mathbf{h}}}_{\text{f}} & = {\mathbf{h}}_{\text{f}} - {\mathbf{B}}\left( {\begin{array}{*{20}c} 0 \\ {\frac{{{\mathbf{\overset{\lower0.5em\hbox{$\smash{\scriptscriptstyle\frown}$}}{m} }}_{\text{y}} }}{\varepsilon }} \\ \end{array} } \right) + \frac{1}{\varepsilon }{\mathbf{r}}_{{{\text{f}}\,}} \left[ { - 2\left( {{\mathbf{m}} - {\mathbf{\overset{\lower0.5em\hbox{$\smash{\scriptscriptstyle\frown}$}}{m} }}} \right) - \left( {\begin{array}{*{20}c} {\mathbf{0}} \\ {{\mathbf{\overset{\lower0.5em\hbox{$\smash{\scriptscriptstyle\frown}$}}{m} }}_{\text{y}} }
\\ \end{array} } \right)}
\right]^{\text{T}} \left( {\begin{array}{*{20}c}
{\mathbf{0}} \\ {{\mathbf{\overset{\lower0.5em\hbox{$\smash{\scriptscriptstyle\frown}$}}{m} }}_{\text{y}} } \\ \end{array} } \right), \\ \end{aligned} $$

and

F.6 $$ \begin{aligned} \left| {{\tilde{\mathbf{r}}}_{\text{f}} } \right| & = \left| {{\mathbf{r}}_{\text{f}} } \right| \le C_{{{\mathbf{r}}{\text{f}}}} = :\tilde{C}_{{{\mathbf{r}}{\text{f}}}} , \\ \left| {{\tilde{\mathbf{h}}}_{\text{f}} } \right| & \le \left| {{\mathbf{h}}_{\text{f}} } \right| + \left\| {\mathbf{B}} \right\|\left| {\frac{{{\mathbf{\overset{\lower0.5em\hbox{$\smash{\scriptscriptstyle\frown}$}}{m} }}_{\text{y}} }}{\varepsilon }} \right| + \frac{1}{\varepsilon }\left| {{\mathbf{r}}_{{{\text{f}}\,}} } \right|\left[ {2\left| {{\mathbf{m}}_{\text{y}} - {\mathbf{\overset{\lower0.5em\hbox{$\smash{\scriptscriptstyle\frown}$}}{m} }}_{\text{y}} } \right| + \left| {{\mathbf{\overset{\lower0.5em\hbox{$\smash{\scriptscriptstyle\frown}$}}{m} }}_{\text{y}} } \right|} \right]\left| {{\mathbf{\overset{\lower0.5em\hbox{$\smash{\scriptscriptstyle\frown}$}}{m} }}_{\text{y}} } \right| \\ & \le \left| {{\mathbf{h}}_{\text{f}} } \right| + \left\| {\mathbf{B}} \right\|\rho_{\text{m}} + \frac{1}{\varepsilon }\left| {{\mathbf{r}}_{{{\text{f}}\,}} } \right|\left[ {2\eta + \varepsilon \rho_{\text{m}} } \right]\varepsilon \rho_{\text{m}} \\ & \le C_{{{\mathbf{h}}{\text{f}}}} + \left\| {\mathbf{B}} \right\|\rho_{\text{m}} + C_{{{\mathbf{r}}{\text{f}}}} \left[ {2\eta + \varepsilon \rho_{\text{m}} } \right]\rho_{\text{m}} = :\tilde{C}_{{{\mathbf{h}}{\text{f}}}} . \\ \end{aligned} $$

Substituting Eq. (E.4) into Eq. (4.2) in the main text gives

F.7 $$ \frac{{{\text{d}}{\mathbf{m}}}}{{{\text{d}}t}} = {\text{diag}}({\mathbf{m}})\left[ {{\mathbf{B}}({\mathbf{m}} - {\overset{\lower0.5em\hbox{$\smash{\scriptscriptstyle\frown}$}}{\tilde{\mathbf{m}} }}) + {\tilde{\mathbf{r}}}_{{{\text{f}}\,}} \left| {{\mathbf{m}} - {\overset{\lower0.5em\hbox{$\smash{\scriptscriptstyle\frown}$}}{\tilde{\mathbf{m}} }}} \right|^{2} + \varepsilon {\tilde{\mathbf{h}}}_{\text{f}} } \right] . $$

### F.2 Decomposition into not-small and small population densities

We decompose Eq. (F.7) into equations for the $L$ phenotypes with larger population densities ${\mathbf{m}}_{\text{x}} = (m_{1} , \ldots ,m_{L} )^{\text{T}}$ and the other $K = M - L$ phenotypes with small population densities ${\mathbf{m}}_{\text{y}} = (m_{L + 1} , \ldots ,m_{M} )^{\text{T}}$,

F.8 $$ \frac{{{\text{d}}{\mathbf{m}}_{{\mathbf{x}}} }}{{{\text{d}}t}} = {\text{diag(}}{\mathbf{m}}_{\text{x}} )\left[ {{\mathbf{B}}_{\text{xx}} ({\mathbf{m}}_{\text{x}} - {\mathbf{\overset{\lower0.5em\hbox{$\smash{\scriptscriptstyle\frown}$}}{m} }}_{\text{x}} ) + {\mathbf{B}}_{\text{xy}} {\mathbf{m}}_{\text{y}} + {\tilde{\mathbf{r}}}_{{{\text{f}}{\mathbf{x}}}} \left| {{\mathbf{m}} - {\overset{\lower0.5em\hbox{$\smash{\scriptscriptstyle\frown}$}}{\tilde{\mathbf{m}} }}} \right|^{2} + \varepsilon {\tilde{\mathbf{h}}}_{{{\text{f}}{\mathbf{x}}}} } \right] , $$

F.9 $$ \frac{{{\text{d}}{\mathbf{m}}_{\text{y}} }}{{{\text{d}}t}} = {\text{diag(}}{\mathbf{m}}_{\text{y}} )\left[ {{\mathbf{B}}_{\text{yx}} ({\mathbf{m}}_{\text{x}} - {\mathbf{\overset{\lower0.5em\hbox{$\smash{\scriptscriptstyle\frown}$}}{m} }}_{\text{x}} ) + {\mathbf{B}}_{\text{yy}} {\mathbf{m}}_{\text{y}} + {\tilde{\mathbf{r}}}_{{{\text{fy}}\,}} \left| {{\mathbf{m}} - {\overset{\lower0.5em\hbox{$\smash{\scriptscriptstyle\frown}$}}{\tilde{\mathbf{m}} }}} \right|^{2} + \varepsilon {\tilde{\mathbf{h}}}_{\text{fy}} } \right] , $$

where $$ {\mathbf{m}} = \left( {\begin{array}{*{20}c} {{\mathbf{m}}_{\text{x}} } \\ {{\mathbf{m}}_{\text{y}} } \\ \end{array} } \right) $$, $$ {\tilde{\mathbf{r}}}_{\text{x}} = (\tilde{r}_{1} , \ldots ,\tilde{r}_{L} )^{\text{T}} $$, $$ {\tilde{\mathbf{r}}}_{\text{y}} = (\tilde{r}_{L + 1} , \ldots ,\tilde{r}_{M} )^{\text{T}} $$, $$ {\tilde{\mathbf{h}}}_{\text{x}} = (\tilde{h}_{1} , \ldots ,\tilde{h}_{L} )^{\text{T}} $$, $$ {\tilde{\mathbf{h}}}_{\text{y}} = (\tilde{h}_{L + 1} , \ldots ,\tilde{h}_{M} )^{\text{T}} $$, and

F.10 $$ {\mathbf{B}}_{\text{xx}} = \left( {\begin{array}{*{20}c} {b_{1,1} } & \ldots & {b_{1,L} } \\ \ldots & \ldots & \ldots \\ {b_{L,1} } & \ldots & {b_{L,L} } \\ \end{array} } \right),\quad {\mathbf{B}}_{\text{xy}} = \left( {\begin{array}{*{20}c} {b_{1,L + 1} } & \ldots & {b_{1,M} } \\ \ldots & \ldots & \ldots \\ {b_{L,L + 1} } & \ldots & {b_{L,M} } \\ \end{array} } \right). $$

F.11 $$ {\mathbf{B}}_{\text{yx}} = \left( {\begin{array}{*{20}c} {b_{L + 1,1} } & \ldots & {b_{L + 1,L} } \\ \ldots & \ldots & \ldots \\ {b_{M,1} } & \ldots & {b_{M,L} } \\ \end{array} } \right),\quad {\mathbf{B}}_{\text{yy}} = \left( {\begin{array}{*{20}c} {b_{L + 1,L + 1} } & \ldots & {b_{L + 1,M} } \\ \ldots & \ldots & \ldots \\ {b_{M,L + 1} } & \ldots & {b_{M,M} } \\ \end{array} } \right). $$

### F.3 Variable transformation

If $\left| {{\mathbf{m}} - {\tilde{\mathbf{m}}}} \right|$ is sufficiently small, so that the nonlinear term in Eq. (F.8) can be neglected, and $\varepsilon = 0$, then $\frac{{{\text{d}}{\mathbf{m}}_{\text{x}} }}{{{\text{d}}t}} = 0$ equals

F.12 $$ {\mathbf{B}}_{\text{xx}} ({\mathbf{m}}_{\text{x}} - {\mathbf{\overset{\lower0.5em\hbox{$\smash{\scriptscriptstyle\frown}$}}{m} }}_{\text{x}} ) + {\mathbf{B}}_{\text{xy}} {\mathbf{m}}_{\text{y}} = 0 , $$

which gives an approximate slow manifold defined by Eq. (5.3b) in the main text,

F.13 $$ {\tilde{\mathbf{m}}}_{\text{x}} ({\mathbf{m}}_{\text{y}} ) = {\mathbf{\overset{\lower0.5em\hbox{$\smash{\scriptscriptstyle\frown}$}}{m} }}_{\text{x}} - {\mathbf{Tm}}_{\text{y}} $$

with ${\mathbf{T}}: = {\mathbf{B}}_{\text{xx}}^{ - 1} {\mathbf{B}}_{\text{xy}}$. Even when $\varepsilon > 0$, the dynamics is expected to be effectively characterized by its projection on this manifold. Thus, we introduce ${\mathbf{x}} = {\mathbf{P}}({\mathbf{m}}_{\text{x}} - {\tilde{\mathbf{m}}}_{\text{x}} ({\mathbf{m}}_{\text{y}} ))$ and ${\mathbf{y}} = {\mathbf{m}}_{\text{y}}$, to capture the fast convergence to the manifold and slow dynamics along it, respectively. The vector $$ {\mathbf{w}} = \left( \begin{aligned} {\mathbf{x}} \hfill \\ {\mathbf{y}} \hfill \\ \end{aligned} \right) $$ is written as

F.14 $$ {\mathbf{w}} = \left( \begin{aligned} {\mathbf{x}} \hfill \\ {\mathbf{y}} \hfill \\ \end{aligned} \right) = \left( \begin{aligned} {\mathbf{P}}({\mathbf{m}}_{\text{x}} - {\mathbf{\overset{\lower0.5em\hbox{$\smash{\scriptscriptstyle\frown}$}}{m} }}_{\text{x}} + {\mathbf{Tm}}_{\text{y}} ) \hfill \\ {\mathbf{m}}_{\text{y}} \hfill \\ \end{aligned} \right) = {\mathbf{Q}}\left( \begin{aligned} {\mathbf{m}}_{\text{x}} - {\mathbf{\overset{\lower0.5em\hbox{$\smash{\scriptscriptstyle\frown}$}}{m} }}_{\text{x}} \hfill \\ {\mathbf{m}}_{\text{y}} \hfill \\ \end{aligned} \right) = {\mathbf{Q}}\left( {{\mathbf{m}} - {\tilde{\mathbf{m}}}} \right) , $$

with

F.15 $$ \begin{aligned} {\mathbf{Q}} & : = \left( {\begin{array}{cc} {\mathbf{P}} & {{\mathbf{PT}}} \\ 0 & {{\mathbf{I}}_{\text{y}} } \\ \end{array} } \right), \\ {\mathbf{Q}}^{ - 1} & = \left( {\begin{array}{cc} {{\mathbf{P}}^{ - 1} } & { - {\mathbf{T}}} \\ 0 & {{\mathbf{I}}_{\text{y}} } \\ \end{array} } \right), \\ \end{aligned} $$

where ${\mathbf{I}}_{\text{y}}$ is the $K \times K$ identity matrix. Then, by Eq. (F.7),

F.16 $$ \begin{aligned} \frac{{{\text{d}}{\mathbf{w}}}}{{{\text{d}}t}} & = {\mathbf{Q}}\frac{{{\text{d}}{\mathbf{m}}}}{{{\text{d}}t}} \\ & = {\mathbf{Q}}{\text{diag(}}{\mathbf{m}} )\left[ {{\mathbf{B}}({\mathbf{m}} - {\overset{\lower0.5em\hbox{$\smash{\scriptscriptstyle\frown}$}}{\tilde{\mathbf{m}} }}) + {\tilde{\mathbf{r}}}_{\text{f}} \left| {{\mathbf{m}} - {\overset{\lower0.5em\hbox{$\smash{\scriptscriptstyle\frown}$}}{\tilde{\mathbf{m}} }}} \right|^{2} + \varepsilon {\tilde{\mathbf{h}}}_{\text{f}} } \right] \\ & = {\mathbf{Q}}{\text{diag(}}{\mathbf{m}} ){\mathbf{B}}({\mathbf{m}} - {\overset{\lower0.5em\hbox{$\smash{\scriptscriptstyle\frown}$}}{\tilde{\mathbf{m}} }}) + {\mathbf{Q}}{\text{diag(}}{\mathbf{m}} ){\tilde{\mathbf{r}}}_{\text{f}} \left| {{\mathbf{m}} - {\overset{\lower0.5em\hbox{$\smash{\scriptscriptstyle\frown}$}}{\tilde{\mathbf{m}} }}} \right|^{2} + \varepsilon {\mathbf{Q}}{\text{diag(}}{\mathbf{m}} ){\tilde{\mathbf{h}}}_{\text{f}} . \\ \end{aligned} $$

${\mathbf{Q}}{\text{diag(}}{\mathbf{m}} )$ is further transformed as

F.17 $$ \begin{aligned} {\mathbf{Q}}{\text{diag(}}{\mathbf{m}} )& = \left( {\begin{array}{cc} {\mathbf{P}} & {{\mathbf{PT}}} \\ 0 & {{\mathbf{I}}_{\text{y}} } \\ \end{array} } \right)\left( {\begin{array}{cc} {{\text{diag}}({\mathbf{\overset{\lower0.5em\hbox{$\smash{\scriptscriptstyle\frown}$}}{m} }}_{\text{x}} ) + {\text{diag}}({\mathbf{m}}_{\text{x}} - {\mathbf{\overset{\lower0.5em\hbox{$\smash{\scriptscriptstyle\frown}$}}{m} }}_{\text{x}} )} & 0 \\ 0 & {{\text{diag}}({\mathbf{m}}_{\text{y}} )} \\ \end{array} } \right) \\ & = \left( {\begin{array}{cc} {\mathbf{P}} & 0 \\ 0 & {{\mathbf{I}}_{\text{y}} } \\ \end{array} } \right)\left( {\begin{array}{*{20}c} {{\text{diag}}({\mathbf{\overset{\lower0.5em\hbox{$\smash{\scriptscriptstyle\frown}$}}{m} }}_{\text{x}} )} & 0 \\ 0 & {{\text{diag}}({\mathbf{m}}_{\text{y}} )} \\ \end{array} } \right) + \left( {\begin{array}{*{20}c} {\mathbf{P}} & {{\mathbf{PT}}} \\ 0 & 0 \\ \end{array} } \right)\left( {\begin{array}{cc} {{\text{diag}}({\mathbf{m}}_{\text{x}} - {\mathbf{\overset{\lower0.5em\hbox{$\smash{\scriptscriptstyle\frown}$}}{m} }}_{\text{x}} )} & 0 \\ 0 & {{\text{diag}}({\mathbf{m}}_{\text{y}} )} \\ \end{array} } \right) \\ & = \left( {\begin{array}{*{20}c} {\mathbf{P}} & 0 \\ 0 & {{\mathbf{I}}_{\text{y}} } \\ \end{array} } \right)\left( {\begin{array}{cc} {{\text{diag}}({\mathbf{\overset{\lower0.5em\hbox{$\smash{\scriptscriptstyle\frown}$}}{m} }}_{\text{x}} )} & 0 \\ 0 & {{\text{diag}}({\mathbf{m}}_{\text{y}} )} \\ \end{array} } \right) + \left( {\begin{array}{cc} {\mathbf{P}} & {{\mathbf{PT}}} \\ 0 & 0 \\ \end{array} } \right){\text{diag}}({\mathbf{m}} - {\tilde{\mathbf{m}}}). \\ \end{aligned} $$

Substituting Eq. (F.17) into Eq. (F.16) gives

F.18 $$ \begin{aligned} \frac{{{\text{d}}{\mathbf{w}}}}{{{\text{d}}t}} & = \left[ {\left( {\begin{array}{*{20}c} {\mathbf{P}} & 0 \\ 0 & {{\mathbf{I}}_{\text{y}} } \\ \end{array} } \right)\left( {\begin{array}{*{20}c} {{\text{diag}}({\mathbf{\overset{\lower0.5em\hbox{$\smash{\scriptscriptstyle\frown}$}}{m} }}_{\text{x}} )} & 0 \\ 0 & {{\text{diag}}({\mathbf{m}}_{\text{y}} )} \\ \end{array} } \right) + \left( {\begin{array}{*{20}c} {\mathbf{P}} & {{\mathbf{PT}}} \\ 0 & 0 \\ \end{array} } \right){\text{diag}}({\mathbf{m}} - {\overset{\lower0.5em\hbox{$\smash{\scriptscriptstyle\frown}$}}{\tilde{\mathbf{m}} }})} \right]{\mathbf{B}}({\mathbf{m}} - {\overset{\lower0.5em\hbox{$\smash{\scriptscriptstyle\frown}$}}{\tilde{\mathbf{m}} }}) \\ & \quad + \,{\mathbf{Q}}{\text{diag}}({\mathbf{m}}){\tilde{\mathbf{r}}}_{\text{f}} \left| {{\mathbf{m}} - {\overset{\lower0.5em\hbox{$\smash{\scriptscriptstyle\frown}$}}{\tilde{\mathbf{m}} }}} \right|^{2} + \varepsilon {\mathbf{Q}}{\text{diag}}({\mathbf{m}}){\tilde{\mathbf{h}}}_{\text{f}} \\ & = \left( {\begin{array}{*{20}c} {{\mathbf{P}}{\text{diag}}({\mathbf{\overset{\lower0.5em\hbox{$\smash{\scriptscriptstyle\frown}$}}{m} }}_{\text{x}} )} & 0 \\ 0 & {{\text{diag}}({\mathbf{m}}_{\text{y}} )} \\ \end{array} } \right){\mathbf{BQ}}^{ - 1} {\mathbf{w}} \\ & \quad + \,\left[ {\left( {\begin{array}{*{20}c} {\mathbf{P}} & {{\mathbf{PT}}} \\ 0 & 0 \\ \end{array} } \right){\text{diag}}({\mathbf{m}} - {\overset{\lower0.5em\hbox{$\smash{\scriptscriptstyle\frown}$}}{\tilde{\mathbf{m}} }}){\mathbf{B}}({\mathbf{m}} - {\overset{\lower0.5em\hbox{$\smash{\scriptscriptstyle\frown}$}}{\tilde{\mathbf{m}} }}) + {\mathbf{Q}}{\text{diag}}({\mathbf{m}}){\tilde{\mathbf{r}}}_{\text{f}} \left| {{\mathbf{m}} - {\overset{\lower0.5em\hbox{$\smash{\scriptscriptstyle\frown}$}}{\tilde{\mathbf{m}} }}} \right|^{2} } \right] + \varepsilon {\mathbf{Q}}{\text{diag}}({\mathbf{m}}){\tilde{\mathbf{h}}}_{\text{f}} \\ & = \left( {\begin{array}{*{20}c} {{\mathbf{P}}{\text{diag}}({\mathbf{\overset{\lower0.5em\hbox{$\smash{\scriptscriptstyle\frown}$}}{m} }}_{\text{x}} )} & 0 \\ 0 & {{\text{diag}}({\mathbf{m}}_{\text{y}} )} \\ \end{array} } \right){\mathbf{BQ}}^{ - 1} {\mathbf{w}} + {\mathbf{r}}^{*} \left| {\mathbf{w}} \right|^{2} + \varepsilon {\mathbf{h}}^{*} , \\ \end{aligned} $$

with

F.19 $$ \begin{aligned} {\mathbf{r}}^{*} & : = \frac{1}{{\left| {\mathbf{w}} \right|^{2} }}\left[ {\left( {\begin{array}{cc} {\mathbf{P}} & {{\mathbf{PT}}} \\ 0 & 0 \\ \end{array} } \right){\text{diag}}({\mathbf{m}} - {\overset{\lower0.5em\hbox{$\smash{\scriptscriptstyle\frown}$}}{\tilde{\mathbf{m}} }}){\mathbf{B}}({\mathbf{m}} - {\overset{\lower0.5em\hbox{$\smash{\scriptscriptstyle\frown}$}}{\tilde{\mathbf{m}} }}) + {\mathbf{Q}}{\text{diag}}({\mathbf{m}}){\tilde{\mathbf{r}}}_{\text{f}} \left| {{\mathbf{m}} - {{\overset{\lower0.5em\hbox{$\smash{\scriptscriptstyle\frown}$}}{\tilde{\mathbf{m}}} }}} \right|^{2} } \right], \\ {\mathbf{h}}^{*} & : = {\mathbf{Q}}{\text{diag}}({\mathbf{m}}){\tilde{\mathbf{h}}}_{\text{f}} , \\ \end{aligned} $$

where ${\mathbf{r}}^{*}$ is worked out below after the derivation of the equation for ${\text{d}}{\mathbf{w}}/{\text{d}}t$, and estimated in Appendix F.4. ${\mathbf{BQ}}^{ - 1}$ is transformed by using ${\mathbf{T}} = {\mathbf{B}}_{\text{xx}}^{ - 1} {\mathbf{B}}_{\text{xy}}$, as

F.20 $$ \begin{aligned} {\mathbf{BQ}}^{ - 1} & = \left( {\begin{array}{cc} {{\mathbf{B}}_{\text{xx}} } & {{\mathbf{B}}_{\text{xy}} } \\ {{\mathbf{B}}_{\text{yx}} } & {{\mathbf{B}}_{\text{yy}} } \\ \end{array} } \right)\left( {\begin{array}{cc} {{\mathbf{P}}^{ - 1} } & { - {\mathbf{T}}} \\ 0 & {{\mathbf{I}}_{\text{y}} } \\ \end{array} } \right) \\ & = \left( {\begin{array}{cc} {{\mathbf{B}}_{\text{xx}} {\mathbf{P}}^{ - 1} } & { - {\mathbf{B}}_{\text{xx}} {\mathbf{T}} + {\mathbf{B}}_{\text{xy}} } \\ {{\mathbf{B}}_{\text{yx}} {\mathbf{P}}^{ - 1} } & { - {\mathbf{B}}_{\text{yx}} {\mathbf{T}} + {\mathbf{B}}_{\text{yy}} } \\ \end{array} } \right) \\ & = \left( {\begin{array}{cc} {{\mathbf{B}}_{\text{xx}} {\mathbf{P}}^{ - 1} } & 0 \\ {{\mathbf{B}}_{\text{yx}} {\mathbf{P}}^{ - 1} } & { - {\mathbf{B}}_{\text{yx}} {\mathbf{B}}_{\text{xx}}^{ - 1} {\mathbf{B}}_{\text{yy}} + {\mathbf{B}}_{\text{yy}} } \\ \end{array} } \right) = \left( {\begin{array}{cc} {{\mathbf{B}}_{\text{xx}} {\mathbf{P}}^{ - 1} } & 0 \\ {\mathbf{U}} & {{\mathbf{J}}_{\text{y}} } \\ \end{array} } \right), \\ \end{aligned} $$

with ${\mathbf{U}} = {\mathbf{B}}_{\text{yx}} {\mathbf{P}}^{ - 1}  \, \rm and \, {\mathbf{J}}_{\text{y}} = - {\mathbf{B}}_{\text{yx}} {\mathbf{B}}_{\text{xx}}^{ - 1} {\mathbf{B}}_{\text{yy}} + {\mathbf{B}}_{\text{yy}}$. Eq. (F.20) transforms the first term of the last line of Eq. (F.18) into

F.21 $$ \begin{aligned} & \left( {\begin{array}{*{20}c} {{\mathbf{P}}{\text{diag}}({\mathbf{\overset{\lower0.5em\hbox{$\smash{\scriptscriptstyle\frown}$}}{m} }}_{\text{x}} )} & 0 \\ 0 & {{\text{diag}}({\mathbf{y}})} \\ \end{array} } \right){\mathbf{BQ}}^{ - 1} {\mathbf{w}} \\ & \quad = \left( {\begin{array}{*{20}c} {{\mathbf{P}}{\text{diag}}({\mathbf{\overset{\lower0.5em\hbox{$\smash{\scriptscriptstyle\frown}$}}{m} }}_{\text{x}} )} & 0 \\ 0 & {{\text{diag}}({\mathbf{y}})} \\ \end{array} } \right)\left( {\begin{array}{*{20}c} {{\mathbf{B}}_{\text{xx}} {\mathbf{P}}^{ - 1} } & 0 \\ {\mathbf{U}} & {\mathbf{J}} \\ \end{array} } \right){\mathbf{w}} \\ & \quad = \left( {\begin{array}{*{20}c} {{\mathbf{P}}{\text{diag}}({\mathbf{\overset{\lower0.5em\hbox{$\smash{\scriptscriptstyle\frown}$}}{m} }}_{\text{x}} ){\mathbf{B}}_{\text{xx}} {\mathbf{P}}^{ - 1} } & 0 \\ {{\text{diag}}({\mathbf{y}}){\mathbf{U}}} & {{\text{diag}}({\mathbf{y}}){\mathbf{J}}} \\ \end{array} } \right){\mathbf{w}} \\ & \quad = \left( {\begin{array}{*{20}c} {{\mathbf{A}}_{\text{x}} } & 0 \\ {{\text{diag}}({\mathbf{y}}){\mathbf{U}}} & {{\text{diag}}({\mathbf{y}}){\mathbf{J}}} \\ \end{array} } \right){\mathbf{w}} \\ & \quad = \left( {\begin{array}{*{20}c} {{\mathbf{A}}_{\text{x}} {\mathbf{x}}} \\ {{\text{diag}}({\mathbf{y}})\left[ {{\mathbf{Ux}} + {\mathbf{J}}_{\text{y}} {\mathbf{y}}} \right]} \\ \end{array} } \right), \\ \end{aligned} $$

with $$ {\mathbf{A}}_{\text{x}} = {\mathbf{P}}{\text{diag}}({\mathbf{\overset{\lower0.5em\hbox{$\smash{\scriptscriptstyle\frown}$}}{m} }}_{\text{x}} ){\mathbf{B}}_{\text{xx}} {\mathbf{P}}^{ - 1} $$. Therefore, Eq. (F.18) is transformed into

F.22 $$ \frac{\text{d}}{{{\text{d}}t}}\left( {\begin{array}{*{20}c} {\mathbf{x}} \\ {\mathbf{y}} \\ \end{array} } \right) = \left( {\begin{array}{*{20}c} {{\mathbf{A}}_{\text{x}} {\mathbf{x}}} \\ {{\text{diag}}({\mathbf{y}})\left[ {{\mathbf{Ux}} + {\mathbf{J}}_{\text{y}} {\mathbf{y}}} \right]} \\ \end{array} } \right) + {\mathbf{r}}^{*} \left| {\mathbf{w}} \right|^{2} + \varepsilon {\mathbf{h}}^{*} . $$

In addition, ${\mathbf{r}}^{*}$ and ${\mathbf{h}}^{*}$ given in Eq. (F.19) are further transformed into

F.23 $$ \begin{aligned} {\mathbf{r}}^{*} & = \frac{1}{{\left| {\mathbf{w}} \right|^{2} }}\left[ {\left( {\begin{array}{*{20}c} {\mathbf{P}} & {{\mathbf{PT}}} \\ 0 & 0 \\ \end{array} } \right){\text{diag}}({\mathbf{m}} - {\overset{\lower0.5em\hbox{$\smash{\scriptscriptstyle\frown}$}}{\tilde{\mathbf{m}} }}){\mathbf{B}}({\mathbf{m}} - {\overset{\lower0.5em\hbox{$\smash{\scriptscriptstyle\frown}$}}{\tilde{\mathbf{m}} }}) + \left( {\begin{array}{*{20}c} {\mathbf{P}} & {{\mathbf{PT}}} \\ 0 & {{\mathbf{I}}_{\text{y}} } \\ \end{array} } \right){\text{diag}}({\mathbf{m}}){\tilde{\mathbf{r}}}_{\text{f}} \left| {{\mathbf{m}} - {\overset{\lower0.5em\hbox{$\smash{\scriptscriptstyle\frown}$}}{\tilde{\mathbf{m}} }}} \right|^{2} } \right] \\ & = \frac{1}{{\left| {\mathbf{w}} \right|^{2} }}\left( {\begin{array}{*{20}c} {\left( {\begin{array}{*{20}c} {\mathbf{P}} & {{\mathbf{PT}}} \\ \end{array} } \right){\text{diag}}({\mathbf{m}} - {\overset{\lower0.5em\hbox{$\smash{\scriptscriptstyle\frown}$}}{\tilde{\mathbf{m}} }}){\mathbf{B}}({\mathbf{m}} - {\overset{\lower0.5em\hbox{$\smash{\scriptscriptstyle\frown}$}}{\tilde{\mathbf{m}} }}) + \left( {\begin{array}{*{20}c} {\mathbf{P}} & {{\mathbf{PT}}} \\ \end{array} } \right){\text{diag}}({\mathbf{m}}){\tilde{\mathbf{r}}}_{{{\text{f}}\,}} \left| {{\mathbf{m}} - {\overset{\lower0.5em\hbox{$\smash{\scriptscriptstyle\frown}$}}{\tilde{\mathbf{m}} }}} \right|^{2} } \\ {\left( {\begin{array}{*{20}c} {\mathbf{0}} & {{\mathbf{I}}_{\text{y}} } \\ \end{array} } \right){\text{diag}}({\mathbf{m}}){\tilde{\mathbf{r}}}_{\text{f}} \left| {{\mathbf{m}} - {\overset{\lower0.5em\hbox{$\smash{\scriptscriptstyle\frown}$}}{\tilde{\mathbf{m}} }}} \right|^{2} } \\ \end{array} } \right) \\ & = \left( {\begin{array}{*{20}c} {\frac{1}{{\left| {\mathbf{w}} \right|^{2} }}\left( {\begin{array}{*{20}c} {\mathbf{P}} & {{\mathbf{PT}}} \\ \end{array} } \right)\left[ {{\text{diag}}({\mathbf{m}} - {\overset{\lower0.5em\hbox{$\smash{\scriptscriptstyle\frown}$}}{\tilde{\mathbf{m}} }}){\mathbf{B}}({\mathbf{m}} - {\overset{\lower0.5em\hbox{$\smash{\scriptscriptstyle\frown}$}}{\tilde{\mathbf{m}} }}) + {\text{diag}}({\mathbf{m}}){\tilde{\mathbf{r}}}_{{{\text{f}}\,}} \left| {{\mathbf{m}} - {\overset{\lower0.5em\hbox{$\smash{\scriptscriptstyle\frown}$}}{\tilde{\mathbf{m}} }}} \right|^{2} } \right]} \\ {{\text{diag}}({\mathbf{y}})\frac{1}{{\left| {\mathbf{w}} \right|^{2} }}{\tilde{\mathbf{r}}}_{\text{fy}} \left| {{\mathbf{m}} - {{\overset{\lower0.5em\hbox{$\smash{\scriptscriptstyle\frown}$}}{\tilde{\mathbf{m}}} }}} \right|^{2} } \\ \end{array} } \right) \\ & = :\left( {\begin{array}{*{20}c} {{\tilde{\mathbf{r}}}_{\text{x}} } \\ {{\text{diag}}({\mathbf{y}}){\tilde{\mathbf{r}}}_{\text{y}} } \\ \end{array} } \right), \\ \end{aligned} $$

F.24 $$ \begin{aligned} {\mathbf{h}}^{*} & = \left( {\begin{array}{*{20}c} {\mathbf{P}} & {{\mathbf{PT}}} \\ 0 & {{\mathbf{I}}_{\text{y}} } \\ \end{array} } \right){\text{diag}}({\mathbf{m}}){\tilde{\mathbf{h}}}_{\text{f}} = \left( {\begin{array}{*{20}c} {\left( {\begin{array}{*{20}c} {\mathbf{P}} & {{\mathbf{PT}}} \\ \end{array} } \right){\text{diag}}({\mathbf{m}}){\tilde{\mathbf{h}}}_{\text{f}} } \\ {\left( {\begin{array}{*{20}c} {\mathbf{0}} & {{\mathbf{I}}_{\text{y}} } \\ \end{array} } \right){\text{diag}}({\mathbf{m}}){\tilde{\mathbf{h}}}_{\text{f}} } \\ \end{array} } \right) = \left( {\begin{array}{*{20}c} {\left( {\begin{array}{*{20}c} {\mathbf{P}} & {{\mathbf{PT}}} \\ \end{array} } \right){\text{diag}}({\mathbf{m}}){\tilde{\mathbf{h}}}_{\text{f}} } \\ {{\text{diag}}({\mathbf{y}}){\tilde{\mathbf{h}}}_{\text{fy}} } \\ \end{array} } \right) \\ & = :\left( {\begin{array}{*{20}c} {{\tilde{\mathbf{h}}}_{\text{x}} } \\ {{\text{diag}}({\mathbf{y}}){\tilde{\mathbf{h}}}_{\text{y}} } \\ \end{array} } \right), \\ \end{aligned} $$

with

F.25 $$ \begin{aligned} {\tilde{\mathbf{r}}}_{\text{x}} & = \frac{1}{{\left| {\mathbf{w}} \right|^{2} }}\left( {\begin{array}{*{20}c} {\mathbf{P}} & {{\mathbf{PT}}} \\ \end{array} } \right)\left[ {{\text{diag}}({\mathbf{m}} - {\overset{\lower0.5em\hbox{$\smash{\scriptscriptstyle\frown}$}}{\tilde{\mathbf{m}} }}){\mathbf{B}}({\mathbf{m}} - {\overset{\lower0.5em\hbox{$\smash{\scriptscriptstyle\frown}$}}{\tilde{\mathbf{m}} }}) + {\text{diag}}({\mathbf{m}}){\tilde{\mathbf{r}}}_{{{\text{f}}\,}} \left| {{\mathbf{m}} - {\overset{\lower0.5em\hbox{$\smash{\scriptscriptstyle\frown}$}}{\tilde{\mathbf{m}} }}} \right|^{2} } \right], \\ {\tilde{\mathbf{r}}}_{\text{y}} & = \frac{1}{{\left| {\mathbf{w}} \right|^{2} }}{\tilde{\mathbf{r}}}_{\text{fy}} \left| {{\mathbf{m}} - {\overset{\lower0.5em\hbox{$\smash{\scriptscriptstyle\frown}$}}{\tilde{\mathbf{m}} }}} \right|^{2} , \\ {\tilde{\mathbf{h}}}_{\text{x}} & = \left( {\begin{array}{*{20}c} {\mathbf{P}} & {{\mathbf{PT}}} \\ \end{array} } \right){\text{diag}}({\mathbf{m}}){\tilde{\mathbf{h}}}_{\text{f}} , \\ {\tilde{\mathbf{h}}}_{\text{y}} & = {\tilde{\mathbf{h}}}_{\text{fy}}. \\ \end{aligned}. $$

Substituting Eqs. (F.23) and (F.24) into Eq. (F.22) gives Eq. (5.4a) in the main text,

F.26 $$ \begin{aligned} \frac{\text{d}}{{{\text{d}}t}}\left( {\begin{array}{*{20}c} {\mathbf{x}} \\ {\mathbf{y}} \\ \end{array} } \right) & = \left( {\begin{array}{*{20}c} {{\mathbf{A}}_{\text{x}} {\mathbf{x}}} \\ {{\text{diag}}({\mathbf{y}})\left[ {{\mathbf{Ux}} + {\mathbf{J}}_{\text{y}} {\mathbf{y}}} \right]} \\ \end{array} } \right) + \left( {\begin{array}{*{20}c} {{\tilde{\mathbf{r}}}_{\text{x}} } \\ {{\text{diag}}({\mathbf{y}}){\tilde{\mathbf{r}}}_{\text{y}} } \\ \end{array} } \right)\left| {\mathbf{w}} \right|^{2} + \varepsilon \left( {\begin{array}{*{20}c} {{\tilde{\mathbf{h}}}_{\text{x}} } \\ {{\text{diag}}({\mathbf{y}}){\tilde{\mathbf{h}}}_{\text{y}} } \\ \end{array} } \right) \\ & = \left( {\begin{array}{*{20}c} {{\mathbf{A}}_{\text{x}} {\mathbf{x}} + \varepsilon {\tilde{\mathbf{h}}}_{\text{x}} + {\tilde{\mathbf{r}}}_{\text{x}} \left| {\mathbf{w}} \right|^{2} } \\ {{\text{diag}}({\mathbf{y}})\left[ {{\mathbf{J}}_{\text{y}} {\mathbf{y}} + {\mathbf{Ux}} + \varepsilon {\tilde{\mathbf{h}}}_{\text{y}} + {\tilde{\mathbf{r}}}_{\text{y}} \left| {\mathbf{w}} \right|^{2} } \right]} \\ \end{array} } \right). \\ \end{aligned} $$

### F.4 Finding $\tilde{C}_{{{\mathbf{rx}}}} ,\tilde{C}_{{{\mathbf{ry}}}} ,\tilde{C}_{{{\mathbf{hx}}}} , \, {\rm and}\, {\tilde{C}}_{{{\mathbf{hy}}}}$

By using the following relationships,

F.27 $$ \begin{aligned} \frac{{\left| {{\mathbf{m}} - {\overset{\lower0.5em\hbox{$\smash{\scriptscriptstyle\frown}$}}{\tilde{\mathbf{m}} }}} \right|}}{{\left| {\mathbf{w}} \right|}} & \le \frac{{\left| {{\mathbf{Q}}^{ - 1} {\mathbf{w}}} \right|}}{{\left| {\mathbf{w}} \right|}} = \left\| {{\mathbf{Q}}^{ - 1} } \right\|, \\ \frac{{\left\| {{\text{diag}}({\mathbf{m}} - {\overset{\lower0.5em\hbox{$\smash{\scriptscriptstyle\frown}$}}{\tilde{\mathbf{m}} }})} \right\|}}{{\left| {\mathbf{w}} \right|}} & \le \frac{{\max_{i} \left( {\left| {m_{i} - \overset{\lower0.5em\hbox{$\smash{\scriptscriptstyle\frown}$}}{\tilde{m}}_{i} } \right|} \right)}}{{\left| {\mathbf{w}} \right|}} = \frac{{\left| {{\mathbf{m}} - {\overset{\lower0.5em\hbox{$\smash{\scriptscriptstyle\frown}$}}{\tilde{\mathbf{m}} }}} \right|}}{{\left| {\mathbf{w}} \right|}}\frac{{\max_{i} \left( {\left| {m_{i} - \overset{\lower0.5em\hbox{$\smash{\scriptscriptstyle\frown}$}}{\tilde{m}}_{i} } \right|} \right)}}{{\left| {{\mathbf{m}} - {\overset{\lower0.5em\hbox{$\smash{\scriptscriptstyle\frown}$}}{\tilde{\mathbf{m}} }}} \right|}} \\ & \le \left\| {{\mathbf{Q}}^{ - 1} } \right\|\sqrt {\frac{{\max_{i} \left( {\left| {m_{i} - \overset{\lower0.5em\hbox{$\smash{\scriptscriptstyle\frown}$}}{\tilde{m}}_{i} } \right|^{2} } \right)}}{{\sum\nolimits_{i = 1}^{M} {\left| {m_{i} - \overset{\lower0.5em\hbox{$\smash{\scriptscriptstyle\frown}$}}{\tilde{m}}_{i} } \right|^{2} } }}} \le \left\| {{\mathbf{Q}}^{ - 1} } \right\|, \\ \end{aligned} $$

the following formulas for $\tilde{C}_{{{\mathbf{rx}}}} ,\tilde{C}_{{{\mathbf{ry}}}} ,\tilde{C}_{{{\mathbf{hx}}}} , \, {\rm and}\, {\tilde{C}}_{{{\mathbf{hy}}}}$ are found,

F.28 $$ \begin{aligned} \left| {{\tilde{\mathbf{r}}}_{\text{x}} } \right| & = \left| {\left( {\begin{array}{*{20}c} {\mathbf{P}} & {{\mathbf{PT}}} \\ \end{array} } \right)\left[ {\frac{{{\text{diag}}({\mathbf{m}} - {\overset{\lower0.5em\hbox{$\smash{\scriptscriptstyle\frown}$}}{\tilde{\mathbf{m}} }})}}{{\left| {\mathbf{w}} \right|}}{\mathbf{B}}\frac{{({\mathbf{m}} - {\overset{\lower0.5em\hbox{$\smash{\scriptscriptstyle\frown}$}}{\tilde{\mathbf{m}} }})}}{{\left| {\mathbf{w}} \right|}} + {\text{diag}}({\mathbf{m}}){\tilde{\mathbf{r}}}_{{{\text{f}}\,}} \frac{{\left| {{\mathbf{m}} - {\overset{\lower0.5em\hbox{$\smash{\scriptscriptstyle\frown}$}}{\tilde{\mathbf{m}} }}} \right|^{2} }}{{\left| {\mathbf{w}} \right|^{2} }}} \right]} \right| \\ & \le \left( {\left\| {\mathbf{P}} \right\| + \left\| {{\mathbf{PT}}} \right\|} \right)\left[ {\left\| {{\mathbf{Q}}^{ - 1} } \right\|\left\| {\mathbf{B}} \right\|\left\| {{\mathbf{Q}}^{ - 1} } \right\| + \mathop {\hbox{max} }\limits_{i} (m_{i} )\tilde{C}_{{{\mathbf{r}}{\text{f}}}} \left\| {{\mathbf{Q}}^{ - 1} } \right\|^{2} } \right] \\ & \le \left\| {\mathbf{P}} \right\|\left( {1 + \left\| {\mathbf{T}} \right\|} \right)\left\| {{\mathbf{Q}}^{ - 1} } \right\|^{2} \left[ {\left\| {\mathbf{B}} \right\| + \eta \tilde{C}_{{{\mathbf{r}}{\text{f}}}} } \right] = :\tilde{C}_{{{\mathbf{r}}{\text{x}}}} , \\ \end{aligned} $$

F.29 $$ \left| {{\tilde{\mathbf{r}}}_{\text{y}} } \right| \le \left| {{\tilde{\mathbf{r}}}_{\text{fy}} } \right|\frac{{\left| {{\mathbf{m}} - {\overset{\lower0.5em\hbox{$\smash{\scriptscriptstyle\frown}$}}{\tilde{\mathbf{m}} }}} \right|^{2} }}{{\left| {\mathbf{w}} \right|^{2} }} \le \tilde{C}_{{{\mathbf{r}}{\text{f}}}} \left\| {{\mathbf{Q}}^{ - 1} } \right\|^{2} = :\tilde{C}_{{{\mathbf{r}}{\text{y}}}} , $$

F.30 $$ \left| {{\tilde{\mathbf{h}}}_{\text{x}} } \right| \le \left\| {\mathbf{P}} \right\|\left( {1 + \left\| {\mathbf{T}} \right\|} \right)\eta \tilde{C}_{{{\mathbf{h}}{\text{f}}}} = :\tilde{C}_{{{\mathbf{h}}{\text{x}}}} , $$

F.31 $$ \left| {{\tilde{\mathbf{h}}}_{\text{y}} } \right| = \left| {{\tilde{\mathbf{h}}}_{\text{fy}} } \right| \le \tilde{C}_{{{\mathbf{h}}{\text{f}}}} = :\tilde{C}_{{{\mathbf{h}}{\text{y}}}} . $$

### F.5 Finding $\tilde{C}_{{\mathbf{r}}} \, {\rm{and}}\, \tilde{C}_{{\mathbf{h}}}$

From Eq. (5.6b) in the main text and Eqs. (F.28) to (F.31), we see

F.32 $$ \begin{aligned} \left| {{\tilde{\mathbf{r}}}} \right| & = \left| {\left( {\begin{array}{*{20}c} {{\tilde{\mathbf{r}}}_{{\mathbf{x}}} } \\ {d{\tilde{\mathbf{r}}}_{{\mathbf{y}}} } \\ \end{array} } \right)} \right| = \sqrt {\left| {{\tilde{\mathbf{r}}}_{{\mathbf{x}}} } \right|^{2} + d^{2} \left| {{\tilde{\mathbf{r}}}_{{\mathbf{y}}} } \right|^{2} } \le \sqrt {\tilde{C}_{{{\mathbf{rx}}}}^{2} + d^{2} \tilde{C}_{{{\mathbf{ry}}}}^{2} } = :\tilde{C}_{{\mathbf{r}}} , \\ \left| {{\tilde{\mathbf{h}}}} \right| & = \left| {\left( {\begin{array}{*{20}c} {{\tilde{\mathbf{h}}}_{{\mathbf{x}}} } \\ {d{\tilde{\mathbf{h}}}_{{\mathbf{y}}} } \\ \end{array} } \right)} \right| = \sqrt {\left| {{\tilde{\mathbf{h}}}_{{\mathbf{x}}} } \right|^{2} + d^{2} \left| {{\tilde{\mathbf{h}}}_{{\mathbf{y}}} } \right|^{2} } \le \sqrt {\tilde{C}_{{{\mathbf{hx}}}}^{2} + d^{2} \tilde{C}_{{{\mathbf{hy}}}}^{2} } = :\tilde{C}_{{\mathbf{h}}} . \\ \end{aligned} $$

## Appendix G: Proof of Lemma 9

We denote the eigenvalues of ${\mathbf{J}}_{\text{x}}$ and $\frac{1}{2}\left( {{\mathbf{J}}_{\text{y}} + {\mathbf{J}}_{\text{y}}^{\text{T}} } \right)$ by $\lambda_{1} , \ldots ,\lambda_{L}$ and $\lambda_{L + 1} , \ldots ,\lambda_{M}$, respecitively. We prove Lemma 9 by showing that for $\lambda_{{{\text{x\,max}}}} : = \max_{i = 1, \ldots ,L} \left( {\text{Re} (\lambda_{i} )} \right) < 0$, $\lambda_{{{\text{y\,max}}}} : = \max_{i = L + 1, \ldots ,M} \left( {\text{Re} (\lambda_{i} )} \right) < 0$, and a sufficiently small $d$, $\frac{1}{2}\left[ {{\tilde{\mathbf{A}}}^{\text{T}} + {\tilde{\mathbf{A}}}} \right]$ is negative definite,

G.1 $$ \begin{aligned} {\mathbf{w}}^{\text{T}} {\tilde{\mathbf{A}}\mathbf{w}} & = \left( {\begin{array}{*{20}c} {\mathbf{x}} \\ {\mathbf{y}} \\ \end{array} } \right)^{\text{T}} \left( {\begin{array}{*{20}c} {{\mathbf{A}}_{\text{x}} } & 0 \\ {d{\mathbf{U}}} & {d{\mathbf{J}}_{\text{y}} } \\ \end{array} } \right)\left( {\begin{array}{*{20}c} {\mathbf{x}} \\ {\mathbf{y}} \\ \end{array} } \right) \\ & = {\mathbf{x}}^{\text{T}} {\mathbf{A}}_{\text{x}} {\mathbf{x}} + d{\mathbf{y}}^{\text{T}} {\mathbf{Ux}} + d{\mathbf{y}}^{\text{T}} {\mathbf{J}}_{\text{y}} {\mathbf{y}} \\ & = {\mathbf{x}}^{\text{T}} {\mathbf{A}}_{\text{x}} {\mathbf{x}} + d{\mathbf{y}}^{\text{T}} {\mathbf{Ux}} + d{\mathbf{y}}^{\text{T}} \frac{1}{2}\left( {{\mathbf{J}}_{\text{y}} + {\mathbf{J}}_{\text{y}}^{\text{T}} } \right){\mathbf{y}} \\ & \le \lambda_{{{\text{x\,max}}}} \left| {\mathbf{x}} \right|^{2} + d\left\| {\mathbf{U}} \right\|\left| {\mathbf{x}} \right|\left| {\mathbf{y}} \right| + d\lambda_{{{\text{y\,max}}}} \left| {\mathbf{y}} \right|^{2} \\ & = \lambda_{{{\text{x\,max}}}} \left[ {\left| {\mathbf{x}} \right|^{2} + 2\frac{{d\left\| {\mathbf{U}} \right\|\left| {\mathbf{x}} \right|\left| {\mathbf{y}} \right|}}{{2\lambda_{{{\text{x\,max}}}} }} + \frac{{d^{2} \left\| {\mathbf{U}} \right\|^{2} \left| {\mathbf{y}} \right|^{2} }}{{4\lambda^{2}_{{{\text{x\,max}}}} }}} \right] - \frac{{d^{2} \left\| {\mathbf{U}} \right\|^{2} \left| {\mathbf{y}} \right|^{2} }}{{4\lambda_{{{\text{x\,max}}}} }} + d\lambda_{{{\text{y\,max}}}} \left| {\mathbf{y}} \right|^{2} \\ & = \lambda_{{{\text{x\,max}}}} \left[ {\left| {\mathbf{x}} \right| + \frac{{d\left\| {\mathbf{U}} \right\|\left| {\mathbf{y}} \right|}}{{2\lambda_{{{\text{x\,max}}}} }}} \right]^{2} - \frac{{d^{2} \left\| {\mathbf{U}} \right\|^{2} \left| {\mathbf{y}} \right|^{2} }}{{4\lambda_{{{\text{x\,max}}}} }} + d\lambda_{{{\text{y\,max}}}} \left| {\mathbf{y}} \right|^{2} \\ & = \lambda_{{{\text{x\,max}}}} \left[ {\left| {\mathbf{x}} \right| + \frac{{d\left\| {\mathbf{U}} \right\|\left| {\mathbf{y}} \right|}}{{2\lambda_{{{\text{x\,max}}}} }}} \right]^{2} + \frac{{d\left| {\mathbf{y}} \right|^{2} \left\| {\mathbf{U}} \right\|^{2} }}{{4\lambda_{{{\text{x\,max}}}} }}\left[ {\frac{{4\lambda_{{{\text{x\,max}}}} \lambda_{{{\text{y\,max}}}} }}{{\left\| {\mathbf{U}} \right\|^{2} }} - d} \right]. \\ \end{aligned} $$

Thus, ${\mathbf{w}}^{\text{T}} {\tilde{\mathbf{A}}\mathbf{w}}$ is always negative for any ${\mathbf{w}}$, under $\lambda_{x\hbox{max} } < 0$, $\lambda_{y\hbox{max} } < 0$, and a sufficiently small $d$ so that

G.2 $$ d < \frac{{4\lambda_{x\hbox{max} } \lambda_{y\hbox{max} } }}{{\left\| {\mathbf{U}} \right\|^{2} }} . $$

In this case, all eigenvalues of $\frac{1}{2}\left[ {{\tilde{\mathbf{A}}}^{\text{T}} + {\tilde{\mathbf{A}}}} \right]$ are negative. □

## Appendix H: Derivation of Eq. (5.8c)

Here we derive Eq. (5.8c) in the main text. As explained in the main text, the contour curve $V = V_{0}$ has an inscribed circle $\left| {\mathbf{w}} \right| = \tilde{\phi }_{\text{h}}$, and a circumscribed circle $\left| {\mathbf{w}} \right| = \alpha \tilde{\phi }_{\text{h}}$. Specifically, the contour curve is defined by

H.1 $$ E_{\text{V}} = \left\{ {\left. {{\mathbf{w}} = (x_{1} , \ldots ,x_{L} ,y_{1} , \ldots ,y_{K} )^{\text{T}} } \right|\sum\limits_{i = 1}^{L} {x_{i}^{2} } + 2d\sum\limits_{j = 1}^{K} {y_{j} } = V_{0} } \right\} , $$

where $V_{0}$ is determined so that $\left| {\mathbf{w}} \right| = \tilde{\phi }_{\text{h}}$ is an inscribed circle of $V = V_{0}$, i.e.,

H.2 $$ \tilde{\phi }_{\text{h}}^{ 2} = \mathop {\hbox{min} }\limits_{{{\mathbf{w}} \in E_{\text{V}} }} \left( {\left| {\mathbf{w}} \right|^{2} } \right) . $$

For convenience, we write ${\mathbf{y}}$ for ${\mathbf{y}} = (y_{1} , \ldots ,y_{K} )^{\text{T}}$, which is identical to ${\mathbf{y}} = (y_{L + 1} , \ldots ,y_{M} )^{\text{T}}$ in the other appendices and in the main text. On the other hand, the radius of the circumscribed circle, $\alpha \tilde{\phi }_{\text{h}}$, satisfies

H.3 $$ \alpha^{2} \tilde{\phi }_{\text{h}}^{ 2} = \mathop {\hbox{max} }\limits_{{{\mathbf{w}} \in E_{\text{V}} }} \left( {\left| {\mathbf{w}} \right|^{2} } \right) . $$

Thus, $\alpha$ is given by

H.4 $$ \alpha = \sqrt {\frac{{\alpha^{2} \tilde{\phi }_{\text{h}}^{ 2} }}{{\tilde{\phi }_{\text{h}}^{ 2} }}} = \sqrt {\frac{{\max_{{{\mathbf{w}} \in E_{\text{V}} }} \left( {\left| {\mathbf{w}} \right|^{2} } \right)}}{{\min_{{{\mathbf{w}} \in E_{\text{V}} }} \left( {\left| {\mathbf{w}} \right|^{2} } \right)}}} . $$

To calculate the maximum and minimum of $\left| {\mathbf{w}} \right|^{2}$ for ${\mathbf{w}} \in E_{\text{V}}$ we proceed as follows. To make calculation easier, we decompose the expression of $E_{\text{V}}$ as $E_{\text{V}} = \left\{ {\left. {\mathbf{w}} \right|\,\,{\mathbf{x}} \in E_{\text{Vx}} ,\,\,{\mathbf{y}} \in E_{\text{Vy}} ,\,\,q \in [0,1]} \right\}$, with

H.5 $$ E_{\text{Vx}} = \left\{ { {\mathbf{x}} \big|\sum\limits_{i = 1}^{L} {x_{i}^{2} } = (1 - q)V_{0} } \right\}, $$

H.6 $$ E_{\text{Vy}} = \left\{ { {\mathbf{y}} \big|2d\sum\limits_{j = 1}^{K} {y_{j} } = qV_{0} ,\,\,y_{j} \ge 0\;\;{\text{for}}\;{\text{all }}j = 1, \ldots ,K} \right\} . $$

Then, $\min_{{{\mathbf{w}} \in E_{\text{V}} }} \left( {\left| {\mathbf{w}} \right|^{2} } \right)$ and $\max_{{{\mathbf{w}} \in E_{\text{V}} }} \left( {\left| {\mathbf{w}} \right|^{2} } \right)$ are expressed as

H.7 $$ \mathop { \hbox{min} }\limits_{{{\mathbf{w}} \in E_{\text{V}} }} \left( {\left| {\mathbf{w}} \right|^{2} } \right) = \mathop { \hbox{min} }\limits_{q \in [0,1]} \left( {\mathop { \hbox{min} }\limits_{{{\mathbf{x}} \in E_{\text{Vx}} }} \left( {\left| {\mathbf{x}} \right|^{2} } \right) + \mathop { \hbox{min} }\limits_{{{\mathbf{y}} \in E_{\text{Vy}} }} \left( {\left| {\mathbf{y}} \right|^{2} } \right)} \right) , $$

H.8 $$ \mathop { \hbox{max} }\limits_{{{\mathbf{w}} \in E_{\text{V}} }} \left( {\left| {\mathbf{w}} \right|^{2} } \right) = \mathop { \hbox{max} }\limits_{q \in [0,1]} \left( {\mathop { \hbox{max} }\limits_{{{\mathbf{x}} \in E_{\text{Vx}} }} \left( {\left| {\mathbf{x}} \right|^{2} } \right) + \mathop { \hbox{max} }\limits_{{{\mathbf{y}} \in E_{\text{Vy}} }} \left( {\left| {\mathbf{y}} \right|^{2} } \right)} \right) . $$

Before performing the extremisations, we first work out the various expressions for a given value of $q$. We start with the ${\mathbf{x}}$-part of ${\mathbf{w}}$. Clearly,

H.9 $$ \mathop { \hbox{min} }\limits_{{{\mathbf{x}} \in E_{\text{Vx}} }} \left( {\left| {\mathbf{x}} \right|^{2} } \right) = \mathop { \hbox{max} }\limits_{{{\mathbf{x}} \in E_{\text{Vx}} }} \left( {\left| {\mathbf{x}} \right|^{2} } \right) = (1 - q)V_{0} . $$

For the calculation of $\max_{{{\mathbf{y}} \in E_{\text{Vy}} }} \left( {\left| {\mathbf{y}} \right|^{2} } \right)$, the $\left| {\mathbf{y}} \right|^{2}$ can be transformed by $2d\sum\nolimits_{j = 1}^{K} {y_{j} } = qV_{0}$ into

H.10 $$ \left| {\mathbf{y}} \right|^{2} = \sum\limits_{i = 1}^{K} {y_{i}^{2} } = \left[ {\sum\limits_{i = 1}^{K} {y_{i} } } \right]^{2} - 2\sum\limits_{j = 1}^{K} {\sum\limits_{k = j + 1}^{K} {y_{j} } y_{k} } = \frac{{q^{2} V_{0}^{2} }}{{4d^{2} }} - 2\sum\limits_{j = 1}^{K} {\sum\limits_{k = j + 1}^{K} {y_{j} } y_{k} } .$$

Since $y_{i} \ge 0$ for $i = 1, \ldots ,K$, $\max_{{{\mathbf{y}} \in E_{\text{Vy}} }} \left( {\left| {\mathbf{y}} \right|^{2} } \right)$ is given by

H.11 $$ \mathop { \hbox{max} }\limits_{{{\mathbf{y}} \in E_{\text{Vy}} }} \left( {\left| {\mathbf{y}} \right|^{2} } \right) = \frac{{q^{2} V_{0}^{2} }}{{4d^{2} }}, $$

which is attained when $y_{{j_{0} }} = \frac{{qV_{0} }}{2d}$ and $y_{{j \ne j_{0} }} = 0$ for some $j_{0} = 1, \ldots ,K.$

To calculate $\min_{{{\mathbf{y}} \in E_{\text{Vy}} }} \left( {\left| {\mathbf{y}} \right|^{2} } \right)$, Eq. (H.10) is further transformed into

H.12 $$ \begin{aligned} \sum\limits_{i = 1}^{K} {y_{i}^{2} } & = \left[ {\sum\limits_{i = 1}^{K} {y_{i} } } \right]^{2} - 2\sum\limits_{j = 1}^{K} {\sum\limits_{k = j + 1}^{K} {y_{j} y_{k} } } \\ & = \frac{{q^{2} V_{0}^{2} }}{{4d^{2} }} + \sum\limits_{j = 1}^{K} {\sum\limits_{k = j + 1}^{K} {(y_{j} - y_{k} )^{2} } } - \sum\limits_{j = 1}^{K} {\sum\limits_{k = j + 1}^{K} {(y_{j}^{2} + y_{k}^{2} )} } \\ & = \frac{{q^{2} V_{0}^{2} }}{{4d^{2} }} + \sum\limits_{j = 1}^{K} {\sum\limits_{k = j + 1}^{K} {(y_{j} - y_{k} )^{2} } } \\ & \quad - \,\sum\limits_{j = 1}^{K} {\sum\limits_{k = j + 1}^{K} {y_{j}^{2} } } - \sum\limits_{j = 1}^{K} {\sum\limits_{k = j + 1}^{K} {y_{k}^{2} } } \\ & = \frac{{q^{2} V_{0}^{2} }}{{4d^{2} }} + \sum\limits_{j = 1}^{K} {\sum\limits_{k = j + 1}^{K} {(y_{j} - y_{k} )^{2} } } \\ & \quad - \,\left[ {(K - 1)y_{1}^{2} + \cdots + y_{K - 1}^{2} } \right] - \left[ {(y_{2}^{2} + \cdots + y_{K}^{2} ) + \cdots + (y_{K}^{2} )} \right] \\ & = \frac{{q^{2} V_{0}^{2} }}{{4d^{2} }} + \sum\limits_{j = 1}^{K} {\sum\limits_{k = j + 1}^{K} {(y_{j} - y_{k} )^{2} } } - (K - 1)\sum\limits_{j = 1}^{K} {y_{j}^{2} } . \\ \end{aligned} $$

Solving Eq. (H.12) for $\sum\nolimits_{i = 1}^{K} {y_{i}^{2} } = \left| {\mathbf{y}} \right|^{2}$ gives

H.13 $$ \left| {\mathbf{y}} \right|^{2} = \sum\limits_{i = 1}^{K} {y_{i}^{2} } = \frac{{q^{2} V_{0}^{2} }}{{4d^{2} K}} + \frac{1}{K}\sum\limits_{j = 1}^{K} {\sum\limits_{k = j + 1}^{K} {(y_{j} - y_{k} )^{2} } } . $$

Therefore,

H.14 $$  {\mathop { \hbox{min} }\limits_{{{\mathbf{y}} \in E_{\text{Vy}} }} \left( {\left| {\mathbf{y}} \right|^{2} } \right) = \frac{{q^{2} V^{2} }}{{4d^{2} K}}} , $$

which is attained when $y_{i} = \frac{{qV_{0} }}{2dK}$ for all $i = 1, \ldots K$. Substituting Eqs. (H.9), (H.11), and (H.14) into Eqs. (H.7) and (H.8) gives

H.15 $$ \begin{aligned} \mathop { \hbox{min} }\limits_{{{\mathbf{w}} \in E_{\text{V}} }} \left( {\left| {\mathbf{w}} \right|^{2} } \right) & = \mathop { \hbox{min} }\limits_{q \in [0,1]} \left( {(1 - q)V_{0} + \frac{{q^{2} V_{0}^{2} }}{{4d^{2} K}}} \right) \\ & = \mathop { \hbox{min} }\limits_{q \in [0,1]} \left( {\frac{{V_{0}^{2} }}{{4d^{2} K}}q^{2} - V_{0} q + V_{0} } \right) \\ & = \mathop { \hbox{min} }\limits_{q \in [0,1]} \left( {\frac{{V_{0}^{2} }}{{4d^{2} K}}\left[ {q - \frac{{2d^{2} K}}{{V_{0} }}} \right]^{2} + V_{0} - Kd^{2} } \right) \\ & = \left\{ {\begin{array}{*{20}l} {V_{0} - Kd^{2} } \hfill & {{\text{for}}\;\;\frac{{2d^{2} K}}{{V_{0} }} \le 1\quad \left( {{\text{attained}}\;{\text{when}}\;q = \frac{{2d^{2} K}}{{V_{0} }}} \right)} \hfill \\ {\frac{{V_{0}^{2} }}{{4d^{2} K}}} \hfill & {{\text{for}}\;\;\frac{{2d^{2} K}}{{V_{0} }} > 1\quad \left( {{\text{attained}}\;{\text{when}}\;q = 1} \right)} \hfill \\ \end{array} } \right. \\ \end{aligned} $$

and

H.16 $$ \begin{aligned} \mathop { \hbox{max} }\limits_{{{\mathbf{w}} \in E_{\text{V}} }} \left( {\left| {\mathbf{w}} \right|^{2} } \right) & = \mathop { \hbox{max} }\limits_{q \in [0,1]} \left( {\frac{{q^{2} V_{0}^{2} }}{{4d^{2} }} + (1 - q)V_{0} } \right) \\ & = \hbox{max} \left\{ {\frac{{V_{0}^{2} }}{{4d^{2} }},V_{0} } \right\}\quad \left( {{\text{attained}}\;{\text{when}}\;q = 0\,\,{\text{or}}\,\,1} \right). \\ \end{aligned} $$

Thus, from Eqs. (H.2) and (H.15), $V_{0}$ is obtained as

H.17 $$ V_{0} = \left\{ {\begin{array}{*{20}l} {\tilde{\phi }_{\text{h}}^{ 2} + Kd^{2} } \hfill & {{\text{for}}\;\;K \le \frac{{\tilde{\phi }_{\text{h}}^{2} }}{{d^{2} }}} \hfill \\ {2d\sqrt K \tilde{\phi }_{\text{h}} } \hfill & {{\text{for}}\;\;K > \frac{{\tilde{\phi }_{\text{h}}^{2} }}{{d^{2} }}} \hfill. \\ \end{array} } \right. $$

Finally, substituting Eqs. (H.16) and (H.17) into Eq. (H.4) gives

H.18 $$ \alpha = \sqrt {\frac{{\max_{{{\mathbf{w}} \in E_{\text{V}} }} \left( {\left| {\mathbf{w}} \right|^{2} } \right)}}{{\tilde{\phi }_{\text{h}}^{ 2} }}} = \left\{ {\begin{array}{ll} 1 \hfill & {{\text{for}}\;\;K = 0} \hfill \\ {\hbox{max} \left\{ {\frac{{\tilde{\phi }_{\text{h}}^{ 2} + Kd^{2} }}{{2d\tilde{\phi }_{\text{h}} }},\frac{{\sqrt {\tilde{\phi }_{\text{h}}^{ 2} + Kd^{2} } }}{{\tilde{\phi }_{\text{h}} }}} \right\}} \hfill & {{\text{for}}\;\;0 < K \le \frac{{\tilde{\phi }_{\text{h}}^{ 2} }}{{d^{2} }}} \hfill \\ {\hbox{max} \left\{ {\sqrt K ,\frac{{\sqrt {2dK^{1/2} \tilde{\phi }_{\text{h}} } }}{{\tilde{\phi }_{\text{h}} }}} \right\}} \hfill & {{\text{for}}\;\;\frac{{\tilde{\phi }_{\text{h}}^{ 2} }}{{d^{2} }} < K,} \hfill \\ \end{array} } \right. $$

where $\max_{{{\mathbf{w}} \in E_{\text{V}} }} \left( {\left| {\mathbf{w}} \right|^{2} } \right) = \max_{{{\mathbf{w}} \in E_{\text{V}} }} \left( {\left| {\mathbf{x}} \right|^{2} } \right) = \tilde{\phi }_{\text{h}}^{ 2}$ for $K = 0$ is used.

## Appendix I: Generalization to higher-dimensional trait spaces

Throughout the manuscript, the dimensionality of the considered trait space matters only when the fitness function (or its derivatives with respect to population densities or phenotypes) is Taylor expanded, as in Eqs. (A.11), (B.10), (B.23), (C.1), and (C.8), for a non-representative phenotype $s_{j}$ for $j = M + 1, \ldots ,N + 1$, around its representative phenotype $s_{{{\text{cid}}(j)}}$. Those equations are readily extended to higher-dimensional trait spaces, by replacing those derivatives with the corresponding directional derivatives, as explained below.

We consider a trait space of arbitrary dimension $Z$, having $N$ resident phenotypes ${\mathbf{v}}_{1} , \ldots ,{\mathbf{v}}_{N}$ and a mutant ${\mathbf{v}}^{{\prime }} = {\mathbf{v}}_{N + 1}$, where the $j$th phenotype is denoted by ${\mathbf{v}}_{j} = (v_{j,1} , \ldots ,v_{j,Z} )^{\text{T}} \in {\mathbb{R}}^{Z}$. The fitness function, denoted by $F({\mathbf{v}}_{j} ;{\mathbf{V}}^{{\prime }} ;{\mathbf{n}}^{{\prime }} )$ with ${\mathbf{V}}^{{\prime }} = ({\mathbf{v}}_{1} , \ldots ,{\mathbf{v}}_{N + 1} )$ and ${\mathbf{n}}^{{\prime }} = (n_{1} , \ldots ,n_{N + 1} )^{\text{T}}$, is assumed to satisfy all axioms (i–v) in Sect. 2. Analogously to the one-dimensional case, we permute and cluster those $N + 1$ phenotypes into $M$ groups so that their representatives, i.e., approximate phenotypes, ${\mathbf{V}}_{\text{a}} = ({\mathbf{v}}_{1} , \ldots ,{\mathbf{v}}_{M} )^{\text{T}}$ satisfy $\left| {{\mathbf{v}}_{j} - {\mathbf{v}}_{{{\text{cid}}(j)}} } \right| < \varepsilon$ for all $j = M + 1, \ldots ,N + 1$ and ${\text{cid}}(j) \in \{ 1, \ldots ,M\}$. To expand $F({\mathbf{v}}_{j} ;{\mathbf{V}}^{{\prime }} ;{\mathbf{n}}^{{\prime }} )$ using directional derivatives, we introduce ${\mathbf{u}}_{j} (s_{j} )$ for $j = M + 1, \ldots ,N + 1$ as

I.1 $$ {\mathbf{u}}_{j} (s_{j} ): = {\mathbf{v}}_{{{\text{cid}}(j)}} + (s_{j} - s_{{{\text{cid}}(j)}} ){\mathbf{e}}_{{j,{\text{cid}}(j)}} , $$

with a scalar parameter $s_{j}$,

I.2 $$ {\mathbf{e}}_{{j,{\text{cid}}(j)}} : = \frac{{{\mathbf{v}}_{j} - {\mathbf{v}}_{{{\text{cid}}(j)}} }}{{\left| {{\mathbf{v}}_{j} - {\mathbf{v}}_{{{\text{cid}}(j)}} } \right|}} , $$

and $s_{{{\text{cid}}(j)}} \in \left\{ {s_{1} , \ldots ,s_{M} } \right\}$, where $s_{1} , \ldots ,s_{M}$ can be chosen arbitrarily as long as ${\mathbf{u}}_{j} (s_{j} ) = {\mathbf{v}}_{j}$ holds for $j = M + 1, \ldots ,N + 1$ (because all the expansions of the fitness function in this paper are in non-representative phenotypes that correspond to $s_{M + 1} , \ldots ,s_{N + 1}$). Notice that ${\mathbf{u}}_{j} (s_{{{\text{cid}}(j)}} ) = {\mathbf{v}}_{{{\text{cid}}(j)}}$. For notational convenience, we also introduce for $j = 1, \ldots ,M$

I.3 $$ {\mathbf{u}}_{j} (s_{j} ): = {\mathbf{v}}_{j} . $$

Then we define for $j = 1, \ldots ,N + 1$

I.4 $$ F_{j} (s_{j} ;{\mathbf{s}}^{{\prime }} ;{\mathbf{n}}^{{\prime }} ): = F({\mathbf{u}}_{j} (s_{j} );{\mathbf{U}}^{{\prime }} ({\mathbf{s}}^{{\prime }} );{\mathbf{n}}^{{\prime }} ) $$

with ${\mathbf{s}}^{{\prime }} : = (s_{1} , \ldots ,s_{N + 1} )^{\text{T}}$, ${\mathbf{u}}_{j} (s_{j} ) = {\mathbf{v}}_{j}$, and ${\mathbf{U}}^{{\prime }} ({\mathbf{s}}^{{\prime }} ) = ({\mathbf{u}}_{1} (s_{1} ), \ldots ,{\mathbf{u}}_{N + 1} (s_{N + 1} )) = {\mathbf{V}}^{{\prime }}$. Note that the $F_{j} (s_{j} ;{\mathbf{s}}^{{\prime }} ;{\mathbf{n}}^{{\prime }} )$ satisfy the smoothness axiom (i), the reducibility axiom (ii), and the bounded-world axiom (iii). The exchangeability axiom (iv) is also satisfied between $s_{j}$ and its representative phenotype $s_{{{\text{cid}}(j)}}$, for $j = M + 1, \ldots ,N + 1$ (i.e., $F_{j} (s_{j} ;{\mathbf{s}}^{{\prime }} ;{\mathbf{n}}^{{\prime }} ) = F_{{{\text{cid}}(j)}} (s_{{{\text{cid}}(j)}} ;{\mathbf{s}}^{{\prime }} ;{\mathbf{n}}^{{\prime }} )$ for $s_{j} = s_{{{\text{cid}}(j)}}$). Thus, by Taylor’s theorem, $F_{j} (s_{j} ;{\mathbf{s}}^{{\prime }} ;{\mathbf{n}}^{{\prime }} )$ for $j = M + 1, \ldots ,N + 1$ can be expanded in $s_{j}$ around $s_{{{\text{cid}}(j)}}$ as

I.5 $$ \begin{aligned} F_{j} (s_{j} ;{\mathbf{s}}^{{\prime }} ;{\mathbf{n}}^{{\prime }} ) & = \left. {F_{j} (s_{j} ;{\mathbf{s}}^{{\prime }} ;{\mathbf{n}}^{{\prime }} )} \right|_{{s_{j} = s_{{{\text{cid}}(j)}} }} + \left. {\frac{{\partial F_{j} (s_{j} ;{\mathbf{s}}^{{\prime }} ;{\mathbf{n}}^{{\prime }} )}}{{\partial s_{j} }}} \right|_{{s_{j} = s_{{j{\text{T}}}} }} (s_{j} - s_{{{\text{cid}}(j)}} ) \\ & = \left. {F_{{{\text{cid}}(j)}} (s_{j} ;{\mathbf{s}}^{{\prime }} ;{\mathbf{n}}^{{\prime }} )} \right|_{{s_{j} = s_{{{\text{cid}}(j)}} }} + \left. {\frac{{\partial F_{j} (s_{j} ;{\mathbf{s}}^{{\prime }} ;{\mathbf{n}}^{{\prime }} )}}{{\partial s_{j} }}} \right|_{{s_{j} = s_{{j{\text{T}}}} }} (s_{j} - s_{{{\text{cid}}(j)}} ) \\ \end{aligned} $$

with some appropriately chosen $$ s_{{j{\text{T}}}} \in [s_{j} ,s_{{{\text{cid}}(j)}} ] $$. The derivatives of $$ F_{j} (s_{j} ;{\mathbf{s}}^{{\prime }} ;{\mathbf{n}}^{{\prime }} ) $$ with respect to population densities or phenotypes can be expanded in the same manner. Therefore, for all $$ \cdot = i,j,k $$ replacing $$ F(s_{ \cdot } ;{\mathbf{s}}^{{\prime }} ;{\mathbf{n}}^{{\prime }} ) $$ with $$ F_{ \cdot } (s_{ \cdot } ;{\mathbf{s}}^{{\prime }} ;{\mathbf{n}}^{{\prime }} ) $$, $$ F(z_{
\cdot
}
;{\mathbf{s}}^{{\prime }} ;{\mathbf{n}}^{{\prime }} ) $$ with $$ F_{ \cdot } (z_{ \cdot } ;{\mathbf{s}}^{{\prime }} ;{\mathbf{n}}^{{\prime }} ) $$, $$ F(s_{ \cdot } ;{\mathbf{s}}^{{\prime }} ;{\mathbf{\overset{\lower0.5em\hbox{$\smash{\scriptscriptstyle\frown}$}}{n^{{\prime }}} }} ) $$ with $$ F_{ \cdot } (s_{ \cdot } ;{\mathbf{s}}^{{\prime }} ;{\mathbf{\overset{\lower0.5em\hbox{$\smash{\scriptscriptstyle\frown}$}}{n^{{\prime }} } }}) $$, $$ \overset{\lower0.5em\hbox{$\smash{\scriptscriptstyle\smile}$}}{F} (s_{ \cdot } ;{\mathbf{s}}^{{\prime }} ;{\mathbf{m}}^{{\prime }} ) $$ with $$ \overset{\lower0.5em\hbox{$\smash{\scriptscriptstyle\smile}$}}{F}_{ \cdot } (s_{ \cdot } ;{\mathbf{s}}^{{\prime }} ;{\mathbf{m}}^{{\prime }} ) $$, $$ F(s_{ \cdot } ;{\mathbf{s}}_{\text{a}} ;{\mathbf{m}}) $$ with $$ F_{ \cdot } (s_{ \cdot } ;{\mathbf{s}}_{\text{a}} ;{\mathbf{m}}) $$ and $$ F(s_{{{\text{cid}}( \cdot )}} + \varepsilon \rho_{ \cdot } ;{\mathbf{s}}^{{\prime }} ;{\mathbf{n}}^{{\prime }} ) $$ with $$ F_{ \cdot } (s_{{{\text{cid}}( \cdot )}} + \varepsilon \rho_{ \cdot } ;{\mathbf{s}}^{{\prime }} ;{\mathbf{n}}^{{\prime }} ) $$ throughout this paper (except in this section, Sect. 7, and Appendix K) gives the complete proofs for Theorems 1–3 for the fitness function $$ F({\mathbf{v}}_{j} ;{\mathbf{V}}^{{\prime }} ;{\mathbf{n}}^{{\prime }} ) $$.

## Appendix J: Tighter estimates

### J.1 Main derivation

Here we derive an approximability condition based on the first-order approximation of the perturbation term, corresponding to the zeroth-order approximability condition in Sect. 4, Eq. (4.8a),

J.1 $$ \sqrt \varepsilon < \frac{{ - \lambda_{\rm max} }}{{2\sqrt {C_{{\mathbf{h}}} C_{{\mathbf{r}}} } }} , $$

which is applied when the initial equilibrium population densities of approximate phenotypes are not small. The standardized dynamics of the approximate phenotypes is given by Eq. (4.4) in the main text,

J.2 $$ \frac{{{\text{d}}{\mathbf{x}}}}{{{\text{d}}t}} = {\mathbf{Ax}} + {\mathbf{r}}\left| {\mathbf{x}} \right|^{2} + \varepsilon {\mathbf{h}} . $$

By Taylor’s theorem, the perturbation term expands as

J.3 $$ \begin{aligned} {\mathbf{h}} & = \left. {\mathbf{h}} \right|_{{{\mathbf{x}} = {\mathbf{0}}}} + \left. {\frac{{\partial {\mathbf{h}}}}{{\partial {\mathbf{x}}}}} \right|_{{{\mathbf{x}} = {\mathbf{x}}_{\text{T}} }} {\mathbf{x}} \\ & = {\mathbf{\overset{\lower0.5em\hbox{$\smash{\scriptscriptstyle\frown}$}}{h} }} + {\mathbf{Hx}}, \\ \end{aligned} $$

where ${\mathbf{H}}$ is an $M \times M$ matrix given by

J.4 $$ {\mathbf{H}} = \left( {\begin{array}{*{20}c} {\frac{{\partial h_{1} }}{{\partial x_{1} }}} & \ldots & {\frac{{\partial h_{1} }}{{\partial x_{M} }}} \\ \ldots & \ldots & \ldots \\ {\frac{{\partial h_{M} }}{{\partial x_{1} }}} & \ldots & {\frac{{\partial h_{M} }}{{\partial x_{M} }}} \\ \end{array} } \right)_{{{\mathbf{x}} = {\mathbf{x}}_{\text{T}} }} , $$

where $$ {\mathbf{x}} = (x_{1} , \ldots ,x_{M} )^{\text{T}} $$ and $$ {\mathbf{x}}_{\text{T}} = \theta_{\text{T}} {\mathbf{x}} $$ with some appropriately chosen $$ \theta_{\text{T}} \in [0,1] $$. There exist constants $$ \overset{\lower0.5em\hbox{$\smash{\scriptscriptstyle\frown}$}}{C_{{\mathbf{h}}}} $$ and $$ C_{{\mathbf{H}}} $$ such that $$ \left| {{\mathbf{\overset{\lower0.5em\hbox{$\smash{\scriptscriptstyle\frown}$}}{h} }}} \right| \le \overset{\lower0.5em\hbox{$\smash{\scriptscriptstyle\frown}$}}{C_{{\mathbf{h}}}} $$ and $$ \left\| {\mathbf{H}} \right\| \le C_{{\mathbf{H}}} $$; see next subsection. We introduce

J.5 $$ c_{{\mathbf{h}}} (\phi_{\text{x}} ): = \overset{\lower0.5em\hbox{$\smash{\scriptscriptstyle\frown}$}}{C_{{\mathbf{h}}}} + C_{{\mathbf{H}}} \phi_{\text{x}} , $$

which clearly satisfies

J.6 $$ \mathop {\hbox{max} }\limits_{{\left| {\mathbf{x}} \right| \le \phi_{\text{x}} }} \left( {\left| {\mathbf{h}} \right|} \right) \le c_{{\mathbf{h}}} (\phi_{\text{x}} ) . $$

This means that $\left| {\mathbf{h}} \right|$ does not exceed $c_{{\mathbf{h}}} (\phi_{\text{x}} )$ when ${\mathbf{x}}$ is within a circle of radius $\phi_{\text{x}}$. Thus, replacing $C_{\text{h}}$ with $c_{\text{h}} (\phi_{\text{x}} )$ in Eqs. (4.8a) and (4.8b) in Lemma 5 in Sect. 4, we have

J.7 $$ \varepsilon < \frac{{\lambda_{\rm max}^{2} }}{{4c_{\text{h}} (\phi_{\text{x}} )C_{\text{r}} }} = \frac{{\lambda_{\rm max}^{2} }}{{4(\overset{\lower0.5em\hbox{$\smash{\scriptscriptstyle\frown}$}}{C_{{\mathbf{h}}}} + C_{{\mathbf{H}}} \phi_{\text{x}} )C_{{\mathbf{r}}} }} $$

and

J.8 $$ \left| {\mathbf{x}} \right| \le 2\varepsilon \frac{{c_{{\mathbf{h}}} (\phi_{\text{x}} )}}{{\left| {\lambda_{\rm max} } \right|}} = 2\varepsilon \frac{{\overset{\lower0.5em\hbox{$\smash{\scriptscriptstyle\frown}$}}{C_{{\mathbf{h}}}} + C_{{\mathbf{H}}} \phi_{\text{x}} }}{{\left| {\lambda_{\rm max} } \right|}} . $$

Equation (J.8) is ensured by Eq. (J.7), as long as $\phi_{\text{x}}$ is appropriately chosen so that the right-hand side of Eq. (J.8) does not exceed $\phi_{\text{x}}$, i.e.,

J.9 $$ 2\varepsilon \frac{{\overset{\lower0.5em\hbox{$\smash{\scriptscriptstyle\frown}$}}{C_{{\mathbf{h}}}} + C_{{\mathbf{H}}} \phi_{\text{x}} }}{{\left| {\lambda_{\rm max} } \right|}} \le \phi_{\text{x}} . $$

Clearly, the smallest $\phi_{\text{x}}$ that satisfies both Eq. (J.7) and Eq. (J.9) makes the approximability condition Eq. (J.7) the easiest to satisfy for a given $\lambda_{\rm max}$, and simultaneously makes the right-hand side of Eq. (J.8) the smallest. Such a $\phi_{\text{x}}$ is given by solving Eq. (J.9) assuming equality, i.e.,

J.10 $$ \phi_{\text{x}} = \frac{{2\varepsilon \overset{\lower0.5em\hbox{$\smash{\scriptscriptstyle\frown}$}}{C_{{\mathbf{h}}}} }}{{\left| {\lambda_{\rm max} } \right| - 2\varepsilon C_{{\mathbf{H}}} }} . $$

Substituting Eq. (J.10) into Eq. (J.7) gives the improved stability condition, Eq. (6.2) in the main text,

J.11 $$ \sqrt \varepsilon < \frac{{ - \lambda_{\rm max} }}{{\sqrt {4\overset{\lower0.5em\hbox{$\smash{\scriptscriptstyle\frown}$}}{C_{{\mathbf{h}}}} C_{{\mathbf{r}}} + 2C_{{\mathbf{H}}} \big| {\lambda_{\rm max} } \big|} }} . $$

This approximability condition can be further improved by the higher-order approximation of the nonlinear term and/or perturbation term, although the resultant conditions will be less simple than Eq. (J.11).

### J.2 Finding $C_{{\mathbf{h}}}$ and $C_{{\mathbf{H}}}$

From Eqs. (C.11), (C.19), and (D.2), ${\mathbf{h}}$ is given by

J.12 $$ {\mathbf{h}} = {\mathbf{Ph}}_{\text{m}} { = }{\mathbf{P}}{\text{diag}}({\mathbf{m}}){\mathbf{h}}_{\text{f}} . $$

with $$ {\mathbf{h}}_{\text{f}} = (h_{{{\text{f}}1}} , \ldots ,h_{{{\text{f}}M}} )^{\text{T}} $$ and

J.13 $$ h_{{{\text{f}}i}} = \frac{1}{\varepsilon }F(s_{i} ;{\mathbf{s}}^{{\prime }} ;{\mathbf{\overset{\lower0.5em\hbox{$\smash{\scriptscriptstyle\frown}$}}{n^{{\prime }} } }}) - \overset{\lower0.5em\hbox{$\smash{\scriptscriptstyle\frown}$}}{F}_{\varepsilon i} + \sum\limits_{{j \in {\text{com}}(i)}} {p_{j} F_{\varepsilon j} } . $$

As for $$ \overset{\lower0.5em\hbox{$\smash{\scriptscriptstyle\frown}$}}{C_{{\mathbf{h}}}} $$,

J.14 $$ {\mathbf{\overset{\lower0.5em\hbox{$\smash{\scriptscriptstyle\frown}$}}{h} }} = \left. {\mathbf{h}} \right|_{{{\mathbf{m}} = {\mathbf{\overset{\lower0.5em\hbox{$\smash{\scriptscriptstyle\frown}$}}{m} }}}} = \left[ {{\mathbf{P}}{\text{diag}}({\mathbf{\overset{\lower0.5em\hbox{$\smash{\scriptscriptstyle\frown}$}}{m} }}){\mathbf{h}}_{\text{f}} } \right]_{{{\mathbf{m}} = {\mathbf{\overset{\lower0.5em\hbox{$\smash{\scriptscriptstyle\frown}$}}{m} }}}} $$

gives

J.15 $$ \left| {{\mathbf{\overset{\lower0.5em\hbox{$\smash{\scriptscriptstyle\frown}$}}{h} }}} \right| \le \left\| {{\mathbf{P}}{\text{diag}}({\mathbf{\overset{\lower0.5em\hbox{$\smash{\scriptscriptstyle\frown}$}}{m} }}){\mathbf{h}}_{\text{f}} } \right\|_{{{\mathbf{m}} = {\mathbf{\overset{\lower0.5em\hbox{$\smash{\scriptscriptstyle\frown}$}}{m} }}}} = :\overset{\lower0.5em\hbox{$\smash{\scriptscriptstyle\frown}$}}{C_{{\mathbf{h}}}} . $$

As for $$ C_{{\mathbf{H}}} $$, we first transform $$ {\mathbf{H}} $$ by using $$ {\mathbf{x}} = {\mathbf{P}}({\mathbf{m}} - {\mathbf{\overset{\lower0.5em\hbox{$\smash{\scriptscriptstyle\frown}$}}{m} }}) $$ and Eq. (J.12) as

J.16 $$ \begin{aligned} {\mathbf{H}} & = \frac{{\partial {\mathbf{h}}}}{{\partial {\mathbf{x}}}} = \frac{{\partial {\mathbf{h}}}}{{\partial {\mathbf{m}}}}\frac{{\partial {\mathbf{m}}}}{{\partial {\mathbf{x}}}} = {\mathbf{P}}\frac{{\partial [{\text{diag}}({\mathbf{m}}){\mathbf{h}}_{\text{f}} ]}}{{\partial {\mathbf{m}}}}{\mathbf{P}}^{ - 1} \\ & = {\mathbf{P}}\left( {\begin{array}{*{20}c} {\frac{{\partial m_{1} h_{\text{f1}} }}{{\partial m_{1} }}} & \cdots & {\frac{{\partial m_{1} h_{\text{f1}} }}{{\partial m_{M} }}} \\ \vdots & \ddots & \vdots \\ {\frac{{\partial m_{M} h_{{{\text{f}}M}} }}{{\partial m_{1} }}} & \cdots & {\frac{{\partial m_{M} h_{{{\text{f}}M}} }}{{\partial m_{M} }}} \\ \end{array} } \right){\mathbf{P}}^{ - 1} . \\ \end{aligned} $$

To calculate $$ \frac{{\partial m_{i} h_{{{\text{f}}i}} }}{{\partial m_{k} }} $$ for $$ i,k = 1, \ldots ,M $$, we transform $$ m_{i} h_{{{\text{f}}i}} $$ by using Eq. (J.13), Eqs. (A.6), and $$ F(s_{i} ;{\mathbf{s}}^{{\prime }} ;{\mathbf{\overset{\lower0.5em\hbox{$\smash{\scriptscriptstyle\frown}$}}{n^{{\prime }}} }} ) = 0 $$ for $$ i = 1, \ldots ,M $$ as

J.17 $$ \begin{aligned} m_{i} h_{{{\text{f}}i}} & = - m\overset{\lower0.5em\hbox{$\smash{\scriptscriptstyle\frown}$}}{F}_{\varepsilon i} + \sum\limits_{{j \in {\text{com}}(i)}} {n_{j} F_{\varepsilon j} } \\ & = - m_{i} \overset{\lower0.5em\hbox{$\smash{\scriptscriptstyle\frown}$}}{F}_{\varepsilon i} + n_{i} F_{\varepsilon i} + \sum\limits_{{j \in {\text{com}}(i),j \ne i}} {n_{j} F_{\varepsilon j} } \\ & = - m\overset{\lower0.5em\hbox{$\smash{\scriptscriptstyle\frown}$}}{F}_{\varepsilon i} + \left( {m_{i} - \frac{1}{\varepsilon }\sum\limits_{{j \in {\text{com}}(i),j \ne i}} {m_{j} } } \right)F_{\varepsilon i} + \frac{1}{\varepsilon }\sum\limits_{{j \in {\text{com}}(i),j \ne i}} {m_{j} F_{\varepsilon j} } \\ & = m_{i} (F_{\varepsilon i} - \overset{\lower0.5em\hbox{$\smash{\scriptscriptstyle\frown}$}}{F}_{\varepsilon i} ) - \frac{1}{\varepsilon }\sum\limits_{{j \in {\text{com}}(i),j \ne i}} {m_{j} F_{\varepsilon i} } + \frac{1}{\varepsilon }\sum\limits_{{j \in {\text{com}}(i),j \ne i}} {m_{j} F_{\varepsilon j} } . \\ \end{aligned} $$

Since $j \in {\text{com}}(i)$ and $j \ne i$ require $j \in \left\{ {M + 1, \ldots ,N + 1} \right\}$, we see for $i,k = 1, \ldots ,M$

J.18 $$ \begin{aligned} \frac{{\partial m_{i} h_{{{\text{f}}i}} }}{{\partial m_{k} }} & = \frac{{\partial m_{i} }}{{\partial m_{k} }}(F_{\varepsilon i} - \overset{\lower0.5em\hbox{$\smash{\scriptscriptstyle\frown}$}}{F}_{\varepsilon i} ) + m_{i} \frac{{\partial F_{\varepsilon i} }}{{\partial m_{k} }} - \frac{1}{\varepsilon }\sum\limits_{{j \in {\text{com}}(i),j \ne i}} {m_{j} \frac{{\partial F_{\varepsilon i} }}{{\partial m_{k} }}} + \frac{1}{\varepsilon }\sum\limits_{{j \in {\text{com}}(i),j \ne i}} {m_{j} \frac{{\partial F_{\varepsilon j} }}{{\partial m_{k} }}} \\ & = \delta (i - k)(F_{\varepsilon i} - \overset{\lower0.5em\hbox{$\smash{\scriptscriptstyle\frown}$}}{F}_{\varepsilon i} ) + \sum\limits_{{j \in {\text{com}}(i)}} {n_{j} \frac{{\partial F_{\varepsilon i} }}{{\partial m_{k} }}} - \sum\limits_{{j \in {\text{com}}(i),j \ne i}} {n_{j} \frac{{\partial F_{\varepsilon i} }}{{\partial m_{k} }}} + \sum\limits_{{j \in {\text{com}}(i),j \ne i}} {n_{j} \frac{{\partial F_{\varepsilon j} }}{{\partial m_{k} }}} \\ & = \delta (i - k)(F_{\varepsilon i} - \overset{\lower0.5em\hbox{$\smash{\scriptscriptstyle\frown}$}}{F}_{\varepsilon i} ) + \sum\limits_{{j \in {\text{com}}(i)}} {n_{j} \frac{{\partial F_{\varepsilon j} }}{{\partial m_{k} }}} \\ & = \delta (i - k)(F_{\varepsilon i} - \overset{\lower0.5em\hbox{$\smash{\scriptscriptstyle\frown}$}}{F}_{\varepsilon i} ) + {\mathbf{c}}_{i}^{\text{T}} {\text{diag}}({\mathbf{n^{\prime}}})\frac{{\partial {\mathbf{F^{\prime}}}_{\varepsilon } }}{{\partial m_{k} }}, \\ \end{aligned} $$

where $\delta (i - k) = 1$ for $i = k$, $\delta (i - k) = 0$ otherwise, ${\mathbf{F}}_{\varepsilon }^{{\prime }} : = (F_{\varepsilon 1} , \ldots ,F_{\varepsilon, \,N + 1} )^{\text{T}}$, and ${\mathbf{c}}_{i}$ is a vector of length $N + 1$ with entries 0 or 1 such that ${\mathbf{c}}_{i}^{\text{T}} {\mathbf{n}}^{{\prime }} = \sum\nolimits_{{j \in {\text{com}}(i)}} {n_{j} } = m_{i}$. We transform Eq. (J.18) into vector–matrix form,

J.19 $$ \begin{aligned} \frac{{\partial \left[ {{\text{diag}}({\mathbf{m}}){\mathbf{h}}_{\text{f}} } \right]}}{{\partial {\mathbf{m}}}} & = {\text{diag}}({\mathbf{F}}_{\varepsilon } - {\mathbf{\overset{\lower0.5em\hbox{$\smash{\scriptscriptstyle\frown}$}}{F} }}_{\varepsilon } ) + \left( {\begin{array}{*{20}c} {{\mathbf{c}}_{1}^{\text{T}} {\text{diag}}({\mathbf{n}}^{{\prime }} )\frac{{\partial {\mathbf{F}}_{\varepsilon }^{{\prime }} }}{{\partial m_{1} }}} & \cdots & {{\mathbf{c}}_{1}^{\text{T}} {\text{diag}}({\mathbf{n}}^{{\prime }} )\frac{{\partial {\mathbf{F}}_{\varepsilon }^{{\prime }} }}{{\partial m_{M} }}} \\ \vdots & \ddots & \vdots \\ {{\mathbf{c}}_{M}^{\text{T}} {\text{diag}}({\mathbf{n}}^{{\prime }} )\frac{{\partial {\mathbf{F^{\prime}}}_{\varepsilon } }}{{\partial m_{1} }}} & \cdots & {{\mathbf{c}}_{M}^{\text{T}} {\text{diag}}({\mathbf{n}}^{{\prime }} )\frac{{\partial {\mathbf{F}}_{\varepsilon }^{{\prime }} }}{{\partial m_{M} }}} \\ \end{array} } \right) \\ & = {\text{diag}}({\mathbf{F}}_{\varepsilon } - {\mathbf{\overset{\lower0.5em\hbox{$\smash{\scriptscriptstyle\frown}$}}{F} }}_{\varepsilon } ) + \left( {\begin{array}{*{20}c} {{\mathbf{c}}_{1}^{\text{T}} {\text{diag}}({\mathbf{n}}^{{\prime }} )} \\ \vdots \\ {{\mathbf{c}}_{M}^{\text{T}} {\text{diag}}({\mathbf{n}}^{{\prime }} )} \\ \end{array} } \right)\left( {\begin{array}{*{20}c} {\frac{{\partial {\mathbf{F}}_{\varepsilon }^{{\prime }} }}{{\partial m_{1} }}} & \cdots & {\frac{{\partial {\mathbf{F}}_{\varepsilon }^{{\prime }} }}{{\partial m_{M} }}} \\ \end{array} } \right) \\ & = {\text{diag}}({\mathbf{F}}_{\varepsilon } - {\mathbf{\overset{\lower0.5em\hbox{$\smash{\scriptscriptstyle\frown}$}}{F} }}_{\varepsilon } ) + \left( {\begin{array}{*{20}c} {{\mathbf{c}}_{1}^{\text{T}} } \\ \vdots \\ {{\mathbf{c}}_{M}^{\text{T}} } \\ \end{array} } \right){\text{diag}}({\mathbf{n}}^{{\prime }} )\frac{{\partial {\mathbf{F}}_{\varepsilon }^{{\prime }} }}{{\partial {\mathbf{m}}}}. \\ \end{aligned} $$

Substituting Eq. (J.19) into Eq. (J.16), we find

J.20 $$ \begin{aligned} \left\| {\mathbf{H}} \right\| & \le \left\| {\mathbf{P}} \right\|\left\| {\frac{{\partial [{\text{diag}}({\mathbf{m}}){\mathbf{h}}_{\text{f}} ]}}{{\partial {\mathbf{m}}}}} \right\|\left\| {{\mathbf{P}}^{ - 1} } \right\| \\ & \le \left\| {\mathbf{P}} \right\|\left[ {\left\| {{\text{diag}}({\mathbf{F}}_{\varepsilon } - {\mathbf{\overset{\lower0.5em\hbox{$\smash{\scriptscriptstyle\frown}$}}{F} }}_{\varepsilon } )} \right\| + \left\| {\left( {\begin{array}{*{20}c} {{\mathbf{c}}_{1}^{\text{T}} } \\ {} \\ {{\mathbf{c}}_{M}^{\text{T}} } \\ \end{array} } \right)} \right\|\left\| {{\text{diag}}({\mathbf{n}}^{{\prime }} )} \right\|\left\| {\frac{{\partial {\mathbf{F}}_{\varepsilon }^{{\prime }} }}{{\partial {\mathbf{m}}}}} \right\|} \right]\left\| {{\mathbf{P}}^{ - 1} } \right\| \\ & \le \left\| {\mathbf{P}} \right\|\left[ {2\mathop {\hbox{max} }\limits_{{i \in \{ 1, \ldots ,M\} }} (\left| {F_{\varepsilon i} } \right|) + (N + 1)\mathop {\hbox{max} }\limits_{{i \in \{ 1, \ldots ,N + 1\} }} (\left| {n_{i} } \right|)\left\| {\frac{{\partial {\mathbf{F}}_{\varepsilon }^{{\prime }} }}{{\partial {\mathbf{m}}}}} \right\|} \right]\left\| {{\mathbf{P}}^{ - 1} } \right\| \\ & \le \left\| {\mathbf{P}} \right\|\left\| {{\mathbf{P}}^{ - 1} } \right\|\left[ {2C_{{{\text{F}}\varepsilon }} + (N + 1)\eta C_{{{\mathbf{F}}\varepsilon {\mathbf{m}}}} } \right] = :C_{{\mathbf{H}}} , \\ \end{aligned} $$

with

J.21 $$ C_{{{\text{F}}\varepsilon }} : = \hbox{max} \left\{ {\left| {\frac{{\partial F(s_{{{\text{cid}}(i)}} + \varepsilon \rho_{i} ;{\mathbf{s}}_{a}^{\prime } + \varepsilon {\varvec{\uprho}}^{\prime } ;{\mathbf{n}}^{\prime } )}}{\partial \varepsilon }} \right|_{{\varepsilon = \varepsilon_{{{\text{T}}i}} }} \;\left| {\begin{array}{*{20}l} {i = 1, \ldots ,M,} \hfill \\ {{\mathbf{n}}^{\prime } \in [0,\eta ]^{N + 1} ,\;\varepsilon_{{{\text{T}}i}} \in [0,\varepsilon ]} \hfill \\ \end{array} } \right.} \right\} $$

and

J.22 $$ \begin{aligned} C_{{{\mathbf{F}}\varepsilon {\mathbf{m}}}} & : = \hbox{max} \left\{ {\left\| {\frac{{\partial
{\mathbf{F}}_{\varepsilon }^{{\prime }} }}{{\partial {\mathbf{m}}}}} \right\|\,\,\,\left| \begin{aligned} \varepsilon_{{{\text{T}}i}} \in [0,\varepsilon ], \hfill \\ m_{1} , \ldots
,m_{M} \in [0,\eta ], \hfill \\ m_{M + 1} , \ldots ,m_{N + 1} \in [0,\varepsilon \eta ] \hfill \\ \end{aligned} \right.\,\,} \right\}, \\ {\mathbf{F}}_{\varepsilon }^{{\prime }} & = \left( {\begin{array}{l} {F_{\varepsilon 1} } \\ \vdots \\ {F_{\varepsilon ,N + 1} } \\ \end{array} } \right) = \left( {\begin{array}{*{20}c} {\frac{{\partial \overset{\lower0.5em\hbox{$\smash{\scriptscriptstyle\smile}$}}{F} (s_{{{\text{cid}}(1)}} + \varepsilon \rho_{1} ;{\mathbf{s}}_{\text{a}}^{{\prime }} + \varepsilon {\varvec{\uprho}}^{{\prime }} ;{\mathbf{m}}^{{\prime }} )}}{\partial \varepsilon }} \\ \vdots \\ {\frac{{\partial \overset{\lower0.5em\hbox{$\smash{\scriptscriptstyle\smile}$}}{F} (s_{{{\text{cid}}(N + 1)}} + \varepsilon \rho_{N + 1} ;{\mathbf{s}}_{\text{a}}^{{\prime }} + \varepsilon {\varvec{\uprho}}^{{\prime }} ;{\mathbf{m}}^{{\prime }} )}}{\partial \varepsilon }} \\ \end{array} } \right), \\ \end{aligned} $$

where $$ F_{\varepsilon i} $$ is defined by Eq. (C.2) and $${{ \overset{\lower0.5em\hbox{$\smash{\scriptscriptstyle\smile}$}}{F} (s_{i} ;{{\mathbf{s}}}^{{\prime }}; {\mathbf{m}}^{{\prime }})}} = {{F} (s_{i} ;{{\mathbf{s}}}^{{\prime }}; {\mathbf{n}}^{{\prime }})}$$ as in Lemma 1.

## Appendix K: Proof of Eq. (8.2)

### K.1 Main proof

First, we derive Eqs. (8.1) from Eq. (4.1a) in the main text,

K.1 $$ \begin{aligned} \frac{{{\text{d}}p_{N + 1} }}{{{\text{d}}t}} & = \frac{\text{d}}{{{\text{d}}t}}\left[ {\frac{{n_{N + 1} }}{{m_{i} }}} \right] = \frac{1}{{m_{i} }}\frac{{{\text{d}}n_{N + 1} }}{{{\text{d}}t}} - \frac{{n_{N + 1} }}{{m_{i}^{2} }}\frac{{{\text{d}}m_{i} }}{{{\text{d}}t}} \\ & = \frac{{n_{N + 1} }}{{m_{i} }}F(s_{N + 1} ;{\mathbf{s^{\prime}}};{\mathbf{n^{\prime}}}) - \frac{{n_{N + 1} }}{{m_{i} }}f(s_{i} ;{\mathbf{s}}^{{\prime }} ;{\mathbf{n}}^{{\prime }} ) \\ & = p_{N + 1} F(s_{N + 1} ;{\mathbf{s}}^{{\prime }} ;{\mathbf{n}}^{{\prime }} ) - p_{N + 1} \sum\limits_{{j \in {\text{com}}(i)}} {p_{j} F(s_{j} ;{\mathbf{s}}^{{\prime }} ;{\mathbf{n}}^{{\prime }} )} \\ & = p_{N + 1} \left[ {F(s_{N + 1} ;{\mathbf{s}}^{{\prime }} ;{\mathbf{n}}^{{\prime }} ) - \sum\limits_{{j \in {\text{com}}(i)}} {p_{j} F(s_{j} ;{\mathbf{s}}^{{\prime }} ;{\mathbf{n}}^{{\prime }} )} } \right]. \\ \end{aligned} $$

This is further transformed as

K.2 $$ \begin{aligned} \frac{{{\text{d}}p_{N + 1} }}{{{\text{d}}t}} & = p_{N + 1} \left[ {(1 - p_{N + 1} )F(s_{N + 1} ;{\mathbf{s}}^{{\prime }} ;{\mathbf{n}}^{{\prime }} ) - \sum\limits_{{j \in {\text{com}}(i),j \ne N + 1}} {p_{j} F(s_{j} ;{\mathbf{s}}^{{\prime }} ;{\mathbf{n}}^{{\prime }} )} } \right] \\ & = p_{N + 1} (1 - p_{N + 1} )\left[ {F(s_{N + 1} ;{\mathbf{s}}^{{\prime }} ;{\mathbf{n}}^{{\prime }} ) - \frac{1}{{1 - p_{N + 1} }}\sum\limits_{{j \in {\text{com}}(i),j \ne N + 1}} {p_{j} F(s_{j} ;{\mathbf{s}}^{{\prime }} ;{\mathbf{n}}^{{\prime }} )} } \right] \\ & = p_{N + 1} (1 - p_{N + 1} )\left[ {F(s_{N + 1} ;{\mathbf{s}}^{{\prime }} ;{\mathbf{n}}^{{\prime }} ) - \sum\limits_{{j \in {\text{com}}(i),j \ne N + 1}} {\tilde{p}_{j} F(s_{j} ;{\mathbf{s}}^{{\prime }} ;{\mathbf{n}}^{{\prime }} )} } \right], \\ \end{aligned} $$

where $\tilde{p}_{j} = p_{j} /(1 - p_{N + 1} ) = n_{j} /(m_{i} - n_{N + 1} )$ is the proportion of phenotype $s_{j}$ in the $i$th cluster when the mutant $s_{N + 1}$ is removed. Thus, the sign of $\frac{{{\text{d}}p_{N + 1} }}{{{\text{d}}t}}$ is determined by the terms inside the square bracket. The $F(s_{j} ;{\mathbf{s}}^{{\prime }} ;{\mathbf{n}}^{{\prime }} )$ is expanded in $s_{j}$ around $s_{i} = s_{{{\text{cid}}(j)}}$ as

K.3 $$ \begin{aligned} F(s_{j} ;{\mathbf{s}}^{{\prime }} ;{\mathbf{n}}^{{\prime }} ) & = F(s_{i} ;{\mathbf{s}}^{{\prime }} ;{\mathbf{n}}^{{\prime }} ) + \left. {\frac{{\partial F(z_{j} ;{\mathbf{s}}^{{\prime }} ;{\mathbf{n}}^{{\prime }} )}}{{\partial z_{j} }}} \right|_{{z_{j} = s_{i} }} (s_{j} - s_{i} ) + \frac{1}{2}\left. {\frac{{\partial^{2} F(z_{j} ;{\mathbf{s}}^{{\prime }} ;{\mathbf{n}}^{{\prime }} )}}{{\partial z_{j}^{2} }}} \right|_{{z_{j} = s_{{j{\text{T}}}} }} (s_{j} - s_{i} )^{2} \\ & \quad = F(s_{i} ;{\mathbf{s}}^{{\prime }} ;{\mathbf{n}}^{{\prime }} ) + \left. {\frac{{\partial F(z_{j} ;{\mathbf{s}}^{{\prime }} ;{\mathbf{n}}^{{\prime }} )}}{{\partial z_{j} }}} \right|_{{z_{j} = s_{i} }} \rho_{j} \varepsilon + \frac{1}{2}\left. {\frac{{\partial^{2} F(z_{j} ;{\mathbf{s}}^{{\prime }} ;{\mathbf{n}}^{{\prime }} )}}{{\partial z_{j}^{2} }}} \right|_{{z_{j} = s_{{j{\text{T}}}} }} \rho_{j}^{2} \varepsilon^{2} \\ & \quad = F(s_{i} ;{\mathbf{s}}^{{\prime }} ;{\mathbf{n}}^{{\prime }} ) + F_{\text{z}} (s_{i} )\rho_{j} \varepsilon + \frac{1}{2}F_{\text{zz}} (s_{{j{\text{T}}}} )\rho_{j}^{2} \varepsilon^{2} \\ \end{aligned} $$

with some appropriately chosen $s_{{j{\text{T}}}} \in [s_{i} ,s_{j} ]$, where $\left| {\rho_{j} } \right| \le 1$ because the within-cluster phenotypic differences do not exceed $\varepsilon$. Substituting Eq. (K.3) into Eq. (K.2) transforms the term inside of the square bracket as

K.4 $$ \begin{aligned}    & F(s_{{N + 1}} ;{\mathbf{s}}^{\prime } ;{\mathbf{n}}^{\prime } ) - \sum\limits_{{j \in {\text{com}}(i),j \ne N + 1}} {\tilde{p}_{j} F(s_{j} ;{\mathbf{s}}^{\prime } ;{\mathbf{n}}^{\prime } )}  \\     & \quad  = F(s_{i} ;{\mathbf{s}}^{\prime } ;{\mathbf{n}}^{\prime } ) + F_{z} (s_{i} )\rho _{{N + 1}} \varepsilon  + \frac{1}{2}F_{{zz}} (s_{{N + 1,{\text{T}}}} )\rho _{{N + 1}}^{2} \varepsilon ^{2}  \\     & \quad \quad  - \,\sum\limits_{{j \in {\text{com}}(i),j \ne N + 1}} {\tilde{p}_{j} \left[ {F(s_{i} ;{\mathbf{s}}^{\prime } ;{\mathbf{n}}^{\prime } ) + F_{z} (s_{i} )\rho _{j} \varepsilon  + \frac{1}{2}F_{{zz}} (s_{{j{\text{T}}}} )\rho _{j}^{2} \varepsilon ^{2} } \right]}  \\     & \quad  = \varepsilon F_{z} (s_{i} )\left[ {\rho _{{N + 1}}  - \sum\limits_{{j \in {\text{com}}(i),j \ne N + 1}} {\tilde{p}_{j} \rho _{j} } } \right] \\ &\qquad + \frac{{\varepsilon ^{2} }}{2}\left[ {F_{{zz}} (s_{{N + 1,{\text{T}}}} )\rho _{{N + 1}}^{2}  - \sum\limits_{{j \in {\text{com}}(i),j \ne N + 1}} {\tilde{p}_{j} F_{{zz}} (s_{{j{\text{T}}}} )\rho _{j}^{2} } } \right]. \\  \end{aligned}  $$

The second term satisfies

K.5 $$ \begin{aligned} & \left| {F_{zz} (s_{{N + 1 , {\text{T}}}} )\rho_{N + 1}^{2} - \sum\limits_{{j \in {\text{com}}(i),j \ne N + 1}} {\tilde{p}_{j} F_{zz} (s_{{j{\text{T}}}} )\rho_{j}^{2} } } \right| \\ & \quad \le \left| {F_{zz} (s_{{N + 1 , {\text{T}}}} )} \right|\rho_{N + 1}^{2} + \mathop {\hbox{max} }\limits_{{j \in {\text{com}}(i),j \ne N + 1}} \left| {F_{zz} (s_{{j{\text{T}}}} )} \right|\rho_{j}^{2} \\ & \quad \le 2\mathop {\hbox{max} }\limits_{{j \in {\text{com}}(i),j \ne i}} \left( {\left| {F_{zz} (s_{{j{\text{T}}}} )} \right|} \right) \\ & \quad \le 2C_{\text{Fzz}}^{{\prime }}, \\ \end{aligned} $$

where the constant $C_{\text{Fzz}}^{{\prime }}$ is defined by

K.6 $$ \begin{aligned} C_{\text{Fzz}}^{{\prime }} & : = \hbox{max} \left\{ \left. {\left| {\frac{{\partial^{2} F(z;{\mathbf{s}}^{{\prime }} ;{\mathbf{n}}^{{\prime }} )}}{{\partial z^{2} }}} \right|_{{z = s_{{j{\text{T}}}} }} } \right| \, \left| {\Delta {\mathbf{m}}^{{\prime }} } \right| \in [0,\varepsilon C_{{\mathbf{m}}}^{{\prime }} ], \right.\\ & \left. \quad j = M + 1, \ldots ,N + 1,\;s_{{j{\text{T}}}} \in [s_{j} ,s_{{{\text{cid(}}j)}} ] \right\} , \end{aligned} $$

while the first term is further transformed by expanding $F_{z} (s_{i} )$ in ${\mathbf{m}}^{{\prime }}$ as

K.7 $$ \begin{aligned}& \varepsilon F_{z} (s_{i} )\left[ {\rho_{N + 1} - \sum\limits_{{j \in {\text{com}}(i),j \ne N + 1}} {\tilde{p}_{j} (s_{i} )\rho_{j} } } \right] \\ & = F_{z} (s_{i} )\varepsilon \left[ {\rho_{N + 1} - \bar{\rho }_{i} } \right] \\ & = \left[ {\left. {\frac{{\partial F(z;{\mathbf{s}}^{{\prime }} ;{\mathbf{\overset{\lower0.5em\hbox{$\smash{\scriptscriptstyle\frown}$}}{n^{\prime}} }})}}{\partial z}} \right|_{{z = s_{i} }} + \left. {\frac{{\partial^{2} F(z;{\mathbf{s}}^{{\prime }} ;{\mathbf{n}}^{{\prime }} )}}{{\partial z\partial {\mathbf{m}}^{{\prime }} }}} \right|_{{z = s_{i} ,\Delta {\mathbf{m}}^{{\prime }} = \Delta {\mathbf{m^{\prime}}}_{\text{T}} }} \Delta {\mathbf{m}}^{{\prime }} } \right]\varepsilon \left[ {\rho_{N + 1} - \bar{\rho }_{i} } \right] \\ & \ge \left[ {\overset{\lower0.5em\hbox{$\smash{\scriptscriptstyle\frown}$}}{F}_{\text{z}} (s_{i} ) - C_{{{\text{Fz}}{\mathbf{m}}}}^{{\prime }} \left| {\Delta {\mathbf{m}}^{{\prime }} } \right|} \right]\varepsilon \left[ {\rho_{N + 1} - \bar{\rho }_{i} } \right] \\ & \ge \overset{\lower0.5em\hbox{$\smash{\scriptscriptstyle\frown}$}}{F}_{\text{z}} (s_{i} )\varepsilon (\rho_{N + 1} - \bar{\rho }_{i} ) - \varepsilon C_{{{\text{Fz}}{\mathbf{m}}}}^{{\prime }} \left| {\Delta {\mathbf{m}}^{{\prime }} } \right|, \\ \end{aligned} $$

where $\bar{\rho }: = \sum\nolimits_{{j \in {\text{com}}(i),j \ne N + 1}} {\tilde{p}_{j} (s_{i} )\rho_{j} }$ is the average value for $\rho_{j}$ of this cluster when the mutant $s_{N + 1}$ is removed, satisfying $|\rho_{N + 1} - \bar{\rho }_{i} | \le 1$, and $C_{{{\text{Fz}}{\mathbf{m}}}}^{{\prime }}$ is calculated in the next subsection. Substituting Eqs. (K.4), (K.5), and (K.7) into Eq. (K.2) gives

K.8 $$ \begin{aligned} \frac{{{\text{d}}p_{N + 1} }}{{{\text{d}}t}} & \ge p_{N + 1} (1 - p_{N + 1} )\left[ {\overset{\lower0.5em\hbox{$\smash{\scriptscriptstyle\frown}$}}{F}_{\text{z}} (s_{i} )\varepsilon (\rho_{N + 1} - \bar{\rho }_{i} ) - \varepsilon C_{{{\text{Fz}}{\mathbf{m}}}}^{{\prime }} \left| {\Delta {\mathbf{m}}^{{\prime }} } \right| - \varepsilon^{2} C_{\text{Fzz}} } \right] \\ & = p_{N + 1} (1 - p_{N + 1} )\left[ {\overset{\lower0.5em\hbox{$\smash{\scriptscriptstyle\frown}$}}{F}_{\text{z}} (s_{i} )(s_{N + 1} - \bar{s}_{i} ) - \varepsilon C_{{{\text{Fz}}{\mathbf{m}}}}^{{\prime }} \left| {\Delta {\mathbf{m}}^{{\prime }} } \right| - \varepsilon^{2} C_{\text{Fzz}} } \right], \\ \end{aligned} $$

where $\bar{s}_{i} = \varepsilon \bar{\rho }_{i} + s_{i} = \sum\nolimits_{{j \in {\text{com}}(i),j \ne N + 1}} {\tilde{p}_{j} (\varepsilon \rho_{j} + s_{i} )} = \sum\nolimits_{{j \in {\text{com}}(i),j \ne N + 1}} {\tilde{p}_{j} s_{j} }$ is the average trait value of this cluster when the mutant $s_{N + 1}$ is removed. For convenience, we assume that the representative phenotype is the most similar phenotype to the mutant, i.e., $\left| {s_{N + 1} - s_{i} } \right| = \min_{{k \in {\text{com}}(i)}} \left( {\left| {s_{N + 1} - s_{k} } \right|} \right)$. Notice that $\left| {s_{N + 1} - s_{i} } \right| \le \left| {s_{N + 1} - \bar{s}_{i} } \right|$. Then, by exploiting $\left| {\Delta {\mathbf{m}}^{{\prime }} } \right| \le \varepsilon C_{{\mathbf{m}}}^{{\prime }}$, a sufficient condition for Eq. (K.8) being always positive is given by

K.9 $$ \overset{\lower0.5em\hbox{$\smash{\scriptscriptstyle\frown}$}}{F}_{\text{z}} (s_{i} )(s_{N + 1} - s_{i} ) > \varepsilon^{2} \left[ {C_{{{\text{Fz}}{\mathbf{m}}}}^{{\prime }} C_{{\mathbf{m}}}^{{\prime }} + C_{\text{Fzz}} } \right] . $$

### K.2 Finding $C_{{{\text{Fz}}{\mathbf{m}}}}^{{\prime }}$

From Eq. (A.7), we find for $j = 1, \ldots ,M$

K.10 $$ \left| {\frac{{\partial^{2} F(z;{\mathbf{s}}^{{\prime }} ;{\mathbf{n}}^{{\prime }} )}}{{\partial m_{j} \partial z}}} \right|_{{z = s_{{j{\text{T}}}} }} = \left| {\sum\limits_{k = 1}^{N + 1} {\frac{{\partial n_{k} }}{{\partial m_{j} }}} \left[ {\frac{{\partial^{2} F(z;{\mathbf{s}}^{{\prime }} ;{\mathbf{n}}^{{\prime }} )}}{{\partial n_{k} \partial z}}} \right]_{{z = s_{{j{\text{T}}}} }} } \right| = \left| {\frac{{\partial^{2} F(z;{\mathbf{s}}^{{\prime }} ;{\mathbf{n}}^{{\prime }} )}}{{\partial n_{j} \partial z}}} \right|_{{z = s_{{j{\text{T}}}} }} \le C_{\text{Fzn}}^{{\prime }} , $$

for any $s_{{j{\text{T}}}} \in [s_{j} ,s_{{{\text{cid}}(j)}} ]$ with

K.11 $$ C_{\text{Fzn}}^{{\prime }} : = \hbox{max} \left\{ {\left. {\left| {\frac{{\partial^{2} F(z;{\mathbf{s}}^{{\prime }} ;{\mathbf{n}}^{{\prime }} )}}{{\partial z\partial n_{j} }}} \right|_{{z = s_{{j{\text{T}}}} }} } \right| \,\, j = 1, \ldots ,N + 1,\;{\mathbf{n}}^{{\prime }} \in [0,\eta ]^{N + 1} ,\;s_{{j{\text{T}}}} \in [s_{{{\text{cid}}(j)}} ,s_{j} ]} \right\} . $$

From Eq. (A.8) and Taylor’s theorem, we find for $j = M + 1, \ldots ,N + 1$ and for any $s_{{j{\text{T}}}} \in [s_{j} ,s_{{{\text{cid}}(j)}} ]$

K.12 $$ \begin{aligned} \left| {\left[ {\frac{{\partial^{2} F(z;{\mathbf{s}}^{{\prime }} ;{\mathbf{n}}^{{\prime }} )}}{{\partial m_{j} \partial z}}} \right]_{{z = s_{{j{\text{T}}}} }} } \right| & = \frac{1}{\varepsilon }\left| {\left[ {\frac{{\partial^{2} F(z;{\mathbf{s}}^{{\prime }} ;{\mathbf{n}}^{{\prime }} )}}{{\partial n_{j} \partial z}} - \frac{{\partial^{2} F(z;{\mathbf{s}}^{{\prime }} ;{\mathbf{n}}^{{\prime }} )}}{{\partial n_{{{\text{cid}}(j)}} \partial z}}} \right]_{{z = s_{{j{\text{T}}}} }} } \right| \\ & = \frac{1}{\varepsilon }\left| \left[ {\frac{{\partial^{2} F(z;{\mathbf{s}}^{{\prime }} ;{\mathbf{n}}^{{\prime }} )}}{{\partial n_{j} \partial z}} - \frac{{\partial^{2} F(z;{\mathbf{s}}^{{\prime }} ;{\mathbf{n}}^{{\prime }} )}}{{\partial n_{{{\text{cid}}(j)}} \partial z}}} \right]_{\begin{subarray}{l} z = s_{{j{\text{T}}}} , \\ s_{j} = s_{{{\text{cid}}(j)}} \end{subarray} } \right. \\ & \quad \left. +  \left[ {\frac{{\partial^{3} F(z;{\mathbf{s}}^{{\prime }} ;{\mathbf{n}}^{{\prime }} )}}{{\partial s_{j} \partial n_{j} \partial z}} - \frac{{\partial^{3} F(z;{\mathbf{s}}^{{\prime }} ;{\mathbf{n}}^{{\prime }} )}}{{\partial s_{j} \partial n_{{{\text{cid}}(j)}} \partial z}}} \right]_{\begin{subarray}{l} z = s_{{j{\text{T}}}} , \\ s_{j} = s_{{j{\text{TT}}}} \end{subarray} } (s_{j} - s_{{{\text{cid}}(j)}} ) \right| \\ & = \left| {\rho_{j} \left[ {\frac{{\partial^{3} F(z;{\mathbf{s}}^{{\prime }} ;{\mathbf{n}}^{{\prime }} )}}{{\partial s_{j} \partial n_{j} \partial z}} - \frac{{\partial^{3} F(z;{\mathbf{s}}^{{\prime }} ;{\mathbf{n}}^{{\prime }} )}}{{\partial s_{j} \partial n_{{{\text{cid}}(j)}} \partial z}}} \right]_{\begin{subarray}{l} z = s_{{j{\text{T}}}} , \\ s_{j} = s_{{j{\text{TT}}}} \end{subarray} } } \right| \le C_{\text{Fzsn}}^{{\prime }} \\ \end{aligned} $$

with some appropriately chosen $s_{{j{\text{TT}}}} \in [s_{j} ,s_{{{\text{cid}}(j)}} ]$ and

K.13 $$ C_{\text{Fzsn}}^{{\prime }} = \hbox{max} \left\{ {\left| {\frac{{\partial^{3} F(z;{\mathbf{s}}^{{\prime }} ;{\mathbf{n}}^{{\prime }} )}}{{\partial z\partial s_{j} \partial n_{j} }} - \frac{{\partial^{3} F(z;{\mathbf{s}}^{{\prime }} ;{\mathbf{n}}^{{\prime }} )}}{{\partial z\partial s_{j} \partial n_{{{\text{cid}}(j)}} }}} \right|_{{z = s_{{j{\text{T}}}} ,s_{j} = s_{{j{\text{TT}}}} }} \left| \begin{aligned} j = 1, \ldots ,N + 1, \, {\mathbf{n}}^{{\prime }} \in [0,\eta ]^{N + 1} , \hfill \\ s_{{j{\text{T}}}} \in [s_{{{\text{cid}}(j)}} ,s_{j} ],s_{{j{\text{TT}}}} \in [s_{{{\text{cid}}(j)}} ,s_{j} ] \hfill \\ \end{aligned} \right.} \right\} . $$

From Eqs. (K.10) and (K.12), we find

K.14 $$ \begin{aligned} \left| {\frac{{\partial^{2} F(z;{\mathbf{s}}^{{\prime }} ;{\mathbf{n}}^{{\prime }} )}}{{\partial {\mathbf{m}}^{{\prime }} \partial z}}} \right|_{{z = s_{{j{\text{T}}}} }} = \left| {\left( {\begin{array}{*{20}c} {\frac{{\partial^{2} F(s_{i} ;{\mathbf{s}}^{{\prime }} ;{\mathbf{n}}^{{\prime }} )}}{{\partial m_{1} \partial z}}} \\ \ldots \\ {\frac{{\partial^{2} F(s_{i} ;{\mathbf{s}}^{{\prime }} ;{\mathbf{n}}^{{\prime }} )}}{{\partial m_{M} \partial z}}} \\ {\frac{{\partial^{2} F(s_{i} ;{\mathbf{s}}^{{\prime }} ;{\mathbf{n}}^{{\prime }} )}}{{\partial m_{M + 1} \partial z}}} \\ \ldots \\ {\frac{{\partial^{2} F(s_{i} ;{\mathbf{s}}^{{\prime }} ;{\mathbf{n}}^{{\prime }} )}}{{\partial m_{N + 1} \partial z}}} \\ \end{array} } \right)} \right|_{{z = s_{{j{\text{T}}}} }} \\ \le \left| {\left( {\begin{array}{*{20}c} {C_{\text{Fzn}}^{{\prime }} } \\ \ldots \\ {C_{\text{Fzn}}^{{\prime }} } \\ {C_{\text{Fzsn}}^{{\prime }} } \\ \ldots \\ {C_{\text{Fzsn}}^{{\prime }} } \\ \end{array} } \right)} \right| = \sqrt {MC_{\text{Fzn}}^{{{\prime }2}} + (N + 1 - M)C_{\text{Fzsn}}^{{{\prime }2}} } : = C_{{{\text{Fz}}{\mathbf{m}}}}^{{\prime }} .\end{aligned} $$

## Appendix L: Derivation of functional response and error estimates in Sect. 7

### L.1 Functional response

For an arbitrary resource distribution expressed along a resource-quality axis $w$ (size, hardness, toxicity, nutrient composition, etc.), incorporation of interference competition into Holling’s disc equation (Holling 1959) gives a generalized Beddington–deAngelis-type functional response,

L.1 $$ g(s_{i} ;{\mathbf{s}};{\mathbf{n}}) = \frac{{\int {R(w)c(w,s_{i} ){\text{d}}w} }}{{\zeta_{1} + \zeta_{2} \int {R(w)c(w,s_{i} ){\text{d}}w} + \zeta_{3} \int {C(w)c(w,s_{i} ){\text{d}}w} }} , $$

(Ito et al. 2009; Ito and Dieckmann 2012), where $R(w)$ is the resource distribution and $c(w,s_{i} )$ describes the niche of a phenotype $s_{i}$, in the form of the individual consumption effort as a function of the resource quality $w$. $C(w)$ is the consumption-effort distribution along the resource-quality axis $w$ invested by all existing phenotypes,

L.2 $$ C(w) = \sum\limits_{i = 1}^{N} {n_{i} c(w,s_{i} )} . $$

Notice that the derivation of Eq. (L.1) in Ito et al. (2009) implicitly assumes that an individual searching resources engages in interference competition not only with the other individuals searching resources but also with individuals handling resources. Even when interference competition occurs only among individuals searching resources, a functional response can be derived by applying the “Holling square argument” (Heesterbeek and Metz 1993), but in a different form from Eq. (L.1). Eq. (L.1) can be transformed into

L.3 $$ \begin{aligned} g(s_{i} ;{\mathbf{s}};{\mathbf{n}}) & = \frac{{\theta (s_{i} )}}{{\zeta_{1} + \zeta_{2} \theta (s_{i} ) + \zeta_{3} \sum\nolimits_{j = 1}^{N} {n_{j} \alpha (s_{j} ,s_{i} )} }}, \\ \alpha (s_{j} ,s_{i} ) & = \int {c(w,s_{j} )c(w,s_{i} ){\text{d}}w} , \\ \theta (s_{i} ) & = \int {R(w)c(w,s_{i} ){\text{d}}w} . \\ \end{aligned}  $$

### L.2 Derivation of $\eta$, $C_{{\mathbf{h}}}$, and $C_{{\mathbf{r}}}$

As for $\eta$, we see from Eqs. (7.1) and (7.2) in the main text that for any $i$

L.4 $$ \begin{aligned} \frac{1}{{n_{i} }}\frac{{{\text{d}}n_{i} }}{{{\text{d}}t}} & = \frac{{\beta \theta (s_{i} )}}{{\zeta_{1} + \zeta_{2} \theta (s_{i} ) + \sum\nolimits_{j = 1}^{2} {n_{j} \alpha (s_{j} ,s_{i} )} }} - 1 \\ & \le \frac{{\beta \theta (s_{i} )}}{{\zeta_{1} + \zeta_{2} \theta (s_{i} ) + n_{i} }} - 1, \\ \end{aligned} $$

which is negative whenever $n_{i}$ satisfies

L.5 $$ n_{i} > [\beta - \zeta_{2} ]C_{\theta } - \zeta_{1} , $$

with $C_{\theta } = \hbox{max} \left\{ {\theta (s)\left| {s \in [s_{1} ,s_{2} ]} \right.} \right\}$. Thus, we find

L.6 $$ \eta = [\beta - \zeta_{2} ]C_{\theta } - \zeta_{1} . $$

As for $C_{{\mathbf{r}}}$, we see from Table 2 that

L.7 $$ \begin{aligned} C_{{\mathbf{r}}} & = \left\| {\mathbf{P}} \right\|\left\| {{\mathbf{P}}^{ - 1} } \right\|^{2} \left[ {\left\| {\mathbf{B}} \right\| + \eta \sqrt M C_{{{\text{F}}{\mathbf{mm}}}} } \right] \\ & = \frac{1}{{\beta \theta (s_{1} )}} + \eta C_{{{\text{F}}{\mathbf{mm}}}} , \\ \end{aligned} $$

since ${\mathbf{P}} = 1$, $M = 1$, and ${\mathbf{B}} = b_{11} = - 1 /[\beta \theta (s_{1} )]$. In addition, we see from Table 2 that

L.8 $$ \begin{aligned} C_{{{\text{F}}{\mathbf{mm}}}} & = \hbox{max} \left\{ {\left. {\left\| {\frac{{\partial^{2} F(s_{i} ;{\mathbf{s}}_{\text{a}} ;{\mathbf{m}})}}{{\partial {\mathbf{m}}\partial {\mathbf{m}}^{\text{T}} }}} \right\|_{\text{Q}} } \right|\;i = 1, \ldots ,M,\;{\mathbf{m}} \in [0,\eta ]^{M} } \right\} \\ & = \hbox{max} \left\{ {\left. {\left| {\frac{{\partial^{2} F(s_{1} ;s_{1} ;n_{1} )}}{{\partial n_{1}^{2} }}} \right|} \right|\;n_{1} \in [0,\eta ]} \right\} \\ & = \hbox{max} \left\{ {\left. {\left| {\frac{{2\beta \theta (s_{1} )}}{{[\zeta_{1} + \zeta_{2} \theta (s_{1} ) + n_{1} ]^{3} }}} \right|} \right|\;n_{1} \in [0,\eta ]} \right\} \\ & = \frac{{2\beta \theta (s_{1} )}}{{[\zeta_{1} + \zeta_{2} \theta (s_{1} )]^{3} }}, \\ \end{aligned} $$

which upon substitution into Eq. (L.7) gives

L.9 $$ C_{{\mathbf{r}}} = \frac{1}{{\beta \theta (s_{1} )}} + \eta \frac{{2\beta \theta (s_{1} )}}{{[\zeta_{1} + \zeta_{2} \theta (s_{1} )]^{3} }}. $$

As for $C_{{\mathbf{h}}}$, we see from Table 2 that

L.10 $$ \begin{aligned} C_{{\mathbf{h}}} & = \left\| {\mathbf{P}} \right\|C_{{{\mathbf{hm}}}} = \left\| {\mathbf{P}} \right\|\eta \sqrt M \left[ {\overset{\lower0.5em\hbox{$\smash{\scriptscriptstyle\frown}$}}{C_{\text{Fz}}^{{\prime }}} + 2C_{{{\text{F}}\varepsilon }}^{{\prime }} } \right] \\ & = \eta \left[ {\overset{\lower0.5em\hbox{$\smash{\scriptscriptstyle\frown}$}}{C_{\text{Fz}}^{{\prime }}} + 2C_{{{\text{F}}\varepsilon }}^{{\prime }} } \right]. \\ \end{aligned} $$

For $$ \overset{\lower0.5em\hbox{$\smash{\scriptscriptstyle\frown}$}}{C_{\text{Fz}}^{{\prime }}}, $$ we see from Table 2 that

L.11 $$ \begin{aligned} \overset{\lower0.5em\hbox{$\smash{\scriptscriptstyle\frown}$}}{C_{\text{Fz}}^{{\prime }}} & = \hbox{max} \left\{ {\left. {\left| {\frac{{\partial F(z_{j} ;{\mathbf{s}}^{{\prime }} ;{{\overset{\lower0.5em\hbox{$\smash{\scriptscriptstyle\frown}$}}{{\mathbf{n}}^{{\prime }}} }} )}}{{\partial z_{j} }}} \right|_{{z_{j} = s_{{j{\text{T}}}} }} } \right|\;j = M + 1, \ldots ,N + 1,\,\,s_{{j{\text{T}}}} \in [s_{j} ,s_{{{\text{cid}}(j)}} ]} \right\} \\ & = \hbox{max} \left\{ {\left| {\frac{{\partial F(z_{2} ;{\mathbf{s^{\prime}}};{{\overset{\lower0.5em\hbox{$\smash{\scriptscriptstyle\frown}$}}{{\mathbf{n}}^{\prime}} }})}}{{\partial z_{2} }}} \right|_{{z_{2} \in [s_{1} ,s_{2} ]}} } \right\} \\ & = \hbox{max} \left\{ {\left| {\frac{\partial }{{\partial z_{2} }}\left[ {\frac{{\beta \theta (z_{2} )}}{{\zeta_{1} + \zeta_{2} \theta (s_{1} ) + \overset{\lower0.5em\hbox{$\smash{\scriptscriptstyle\frown}$}}{n}_{1} \alpha (z_{2} ,s_{1} )}} - 1} \right]} \right|_{{z_{2} \in [s_{1} ,s_{2} ]}} } \right\} \\ & = \hbox{max} \left\{ {\left| {\frac{{\beta \frac{{{\text{d}}\theta (z_{2} )}}{{{\text{d}}z_{2} }}}}{{\zeta_{1} + \zeta_{2} \theta (s_{1} ) + \overset{\lower0.5em\hbox{$\smash{\scriptscriptstyle\frown}$}}{n}_{1} \alpha (z_{2} ,s_{1} )}} + \frac{{\beta \theta (z_{2} )[ - \overset{\lower0.5em\hbox{$\smash{\scriptscriptstyle\frown}$}}{n}_{1} (z_{2} - s_{1} )\alpha (z_{2} ,s_{1} )]}}{{[\zeta_{1} + \zeta_{2} \theta (s_{1} ) + \overset{\lower0.5em\hbox{$\smash{\scriptscriptstyle\frown}$}}{n}_{1} \alpha (z_{2} ,s_{1} )]^{2} }}} \right|_{{z_{2} \in [s_{1} ,s_{2} ]}} } \right\} \\ & \le \hbox{max} \left\{ {\left| {\frac{{\beta \frac{{{\text{d}}\theta (z_{2} )}}{{{\text{d}}z_{2} }}}}{{\zeta_{1} + \zeta_{2} \theta (s_{1} )}}} \right|_{{z_{2} \in [s_{1} ,s_{2} ]}} + \left| {\frac{{\beta \theta (z_{2} )\varepsilon }}{{\zeta_{1} + \zeta_{2} \theta (s_{1} )}}} \right|_{{z_{2} \in [s_{1} ,s_{2} ]}} } \right\} \\ & \le \frac{{\beta C_{\partial \theta } }}{{\zeta_{1} + \zeta_{2} \theta (s_{1} )}} + \frac{{\beta C_{\theta } \varepsilon }}{{\zeta_{1} + \zeta_{2} \theta (s_{1} )}}, \\ \end{aligned} $$

with $C_{\theta } = \hbox{max} \left\{ {\theta (s)\left| {s \in [s_{1} ,s_{2} ]} \right.} \right\}$ and $C_{\partial \theta } = \hbox{max} \left\{ {{\text{d}}\theta (s) / {\text{d}}s\left| {s \in [s_{1} ,s_{2} ]} \right.} \right\}$. For $C_{{{\text{F}}\varepsilon }}^{{\prime }}$, we see from Table 2 that

L.12 $$ \begin{aligned} C_{{{\text{F}}\varepsilon }}^{\prime } & = \hbox{max} \left\{ {\left| {\frac{{\partial F(s_{{{\text{cid}}(j)}} + \varepsilon \rho_{j} ;{\mathbf{s}}_{\text{a}}^{\prime } + \varepsilon {\varvec{\uprho}}^{\prime } ;{\mathbf{n}}^{\prime } )}}{\partial \varepsilon }} \right|_{{\varepsilon = \varepsilon_{{{\text{T}}j}} }} \left| {\begin{array}{*{20}l} {j = 1, \ldots ,N + 1,} \hfill \\ {{\mathbf{n}}^{{\prime }} \in [0,\eta ]^{N + 1} ,} \hfill \\ {\varepsilon_{{{\text{T}}j}} \in [0,\varepsilon ]} \hfill \\ \end{array} } \right.} \right\} \\ & = \hbox{max} \left\{ {\left| {\frac{{\partial F(s_{{{\text{cid}}(j)}} + \varepsilon \rho_{j} ;(s_{1} ,s_{1} + \varepsilon )^{\text{T}} ;(n_{1} ,n_{2} )^{\text{T}} )}}{\partial \varepsilon }} \right|_{{\varepsilon = \varepsilon_{\text{T}} }} \left| {\begin{array}{*{20}l} {j = 1,2,} \hfill \\ {0 \le n_{1} \le \eta ,\;0 \le n_{2} \le \eta ,} \hfill \\ {\varepsilon_{\text{T}} \in [0,\varepsilon ]} \hfill \\ \end{array} } \right.} \right\}, \\ \end{aligned} $$

where for $j = 1$ we see from Eqs. (7.4a) in the main text that

L.13 $$ \begin{aligned}    & \left| {\frac{{\partial F(s_{1} ;(s_{1} ,s_{1}  + \varepsilon )^{{\text{T}}} ;(n_{1} ,n_{2} )^{{\text{T}}} )}}{{\partial \varepsilon }}} \right| \\     & \quad  = \left| {\frac{\partial }{{\partial \varepsilon }}\left[ {\frac{{\beta \theta (s_{1} )}}{{\zeta _{1}  + \zeta _{2} \theta (s_{1} ) + n_{1}  + n_{2} \alpha (s_{1}  + \varepsilon ,s_{1} )}}} \right]} \right| \\     & \quad  = \frac{{\beta \theta (s_{1} )\varepsilon n_{2} \alpha (s_{1}  + \varepsilon ,s_{1} )}}{{[\zeta _{1}  + \zeta _{2} \theta (s_{1} ) + n_{1}  + n_{2} \alpha (s_{1}  + \varepsilon ,s_{1} )]^{2} }} \\     & \quad  \le \frac{{\beta \theta (s_{1} )\varepsilon }}{{\zeta _{1}  + \zeta _{2} \theta (s_{1} ) + n_{1}  + n_{2} \alpha (s_{1}  + \varepsilon ,s_{1} )}} \\     & \quad  \le \frac{{\varepsilon \beta C_{\theta } }}{{\zeta _{1}  + \zeta _{2} C_{{\theta {\text{min}}}} }} \\  \end{aligned}  $$

with $C_{\theta \hbox{min} } = \hbox{min} \left\{ {\theta (s)\left| {s \in [s_{1} ,s_{2} ]} \right.} \right\}$, and for $j = 2$ we see analogously

L.14 $$ \begin{aligned} & \left| {\frac{{\partial F(s_{1} + \varepsilon ;(s_{1} ,s_{1} + \varepsilon )^{\text{T}} ;(n_{1} ,n_{2} )^{\text{T}} )}}{\partial \varepsilon }} \right| \\& = \left| {\frac{\partial }{\partial \varepsilon }\left[ {\frac{{\beta }}{{\zeta_{1} + \zeta_{2} \theta (s_{1} + \varepsilon ) + n_{1} \alpha (s_{1} + \varepsilon ,s_{1} ) + n_{2} }}} \right]} \right| \\ & \le \left[ {\frac{{\beta}}{{\zeta_{1} + \zeta_{2} \theta (s_{1} + \varepsilon ) + n_{1} \alpha (s_{1} + \varepsilon ,s_{1} ) + n_{2} }}  + \frac{{\beta \theta (s_{1} + \varepsilon )\zeta_{2} }}{{[\zeta_{1} + \zeta_{2} \theta (s_{1} + \varepsilon ) + n_{1} \alpha (s_{1} + \varepsilon ,s_{1} ) + n_{2} ]^{2} }}} \right]\left| {\frac{{\partial \theta (s_{1} + \varepsilon )}}{\partial \varepsilon }} \right| \\ & \quad \quad + \,\frac{{\beta \theta (s_{1} + \varepsilon )n_{1} \varepsilon \alpha (s_{1} + \varepsilon ,s_{1} )}}{{[\zeta_{1} + \zeta_{2} \theta (s_{1} + \varepsilon ) + n_{1} \alpha (s_{1} + \varepsilon ,s_{1} ) + n_{2} ]^{2} }} \\ & \le \left[ {\frac{\beta }{{\zeta_{1} + \zeta_{2} \theta (s_{1} + \varepsilon ) + n_{1} \alpha (s_{1} + \varepsilon ,s_{1} ) + n_{2} }} + \frac{\beta }{{\zeta_{1} + \zeta_{2} \theta (s_{1} + \varepsilon ) + n_{1} \alpha (s_{1} + \varepsilon ,s_{1} ) + n_{2} }}} \right]C_{\partial \theta } \\ & \quad \quad + \frac{{\beta \theta (s_{1} + \varepsilon )\varepsilon }}{{\zeta_{1} + \zeta_{2} \theta (s_{1} + \varepsilon ) + n_{1} \alpha (s_{1} + \varepsilon ,s_{1} ) + n_{2} }} \\ & \le \frac{\beta }{{\zeta_{1} + \zeta_{2} \theta (s_{1} + \varepsilon )}}C_{\partial \theta } + \frac{\beta }{{\zeta_{1} + \zeta_{2} \theta (s_{1} + \varepsilon )}}C_{\partial \theta } \\ &+ \frac{{\beta \theta (s_{1} + \varepsilon )\varepsilon }}{{\zeta_{1} + \zeta_{2} \theta (s_{1} + \varepsilon )}} \le \frac{{\beta [2C_{\partial \theta } + \varepsilon C_{\theta } ]}}{{\zeta_{1} + \zeta_{2} C_{\theta {\rm min} } }} \\ \end{aligned} $$

Combining Eqs. (L.6), (L.11), (L.12), (L.13), and (L.14), we find

L.15 $$ \begin{aligned} C_{{\mathbf{h}}} & = \eta \left[ {\overset{\lower0.5em\hbox{$\smash{\scriptscriptstyle\frown}$}}{C_{\text{Fz}}^{{\prime }}} + 2C_{{{\text{F}}\varepsilon }}^{{\prime }} } \right] \\ & \le \eta \left[ {\frac{{\beta C_{\partial \theta } }}{{\zeta_{1} + \zeta_{2} \theta (s_{1} )}} + \frac{{\beta C_{\theta } \varepsilon }}{{\zeta_{1} + \zeta_{2} \theta (s_{1} )}} + 2\hbox{max} \left\{ {\frac{{\varepsilon \beta C_{\theta } }}{{\zeta_{1} + \zeta_{2} C_{\theta {\rm min} } }},\frac{{\beta [2C_{\partial \theta } + \varepsilon C_{\theta } ]}}{{\zeta_{1} + \zeta_{2} C_{\theta {\rm min} } }},} \right\}} \right] \\ & \le \eta \left[ {\frac{{\beta C_{\partial \theta } + \beta C_{\theta } \varepsilon }}{{\zeta_{1} + \zeta_{2} C_{\theta {\rm min} } }} + \frac{{2\beta [2C_{\partial \theta } + \varepsilon C_{\theta } ]}}{{\zeta_{1} + \zeta_{2} C_{\theta {\rm min} } }}} \right] \\ & \le \frac{{\eta \beta [5C_{\partial \theta } + 3\varepsilon C_{\theta } ]}}{{\zeta_{1} + \zeta_{2} C_{\theta {\rm min} } }} \\ \end{aligned} $$

with $$ C_{\theta } = \hbox{max} \left\{ {\theta (s)\left| {s \in [s_{1} ,s_{2} ]} \right.} \right\} $$, $$ C_{\theta {\rm min} } = \hbox{min} \left\{ {\theta (s)\left| {s \in [s_{1} ,s_{2} ]} \right.} \right\} $$, and $$ C_{\partial \theta } = \hbox{max} \left\{ {{\text{d}}\theta (s) / {\text{d}}s\left| {s \in [s_{1} ,s_{2} ]} \right.} \right\}. $$
